# ﻿A faunal inventory of methane seeps on the Pacific margin of Costa Rica

**DOI:** 10.3897/zookeys.1222.134385

**Published:** 2025-01-03

**Authors:** Charlotte A. Seid, Avery S. Hiley, Marina F. McCowin, José I. Carvajal, Harim Cha, Shane T. Ahyong, Oliver S. Ashford, Odalisca Breedy, Douglas J. Eernisse, Shana K. Goffredi, Michel E. Hendrickx, Kevin M. Kocot, Christopher L. Mah, Allison K. Miller, Nicolás Mongiardino Koch, Rich Mooi, Timothy D. O'Hara, Fredrik Pleijel, Josefin Stiller, Ekin Tilic, Paul Valentich-Scott, Anders Warén, Mary K. Wicksten, Nerida G. Wilson, Erik E. Cordes, Lisa A. Levin, Jorge Cortés, Greg W. Rouse

**Affiliations:** 1 Scripps Institution of Oceanography, University of California San Diego, La Jolla, California, USA University of California San Diego La Jolla United States of America; 2 Australian Museum, Sydney, New South Wales, Australia Australian Museum Sydney Australia; 3 University of New South Wales, Kensington, New South Wales, Australia University of New South Wales Kensington Australia; 4 Ocean Program, World Resources Institute, London, UK Ocean Program, World Resources Institute London United Kingdom; 5 Universidad de Costa Rica, San José, Costa Rica University of Costa Rica San José Costa Rica; 6 California State University Fullerton, Fullerton, California, USA California State University Fullerton Fullerton United States of America; 7 Occidental College, Los Angeles, California, USA Occidental College Los Angeles United States of America; 8 Instituto de Ciencias del Mar y Limnología, Universidad Nacional Autónoma de México, Mazatlán, Sinaloa, Mexico Universidad Nacional Autónoma de México Mazatlán Mexico; 9 University of Alabama, Tuscaloosa, Alabama, USA University of Alabama Tuscaloosa United States of America; 10 Smithsonian National Museum of Natural History, Washington, DC, USA Smithsonian National Museum of Natural History Washington United States of America; 11 University of Otago, Dunedin, New Zealand University of Otago Dunedin New Zealand; 12 California Academy of Sciences, San Francisco, California, USA California Academy of Sciences San Francisco United States of America; 13 Museums Victoria, Melbourne, Victoria, Australia Museums Victoria Melbourne Australia; 14 University of Gothenburg, Gothenburg, Sweden University of Gothenburg Gothenburg Sweden; 15 University of Copenhagen, Copenhagen, Denmark University of Copenhagen Copenhagen Denmark; 16 Senckenberg Research Institute and Natural History Museum, Frankfurt, Germany Senckenberg Research Institute and Natural History Museum Frankfurt Germany; 17 Santa Barbara Museum of Natural History, Santa Barbara, California, USA Santa Barbara Museum of Natural History Santa Barbara United States of America; 18 Swedish Museum of Natural History, Stockholm, Sweden Swedish Museum of Natural History Stockholm Sweden; 19 Texas A&M University, College Station, Texas, USA Texas A&M University Texas United States of America; 20 Collections & Research, Western Australian Museum, Welshpool, Western Australia, Australia Western Australian Museum Welshpool Australia; 21 School of Biological Sciences, University of Western Australia, Perth, Western Australia, Australia University of Western Australia Perth Australia; 22 Temple University, Philadelphia, Pennsylvania, USA Temple University Philadelphia United States of America

**Keywords:** Biodiversity, biogeography, Central America, chemosynthetic ecosystem, COI, deep sea, DNA ‘barcodes’, molecular taxonomy, review

## Abstract

The methane seeps on the Pacific margin of Costa Rica support extensive animal diversity and offer insights into deep-sea biogeography. During five expeditions between 2009 and 2019, we conducted intensive faunal sampling via 63 submersible dives to 11 localities at depths of 300–3600 m. Based on these expeditions and published literature, we compiled voucher specimens, images, and 274 newly published DNA sequences to present a taxonomic inventory of macrofaunal and megafaunal diversity with a focus on invertebrates. In total 488 morphospecies were identified, representing the highest number of distinct morphospecies published from a single seep or vent region to date. Of these, 131 are described species, at least 58 are undescribed species, and the remainder include some degree of taxonomic uncertainty, likely representing additional undescribed species. Of the described species, 38 are known only from the Costa Rica seeps and their vicinity. Fifteen range extensions are also reported for species known from Mexico, the Galápagos seamounts, Chile, and the western Pacific; as well as 16 new depth records and three new seep records for species known to occur at vents or organic falls. No single evolutionary narrative explains the patterns of biodiversity at these seeps, as even morphologically indistinguishable species can show different biogeographic affinities, biogeographic ranges, or depth ranges. The value of careful molecular taxonomy and comprehensive specimen-based regional inventories is emphasized for biodiversity research and monitoring.

## ﻿﻿Introduction

The Costa Rica margin (CRM) occupies a central position in the biogeographic and tectonic landscape of the eastern Pacific. The subduction of the Cocos Plate beneath the Caribbean Plate at the Middle America Trench gives rise to deep-sea methane seeps (hereafter called “seeps”) (Kahn et al. 1996; McAdoo et al. 1996; Zuleger et al. 1996), as are found on the neighboring continental margins from Oregon to Chile (Sibuet and Olu 1998; Van Dover et al. 2002; Levin et al. 2016). The boundaries of the Cocos Plate adjoin the hydrothermal vent fields of the East Pacific Rise and the Galápagos Rift, which also directly links to the CRM via the Coco Submarine Volcanic Range (Kimura et al. 1997; von Huene et al. 2000; Fiedler and Lavín 2006; Beaulieu et al. 2013). This geographic position suggests the potential for population connectivity to seeps north and south along the continental margins, to the tectonically adjacent vent fields, and to the central and western Pacific via deep-ocean equatorial circulation (Tunnicliffe et al. 1998; Van Dover et al. 2002; Bachraty et al. 2009; Miloslavich et al. 2011). Furthermore, the biogeographic history of the CRM has been influenced by the emergence of the Isthmus of Panama (Central American Isthmus) (Cortés and Wehrtmann 2009), from the closure of deep-water exchange to the western Atlantic approximately 9 million years ago to the formation of the land barrier ~ 2.8 million years ago (O’Dea et al. 2016). The faunal diversity of the CRM thus offers a range of insights into deep-sea biogeography and evolution.

**Figure 1.**
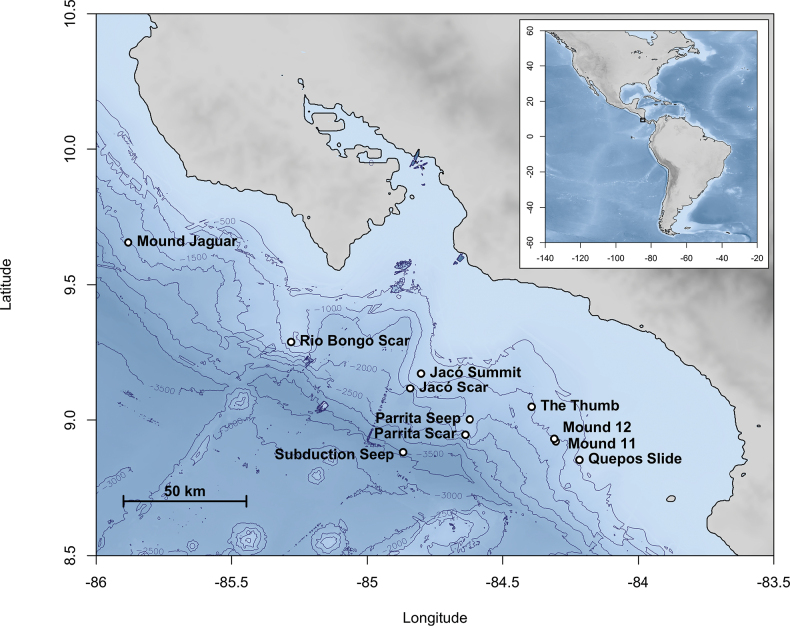
Map of the Costa Rica seep sites sampled in this study. Maps were generated using the R package marmap (Pante and Simon-Bouhet 2013) and bathymetric data from NOAA (https://www.ncei.noaa.gov/maps/autogrid/, accessed 25 August 2021).

Active seepage of methane-rich fluids occurs at more than 100 sites located ca 50 km offshore along the CRM, from southern Nicaragua to the Osa Peninsula (Kahn et al. 1996; McAdoo et al. 1996; Zuleger et al. 1996; Kimura et al. 1997; Bohrmann et al. 2002; Sahling et al. 2008). The seeps occur at depths of ~ 400–3800 m at diverse geological features including mounds, faults, seamount subduction scars, and landslides (Kahn et al. 1996; McAdoo et al. 1996; Bohrmann et al. 2002; Sahling et al. 2008). The sites studied in this work are shown in Fig. 1. The seeps also span hydrographic gradients. Between 400 and 1800 m, the temperature ranges from 9.5 to 2.7 °C and oxygen concentration ranges from 0.04 to 1.9 ml/l, with an oxygen minimum zone at 300–600 m (Bohrmann et al. 2002; Levin et al. 2015). Methane concentrations vary with depth and among sites across three orders of magnitude, from background levels of ~ 2 nmol/l to maximum levels of 75–1506 nmol/l (Bohrmann et al. 2002; Mau et al. 2014). These abiotic gradients invite careful comparisons of biodiversity across the ensemble of seep sites.

**Figure 2.**
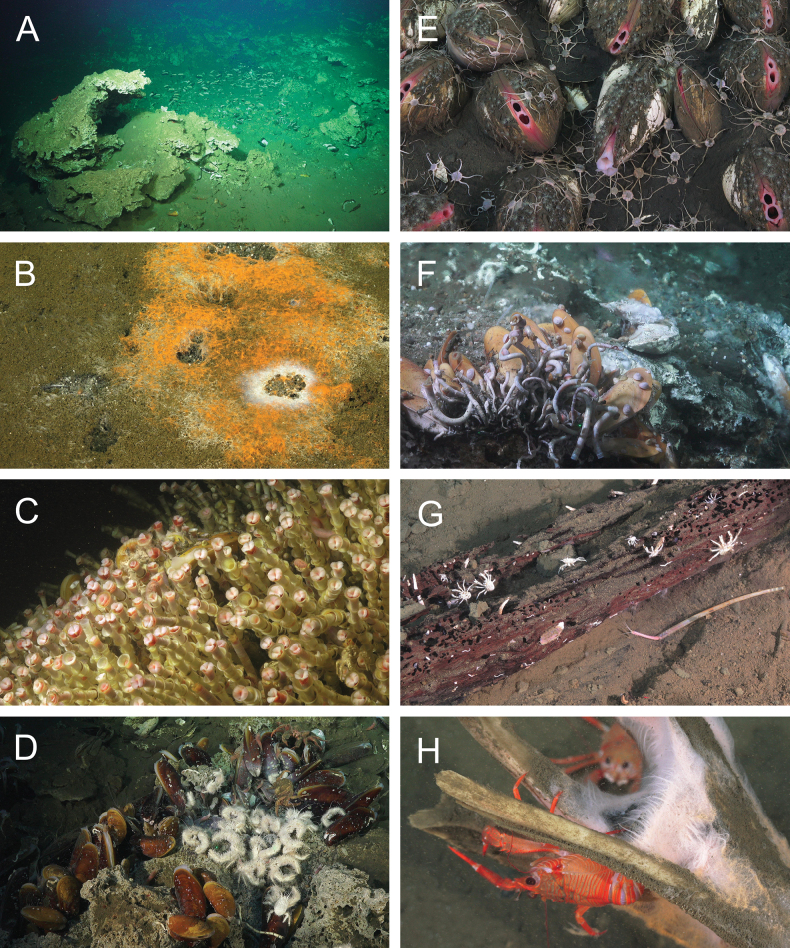
Diversity of habitats at the Costa Rica seeps. Credit: ROV SuBastian/Schmidt Ocean Institute **A** authigenic carbonates at Mound 12 (1006 m, Dive S0215) **B** microbial mat at Rio Bongo Scar (609 m, Dive S0219) **C** vestimentiferan tubeworm aggregation (predominantly *Lamellibrachiabarhami*) at Jacó Scar (1814 m, Dive S0212) **D** mussels (*Bathymodiolusnancyschneiderae*) and yeti crabs (*Kiwapuravida*) on authigenic carbonates at Mound 12 (1006 m, Dive S0215) **E** vesicomyid clams at Jacó Scar (1781 m, Dive S0214) **F** seepage of higher-temperature fluid at the Jacó Scar hydrothermal seep site (1803 m, Dive S0214) **G** wood fall at Jacó Scar (1875 m, Dive S0214) **H** animal fall (billfish skull, utilized by *Grimotheamonodon* squat lobsters) at Quepos Slide (403 m, Dive S0216).

The first submersible dives at the CRM in 1994 revealed biological indicators of chemosynthetic activity, namely the presence of authigenic carbonates, microbial mats, and symbiont-bearing invertebrate megafauna (Fig. 2A–E) (Kahn et al. 1996; McAdoo et al. 1996). Authigenic carbonates are precipitated via anaerobic oxidation of methane (Ritger et al. 1987), in which a consortium of methane-oxidizing (methanotrophic) archaea and sulfate-reducing bacteria generates bicarbonate and hydrogen sulfide (Boetius et al. 2000; Orphan et al. 2001). Hydrogen sulfide, in turn, supports chemosynthesis by sulfur-oxidizing (thiotrophic) microbes (Tunnicliffe et al. 2003). At the CRM seeps, thiotrophic bacteria constitute many of the microbial mats (Mau et al. 2006, 2014; Bailey et al. 2011; Bernardino et al. 2012; Niemann et al. 2013) and play an important role as nutritional symbionts of vestimentiferan tubeworms, bathymodiolin mussels, and vesicomyid clams (Mau et al. 2006; Brzechffa and Goffredi 2021). As at other seeps (Suess et al. 1985; Sibuet and Olu 1998), these large-bodied symbiont-bearing invertebrate groups tend to dominate the biomass, and much of the initial biological characterization of the CRM seeps focused on these conspicuous megafauna (Kahn et al. 1996; Peek et al. 2000; Bohrmann et al. 2002; Goffredi et al. 2003).

The CRM seeps also harbor a variety of animals that do not directly depend on chemosynthetic symbionts for nutrition. Initial records of such non-obligate seep fauna included limpets, snails, crabs, galatheoids, crinoids, actiniarians, corals, ophiuroids, echinoids, holothuroids, sponges, and macrurid fish (Kahn et al. 1996; Bohrmann et al. 2002). Further sampling has revealed diverse macrofaunal assemblages associated with carbonates and sediments at different habitat types (e.g., Levin et al. 2012, 2015; Ashford et al. 2021b; Pereira et al. 2021). To date, 48 animal species have been described from the CRM seeps, including annelids (Aguado and Rouse 2011; Borda et al. 2013; Summers et al. 2014b; Watson et al. 2016; McCowin and Rouse 2018; Rouse et al. 2018; Lindgren et al. 2019; Hatch et al. 2020; Salazar-Vallejo 2020a; Yen and Rouse 2020; Rouse and Kupriyanova 2021; Pearson and Rouse 2022; Villalobos-Guerrero et al. 2024), corals (Opresko and Breedy 2010; Breedy et al. 2019), crustaceans (Thurber et al. 2011; Martin et al. 2018; Rodríguez-Flores et al. 2023), fish (Frable et al. 2023), echinoderms (Payne et al. 2023), mollusks (Barry and Kochevar 1999; Krylova and Sahling 2006; Martin and Goffredi 2012; Warén and Rouse 2016; McCowin et al. 2020), and nemerteans (Sagorny et al. 2022), as well as a ciliate (Pasulka et al. 2017) for a total of 49 eukaryotic species.

Furthermore, the CRM seeps show environmental and biological connections to other chemosynthesis-based habitats. The Jacó Scar “hydrothermal seep” site at 1800 m depth (Fig. 2F) appears to represent an intermediate environment between seeps and hydrothermal vents, as elevated water temperatures (up to 5.2 °C, representing nearly 3 °C above ambient) support certain vent-affiliated fauna (Levin et al. 2012). Wood falls (Fig. 2G) and animal carcasses (Fig. 2H) occur in the vicinity of these seeps, as might be expected from their proximity to a forested coast and its river discharges. Experimental deployments of wood and bone substrates have enabled further investigation of seep-adjacent organic fall communities (Hatch et al. 2020; Pereira et al. 2022; Payne et al. 2023). These intersectional habitats offer the opportunity to address questions of colonization, habitat suitability, and biogeography across different chemosynthesis-based ecosystems.

During five research cruises to the CRM in 2009–2019, we used deep submergence vehicles to conduct intensive faunal sampling. Based on these expeditions and published literature, we compiled voucher specimen records, images, and DNA sequences to present a taxonomic inventory of faunal diversity at these seeps. We report new range extensions and depth records, discuss connections to other chemosynthesis-based ecosystems, and assess biogeographic patterns.

## ﻿﻿Materials and methods

### ﻿﻿Sampling locations

Specimens were collected during five research cruises to the central CRM (Fig. 1): R/V Atlantis with DSV Alvin AT15-44 (2009), AT15-59 (2010), AT37-13 (2017), AT42-03 (2018); R/V Falkor with ROV SuBastian FK190106 (2019). Collection localities, depths, dates, and details are summarized in Table 1.

**Table 1.** Submersible dives and collection events. AD = HOV Alvin, R/V Atlantis. S = ROV SuBastian, R/V Falkor. MC = multicore. Multicores and plankton tows were deployed from R/V Atlantis. Coordinates reflect dive summaries as reported in the HOV Alvin dive logs (https://ndsf.whoi.edu/data/) and the R/V Falkor FK190106 cruise report. Dates reflect local time. Depth ranges reflect the minimum and maximum depth for sample collection events. In most cases, more precise coordinates and depths for specific animals are available on the SIO-BIC online database (https://sioapps.ucsd.edu/collections/bi/).

Cruise	Dive or deployment	Locality	Date	Latitude, Longitude	Depth, m
AT15-44	AD4501	Mound 12	2009-02-22	8.9300, -84.3135	984–997
	AD4502	Mound 12	2009-02-23	8.9285, -84.3132	987–997
	AD4503	Mound 12	2009-02-24	8.9308, -84.3072	967–995
	AD4504	Mound 11	2009-02-25	8.9208, -84.3054	1004–1011
	AD4505	Mound 11	2009-02-26	8.9198, -84.3055	1019–1025
	AD4506	Parrita Seep	2009-02-27	8.9718, -84.6235	1030–1179
	AD4507	Parrita Scar	2009-02-28	8.9353, -84.6465	1659–1667
	AD4508	Parrita Seep	2009-03-01	9.0303, -84.6230	1401–1419
	AD4509	Jacó Scar and Jacó Slope	2009-03-03	9.1172, -84.8425	974–1856
	AD4510	Jacó Summit	2009-03-04	9.1723, -84.7987	741–744
	AD4511	Mound 12	2009-03-05	8.9305, -84.3123	988–997
	AD4512	Quepos Slide	2009-03-06	8.8536, -84.2181	344–411
	AD4513	Jacó Scar	2009-03-07	9.1167, -84.8351	1744–1818
	MC1-2	Transition site near Mound 12 (~ 500 m from active seep)	2009-02-21	8.9316, -84.3168	1019
AT15-59	AD4586	Mound 12	2010-01-07	8.9308, -84.3130	982–998
	AD4587	Mound 12	2010-01-08	8.9306, -84.3123	990–996
	AD4588	Mound 12	2010-01-09	8.9308, -84.3125	995–997
	AD4589	Mound 12	2010-01-10	8.9298, -84.3121	997
	AD4590	Jacó Scar	2010-01-11	9.1176, -84.8395	1791–1800
	AD4591	Jacó Scar	2010-01-12	9.1182, -84.8391	1752–1795
	MC1	Transition site near Mound 12 (~ 400 m from active seep)	2010-01-06	8.9325, -84.3158	995
	MC4	Transition site near Mound 11 (~ 400 m from active seep)	2010-01-10	8.9208, -84.3016	1031
	Plankton Tow 6	Jacó Summit	2010-01-12	9.1713, -84.7987	0–350 (350 m wire out)
AT37-13	AD4906	Mound 12	2017-05-21	8.9308, -84.3128	995–1002
	AD4907	Mound 12	2017-05-22	8.9304, -84.3128	990–999
	AD4908	Mound 12	2017-05-23	8.9304, -84.3126	989–1001
	AD4909	Mound 12	2017-05-24	8.9305, -84.3125	967–1000
	AD4910	Mound 12	2017-05-25	8.9304, -84.3126	988–1004
	AD4911	Jacó Scar	2017-05-26	9.1151, -84.8468	1757–1892
	AD4912	Jacó Scar	2017-05-27	9.1154, -84.8362	1795–1859
	AD4913	Jacó Scar	2017-05-28	9.1156, -84.8401	1798–1908
	AD4914	Jacó Scar	2017-05-29	9.1175, -84.8395	1632–1886
	AD4915	Jacó Scar	2017-05-30	9.1180, -84.8404	1741–1885
	AD4916	Jacó Scar	2017-05-31	9.1193, -84.8428	1604–1854
	AD4917	Mound 12	2017-06-01	8.9293, -84.3150	965–1000
	AD4918	Quepos Slide	2017-06-02	8.8535, -84.2177	333–408
	AD4919	Quepos Slide	2017-06-03	8.8527, -84.2174	379–410
	AD4921	Quepos Slide	2017-06-04	8.8532, -84.2155	345–394
	AD4922	Mound 12	2017-06-05	8.9296, -84.3078	964–1009
	AD4923	Parrita Seep	2017-06-06	8.9759, -84.6238	1037–1097
	AD4924	Parrita Seep	2017-06-07	9.0305, -84.6202	1400–1410
AT42-03	AD4971	Jacó Scar	2018-10-17	9.1170, -84.8426	1746–1824
	AD4972	Jacó Scar	2018-10-18	9.1164, -84.8403	1746–1845
	AD4973	Jacó Scar	2018-10-19	9.1148, -84.8398	1784–1887
	AD4974	Mound 12	2018-10-20	8.9297, -84.3078	990–1010
	AD4975	Mound 12	2018-10-21	8.9310, -84.3075	988–1002
	AD4976	Jacó Scar	2018-10-22	9.1139, -84.8401	1836–1887
	AD4977	Jacó Scar	2018-10-23	9.1163, -84.8418	1783
	AD4978	Mound 12	2018-10-24	8.9294, -84.3143	996–999
AT42-03	AD4979	Quepos Slide	2018-10-25	8.8539, -84.2178	380–397
	AD4984	Mound 12	2018-10-30	8.9300, -84.3137	964–998
	AD4985	Mound 12	2018-10-31	8.9303, -84.3129	991–1002
	AD4986	Quepos Slide	2018-11-01	8.8540, -84.2195	308–379
	AD4987	Mound 12 West	2018-11-02	8.9292, -84.3167	995–1012
	AD4988	Mound 11	2018-11-03	8.9193, -84.3027	998–1025
	AD4989	Jacó Scar	2018-11-04	9.1174, -84.8417	1758–1792
	AD4990	Parrita Seep	2018-11-05	9.0321, -84.6197	1400–1435
FK190106	S0212	Jacó Scar	2019-01-06	9.1175, -84.8393	1780–1869
	S0213	Jacó Summit	2019-01-06	9.1734, -84.8038	730–820
	S0214	Jacó Scar	2019-01-07	9.1175, -84.8393	1780–1875
	S0215	Mound 12	2019-01-08	8.9307, -84.3126	982–1016
	S0216	Quepos Slide	2019-01-09	8.8539, -84.2193	275–404
	S0217	The Thumb	2019-01-10	9.0486, -84.3945	940–1074
	S0218	Parrita Scar	2019-01-11	8.9498, -84.6381	1110–1988
	S0219	Rio Bongo Scar	2019-01-13	9.2862, -85.2757	480–661
	S0220	Subduction Plume	2019-01-14	8.8785, -84.8695	3399–3601
	S0230	Mound Jaguar	2019-01-25	9.6558, -85.8813	1895–2000

The submersible dives in this study primarily investigated areas of active methane seepage, as indicated by the presence of non-sedimented authigenic carbonates, microbial mats, or symbiont-bearing megafauna (Levin et al. 2015; Pasulka et al. 2017; Ashford et al. 2021b; Pereira et al. 2021). Certain dives also covered adjacent deep-sea habitat, as defined by the absence of these indicators, and “transition” habitat, characterized by partially sedimented authigenic carbonates, the remains of symbiont-bearing bivalves, and reduced density of seep-associated megafauna (Ashford et al. 2021b; Pereira et al. 2021). The complex biological and geochemical influences of seeps are thought to extend for hundreds of meters up to ~ 1 km in three dimensions (Levin et al. 2016; Ashford et al. 2021b), a spatial extent comparable to that covered by a given submersible dive in this study (typically a maximum radius of 1 km, centered at an active seep site). In a recent ecological study of the transition zone between seeps and adjacent deep-sea habitat at the CRM (Ashford et al. 2021b), the distances between accepted “active” and “transition” or “background” habitats ranged from 257–967 m. Therefore, all specimens collected on seep-focused dives and within ~ 500 m of active seepage were considered within the scope of this study, including a few opportunistically collected specimens from habitats that likely represent background or transition zones (e.g., three multicore deployments in Table 1). Non-seep habitats such as seamounts and Quepos Plateau (von Huene et al. 2000) were investigated during some of the same cruises but are outside the scope of this work.

We generally follow the locality names listed in Sahling et al. (2008). The locality named here as Parrita Seep has been variously cited as “Mound Quepos,” “Quepos Seep,” or “Quepos Seep at Parrita Scar” in previous work (e.g., Han et al. 2004; Sahling et al. 2008; Levin et al. 2015; McCowin and Rouse 2018). Based on the geological categorization of this site as a fault-controlled seep rather than a mound (Sahling et al. 2008) and its proximity to a separate site known as Parrita Scar, we follow other studies (McCowin et al. 2020; Rojas-Jimenez et al. 2020; Ashford et al. 2021b; Rouse and Kupriyanova 2021) in synonymizing these names as Parrita Seep.

### ﻿﻿Specimen collection and vouchering

Most specimens were collected during submersible dives using equipment such as hydraulic arms, suction samplers, scoops, push cores, a Bushmaster Jr. device for sampling tubeworm aggregations (Bergquist et al. 2002), and a customized mussel pot device for sampling mussel aggregations (Cordes et al. 2010). Some specimens were collected opportunistically during multicore deployments or plankton tows adjacent to the main study sites. Some specimens were collected from experimentally deployed substrates such as wood, bone, or carbonate rocks, including rocks experimentally transplanted across different zones at the same locality and similar depth (Pereira et al. 2022), but no animals were transplanted between localities. Associations with wood or bone are indicated in each taxonomic listing, and specimens were collected from naturally occurring seep substrates unless otherwise indicated.

Specimens were maintained alive in chilled seawater, treated with an appropriate relaxing agent, and processed following recommended practices for DNA taxonomy of marine invertebrates (Gemeinholzer et al. 2010; Templado et al. 2010; Glover et al. 2015; Rouse et al. 2022). Live specimens were photographed using a handheld camera (e.g., Canon EOS M5, Canon Rebel T1, Nikon D70, Panasonic DMC-TS4, Ricoh WG-4, Ricoh WG-50) or a photomicroscopy station (e.g., a Leica S8 Apo or MZ9.5 stereomicroscope with a camera attachment for a Canon EOS Rebel T3i, T6s, or T6i; EOS M5; or PowerShot G9). Tissue subsamples or small whole specimens were preserved in 95% ethanol for genetic analysis. Voucher specimens for morphological analysis were typically fixed in 10% seawater formalin for at least 24 h, rinsed with fresh water, and transferred to 50% ethanol for long-term archival. For certain taxa, e.g., echinoderms and crustaceans, large voucher specimens were instead treated with 95% ethanol in several washes for at least 24 h each and then transferred to 50% ethanol for long-term archival. Selected specimens or tissues were treated with RNAlater (Ambion, Austin, TX), paraformaldehyde, glutaraldehyde, or osmium tetroxide. Some specimens from the 2009 and 2010 cruises were treated with the glyoxal fixative Prefer (Anatech Ltd., Battle Creek, MI) for both molecular and morphological purposes, but other fixation-preservation strategies were found to be preferable.

Specimens were deposited in the Scripps Institution of Oceanography Benthic Invertebrate Collection (**SIO-BIC**) and the Museo de Zoología, Universidad de Costa Rica (**MZUCR**, invertebrate collections; **UCR**, fish collection).

### ﻿﻿Taxonomic scope

We targeted benthic invertebrate macrofauna (retained on a 300 µm mesh and typically > 1 mm in size) and megafauna due to the nature of our sampling gear, although a few opportunistically collected exceptions such as large nematodes are reported. The meiofaunal (Neira et al. 2018; Gracia C et al. 2020), fungal (Rojas-Jimenez et al. 2020), and microbial communities of the Costa Rica seeps (Bailey et al. 2011; Niemann et al. 2013; Tavormina et al. 2013; Dekas et al. 2014; Case et al. 2015; Pasulka et al. 2017; Brzechffa and Goffredi 2021; Metcalfe et al. 2021) have been explored in other studies. Our sampling was not intended to be quantitative, as related investigations have explored macrofaunal species richness, biomass, density, and other ecological metrics at these seeps (Levin et al. 2012, 2015; Ashford et al. 2021b, 2021a; Pereira et al. 2021, 2022).

We reported only taxa that were linked to physical specimens, given the importance of museum vouchers for genetic characterization, species descriptions, scientific reproducibility, and the principles of ﻿Findable, Accessible, Interoperable, and Reusable (FAIR) research (Howell et al. 2020). Nonetheless, these expeditions also captured images and video of additional fauna (e.g., medusae, cephalopods, and fishes) that were not collected and that may form the basis of future studies.

### ﻿﻿Specimen identification

Following preliminary morphological identification during shipboard processing, specimens were identified to the lowest possible taxonomic level based on genetics and/or morphology. Genetic identification was facilitated by querying sequences against the NCBI GenBank database (Clark et al. 2016) using the nucleotide BLAST (blastn) suite (https://blast.ncbi.nlm.nih.gov/) (Zhang et al. 2000; Boratyn et al. 2013). Biogeography assessments and access to taxonomic literature were facilitated by the Global Biodiversity Information Facility (GBIF) (GBIF: The Global Biodiversity Information Facility 2024) and the Biodiversity Heritage Library (Gwinn and Rinaldo 2009).

Taxonomic uncertainty was expressed using recommended terminology and practices for open nomenclature (Sigovini et al. 2016; Horton et al. 2021). For example, the abbreviation “stet.” (*stetit*) is used to indicate that further identification to a lower taxonomic level was not attempted given the limitations of the specimen or of available taxonomic resources. Uncertainty at a given taxonomic level is indicated by the abbreviation “inc.” (*incerta*, *incertus*, *incertum*). Undescribed species that have been identified with certainty as new to science but have not yet been formally described or referenced in the literature, are notated with a unique alphanumeric code linked to a museum voucher, e.g., “sp. SIO_BIC_A00001” as opposed to a potentially non-unique designation such as “sp. 1”. Some morphospecies are also notated with unique voucher-linked alphanumeric codes, when multiple morphospecies must be distinguished from one another but cannot be matched with certainty to known species.

### ﻿﻿DNA extraction and sequencing

Genomic DNA was extracted following the manufacturer’s protocol for commercial kits such as the DNeasy Tissue Kit (Qiagen); the EZNA Micro-Elute Genomic DNA Kit (Omega Bio-Tek); or the *Quick*-DNA Miniprep, Microprep Plus, or 96 Plus Kit (Zymo Research, Irvine, CA and Tustin, CA). Polymerase chain reaction (PCR) amplification of phylogenetically informative gene fragments was performed using the primer pairs summarized in Table 2. A typical PCR included 1 μl of each primer (10 μM), 2 μl of genomic DNA, and the appropriate concentration of a commercially available reagents such as Apex 2.0x Taq Red DNA Polymerase Master Mix (Genesee Scientific), Hot Start Taq PCR Master Mix 2X (VWR), or Conquest PCR Master Mix (Lamda Biotech, St. Louis, MO). PCR products were purified with ExoSAP-IT (USB Corporation, Cleveland, OH) or the EZNA Cycle Pure Kit (Omega Bio-Tek). Sanger sequencing was performed by Eurofins Genomics (Louisville, KY), GeneWiz (South Plainfield, NJ), or Retrogen, Inc. (San Diego, CA). Consensus sequences were assembled using Geneious (https://www.geneious.com).

**Table 2.** PCR primers and temperature profiles. Mitochondrial genes: COI = cytochrome c oxidase subunit I; COIII = cytochrome c oxidase subunit III; 16S = ribosomal RNA 16S subunit. Nuclear genes: 18S = ribosomal RNA 18S subunit.

Amplified gene fragment	Primer pair	References for primer sequences	Temperature profile	Taxa
COI	LCO1490/ HCO2198	Folmer et al. 1994	94 °C/180s – (94 °C/30s – 47 °C/45s – 72 °C/60s) * 5 cycles – (94 °C/30s – 52 °C/45s – 72 °C/60s) * 30 cycles – 72 °C/300s	Annelida, Arthropoda, Mollusca, Nemertea
COI	dgLCO/ dgHCO	Meyer 2003	95 °C/120s – (95 °C/40s – 45 °C/40s – 72 °C/60s) * 35 cycles – 72 °C/420s or 95 °C/300s – (95 °C/30s – 48 °C/30s – 72 °C/45s) * 35 cycles – 72 °C/300s	Annelida, Mollusca, Holothuroidea, Caridea
COI	PolyLCO/ PolyHCO	Carr et al. 2011	﻿95 °C/180 s – (95 °C/40 s – 42 °C/40 s – 72 °C/50 s) * 40 cycles – 72 °C/300 s	Annelida
COI	HCO2198/LCO_Apl	Folmer et al. 1994; Bergmeier et al. 2019	95 °C/60s – (95 °C/20s – 52 °C/15s – 72 °C/30s) * 40 cycles – 72 °C/420s	Aplacophora
COI	HCO2198/ CrustF2	Costa et al. 2007	95 °C/60s – (95 °C/30s – 42 °C/90s – 72 °C/60s) *35 cycles – 72 °C/300s	Arthropoda
COI	COIceF/ COIceR	Hoareau and Boissin 2010	95 °C/180s – (94 °C/45s – 48 °C/70s – 72 °C/80s) * 40 cycles – 72 °C/600s	Echinodermata
COI	ECOLa/ HCO2198	Folmer et al. 1994; Knott and Wray 2000 combined as in Deagle et al. 2003	94 °C/240s – (94 °C/30s – 50 °C/30s – 72 °C/45s) * 35 cycles – 72 °C/300s	Asteroidea
COI	Fsco1/ Co13r	Helgen and Rouse 2006	94 °C/180s – (94 °C/45s – 48 °C/45s – 72 °C/60s) * 35 cycles – 72 °C/480s	Crinoidea
COI	COIef/ COIer	Arndt et al. 1996	95 °C/120s – (95 °C/30s – 48 °C/30s – 72 °C/45s) * 35 cycles – 72 °C/600s	Holothuroidea, Echinoidea
COI	VesLCO/ VesHCO	Peek et al. 1997	94 °C/240s – (94 °C/40s – 40 °C/40s – 72 °C/60 s) * 40 cycles – 72 °C/600 s	Vesicomyidae
COI	﻿jgLCO1490/ ﻿jgHCO2198	Geller et al. 2013	95 °C/300s – (95 °C/30s – 48 °C/30s – 72 °C/45s) * 35 cycles – 72 °C/300s	*Hyalogyrina*
COIII	﻿COIIIF/﻿ COIIIR	Geller and Walton 2001	95 °C/120s – (95 °C/30s – 45 °C/30s – 72 °C/60s) * 30 cycles – 72 °C/300s	Actiniaria
16S	16SarL/ 16SbrH	Palumbi 1996; Palumbi et al. 2002	95 °C/180s – (95 °C/40s – 50 °C/40s – 68 °C/50 s) * 35 cycles – 68 °C/300 s or 95 °C/180s – (95 °C/40s – 50 °C/40s – 72 °C/50 s) * 40 cycles – 72 °C/300 s	Annelida, Nemertea, Holothuroidea, Polyplacophora, *Paracrangonareolata*, *Grimotheamonodon*
16S	ANEM16SA/ ANEM16SB	Geller and Walton 2001	95 °C/120s – (95 °C/30s – 60 °C/30s – 72 °C/60s) * 30 cycles – 72 °C/300s	Anthozoa
16S	16S_arL_solenos (CGACTGTTTAACAAAAACATTGCTC)/ 16S_brH_solenos (CCGATTTGAACTCAGATCATGTAG)	this work (K. Kocot)	95 °C/60s – (95 °C/20s – 52 °C/15s – 72 °C/30s) *40 cycles – 72 °C/420s	Aplacophora
16S	AnnF/ 16Sb	Edgecombe et al. 2002; Sjölin et al. 2005 combined as in Stiller et al. 2013	94 °C/120s – (94 °C/40s – 60 °C/40s – 70 °C/45s) * 35 cycles – 72 °C/420s	Sabellidae, Macellicephalinae
18S	Three overlapping fragments: 1F/5R, 3F/bi, a2.0/9R	Giribet et al. 1996, 1999 combined as in Stiller et al. 2013	1F/5R: 95 °C/180s – (95 °C/60s – 49 °C/30s – 72 °C/90s) * 40 cycles – 72 °C/480s; 3F/bi: 95 °C/180s – (95 °C/30s – 52 °C/30s – 72 °C/90s) *40 cycles – 72 °C/480s; a2.0/9R: 95 °C/180s – (95 °C/30s – 49 °C/30s – 72 °C/90s) *40 cycles – 72 °C/480s	Sabellidae
18S	Three overlapping fragments: TimA/1100R2, 3F/bi, a2.0/9R	Giribet et al. 1996, 1999; Norén and Jondelius 1999 combined as in Stiller et al. 2013	TimA/1100R2: 94 °C/180s – (94 °C/30s – 53 °C/45s – 72 °C/120s) * 40 cycles – 72 °C/300s; 3F/bi, a2.0/9R: see previous	Sabellidae
18S	Sol18F/ Sol18R	Neulinger et al. 2006	94 °C/300s – (91 °C/40s – 50 °C/40s – 72 °C/90s) * 40 cycles – 72 °C/300s	Solemyidae

### ﻿﻿Haplotype networks

Sequences were aligned using the MAFFT online service v. 7.471, option L-INS-I (Katoh et al. 2018). Haplotype networks were created with PopART v. 1.7 (Leigh and Bryant 2015) using the TCS algorithm (Clement et al. 2002).

### ﻿﻿Scanning electron microscopy (SEM)

Selected aplacophoran specimens were dried and mounted on stubs without critical point drying or sputter coating. Specimens were imaged on a Phenom Pro SEM.

### ﻿﻿Permits

Specimen collection and field operations were performed under the following permits issued by CONAGEBIO (Comisión Nacional para la Gestión de la Biodiversidad), INCOPESCA (Instituto Costarricense de Pesca y Acuicultura), and SINAC (Sistema Nacional de Áreas de Conservación) under MINAE (Ministerio de Ambiente y Energía), Government of Costa Rica: INCOPESCA-CPI-003-12-2018, R-070-2018-OT-CONAGEBIO, SINAC-CUSBSE-PI-R-032-2018, SINAC-SE-CUS-PI-R-035-2017. In accordance with the Nagoya Protocol on Access and Benefit Sharing, DNA sequencing for this project was authorized by the Contract for the Grant of Prior Informed Consent between MINAE-SINAC-ACMC and Jorge Cortés-Nuñez for the Basic Research Project: “FK190106-Cuantificación de los vínculos biológicos, químicos y físicos entre las comunidades quimiosintéticas con el mar profundo circundante.”

### ﻿﻿Taxonomic listing

For each taxonomic entry, we summarize the known localities and depths of occurrences at the CRM seeps, incorporating both published references and additional material examined in this work. Representative voucher specimens and their associated DNA sequences are listed by dive/deployment number (details in Table 1). Catalog numbers indicate a morphological voucher linked to a tissue sample suitable for genetic analysis, unless otherwise noted. For some groups such as octocorals, morphological vouchers and genetic tissue samples were deposited with separate institutions according to local expertise.

We also summarize the known localities and depths of occurrences beyond the CRM seeps. We indicate new biogeographic records, new depth records (defined here as at least 100 m from a previously reported minimum or maximum depth), and new seep records of species previously associated with vents or organic falls. Exact collection depths are provided where possible; approximations are indicated by ~ .

References indicate previously published records from the CRM seeps. Original species descriptions are indicated by **. References generally include voucher specimen listings with representative images and DNA sequences; where possible, we provide any missing components. Specimen catalog numbers pertain to SIO-BIC unless otherwise indicated. GenBank numbers refer to COI sequences unless otherwise indicated. New sequences are shown in bold.

For higher-level taxonomy, we follow the World Register of Marine Species (WoRMS Editorial Board 2024) or taxon-specific references. We notate higher classification according to the recommended best practice for the DarwinCore term higherClassification (Darwin Core Maintenance Group 2021), with full taxonomic authorities and Linnaean ranks available in the applicable references. Our ordering of major animal groups reflects the phylogeny in Dunn et al. (2014). Entries are listed alphabetically within each category.

### ﻿﻿Annelida

We list entries following the taxonomic arrangement in Rouse et al. (2022).

#### ﻿Annelida | Polychaeta | Errantia | Protodriliformia

**Protodrilidae stet.**

Fig. 3A

**Figure 3.**
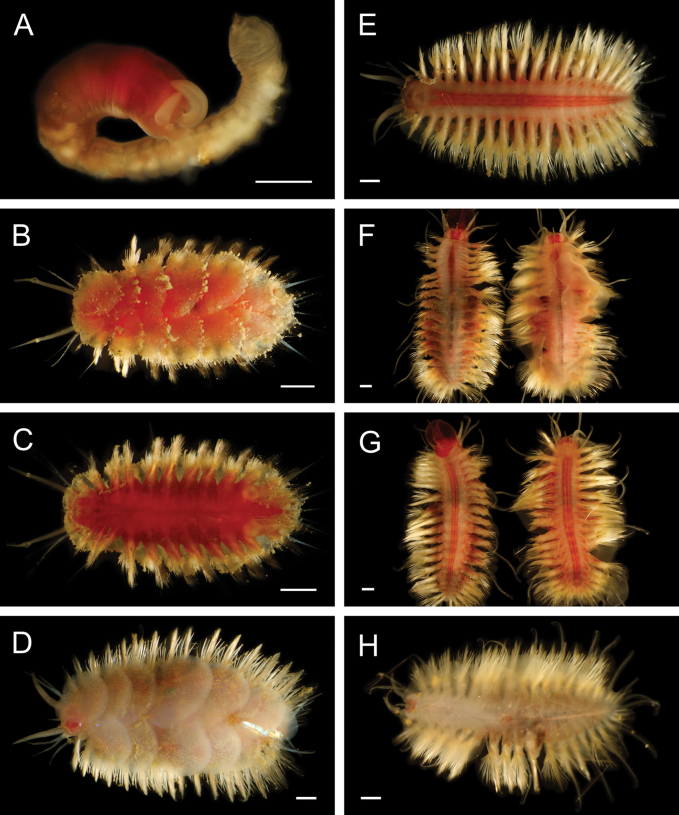
Annelida: Protodrilidae and Polynoidae, representative live images **A**Protodrilidae stet. (A8456) **B***Bathykurila* sp. A sec. Glover et al. 2005 (A10050, dorsal view) **C***Bathykurila* sp. A sec. Glover et al. 2005 (A10050, ventral view) **D***Branchinotogluma* sp. SIO_BIC_A8265 (A8265, dorsal view) **E***Branchinotogluma* sp. SIO_BIC_A8265 (A8265, ventral view) **F***Branchinotogluma* sp. SIO_BIC_A8460 (A8460 and A16362, dorsal view) **G***Branchinotogluma* sp. SIO_BIC_A8460 (A8460 and A16362, ventral view) **H***Branchinotogluma* sp. SIO_BIC_A8460 (A8461, dorsal view). Scale bars: 1 mm.

**Material examined.** AD4906: A8261; AD4923: A8456 (**PQ449314**).

**Localities.** Mound 12 (1002 m), Parrita Seep (~ 1040–1101 m).

#### ﻿Annelida | Polychaeta | Errantia | Aciculata | Phyllodocida | Aphroditiformia | Polynoidae

***Bathykurila* sp. A sec. Glover et al. 2005**

Fig. 3B, C

**Material examined.** AD4906: A8264; S0213: A10050 (**PQ449253**); S0217: A10086.

**Localities.** Jacó Summit (~ 730–820 m), Mound 12 (~ 997–1002 m), Mound 11 (1004–1040 m), The Thumb (1072 m).

**Remarks.**COI sequences of this morphospecies were > 99.42% identical to those of *Bathykurila* haplotype group A (GenBank DQ074778.1, DQ074779.1, DQ074780.1), which morphologically resembles *B.guaymasensis* Pettibone, 1989 as discussed in Glover et al. (2005).

***Branchinotogluma* sp. SIO_BIC_A8265**

Fig. 3D, E

**Material examined.** AD4907: A8265 (OR682087).

**Localities.** Mound 12 (~ 990–999 m).

**Remarks.** An undescribed species. Specimen A8265 was associated with experimentally deployed wood.

***Branchinotogluma* sp. SIO_BIC_A8460**

Fig. 3F–H

**Material examined.** AD4913: A13252 (OR682108); AD4924: A8460 (OR682111), A8461, A16362; S0218: A10190 (OR682109).

**Localities.** Parrita Scar (1364 m), Parrita Seep (~ 1400–1410 m), Jacó Scar (1847 m).

**Remarks.** An undescribed species.

***Branchinotogluma* sp. SIO_BIC_A9682**

Fig. 4A–C

**Figure 4.**
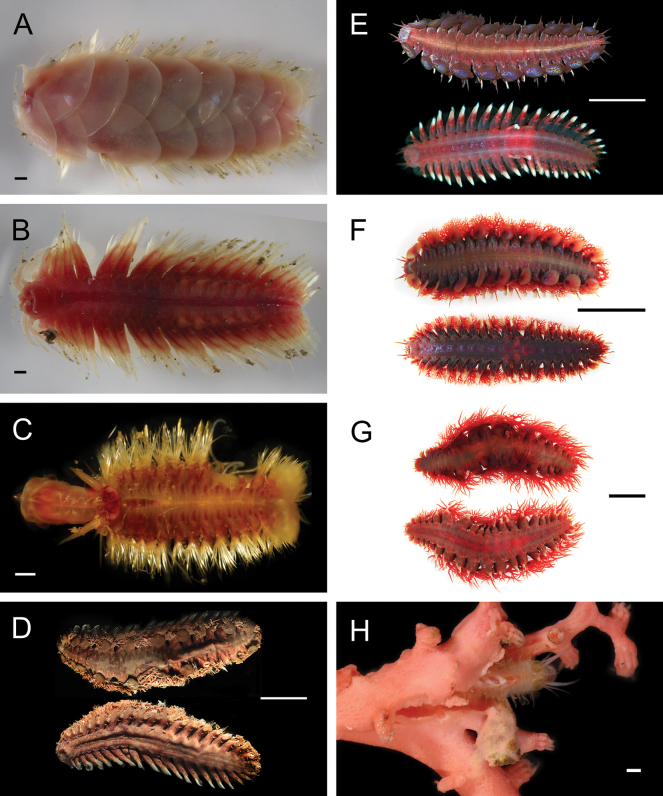
Annelida: Polynoidae, representative images. Live specimens are depicted unless otherwise specified **A***Branchinotogluma* sp. SIO_BIC_A9682 (A9682, dorsal view) **B***Branchinotogluma* sp. SIO_BIC_A9682 (A9682, ventral view) **C***Branchinotogluma* sp. SIO_BIC_A9682 (A9763, dorsal view without scales) **D***Branchipolynoeeliseae* (A6660, preserved specimen, dorsal and ventral views) **E***Branchipolynoehalliseyae* (A1322, dorsal and ventral views) **F***Branchipolynoekajsae* (A6611, dorsal and ventral views) **G***Branchipolynoemeridae* (A6616, dorsal and ventral views) **H**Gorgoniapolynoecf.caeciliae (A8485, with host coralliid Co2947). Scale bars: 1 mm (**A–C, H**); 1 cm (**D–G**).

**Material examined.** AD4505: A1363 (OR682006); AD4924: A8459 (OR682020); AD4972: A9682 (OR682056); AD4978: A9763 (OR682070); S0230: A10185 (OR682045), A10187 (OR682037).

**Localities.** Mound 12 (~ 1000 m), Mound 11 (1025 m), Parrita Seep (~ 1400 m), Jacó Scar (~ 1800 m), Mound Jaguar (1908–1909 m).

**Remarks.** An undescribed species.

***Branchipolynoeeliseae* Lindgren, Hatch, Hourdez, Seid & Rouse, 2019**

Fig. 4D

**Reference.**Lindgren et al. 2019**.

**Localities.** Mound 12 (997 m; type locality), Jacó Scar (~ 1752–1800 m).

**Distribution.** Known only from the CRM seeps.

**Remarks.** Symbiont of the mussels *Bathymodiolusbillschneideri* and *Ba.nancyschneiderae* (Lindgren et al. 2019), typically with one adult worm and sometimes several very small juvenile worms per mussel. Possibly also found in *Ba.earlougheri*, whose locality and depth ranges overlap with the two known host mussel species (McCowin et al. 2020). The absence of *B.eliseae* occurrences in *Ba.earlougheri* is likely an artefact of limited sample size (Lindgren et al. 2019).

***Branchipolynoehalliseyae* Lindgren, Hatch, Hourdez, Seid & Rouse, 2019**

Fig. 4E

**Reference.**Lindgren et al. 2019**.

**Localities.** Mound 12 (~ 1000 m; type locality), Parrita Seep (~ 1400 m), Jacó Scar (1758–1811 m).

**Distribution.** Known only from the CRM seeps.

**Remarks.** Symbiont of the mussels *Bathymodiolusbillschneideri*, *Ba.nancyschneiderae*, and *Ba.earlougheri* (Lindgren et al. 2019), typically with one adult worm and sometimes several very small juvenile worms per mussel.

***Branchipolynoekajsae* Lindgren, Hatch, Hourdez, Seid & Rouse, 2019**

Fig. 4F

**Reference.**Lindgren et al. 2019**.

**Localities.** Mound 12 (~ 1000 m; type locality), Parrita Seep (~ 1400 m), Jacó Scar (~ 1800 m).

**Distribution.** Known only from the CRM seeps.

**Remarks.** Symbiont of the mussels *Bathymodiolusbillschneideri*, *Ba.nancyschneiderae*, and *Ba.earlougheri* (Lindgren et al. 2019), typically with one adult worm and sometimes several very small juvenile worms per mussel.

***Branchipolynoemeridae* Lindgren, Hatch, Hourdez, Seid & Rouse, 2019**

Fig. 4G

**Reference.**Lindgren et al. 2019**.

**Localities.** Mound 12 (~ 1000 m), Jacó Scar (~ 1800 m; type locality).

**Distribution.** Known only from the CRM seeps.

**Remarks.** Symbiont of the mussels *Bathymodiolusbillschneideri* and *Ba.earlougheri* (Lindgren et al. 2019), typically with one adult worm and sometimes several very small juvenile worms per mussel. Possibly also found in *Ba.nancyschneiderae*, which co-occurs with *Ba.earlougheri* at Mound 12 (McCowin et al. 2020). The absence of *B.eliseae* occurrences in *Ba.nancyschneiderae* is likely an artefact of limited sample size (Lindgren et al. 2019).

**Gorgoniapolynoecf.caeciliae (Fauvel, 1913)**

Figs 4H, 5A

**Figure 5.**
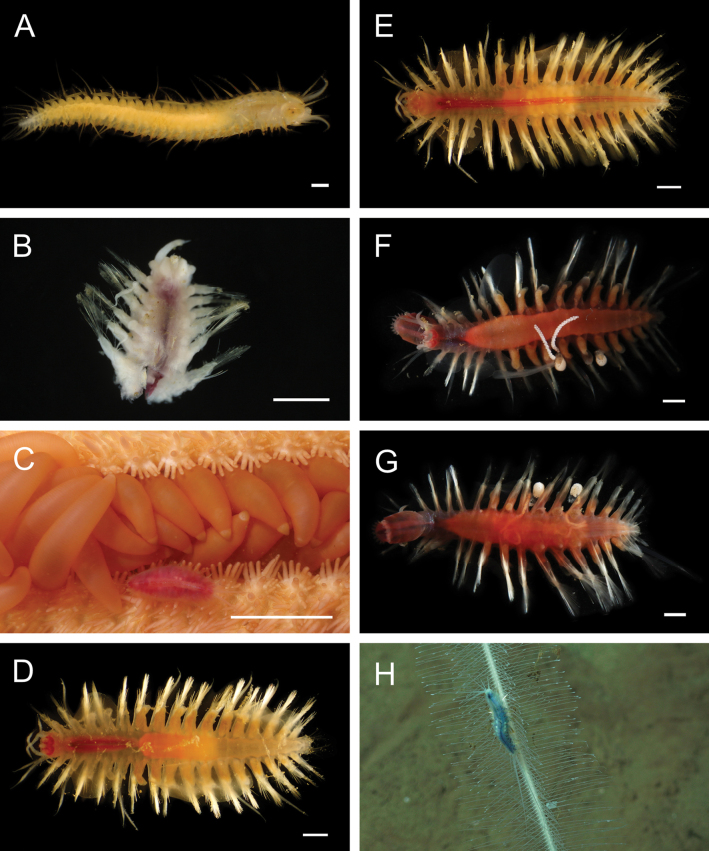
Annelida: Polynoidae, representative images. Live specimens are depicted unless otherwise specified **A**Gorgoniapolynoecf.caeciliae (A8485, removed from host Co2947) **B***Macellicephala* sp. SIO_BIC_A8368 (A8368, preserved specimen) **C***Macellicephala* sp. SIO_BIC_A9775 (A9775) **D***Macellicephala* sp. SIO_BIC_A10055 (A10055, dorsal view) **E***Macellicephala* sp. SIO_BIC_A10055 (A10055, ventral view) **F***Macellicephala* sp. SIO_BIC_A10094 (A10094, dorsal view) **G***Macellicephala* sp. SIO_BIC_A10094 (A10094, ventral view) **H***Macellicephala* sp. SIO_BIC_A10099 (A10099, *in situ* on host cladorhizid sponge P1754). Credit: ROV SuBastian/Schmidt Ocean Institute. Scale bars: 1 mm (**A, B, D–G**); 1 cm (**C**).

**Material examined.** AD4506: A1549; AD4923: A8455 (**PQ449313**), A8485.

**Localities.** Parrita Seep (~ 1030–1094 m).

**Remarks.** Associated with coralliid octocorals: A1549 with coral Co2271, A8455 and A8485 with coral Co2947. The COI sequence of A8455 was 98.20% identical to a reference sequence of Gorgoniapolynoecf.caeciliae molecular operational taxonomic unit (MOTU) 1 from the central Atlantic (ON479554.1), representing one of two lineages in a potential cryptic species complex with inter-lineage COI distances of 2–7% (Maxwell et al. 2022). The CRM specimens warrant further detailed comparison to these Atlantic G.cf.caeciliae lineages as well as to the eastern Pacific species *G.guadalupensis* Pettibone, 1991, which currently has no available reference sequences. *Gorgoniapolynoeguadalupensis* was originally described in association with *Hemicoralliumimperiale* (Bayer, 1955) off Guadelupe Island, western Mexico, 1000–2000 m, and has been recorded from seamounts in the eastern Pacific (Fieberling Guyot, off southern California) and central Pacific (Markus Nekar Chain, west of the Hawaiian Islands) to a minimum known depth of 600 m (Pettibone 1991).

***Macellicephala* sp. SIO_BIC_A8368**

Fig. 5B

**Material examined.** AD4913: A8368 (**PQ449306**).

**Localities.** Jacó Scar (~ 1817–1896 m).

***Macellicephala* sp. SIO_BIC_A9775**

Fig. 5C

**Material examined.** AD4975: A9775 (**PQ449325**).

**Localities.** Mound 12 (1000 m).

**Remarks.** An undescribed species. At least six individuals were associated with the ambulacral groove of an asteroid, *Thrissacanthiaspenicillatus* (E7246).

***Macellicephala* sp. SIO_BIC_A10055**

Fig. 5D, E

**Material examined.** S0213: A10055 (OP648305).

**Localities.** Jacó Summit (~ 730–820 m).

***Macellicephala* sp. SIO_BIC_A10094**

Fig. 5F, G

**Material examined.** S0219: A10094 (**PQ449266**).

**Localities.** Rio Bongo Scar (659 m).

**Remarks.** Afflicted with copepod parasites.

***Macellicephala* sp. SIO_BIC_A10099**

Figs 5H, 6A

**Figure 6.**
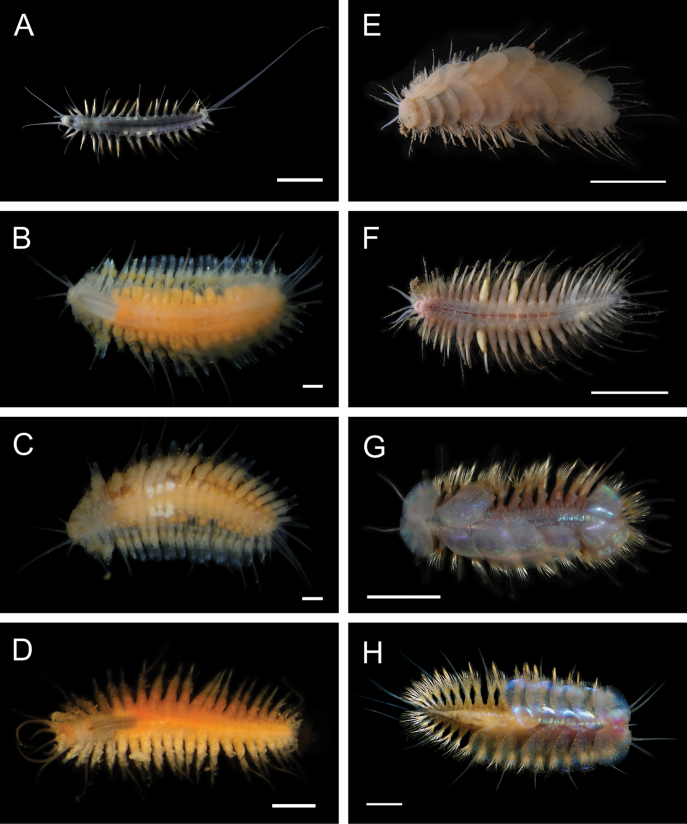
Annelida: Polynoidae, representative live images **A***Macellicephala* sp. SIO_BIC_A10099 (A10099) **B**Macellicephalinae sp. SIO_BIC_A8365 (A8365, dorsal view) **C**Macellicephalinae sp. SIO_BIC_A8365 (A8365, ventral view) **D**Macellicephalinae sp. SIO_BIC_A8458 (A8458) **E**Macellicephalinae sp. SIO_BIC_A10186 (A10186, dorsal view) **F**Macellicephalinae sp. SIO_BIC_A10186 (A10186, ventral view) **G***Peinaleopolynoeelvisi* (A10059) **H***Peinaleopolynoemineoi* (MZUCR 1001-01). Scale bars: 1 cm (**A, G**); 1 mm (**B–F, H**).

**Material examined.** S0220: A10099 (OP648306; 16S: **PQ304650**).

**Localities.** Subduction Plume (3601 m).

**Remarks.** An undescribed species associated with a cladorhizid sponge, P1754.

**Macellicephalinae sp. SIO_BIC_A8365**

Fig. 6B, C

**Material examined.** AD4914: A8365 (**PQ449304**).

**Localities.** Jacó Scar (1839 m).

**Remarks.** Associated with a holothuroid, *Achlyonice* stet. (E7042 or E7043).

**Macellicephalinae sp. SIO_BIC_A8458**

Fig. 6D

**Material examined.** AD4923: A8458 (**PQ449315**).

**Localities.** Parrita Seep (~ 1037–1108 m).

**Macellicephalinae sp. SIO_BIC_A10186**

Fig. 6E, F

**Material examined.** S0230: A10186.

**Localities.** Mound Jaguar (1909 m).

***Peinaleopolynoeelvisi* Hatch, Liew, Hourdez & Rouse, 2020**

Fig. 6G

**Reference.**Hatch et al. 2020**.

**Additional material examined.** S0214: A10059 (**PQ449258**).

**Localities.** Jacó Scar (1845–1887 m).

**Distribution.** Also known from a whale fall in Monterey Submarine Canyon, off California, 1820 m (type locality) and from cow bones experimentally deployed at 2091 m at Seamount 1, which lies on the CRM ca 41 km southwest of Jacó Scar (Hatch et al. 2020).

**Remarks.** A10059 was associated with a naturally occurring wood fall. All known occurrences of *P.elvisi* have been associated with vertebrate bones or wood (naturally occurring and experimentally deployed, for both substrates) (Hatch et al. 2020).

***Peinaleopolynoemineoi* Hatch, Liew, Hourdez & Rouse, 2020**

Fig. 6H

**Reference.**Hatch et al. 2020**.

**Localities.** Mound 12 (992–1011 m; type locality), Mound 11 (1010 m).

**Distribution.** Known only from the CRM seeps.

**Remarks.** All known occurrences of *P.mineoi* have been associated with wood (naturally occurring and experimentally deployed) or experimentally deployed vertebrate bones (Hatch et al. 2020).

**Polynoidae sp. SIO_BIC_A8426**

Fig. 7A–C

**Figure 7.**
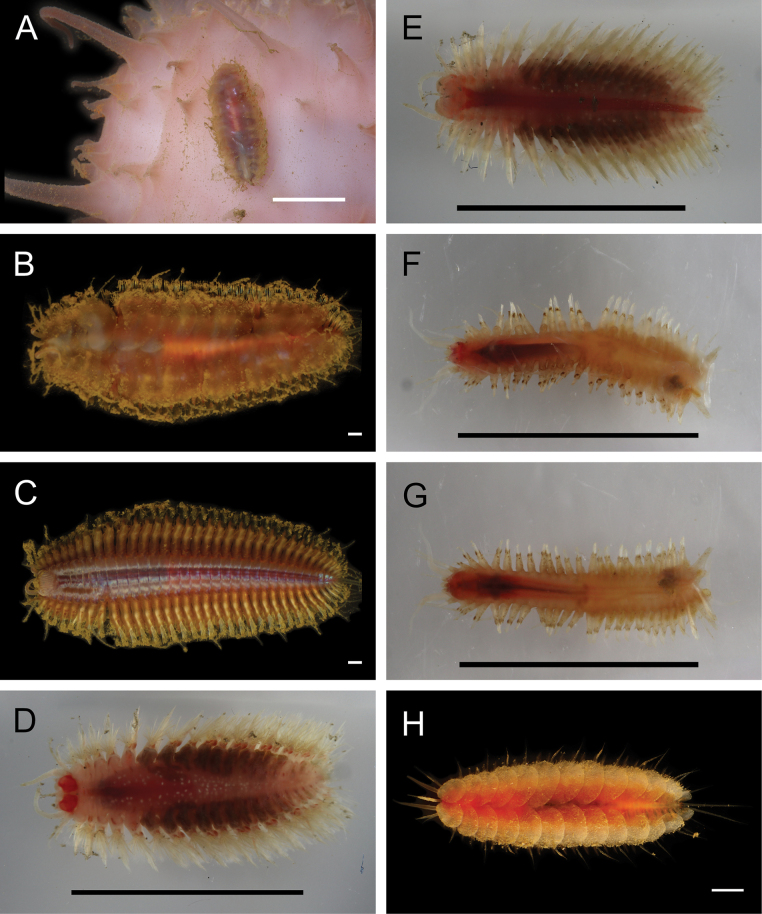
Annelida: Polynoidae, representative live images **A**Polynoidae sp. SIO_BIC_A8426 (A8426, on host holothuroid E7063) **B**Polynoidae sp. SIO_BIC_A8426 (A8426, dorsal view) **C**Polynoidae sp. SIO_BIC_A8426 (A8426, ventral view) **D**Polynoidae sp. SIO_BIC_A9652 (A9652, dorsal view) **E**Polynoidae sp. SIO_BIC_A9652 (A9652, ventral view) **F**Polynoidae sp. SIO_BIC_A9714 (A9714, dorsal view) **G**Polynoidae sp. SIO_BIC_A9714 (A9714, ventral view) **H**Polynoidae sp. SIO_BIC_A10082 (A10082, dorsal view). Scale bars: 1 cm (**A, D–G**); 1 mm (**B, C, H**).

**Material examined.** AD4922: A8426 (**PQ449308**).

**Localities.** Mound 12 (1006 m).

**Remarks.** Associated with a holothuroid, *Bathyplotes* sp. SIO_BIC_E7063.

**Polynoidae sp. SIO_BIC_A9652**

Fig. 7D, E

**Material examined.** AD4972: A9720; AD4973: A9652 (**PQ449319**), A9653.

**Localities.** Jacó Scar (1784 m).

**Polynoidae sp. SIO_BIC_A9714**

Fig. 7F, G

**Material examined.** AD4974: A9714 (**PQ449321**).

**Localities.** Mound 12 (~ 1001–1003 m).

**Polynoidae sp. SIO_BIC_A10082**

Figs 7H, 8A

**Figure 8.**
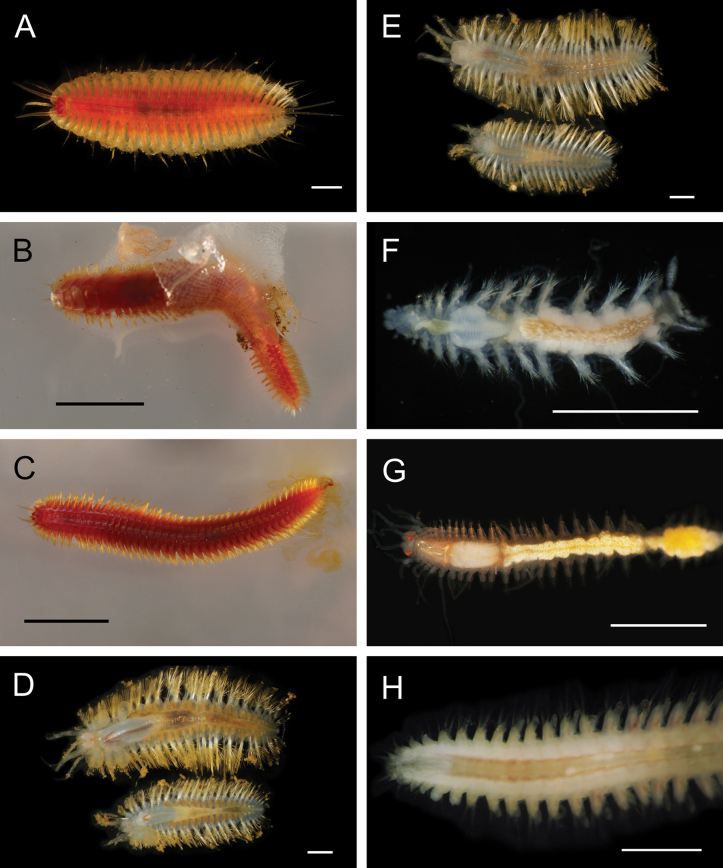
Annelida: Polynoidae, Syllidae, and Nephtyidae, representative live images **A**Polynoidae sp. SIO_BIC_A10082 (A10082, ventral view) **B**Polynoidae sp. SIO_BIC_A10096 (A10096, dorsal view) **C**Polynoidae sp. SIO_BIC_A10096 (A10096, ventral view) **D**Polynoidae sp. SIO_BIC_A10189 (A10189, dorsal view) **E**Polynoidae sp. SIO_BIC_A10189 (A10189, ventral view) **F***Anguillosyllis* sp. SIO_BIC_A12403 (A12403) **G***Synmerosyllis* stet. (A1928) **H***Nephtys* stet. (A1437, dorsal view). Scale bars: 1 mm (**A, D–H**); 1 cm (**B, C**).

**Material examined.** AD4919: A8421; AD4986: A9899; S0216: A10082 (**PQ449262**).

**Localities.** Quepos Slide (~ 308–410 m)

**Remarks.** A10082 was associated with bones from a naturally occurring sailfish carcass.

**Polynoidae sp. SIO_BIC_A10096**

Fig. 8B, C

**Material examined.** S0219: A10095, A10096 (**PQ449267**), A10098, A10109, A10110.

**Localities.** Rio Bongo Scar (~ 480–650 m).

**Remarks.** A10096 and A10098 were commensals in a sponge, *Farreaocca* (P1753).

**Polynoidae sp. SIO_BIC_A10189**

Fig. 8D, E

**Material examined.** AD4913: A8369; S0230: A10189 (**PQ449271**).

**Localities.** Jacó Scar (~ 1817–1896 m), Mound Jaguar (2000 m).

**Remarks.** A10189 was associated with a naturally occurring wood fall.

#### ﻿Annelida | Polychaeta | Errantia | Aciculata | Phyllodocida | Aphroditiformia | Syllidae

***Anguillosyllis* sp. SIO_BIC_A9613**

**Material examined.** AD4975: A9613; AD4978: A9771.

**Localities.** Mound 12 (~ 992–999 m).

**Remarks.** An undescribed species. A9613 was associated with experimentally deployed wood and bone at 992 m.

***Anguillosyllis* sp. SIO_BIC_A12403**

Fig. 8F

**Reference.** (Aguado et al. 2012), in which the published DNA sequences (JF903571, JF903680, JF903756) correspond to specimen A12403 from AT15-59, MC1.

**Localities.** Near Mound 12 (995 m).

**Remarks.** An undescribed species, morphologically similar to *Anguillosyllis* sp. SIO_BIC_A9613. This specimen was collected in a sediment core adjacent to Mound 12, ca 400 m from known sites of active seepage and likely representing the far-transition zone to the surrounding environment.

***Synmerosyllis* stet.**

Fig. 8G

**Reference.**Aguado et al. (2012), in which the published DNA sequences (JF903573, JF903681, JF903759) correspond to specimen A1928 from AD4588.

**Localities.** Mound 12 (997 m).

#### ﻿Annelida | Polychaeta | Errantia | Aciculata | Phyllodocida | Nephtyiformia | Nephtyidae

***Nephtys* stet.**

Figs 8H, 9A

**Figure 9.**
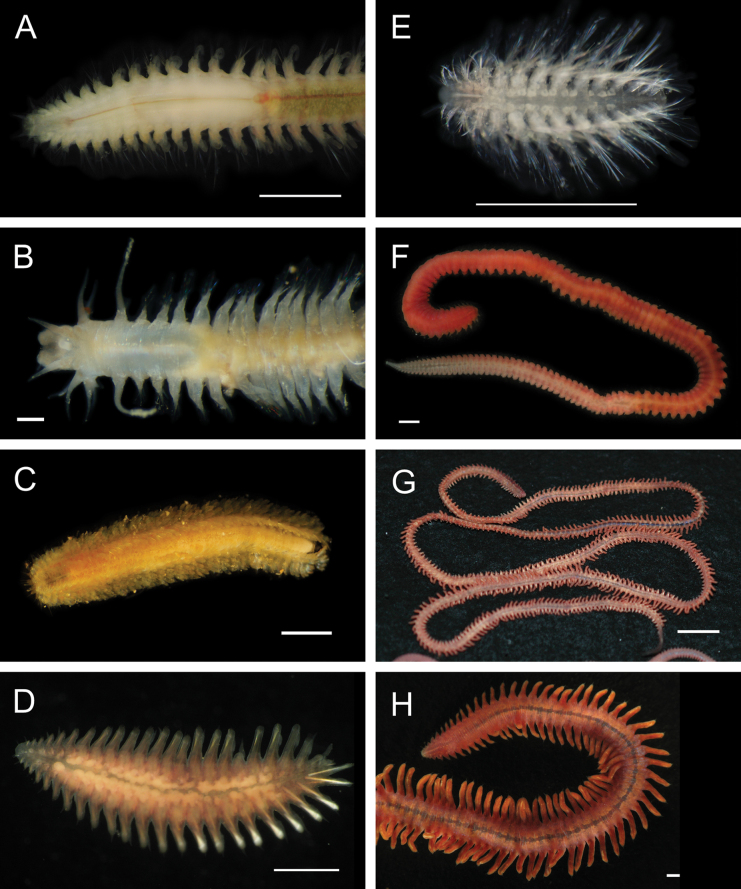
Annelida: Nephtyidae, Pilargidae, and Chrysopetalidae, representative live images **A***Nephtys* stet. (A1437, ventral view) **B***Sigambra* sp. SIO_BIC_A9597 (A9597) **C**Chrysopetalinae sp. SIO_BIC_A8064 **D***Laubierusalvini* (A1323) **E***Micospinaauribohnorum* (A1427) **F***Natsushimasashai* (A1447) **G***Shinkaifontefridae* (A1384) **H***Shinkailongipedata* (A1360). Scale bars: 1 mm (**A–F, H**); 1 cm (**G**).

**Material examined.** AD4510: A1437.

**Localities.** Jacó Summit (742 m).

#### ﻿Annelida | Polychaeta | Errantia | Aciculata | Phyllodocida | Nephtyiformia | Pilargidae

***Sigambra* sp. SIO_BIC_A9597**

Fig. 9B

**Material examined.** AD4503: A1346; AD4972: A9597; AD4987: A9843.

**Localities.** Mound 12 (~ 967–999 m), Jacó Scar (1795 m).

**Remarks.** An undescribed species.

#### ﻿Annelida | Polychaeta | Errantia | Aciculata | Phyllodocida | Hesionoidea | Chrysopetalidae

**Chrysopetalinae sp. SIO_BIC_A8064**

Fig. 9C

**Material examined.** AD4508: A1404, A2410; AD4914: A8063; AD4917: A8065; AD4922: A8064, A10280; AD4974: A9609, A9758; AD4985: A9853; AD4990: A9879.

**Localities.** Mound 12 (~ 965–1002 m), Parrita Seep (1401–1402 m), Jacó Scar (~ 1632–1886 m).

**Remarks.** An undescribed genus and species.

***Laubierusalvini* Aguado & Rouse, 2011**

Fig. 9D

**Reference.**Aguado and Rouse 2011**.

**Additional material examined.** S0214: A10057 (**PQ449257**).

**Localities.** Mound 12 (~ 982–999 m), Mound 11 (~ 1004–1011 m; type locality), Jacó Scar (~ 1752–1860 m).

**Distribution.** Known only from the CRM seeps.

**Remarks.** As noted in the original description, *L.alvini* is a symbiont of the mussel *Bathymodiolusearlougheri*, but not of the two sympatric species subsequently described as *B.billschneideri* and *B.nancyschneiderae*. The worms are found among the gill filaments, with typically 2–25 individuals per host, and the number of individuals linearly correlates with the length of the host (Aguado and Rouse 2011).

***Micospinaauribohnorum* Watson, Carvajal, Sergeeva, Pleijel & Rouse, 2016**

Fig. 9E

**Reference.**Watson et al. 2016**.

**Additional material examined.** S0217: A10088 (**PQ449265**).

**Localities.** Jacó Summit (~ 750 m), Mound 12 (~ 1000 m), Mound 11 (~ 1040 m), The Thumb (1072 m; this study), Jacó Scar (~ 1800 m).

**Distribution.** Also known from a whale fall at 845 m off San Diego, California (type locality) (Watson et al. 2016).

***Natsushimasashai* Aguado & Rouse, 2011**

Fig. 9F

**Reference.**Aguado and Rouse 2011**.

**Localities.** Mound 12 (~ 1000 m; type locality).

**Distribution.** Known only from the CRM seeps.

**Remarks.** A symbiont of the solemyid clam Acharaxcf.johnsoni; the worms are found among the gill lamellae, with no more than four individuals per host, in three possible combinations: one female, one male and one female, or two males and two females (Aguado and Rouse 2011).

***Shinkaifontefridae* Aguado & Rouse, 2011**

Fig. 9G

**Reference.**Aguado and Rouse 2011**.

**Localities.** Parrita Seep (1186 m), Parrita Scar (~ 1660 m), Jacó Scar (~ 1752–1800 m; type locality).

**Distribution.** Known only from the CRM seeps.

**Remarks.** Symbiont of the vesicomyid clams *Phreagenasoyoae* and *Archivesicagigas* (previously cited as *Calyptogenakilmeri* and *Vesicomyagigas*, respectively); the worms are found between the gill lamellae and foot, typically with one male and one female per host (Aguado and Rouse 2011).

***Shinkailongipedata* Miura & Ohta, 1991**

Fig. 9H

**Reference.**Aguado and Rouse 2011.

**Localities.** Mound 11 (~ 1019–1025 m).

**Distribution.** Originally described from hydrothermal vents at Iheya Ridge off southern Japan (Miura and Ohta 1991). The occurrences of *S.longipedata* at the CRM established the first record of this species in the eastern Pacific, based on morphological similarity to specimens from Japan and in the absence of DNA sequences from the type locality (Aguado and Rouse 2011). Genetic data from the type locality will be necessary to confirm the apparent trans-Pacific distribution of this species at both vents and seeps.

**Remarks.** Originally described as a commensal symbiont in the mantle cavity of the vesicomyid clam *Calyptogena* sp. (Miura and Ohta 1991). At the CRM seeps, the host is the vesicomyid clam *Phreagenasoyoae* (previously identified as an “undescribed” vesicomyid); the worms are found between the gill lamellae and foot, typically with one male and one female per host (Aguado and Rouse 2011).

***Vigtorniella* sp. SIO_BIC_A8061**

Fig. 10A

**Figure 10.**
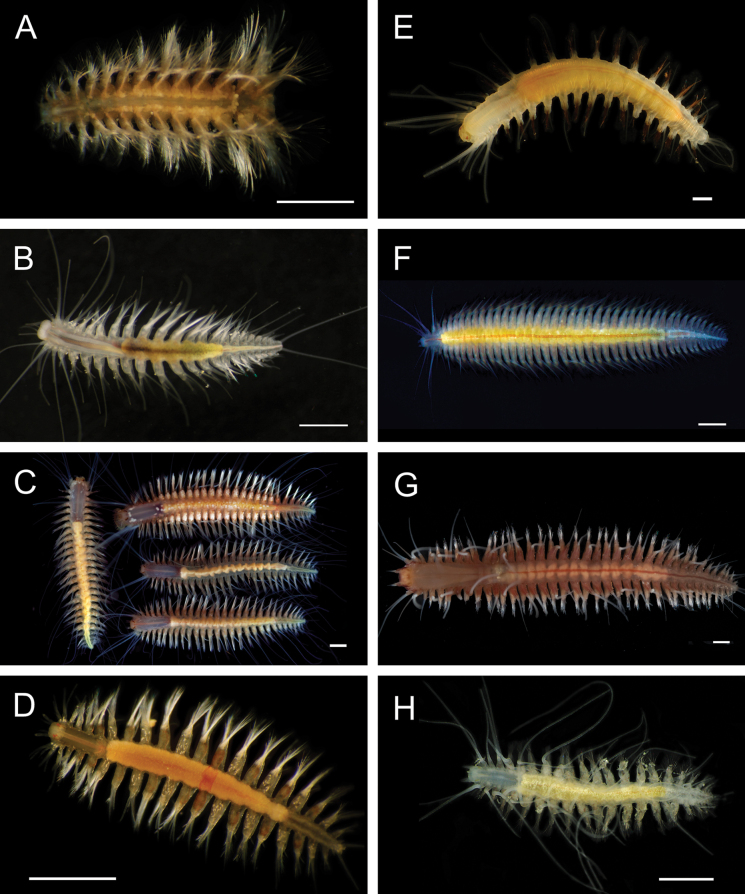
Annelida: Chrysopetalidae and Hesionidae, representative live images **A***Vigtorniella* sp. SIO_BIC_A8061 (A8061) **B**Amphiduropsiscf.axialensis (A8110) **C***Gyptisrobertscrippsi* (A1750) **D***Gyptis* sp. SIO_BIC_A10083 (A10083) **E***Leocrates* gen. inc. (A10184) **F***Neogyptisjeffruoccoi* (A1448) **G***Sirsoedalailamai* (MZUCR 401-01) **H***Sirsoemunki* (A1409). Scale bars: 1 mm.

**Material examined.** AD4914: A8061, A8062.

**Localities.** Jacó Scar (~ 1632–1886 m).

**Remarks.** An undescribed species.

#### ﻿Annelida | Polychaeta | Errantia | Aciculata | Phyllodocida | Hesionoidea | Hesionidae

**Amphiduropsiscf.axialensis (Blake & Hilbig, 1990)**

Fig. 10B

**Reference.**Rouse et al. 2018.

**Localities.** Mound 12 (~ 984–997 m).

**Remarks.** The type locality of *Amphiduropsisaxialensis* is hydrothermal vents at 1545 m on the Axial Seamount of the Juan de Fuca Ridge (Blake and Hilbig 1990). As discussed in Rouse et al. (2018), the specimens above (and other seep specimens from Hydrate Ridge off Oregon, 587–809 m, and the Guaymas Basin, Gulf of California, 1560–1613 m) are morphologically consistent with *A.axialensis* but are conservatively designated as A.cf.axialensis because DNA sequences are not available from the type locality.

***Gyptisrobertscrippsi* Rouse, Carvajal & Pleijel, 2018**

Fig. 10C

**Reference.**Rouse et al. 2018**.

**Localities.** Mound 12 (~ 982–1002 m; type locality), Mound 11 (~ 1019–1025 m), Jacó Scar (1783–1794 m).

**Distribution.** Known only from the CRM seeps.

***Gyptis* sp. SIO_BIC_A10083**

Fig. 10D

**Material examined.** AD4919: A8114; S0216: A10083.

**Localities.** Quepos Slide (~ 379–398 m).

**Remarks.** An undescribed species.

***Leocrates* gen. inc.**

Fig. 10E

**Material examined.** AD4914: A8113; S0230: A10184.

**Localities.** Jacó Scar (1886 m), Mound Jaguar (1909 m).

**Remarks.** Taxonomic placement of these specimens requires further assessment, particularly in light of recent revisions to several hesionid genera (Salazar-Vallejo 2020b).

***Neogyptisjeffruoccoi* Rouse, Carvajal & Pleijel, 2018**

Fig. 10F

**Reference.**Rouse et al. 2018**.

**Localities.** Mound 12 (~ 988–997 m; type locality), Mound 11 (~ 1019–1025 m).

**Distribution.** Also known from seeps in the Guaymas Basin, Gulf of California, 1572–1613 m, and off Del Mar, California, 1020 m (Rouse et al. 2018).

**Remarks.** Found in the mantle cavity of the solemyid clam Acharaxcf.johnsoni, with either one individual or two (one male and one female) per host (Rouse et al. 2018).

***Sirsoedalailamai* Rouse, Carvajal & Pleijel, 2018**

Fig. 10G

**Reference.**Rouse et al. 2018**.

**Localities.** Mound 12 (997 m), Parrita Seep (1402 m), Jacó Scar (1784–1795 m; type locality).

**Distribution.** Also described from a seep in the Guaymas Basin, Gulf of California, 1560–1613 m (Rouse et al. 2018).

**Remarks.** Associated with vestimentiferan and mussel communities at areas of active methane seepage (Rouse et al. 2018).

***Sirsoemunki* Rouse, Carvajal & Pleijel, 2018**

Fig. 10H

**Reference.**Rouse et al. 2018**.

**Additional material examined.** S0217: A10087 (**PQ449264**).

**Localities.** Jacó Slope (1063 m), The Thumb (1072 m; this study), Jacó Scar (~ 1800 m; type locality).

**Distribution.** Known only from the CRM seeps.

***Sirsoe* sp. SIO_BIC_A8288**

Fig. 11A

**Figure 11.**
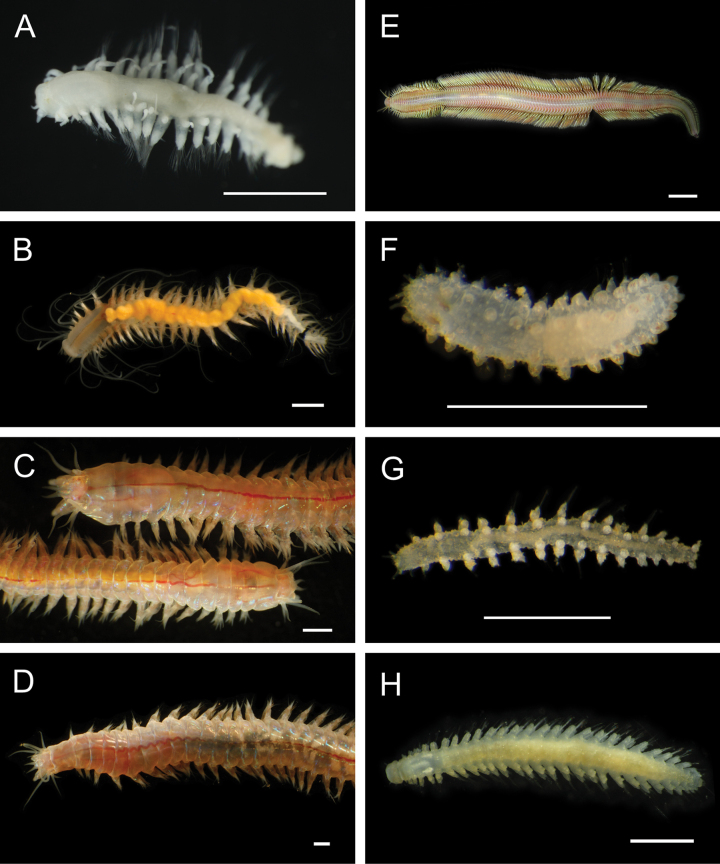
Annelida: Hesionidae, Nereididae, Sphaerodoridae, and Lacydoniidae, representative images. Live specimens are depicted unless otherwise specified **A***Sirsoe* sp. SIO_BIC_A8288 (A8288, preserved specimen) **B***Vrijenhoekia* sp. A sec. Summers et al. 2015 (A9606) **C***Nereis* stet. (A8291) **D**Nereididae sp. SIO_BIC_A9614 (A9614) **E***Pectinereisstrickrotti* (A9889) **F***Sphaerephesia* sp. SIO_BIC_A10069 (A10069) **G***Sphaerodoropsis* sp. SIO_BIC_A10068 (A10068) **H***Lacydonia* sp. SIO_BIC_A1432 (A1432). Scale bars: 1 mm (**A–D, F–H**); 1 cm (**E**).

**Material examined.** AD4909: A8288 (**PQ449293**).

**Localities.** Mound 12 (990 m).

**Remarks.** An undescribed species associated with a microbial mat.

***Vrijenhoekia* sp. A sec. Summers et al. 2015**

Fig. 11B

**Material examined.** AD4508: A1406; AD4907: A8101; AD4974: A9606, A9608, A9612, A9615; AD4988: A9926.

**Localities.** Mound 11 (1010 m), Mound 12 (~ 990–999 m), Parrita Seep (1419 m).

**Remarks.** The undescribed species *Vrijenhoekia* sp. A was previously known only from a single specimen, SIO-BIC A3255, from a seep in the Guaymas Basin, Gulf of California, 1565–1598 m; the specimen was collected in poor condition and diagnostic morphological features could not be assessed (Summers et al. 2015). The CRM specimens represent the first records of this morphospecies beyond the Guaymas Basin and expand the known depth range to ~ 1000–1600 m. The CRM specimens were associated with naturally occurring and experimentally deployed wood (A1406, A9606, A9608, A9926) or experimentally deployed bone (A9615).

#### ﻿Annelida | Polychaeta | Errantia | Aciculata | Phyllodocida | Nereididae

These specimens will be further examined and compared to other eastern Pacific Nereididae in a separate work.

***Nereis* stet.**

Fig. 11C

**Material examined.** AD4906: A8258; AD4910: A8291.

**Localities.** Mound 12 (997–1002 m).

**Remarks.** A8258 was associated with experimentally deployed bone.

**Nereididae sp. SIO_BIC_A9614**

Fig. 11D

**Material examined.** AD4974: A9614.

**Localities.** Mound 12 (992 m)

**Remarks.** An undescribed species. Associated with experimentally deployed wood and pig bones.

***Pectinereisstrickrotti* Villalobos-Guerrero, Huč, Tilic, Hiley & Rouse, 2024**

Fig. 11E

**Reference.**Villalobos-Guerrero et al. 2024**.

**Material examined.** AD4984: A9889.

**Localities.** Mound 12 (996–1010 m).

**Distribution.** Known only from the CRM seeps.

**Remarks.** Known from epitokous males, all collected while swimming just above the seafloor, and from a fragment of an atokous infaunal female recovered from a push core sample (Villalobos-Guerrero et al. 2024).

#### ﻿Annelida | Polychaeta | Errantia | Aciculata | Phyllodocida | Glyceriformia | Sphaerodoridae

***Sphaerephesia* sp. SIO_BIC_A10069**

Fig. 11F

**Material examined.** S0214: A10069 (**PQ449261**; no voucher remaining).

**Localities.** Jacó Scar (1875 m).

**Remarks.** Associated with a naturally occurring wood fall. The closest COI BLASTN result on GenBank was a specimen of Sphaerephesiacf.discolis (Borowski, 1994) from the Brazilian Basin, 5180 m (KR019875.1, 95.39% identity, formerly Sphaerodoropsiscf.discolis) (Capa et al. 2016, 2019). *Sphaerephesiadiscolis* was originally described from the Peru Basin, 4152 m (Capa et al. 2019), so a comparison of the CRM specimen to material from the type locality would be informative.

***Sphaerodoropsis* sp. SIO_BIC_A10068**

Fig. 11G

**Material examined.** S0214: A10068 (**PQ449260**).

**Localities.** Jacó Scar (1875 m).

**Remarks.** Associated with a naturally occurring wood fall.

#### ﻿Annelida | Polychaeta | Errantia | Aciculata | Phyllodocida | Phyllodociformia

These specimens will be further examined and compared to other eastern Pacific *Lacydonia* in a separate work.

***Lacydonia* sp. SIO_BIC_A1432**

Fig. 11H

**Material examined.** AD4504: A1525, A1526; AD4510: A1432.

**Localities.** Jacó Summit (~ 741–744 m), Mound 11 (~ 1004–1011 m).

**Remarks.** An undescribed species.

***Lacydonia* sp. SIO_BIC_A1606**

**Material examined.** AD4510: A1606.

**Localities.** Jacó Summit (~ 741–744 m).

**Remarks.** An undescribed species.

***Lacydonia* sp. SIO_BIC_A9774**

Fig. 12A

**Figure 12.**
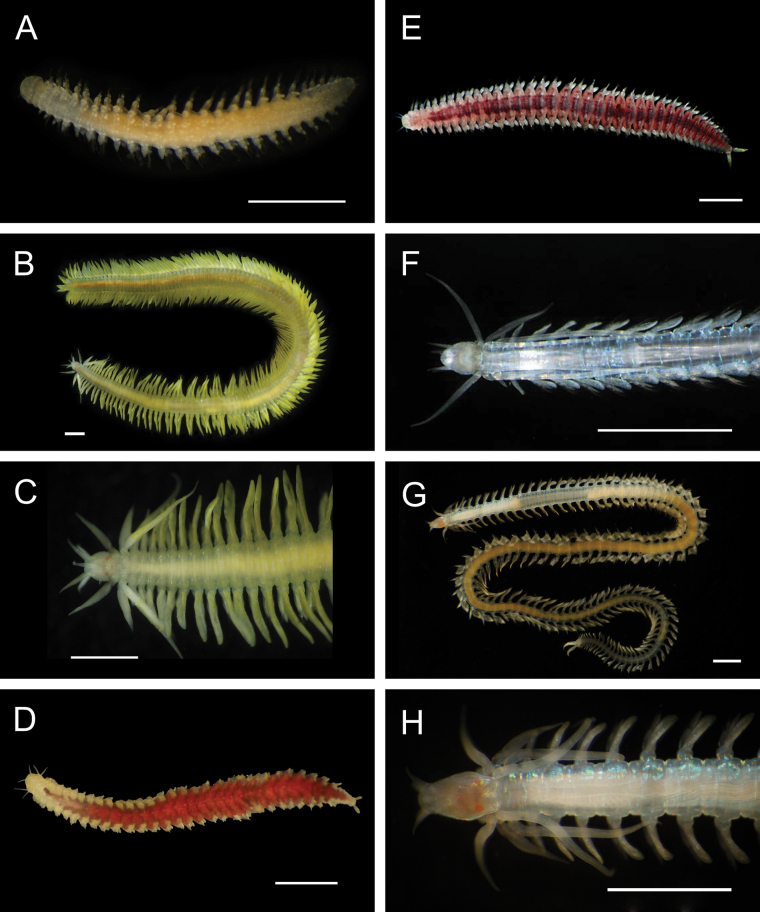
Annelida: Lacydoniidae and Phyllodocidae, representative live images **A***Lacydonia* sp. SIO_BIC_A9774 (A9774) **B***Eulalia* sp. SIO_BIC_A1383 (A1383, wide view) **C***Eulalia* sp. SIO_BIC_A1383 (A1383, anterior detail) **D***Galapagomystidespatricki* (A9934) **E***Galapagomystidesverenae* (A10044) **F***Phyllodoce* sp. SIO_BIC_A1469 (A1469) **G***Phyllodoce* sp. SIO_BIC_A8454 (A8454, wide view) **H***Phyllodoce* sp. SIO_BIC_A8454 (A8454, anterior detail). Scale bars: 1 mm.

**Material examined.** AD4588: A1925; AD4978: A9774; AD4990: A9880; S0217: A10090 (MZ562520).

**Localities.** The Thumb (~ 940–1070 m), Mound 12 (~ 996–999 m), Parrita Seep (1401 m).

**Remarks.** An undescribed species.

***Lacydonia* sp. SIO_BIC_A16347**

**Material examined.** AD4504: A16347.

**Localities.** Mound 11 (~ 1004–1011 m).

**Remarks.** An undescribed species.

#### ﻿Annelida | Polychaeta | Errantia | Aciculata | Phyllodocida | Phyllodociformia | Phyllodocidae

***Eulalia* sp. SIO_BIC_A1383**

Fig. 12B, C

**Material examined.** AD4506: A1383 (**PQ449278**).

**Localities.** Parrita Seep (~ 1030–1179 m).

***Galapagomystidespatricki* Pearson & Rouse, 2022**

Fig. 12D

**Reference.**Pearson and Rouse 2022**.

**Localities.** Parrita Seep (~ 1401–1419 m; type locality), Jacó Scar (1762–1785 m).

**Distribution.** Known only from the CRM seeps and vicinity. One paratype was collected from a multicore sample ca 21 km northwest of Parrita Seep, at 805 m depth (Cruise AT15-44, Multicore 17: 9.1713, -84.7459) (Pearson and Rouse 2022).

**Remarks.** Some specimens were associated with empty vestimentiferan tubes (Pearson and Rouse 2022).

***Galapagomystidesverenae* (Blake & Hilbig, 1990)**

Fig. 12E

**Reference.**Pearson and Rouse 2022.

**Localities.** Mound 12 (~ 984–997 m), Parrita Seep (~ 1400–1410 m), Jacó Scar (~ 1632–1908 m).

**Distribution.** Originally described from vents on the Juan de Fuca and Explorer Ridges, 1545–2195 m (Blake and Hilbig 1990). Also known from seeps in the Guaymas Basin, Gulf of California, to the CRM, 1572 to ~ 1800 m (Pearson and Rouse 2022).

**Remarks.** At the CRM seeps, *G.verenae* is associated with the tubes of juvenile *Escarpiaspicata* and may feed on the blood of vestimentiferans (Pearson and Rouse 2022).

***Phyllodoce* sp. SIO_BIC_A1469**

Fig. 12F

**Material examined.** AT15-44 MC1-2: A1469 (**PQ449283**).

**Localities.** Near Mound 12 (1019 m).

**Remarks.** This specimen was collected in a sediment core adjacent to Mound 12, ca 500 m from known sites of active seepage and likely representing the far-transition zone to the surrounding environment.

***Phyllodoce* sp. SIO_BIC_A8454**

Fig. 12G, H

**Material examined.** AD4505: A1366; AD4508: A1399; AD4922: A8454 (**PQ449312**); AD4975: A9795; AD4978: A9798 (**PQ449326**).

**Localities.** Mound 12 (~ 964–1009 m), Mound 11 (~ 1019–1025 m), Parrita Seep (~ 1401–1419 m).

**Phyllodocidae sp. SIO_BIC_A10054**

Fig. 13A

**Figure 13.**
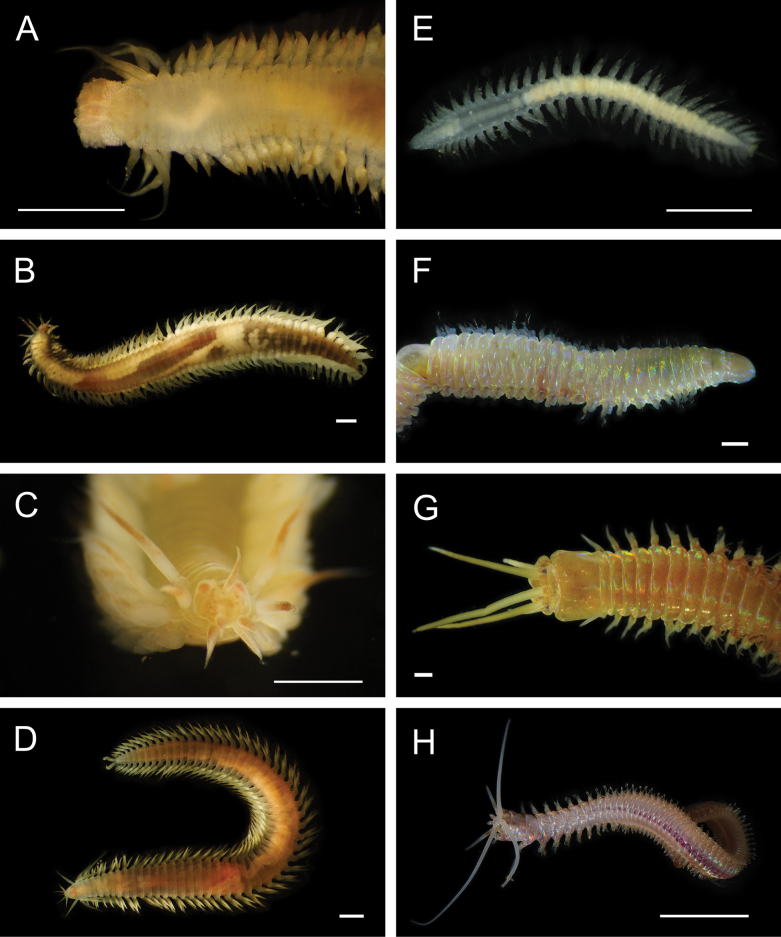
Annelida: Phyllodocidae, *Paralacydonia*, Eunicidae, and Onuphidae, representative live images **A**Phyllodocidae sp. SIO_BIC_A10054 (A10054) **B***Sige* sp. SIO_BIC_A8263 (A8263) **C***Sige* sp. SIO_BIC_A8263 (A8356) **D***Sige* sp. SIO_BIC_A8430 (A8430) **E***Paralacydonia* stet. (A1412, wide view) **F**Lumbrineridae stet. (A1370) **G**Eunicidae stet. (A1431) **H***Hyalinoecia* stet. (A10060). Scale bars: 1 mm (**A–G**); 1 cm (**H**)

**Material examined.** S0212: A10054 (**PQ449256**).

**Localities.** Jacó Scar (~ 1780–1860 m).

***Sige* sp. SIO_BIC_A8263**

Fig. 13B, C

**Material examined.** AD4906: A8263 (**PQ449290**); AD4912: A8356 (**PQ449301**).

**Localities.** Mound 12 (1002 m), Jacó Scar (~ 1795–1859 m).

***Sige* sp. SIO_BIC_A8430**

Fig. 13D

**Material examined.** AD4907: A8284 (**PQ449292**); AD4922: A8430 (**PQ449311**).

**Localities.** Mound 12 (~ 990–1002 m).

**Remarks.** A8430 was associated with a naturally occurring wood fall.

#### ﻿Annelida | Polychaeta | Errantia | Aciculata | Phyllodocida | Phyllodociformia

***Paralacydonia* stet.**

Fig. 13E

**Material examined.** AD4509: A1412.

**Localities.** Jacó Scar (1855 m).

#### ﻿Annelida | Polychaeta | Errantia | Aciculata | Eunicida | Oenonoidea

**Lumbrineridae stet.**

Fig. 13F

**Material examined.** AD4505: A1370 (**PQ448999**).

**Localities.** Mound 11 (~ 1019–1025 m).

#### ﻿Annelida | Polychaeta | Errantia | Aciculata | Eunicida | Eunicoidea | Eunicidae

**Eunicidae stet.**

Fig. 13G

**Material examined.** AD4510: A1431; AD4914: A8386; AD4985: A9895 (**PQ449332**).

**Localities.** Mound 12 (991 m), Jacó Summit (741 m), Jacó Scar (1798 m).

**Remarks.** The closest COI BLASTN result on GenBank was *Leodiceantarctica* (Baird, 1869) (GQ497532.1, 92.64% identity, formerly *Euniceantarctica*), known from Antarctic and sub-Antarctic waters (Baird 1869). The identification of species of *Eunice* and *Leodice*, among other genera, is complicated by the need for taxonomic revision (Zanol et al. 2021).

#### ﻿Annelida | Polychaeta | Errantia | Aciculata | Eunicida | Eunicoidea | Onuphidae

***Hyalinoecia* stet.**

Fig. 13H

**Material examined.** AD4510: A1440; S0214: A10060 (**PQ449259**).

**Localities.** Jacó Summit (~ 741–744 m), Jacó Scar (1875 m).

#### ﻿Annelida | Polychaeta | Errantia | Aciculata | Eunicida | Dorvilleidae

**Ophryotrochacf.batillus Wiklund et al., 2012**

Fig. 14A

**Figure 14.**
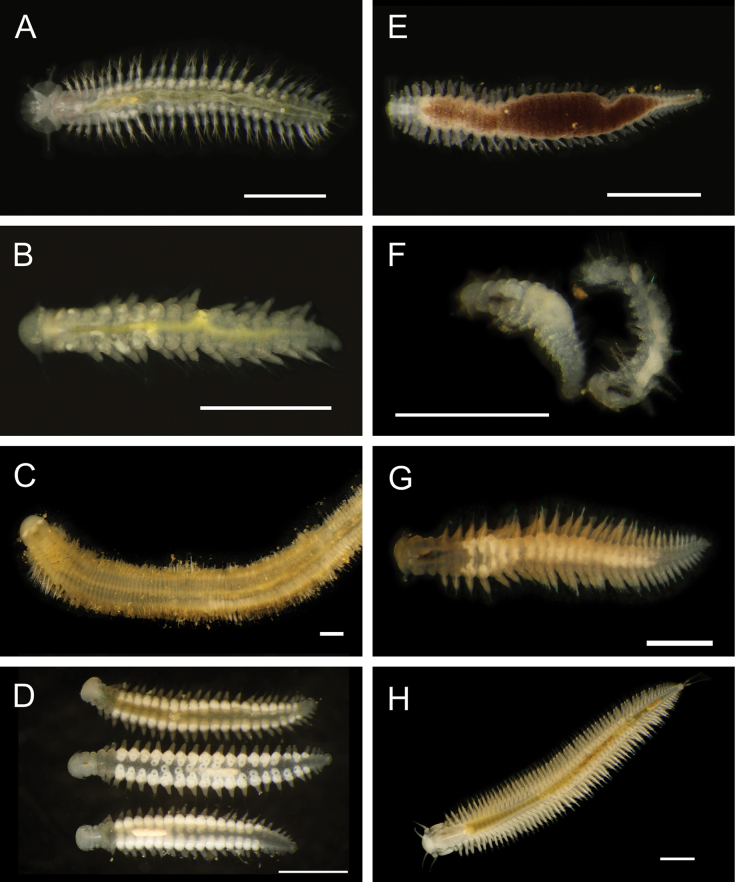
Annelida: Dorvilleidae: *Ophryotrocha*, representative live images **A**Ophryotrochacf.batillus (A9610) **B**Ophryotrochacf.flabella (A1410) **C**Ophryotrochacf.platykephale (A9878) **D***Ophryotrocha* sp. SIO_BIC_A8367 (A8367) **E***Ophryotrocha* sp. SIO_BIC_A9611 (A9611) **F***Ophryotrocha* sp. SIO_BIC_A9723 (A9723) **G***Ophryotrocha* sp. SIO_BIC_A9800 (A9800) **H***Ophryotrocha* sp. SIO_BIC_A10052 (A10052). Scale bars: 1 mm.

**Material examined.** AD4974: A9610 (MT444001).

**Localities.** Mound 12 (992 m).

**Remarks.** Associated with experimentally deployed wood. To be discussed in a forthcoming study. *Ophryotrochabatillus* was originally described from whale falls and wood falls off southern California, 960–1960 m (Wiklund et al. 2012).

**Ophryotrochacf.flabella Wiklund et al., 2012**

Fig. 14B

**Material examined.** AD4509: A1410 (MT435616); AD4988: A9928 (MT435618; no specimen remaining), A9929 (MT435617).

**Localities.** Mound 11 (1010 m), Jacó Slope (1064 m).

**Remarks.** To be discussed in a forthcoming study. *Ophryotrochaflabella* was originally described from whale falls off southern California, 960–1960 m (Wiklund et al. 2012). Specimens A9928 and A9929 were associated with a naturally occurring wood fall.

**Ophryotrochacf.platykephale Blake, 1985**

Fig. 14C

**Material examined.** AD4990: A9878 (MT435620).

**Localities.** Parrita Seep (~ 1400–1435 m).

**Remarks.** To be discussed in a forthcoming study. *Ophryotrochaplatykephale* was originally described from sedimented vents at 2000–2030 m in the Guaymas Basin, Gulf of California (Blake 1985; Solís-Weiss and Hilbig 1992).

***Ophryotrocha* sp. SIO_BIC_A8367**

Fig. 14D

**Material examined.** AD4914: A8367 (**PQ449305**).

**Localities.** Jacó Scar (~ 1632–1886 m).

**Remarks.** Likely an undescribed species.

***Ophryotrocha* sp. SIO_BIC_A9611**

Fig. 14E

**Material examined.** AD4974: A9611 (**PQ449317**).

**Localities.** Mound 12 (992 m).

**Remarks.** An undescribed species, associated with experimentally deployed bone and wood.

***Ophryotrocha* sp. SIO_BIC_A9723**

Fig. 14F

**Material examined.** AD4976: A9723 (**PQ449322**).

**Localities.** Jacó Scar (1887 m).

**Remarks.** Likely an undescribed species. Associated with experimentally deployed wood.

***Ophryotrocha* sp. SIO_BIC_A9800**

Fig. 14G

**Material examined.** AD4912: A8354; AD4913: A8360; AD4978: A9800 (MT435612).

**Localities.** Mound 12 (999 m), Jacó Scar (~ 1795–1859 m).

**Remarks.** An undescribed species.

***Ophryotrocha* sp. SIO_BIC_A10052**

Fig. 14H

**Material examined.** S0213: A10052 (MT435579).

**Localities.** Jacó Summit (~ 730–820 m).

**Remarks.** An undescribed species.

***Ophryotrocha* sp. SIO_BIC_A10084**

Fig. 15A

**Figure 15.**
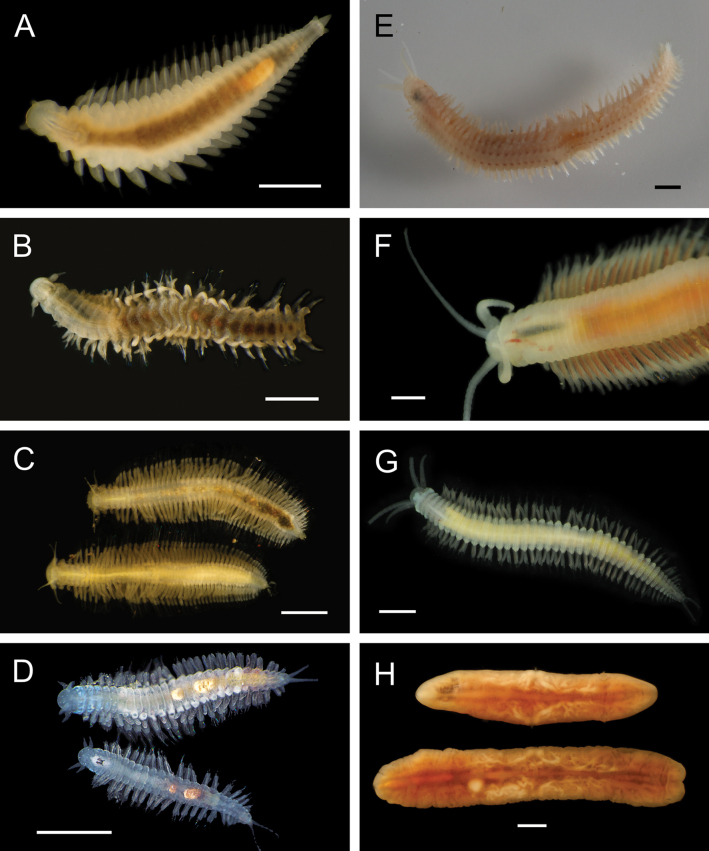
Annelida: Dorvilleidae and Myzostomida, representative live images **A***Ophryotrocha* sp. SIO_BIC_A10084 (A10084) **B***Ophryotrocha* sp. SIO_BIC_A10106 (A10106) **C***Ophryotrocha* sp. SIO_BIC_ A10114 (A10114) **D***Parougiaceruleibohnorum* (A1446) **E**Parougiacf.billiemiroae (A9678) **F**Parougiacf.sulleyi (A1333) **G***Parougiatheloniousblueski* (A1337) **H***Eenymeenymyzostoma* sp. SIO_BIC_A8428 (A8428, dorsal view). Scale bars: 1 mm.

**Material examined.** S0217: A10084 (MT435566), MZUCR 1512-01; S0219: A10113 (MT435562).

**Localities.** Rio Bongo Scar (610 m), The Thumb (1072 m).

**Remarks.** An undescribed species.

***Ophryotrocha* sp. SIO_BIC_A10106**

Fig. 15B

**Material examined.** S0219: A10106 (**PQ449268**).

**Localities.** Rio Bongo Scar (661 m).

**Remarks.** An undescribed species associated with a naturally occurring wood fall.

***Ophryotrocha* sp. SIO_BIC_ A10114**

Fig. 15C

**Material examined.** S0219: A10114 (MT435596), MZUCR 1511-01.

**Localities.** Rio Bongo Scar (~ 480–650 m).

**Remarks.** An undescribed species to be described in a forthcoming study.

***Parougiaceruleibohnorum* Yen & Rouse, 2020**

Fig. 15D

**Reference.**Yen and Rouse 2020**.

**Localities.** Mound 12 (996 m), Parrita Seep (~ 1401–1419 m; type locality).

**Distribution.** Known only from the CRM seeps.

**Parougiacf.billiemiroae Yen & Rouse, 2020**

Fig. 15E

**Reference.**Yen and Rouse 2020.

**Localities.** Jacó Scar (1796 m).

**Remarks.***Parougiabilliemiroaesensu stricto* is found at seeps, 587–1583 m, from Hydrate Ridge off Oregon (type locality) to the Guaymas Basin, Gulf of California (Yen and Rouse 2020). As discussed in Yen and Rouse (2020), the CRM single specimen A9678 is morphologically indistinguishable from *P.billiemiroaesensu stricto*, but its COI haplotype is up to 5.8% divergent from the type series and has been conservatively designated P.cf.billiemiroae.

**Parougiacf.sulleyi Yen & Rouse, 2020**

Fig. 15F

**Reference.**Yen and Rouse 2020.

**Localities.** Mound 12 (~ 987–997 m).

**Remarks.***P.sulleyisensu stricto* is found at seeps, 600–1600 m, from Hydrate Ridge off Oregon to the Guaymas Basin, Gulf of California (type locality) (Yen and Rouse 2020). As discussed in Yen and Rouse (2020), the CRM specimens are morphologically indistinguishable from *P.sulleyisensu stricto* but share a COI haplotype that is 7.4% divergent from the type series. Parougiacf.sulleyi is the same taxon as the “*Dorvillea* sp.” documented by Thurber et al. (2012) as a consumer of archaea at the CRM seeps, representing one of the first records of “archivory” by a metazoan (Yen and Rouse 2020).

***Parougiatheloniousblueski* Yen & Rouse, 2020**

Fig. 15G

**Reference.**Yen and Rouse 2020**.

**Localities.** Mound 12 (987–997 m), Mound 11 (1010 m; type locality).

**Distribution.** Known only from the CRM seeps.

#### ﻿Annelida | Polychaeta | Errantia | Aciculata incertae sedis | Myzostomida

***Eenymeenymyzostoma* sp. SIO_BIC_A8428**

Figs 15H, 16A

**Figure 16.**
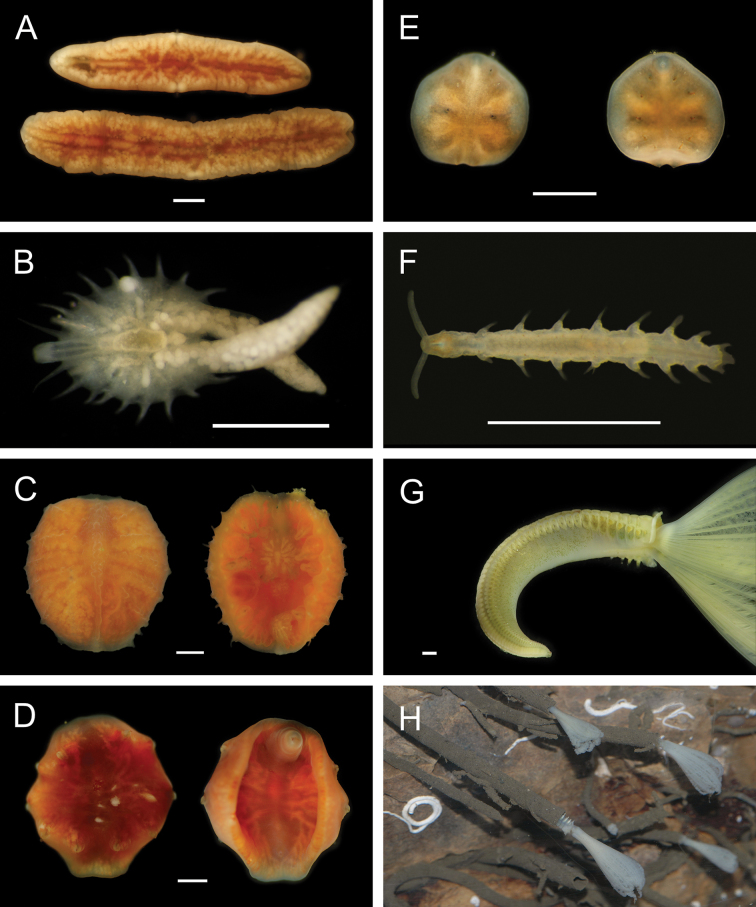
Annelida: Myzostomida, Nerillidae, and Sabellidae, representative live images **A***Eenymeenymyzostoma* sp. SIO_BIC_A8428 (A8428, ventral view) **B***Myzostomajosefinae* (A8362) **C***Pulvinomyzostomuminaki* (A1579, dorsal and ventral views of female with dwarf males) **D***Pulvinomyzostomum* sp. SIO_BIC_A8361 (A8361, dorsal and ventral views of female) **E***Pulvinomyzostomum* sp. SIO_BIC_A8361 (A8361, dorsal and ventral views of male) **F**Nerillidae stet. (A9797) **G***Bispira* sp. SIO_BIC_A9587 (A1396) **H***Bispira* sp. SIO_BIC_A9587 (A1460, in tubes attached to rocks photographed *ex situ* in the shipboard laboratory). Scale bars: 1 mm.

**Material examined.** AD4501: A1476; AD4503: A1340; AD4922: A8427 (**PQ449309**), A8428, A8431.

**Localities.** Mound 12 (~ 966–995 m).

**Remarks.** An undescribed species associated with the antipatharian coral *Lillipathesritamariae*.

***Myzostomajosefinae* Summers & Rouse in Summers et al. 2014**

Fig. 16B

**Material examined.** AD4913: A8362.

**Localities.** Jacó Scar (1878 m).

**Distribution.** Originally described from a whale fall at 1020 m in Monterey Submarine Canyon, off California (type locality) and in the vicinity of sedimented vents and seeps at ~ 1350 m in the Guaymas Basin, Gulf of California (Summers et al. 2014b).

**New records.** The CRM specimen represents a new southern record and a new maximum depth record for this species.

**Remarks.** The specimen, showing the distinctive paired elongate caudal appendages, was associated with the crinoid Psathyrometracf.fragilis (E7034), consistent with previous records of *M.josefinae* on *P.fragilis* (Summers et al. 2014b).

***Pulvinomyzostomuminaki* Summers & Rouse in Summers et al. 2014**

Fig. 16C

**Reference.**Summers et al. 2014b**.

**Localities.** Jacó Scar (1789 m; type locality).

**Distribution.** Known only from the CRM seeps.

**Remarks.** Associated with an antedonid crinoid (E4399) (Summers et al. 2014b).

***Pulvinomyzostomum* sp. SIO_BIC_A8361**

Fig. 16D, E

**Material examined.** AD4913: A8361 (**PQ449303**).

**Localities.** Jacó Scar (1878 m)

**Remarks.** An undescribed species. One female and one male were associated with the crinoid Psathyrometracf.fragilis (E7034).

#### ﻿Annelida | Polychaeta | Errantia | Aciculata incertae sedis | Nerillidae

**Nerillidae stet.**

Fig. 16F

**Material examined.** AD4979: A9797.

**Localities.** Quepos Slide (~ 380–395 m).

#### ﻿Annelida | Polychaeta | Sedentaria | Canalipalpata | Sabellida | Sabellidae

***Bispira* sp. SIO_BIC_A9587**

Figs 16G, H, 17A

**Figure 17.**
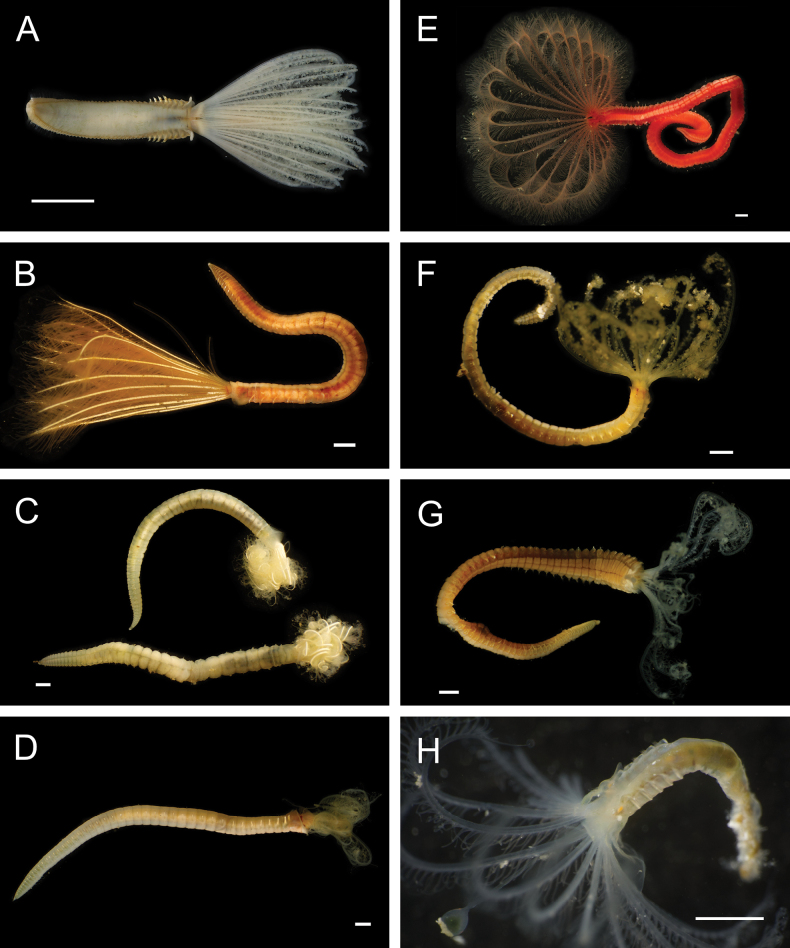
Annelida: Sabellidae and Serpulidae, representative live images **A***Bispira* sp. SIO_BIC_A9587 (A9587) **B***Chone* sp. SIO_BIC_A8422 (A8422) **C***Chone* sp. SIO_BIC_A8462 (A8462) **D***Jasmineira* stet. (A8282) **E***Pseudopotamilla* sp. SIO_BIC_A1455 (A1455) **F***Pseudopotamilla* sp. SIO_BIC_A9732 (A9732) **G**Sabellidae sp. SIO_BIC_ A8286 (A8286) **H***Hyalopomatus* sp. SIO_BIC_A1434 (A1434). Scale bars: 1 cm (**A**); 1 mm (**B–H**).

**Reference.**Goffredi et al. (2020) for characterization of bacterial symbiosis; specimen A9587 was erroneously cited as A9598.

**Material examined.** AD4508: A1396; AD4513: A1460 (**PQ449282**; 18S: **PQ304645**); AD4916: A8387; AD4971: A9587.

**Localities.** Parrita Seep (1402 m; this study), Jacó Scar (~ 1604–1854 m).

**Remarks.** An undescribed species, abundant at zones of active seepage and utilizing methanotrophic bacterial symbionts for nutrition (Goffredi et al. 2020).

***Chone* sp. SIO_BIC_A8422**

Fig. 17B

**Material examined.** AD4918: A8419; AD4919: A8422.

**Localities.** Quepos Slide (~ 379–410 m).

**Remarks.** Likely an undescribed species (María Ana Tovar-Hernández, pers. comm. 8 August 2020).

***Chone* sp. SIO_BIC_A8462**

Fig. 17C

**Material examined.** AD4916: A8389; AD4919: A8462 (16S: **PQ304651**).

**Localities.** Parrita Seep (~ 1400–1410 m), Jacó Scar (1746 m).

**Remarks.** Likely an undescribed species (María Ana Tovar-Hernández, pers. comm. 8 August 2020).

***Jasmineira* stet.**

Fig. 17D

**Material examined.** AD4907: A8282.

**Localities.** Mound 12 (999 m).

***Pseudopotamilla* sp. SIO_BIC_A1455**

Fig. 17E

**Material examined.** AD4512: A1455; AD4918: A8418; AD4979: A9796.

**Localities.** Quepos Slide (~ 338–411 m).

**Remarks.** An undescribed species.

***Pseudopotamilla* sp. SIO_BIC_A9732**

Fig. 17F

**Material examined.** AD4978: A9732.

**Localities.** Mound 12 (~ 996–999 m).

**Sabellidae sp. SIO_BIC_A8286**

Fig. 17G

**Material examined.** AD4908: A8286 (18S: **PQ304646**), A8287.

**Localities.** Mound 12 (1000 m).

#### ﻿Annelida | Polychaeta | Sedentaria | Canalipalpata | Sabellida | Serpulidae

***Hyalopomatus* sp. SIO_BIC_A1434**

Fig. 17H

**Reference.**Rouse and Kupriyanova (2021) for DNA sequences and phyloge­netic analysis.

**Material examined.** AD4510: A1434.

**Localities.** Jacó Summit (741–745 m).

**Remarks.** Possibly an undescribed species (Rouse and Kupriyanova 2021).

***Laminatubusjoycebrooksae* Rouse & Kupriyanova, 2021**

Fig. 18A

**Figure 18.**
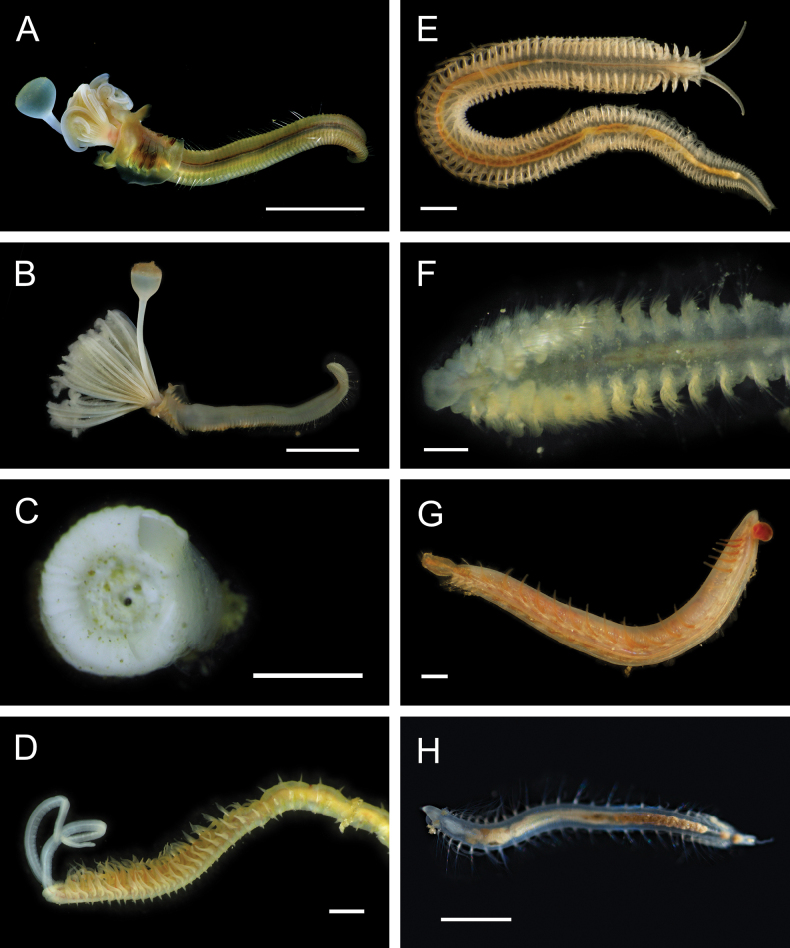
Annelida: Serpulidae, Spionidae, and Opheliidae, representative live images **A***Laminatubusjoycebrooksae* (A1315) **B***Laminatubuspaulbrooksi* (MZUCR 1506-01) **C**Spirorbinae stet. (A1385, body not visible) **D***Aonides* sp. SIO_BIC_A1344 (A1344) **E***Lindaspiodibranchiata* (A10053) **F***Prionospio* stet. (A1415) **G**Opheliidae stet. (A9938) **H***Ophelina* sp. 3 sec. Law et al. 2014 (A1898). Scale bars: 1 cm (**A, B**); 1 mm (**C–H**).

**Reference.**Rouse and Kupriyanova 2021**.

**Localities.** Mound 12 (~ 1000 m; type locality).

**Distribution.** Known only from the CRM seeps.

***Laminatubuspaulbrooksi* Rouse & Kupriyanova, 2021**

Fig. 18B

**Reference.**Rouse and Kupriyanova 2021**.

**Localities.** Parrita Seep (1402 m), Jacó Scar (~ 1800 m; type locality).

**Distribution.** Also known from seeps in the Guaymas Basin and Pescadero Basin, Gulf of California, 1565–2478 m (Rouse and Kupriyanova 2021).

**Remarks.** Abundant at zones of active seepage, this species utilizes methanotrophic bacterial symbionts for nutrition (Goffredi et al. 2020).

***Protis* stet.**

**Material examined.** AD4506: A1553 (18S: **PQ304668**; no image available).

**Localities.** Parrita Seep (1186 m).

**Remarks.** We thank Elena Kupriyanova (Australian Museum Research Institute) for generating the 18S sequence.

**Spirorbinae stet.**

Fig. 18C

**Material examined.** AD4507: A1385.

**Localities.** Parrita Scar (~ 1659–1667 m).

#### ﻿Annelida | Polychaeta | Sedentaria | Canalipalpata | Spionida | Spionidae

***Aonides* sp. SIO_BIC_A1344**

Fig. 18D

**Material examined.** AD4503: A1344; AD4505: A1372, MZUCR 1509-01.

**Localities.** Mound 12 (~ 967–995 m), Mound 11 (~ 1019–1025 m).

**Remarks.** An undescribed species to be described in a forthcoming study.

***Lindaspiodibranchiata* Blake & Maciolek, 1992**

Fig. 18E

**Material examined.** AD4511: A1453 (**PQ449281**); AD4972: A9601 (**PQ449316**); S0213: A10053 (**PQ449255**).

**Localities.** Jacó Summit (~ 730–820 m), Mound 12 (989 m), Jacó Scar (~ 1791–1800 m).

**Distribution.** Originally described from sedimented vents in the Guaymas Basin, Gulf of California, 2004–2008 m (Blake and Maciolek 1992).

**New records**: The CRM specimens represent new southern records, the first records from seeps, and, for specimen A10053, a new minimum depth for this species (820 m as the most conservative value). The COI sequences for the Costa Rica specimens were identical to that of a specimen from the type locality: A3258 (**PQ432663**) from 1581 m at Pinkie’s "Vent", Guaymas Basin.

***Prionospio* stet.**

Fig. 18F

**Material examined.** AD4509: A1415; AD4979: A9809.

**Localities.** Quepos Slide (393 m), Jacó Scar (1855 m).

**Remarks.** These specimens are members of the *Prionospio* complex; the shape of the head of A1415 is consistent with *Apoprionospio*, but some sources place this genus into synonymy with *Prionospio* (Vasily Radashevsky, pers. comm. 8 July 2021).

#### ﻿Annelida | Polychaeta | Sedentaria | Canalipalpata | Capitellida | Opheliidae

**Opheliidae stet.**

Fig. 18G

**Material examined.** AD4971: A9651; AD4972: A9596; AD4977: A9730; AD4989: A9938 (**PQ449337**).

**Localities.** Jacó Scar (1783–1796 m).

**Remarks.** A9651 likely represents an undescribed species of *Ophelina* (Sergio Salazar-Vallejo, pers. comm. 12 February 2020).

***Ophelina* sp. 3 sec. Law et al. 2014**

Fig. 18H

**Reference.**Law et al. (2014), in which GenBank sequences published under the temporary code F14588 (KF511809, KF511823, KF511847, KF511864) correspond to specimen A1898 from AD4587.

**Localities.** Mound 12 (~ 990–996 m).

#### ﻿Annelida | Polychaeta | Sedentaria | Canalipalpata | Capitellida | Capitelliformia | Thalassematidae

We refer to the clade formerly known as Echiura using the revised taxonomy of Goto et al. (2020).

***Prometor* stet.**

Fig. 19A

**Figure 19.**
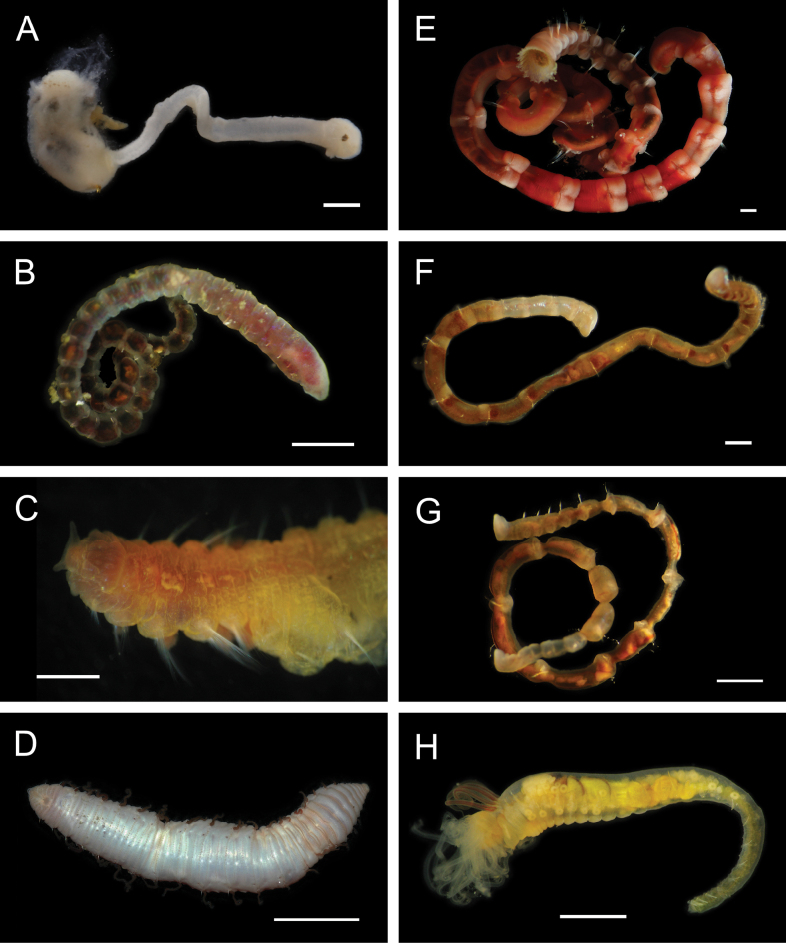
Annelida: Thalassematidae, Capitellidae, Scalibregmatidae, Maldanidae, and Ampharetidae, representative live images **A***Prometor* stet. (A9638) **B**Capitellidae stet. (A1418) **C***Scalibregma* stet. (A1391) **D***Travisia* stet. (A1386) **E**Nicomachecf.lokii (A8257) **F***Notoproctus* sp. SIO_BIC_A9801 (A9801) **G***Notoproctus* sp. SIO_BIC_A9802 (A9802) **H**Ampharetini stet. (A1377). Scale bars: 1 mm (**A–C, E–H**); 1 cm (**D**).

**Material examined.** AD4971: A9638 (**PQ449318**).

**Localities.** Jacó Scar (1824 m).

#### ﻿Annelida | Polychaeta | Sedentaria | Canalipalpata | Capitellida | Capitelliformia | Capitellidae

**Capitellidae stet.**

Fig. 19B

**Material examined.** AD4506: A1376; AD4508: A1403; AD4509: A1418; AD4976: A9755 (16S: **PQ304666**).

**Localities.** Parrita Seep (~ 1186–1419 m), Jacó Scar (~ 974–1887 m).

**Remarks.** Associated with vesicomyid clams (A1376, A1418), *Lamellibrachiabarhami* (A1403), or experimentally deployed wood (A9755).

#### ﻿Annelida | Polychaeta | Sedentaria | Canalipalpata | Terebellida | Scalibregmatidae

***Scalibregma* stet.**

Fig. 19C

**Material examined.** AD4507: A1391 (**PQ449279**); AD4914: A8385.

**Localities.** Parrita Scar (~ 1659–1667 m), Jacó Scar (1886 m).

***Travisia* stet.**

Fig. 19D

**Material examined.** AD4507: A1386.

**Localities.** Parrita Scar (~ 1659–1663 m).

#### ﻿Annelida | Polychaeta | Sedentaria | Canalipalpata | Terebellida | Maldanomorpha | Maldanidae

**Nicomachecf.lokii Kongsrud & Rapp, 2012**

Fig. 19E

**Material examined.** AD4504: A1350 (**PQ450403**); AD4509: A1420 (**PQ450404**); AD4906: A8257; AD4911: A8295.

**Localities.** Mound 11 (~ 1002–1011 m), Mound 12 (995–1001 m), Jacó Scar (~ 974–1856 m).

**Remarks.** The closest COI BLASTN results on GenBank were sequences of *Nicomachelokii* (95.03–98.17% identity to A1350, 95.18–98.31% identity to A1420), and the closest matches (MG975502.1, FR877578.1) were from the type locality of the Loki’s Castle vent system on the Arctic Mid-Ocean Ridge at 2350 m (Kongsrud and Rapp 2012; Eilertsen et al. 2018). *Nicomachelokii* shows genetic connectivity across vents and seeps in the Arctic, Barbados Trench, and East Scotia Sea, 1262–4930 m, with a maximum intraspecific pairwise distance of 4.1% (K2P) (Eilertsen et al. 2018), so further genetic and morphological comparisons to the CRM specimens are warranted.

***Notoproctus* sp. SIO_BIC_A9801**

Fig. 19F

**Material examined.** AD4978: A9801 (**PQ449327**).

**Localities.** Mound 12 (997 m).

**Remarks.** This specimen showed a COI difference of 19.1% (uncorrected) from A9802 (below) so we regard them as separate species.

***Notoproctus* sp. SIO_BIC_A9802**

Fig. 19G

**Material examined.** AD4978: A9802 (**PQ449328**).

**Localities.** Mound 12 (997 m).

#### ﻿Annelida | Polychaeta | Sedentaria | Canalipalpata | Terebellida | Terebelliformia | Ampharetidae

**Ampharetini stet.**

Fig. 19H

**Material examined.** AD4506: A1377 (**PQ450382**).

**Localities.** Parrita Seep (1030 m).

***Amphisamythafauchaldi* Solís-Weiss & Hernández-Alcántara, 1994**

Fig. 20A

**Figure 20.**
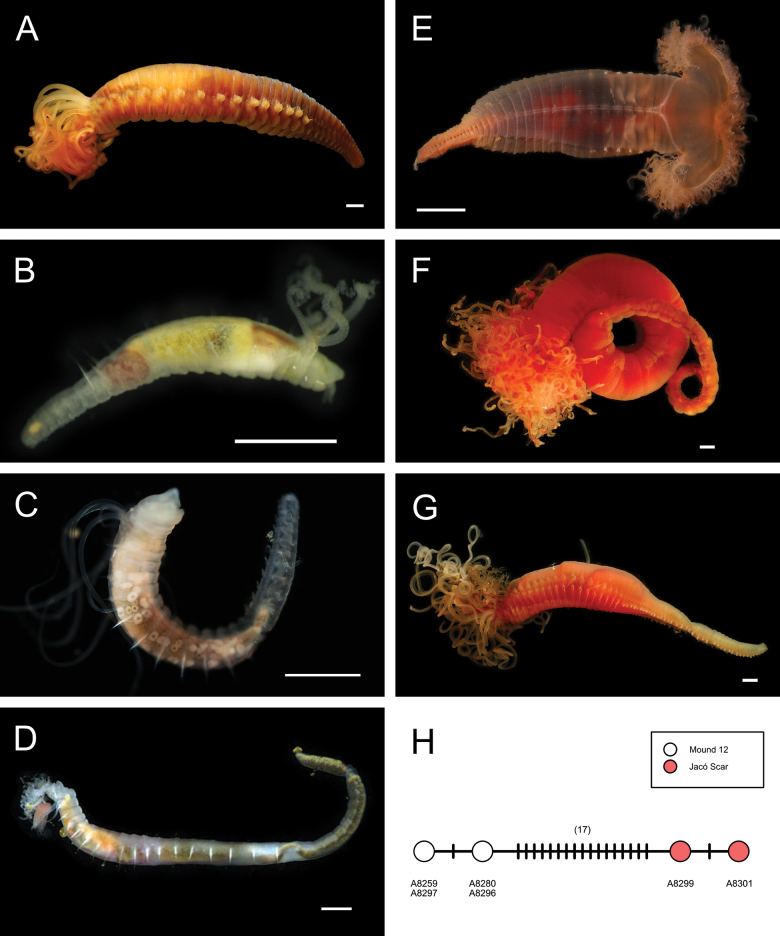
Annelida: Ampharetidae, Trichobranchidae, and Terebellidae, representative live images **A***Amphisamythafauchaldi* (A8352) **B**Grassleiacf.hydrothermalis (A12401) **C***Pavelius* sp. EP-B sec. Stiller et al. 2020 (A1894) **D***Terebellides* stet. (A1293) **E***Biremis* sp. SIO_BIC_A10093 (A10093) **F***Polycirrus* stet. (A10115) **G**Eupolymniacf.heterobranchia (A8296) **H** Haplotype network of Eupolymniacf.heterobranchiaCOI sequences. Scale bars: 1 mm (**A–D, F, G**); 1 cm (**E**).

**References.**Levin et al. 2012; Stiller et al. 2013.

**Additional material examined.** AD4906: A8260 (**PQ449288**, **PQ449289**); AD4912: A8352 (**PQ449299**, **PQ449300**); AD4913: A8358 (**PQ449302**); AD4914: A12602 (**PQ449273**).

**Localities.** Mound 12 (~ 1000 m), Jacó Scar (~ 1800 m).

**Distribution.** Originally described from sedimented hydrothermal vents at 2000 m in the Guaymas Basin, Gulf of California (Solís-Weiss and Hernández-Alcántara 1994), *A.fauchaldi* is also found at seeps at Hydrate Ridge off Oregon, with an overall depth range of 600–2860 m (Stiller et al. 2013).

**Grassleiacf.hydrothermalis Solís-Weiss, 1993**

Fig. 20B

**Material examined.** AD4510: A12401 (**PQ449272**).

**Localities.** Jacó Summit (744 m).

**Remarks.** The closest COI BLASTN result on GenBank was a specimen identified as Grassleiacf.hydrothermalis from seeps at 1572–1583 m in the Guaymas Basin, Gulf of California (KX497032.1, 96.51% identity). *Grassleiahydrothermalis* was described from vents at the Escabana Trough, Gorda Ridge, off northern California, at 3271 m (Solís-Weiss 1993), and has been reported from seeps at 595 m off the Cascadia Margin (Reuscher et al. 2012). Genetic sampling from the type locality will be important to ascertain the distribution of this species.

***Pavelius* sp. EP-B sec. Stiller et al. 2020**

Fig. 20C

**Material examined.** AD4586: A1887 (**PQ449284**); AD4587: A1894 (**PQ449285**).

**Localities.** Mound 12 (~ 982–998 m).

**Remarks.** The CRM specimens represent records of an undescribed species also reported from seeps at Hydrate Ridge off Oregon, 809 m, as cited in supplementary table S2 of Stiller et al. (2020).

#### ﻿Annelida | Polychaeta | Sedentaria | Canalipalpata | Terebellida | Terebelliformia | Trichobranchidae

***Terebellides* stet.**

Fig. 20D

**Material examined.** AT15-44 MC1-2: A1293.

**Localities.** Near Mound 12 (1019 m).

**Remarks.** This specimen was collected in the upper 1 cm of a sediment core adjacent to Mound 12, ca 500 m from known sites of active seepage and likely representing the far-transition zone to the surrounding environment.

#### ﻿Annelida | Polychaeta | Sedentaria | Canalipalpata | Terebellida | Terebelliformia | Terebellidae | Polycirrini

***Biremis* sp. SIO_BIC_A10093**

Fig. 20E

**Material examined.** S0218: A10091, A10092, A10093.

**Localities.** Parrita Scar (1110–1470 m).

**Remarks.** An undescribed species.

***Polycirrus* stet.**

Fig. 20F

**Material examined.** S0219: A10067, A10097, A10115 (**PQ449270**).

**Localities.** Rio Bongo Scar (609–659 m).

**Remarks.** These specimens were buried or partially buried in soft sediments with tentacles extended.

#### ﻿Annelida | Polychaeta | Sedentaria | Canalipalpata | Terebellida | Terebelliformia | Terebellidae | Procleini

**Eupolymniacf.heterobranchia (Johnson, 1901)**

Fig. 20G

**Material examined.** AD4906: A8259 (**PQ449287**); AD4910: A8280 (**PQ449291**), A8296 (**PQ449294**), A8297 (**PQ450386**); AD4911: A8299 (**PQ449295**), A8301 (**PQ449296**).

**Localities.** Mound 12 (998–1002 m), Jacó Scar (1758 m).

**Remarks.** Likely an undescribed species, with morphological similarity to *Eupolymniaheterobranchia*, known from shallow waters from Alaska to Mexico (Banse 1980). The COI sequences showed low identity (~ 80–82%) to GenBank sequences of *E.heterobranchia* from the type locality of Puget Sound (Johnson 1901) (HQ932678.1, HM473379.1, HM473380.1, MN138388.1). *Eupolymnia* is paraphyletic, and nomenclatural revision requires further phylogenetic sampling (Stiller et al. 2020).

The CRM specimens appeared to show genetic structure between Mound 12 and Jacó Scar, with 3.2–3.6% COI divergence (uncorrected, corresponding to 17–19 bp) between localities and a maximum divergence of only < 0.2% (1 bp) within localities (Fig. 20H). We conservatively consider the Mound 12 and Jacó Scar populations to represent the same taxon, pending further sampling and morphological investigation. Yet we acknowledge the possibility of depth-segregated cryptic species, given that the depth-spanning sympatric scaleworm sister species *Branchipolynoehalliseyae* and *B.kajsae* are separated by a comparable COI distance (3.7% uncorrected) (Lindgren et al. 2019).

#### ﻿Annelida | Polychaeta | Sedentaria | Canalipalpata | Terebellida | Terebelliformia | Terebellidae | Terebellini

**Neoamphitritecf.hydrothermalis Reuscher, Fiege & Wehe, 2012**

Fig. 21A

**Figure 21.**
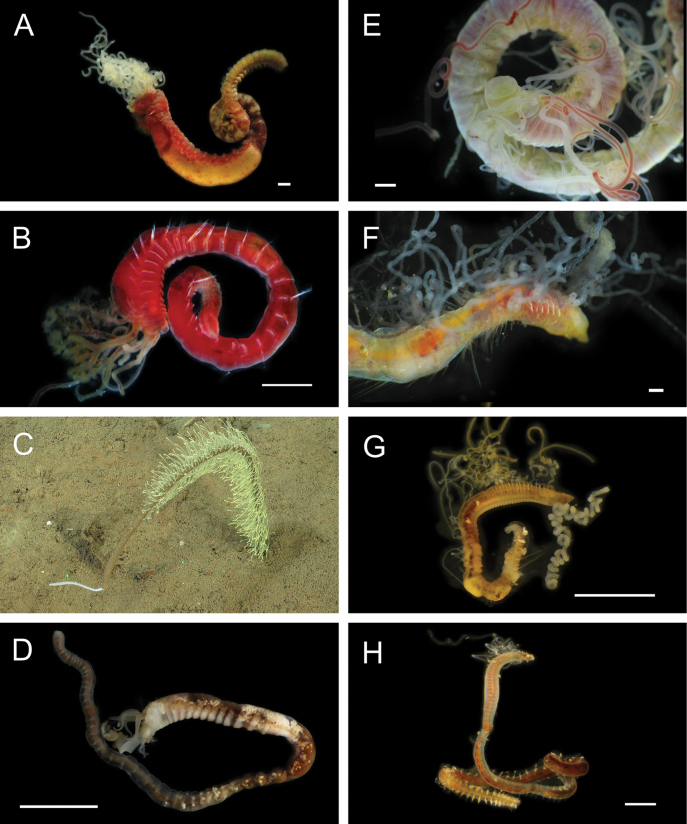
Annelida: Terebellidae, Melinnidae, and Cirratulidae, representative live images **A**Neoamphitritecf.hydrothermalis (A1351) **B**Neoamphitritecf.robusta (A1456) **C**Melinnopsiscf.armipotens (A12604, *in situ*, tentacle protruding from the tube extending from the sediment and covered with hydroids, Co3088). Credit: ROV SuBastian/Schmidt Ocean Institute **D**Melinnopsiscf.armipotens (A12604) **E***Aphelochaeta* sp. SIO_BIC_A1380 (A1380) **F***Aphelochaeta* sp. SIO_BIC_A1380 (A1429) **G***Aphelochaeta* sp. SIO_BIC_A9729 (A9729) **H***Chaetozone* sp. SIO_BIC_A9846 (A9846). Scale bars: 1 mm (**A, B, E–H**); 1 cm (**D**).

**Material examined.** AD4504: A1351 (**PQ450387**).

**Localities.** Mound 11 (1010 m).

**Remarks.** Likely an undescribed species, morphologically similar to *Neoamphitritehydrothermalis*, which is known from western Pacific hydrothermal vents in the Lihir Basin, 1474–1480 m (Reuscher et al. 2012). The COI sequence did not closely match any available GenBank reference sequences (<83% identity).

**Neoamphitritecf.robusta (Johnson, 1901)**

Fig. 21B

**Material examined.** AD4512: A1456 (**PQ450389**); AD4588: A2150 (**PQ449286**).

**Localities.** Quepos Slide (400 m), Mound 12 (~ 995–997 m).

**Remarks.** Likely an undescribed species. Morphologically similar to *Neoamphitriterobusta*, which was described from shallow water in Puget Sound (Johnson 1901) and occurs in the eastern Pacific to depths of at least 1984 m (Hartman and Barnard 1958), including Pacific Costa Rica at 22 m (Wehrtmann and Cortés 2009b). The COI sequences did not closely match any available GenBank reference sequences (<81% identity).

#### ﻿Annelida | Polychaeta | Sedentaria | Canalipalpata | Terebellida | Terebelliformia | Melinnidae

**Melinnopsiscf.armipotens (Moore, 1923)**

Fig. 21C, D

**Material examined.** S0220: A12604 (**PQ449274**).

**Localities.** Subduction Plume (3502 m).

**Remarks.** The tubes of these animals protruded above the sediment and were encrusted with *Candelabrum* hydroids (Co3088). They warrant comparison to *Melinnopsisarmipotens*, known only from southern California, 4075 m (Moore 1923).

#### ﻿Annelida | Polychaeta | Sedentaria | Canalipalpata | Cirratulida | Cirratulidae

We thank Jim Blake (Aquatic Research & Consulting) for morphological identification of these specimens. These specimens will be further discussed in a separate work.

***Aphelochaeta* sp. SIO_BIC_A1380**

Fig. 21E, F

**Material examined.** AD4506: A1380 (**PQ449000**), AD4510: A1429.

**Localities.** Jacó Summit (741 m), Parrita Seep (1186 m).

***Aphelochaeta* sp. SIO_BIC_A9729**

Fig. 21G

**Material examined.** AD4977: A9729 (**PQ449323**).

**Localities.** Jacó Scar (1783 m).

**Remarks.** Likely an undescribed species.

***Chaetozone* sp. SIO_BIC_A9846**

Fig. 21H

**Material examined.** AD4987: A9846 (**PQ449331**).

**Localities.** Mound 12 (999 m).

**Remarks.** An undescribed species.

***Cirratulus* stet.**

Fig. 22A

**Figure 22.**
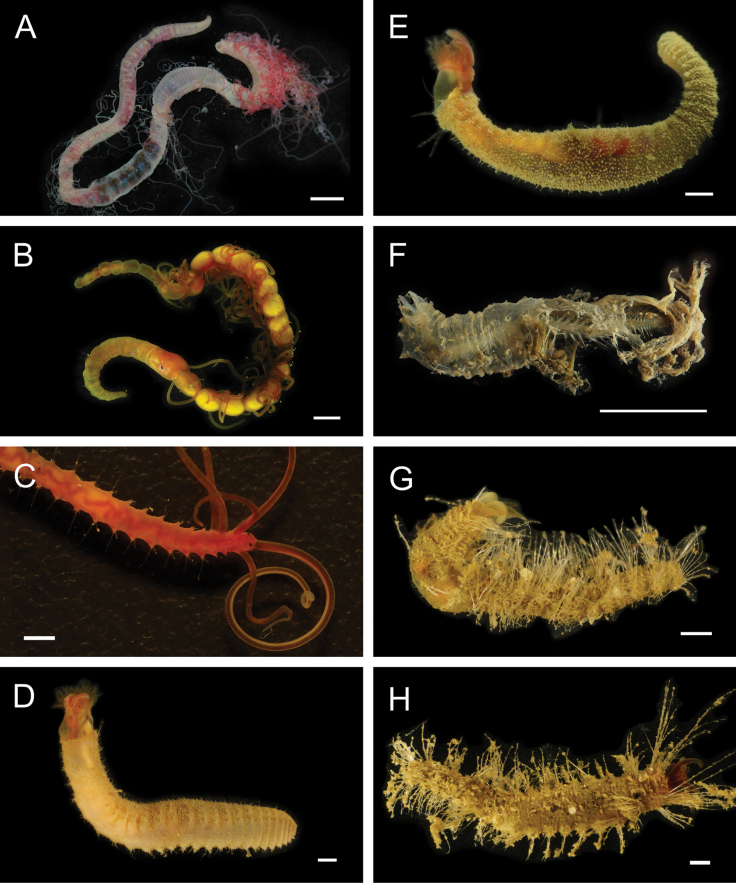
Annelida: Cirratulidae and Flabelligeridae, representative live images **A***Cirratulus* stet. (A1435) **B**Raricirruscf.maculatus (A1342) **C***Macrochaeta* stet. (A8420) **D**Bradabyssacf.pilosa (A9902) **E***Bradabyssa* sp. SIO_BIC_A1356 (A1356) **F**Flabelligeracf.bophortica (A9751) **G***Lamispinapolycerata* (MZUCR 1504-01) **H***Saphobranchiacanela* (A9607). Scale bars: 1 cm (**A, F**); 1 mm (**B–E, G, H**).

**Material examined.** AD4510: A1435 (**PQ449280**); AD4912: A8303 (**PQ449297**); AD4973: A9677 (**PQ449320**); AD4989: A9907 (**PQ449334**), A9962 (**PQ449339**); S0213: A10051 (**PQ449254**).

**Localities.** Jacó Summit (~ 741–744 m), Jacó Scar (~ 1768–1811 m).

**Remarks.** Multiple species may be represented.

**Raricirruscf.maculatus Hartman, 1961**

Fig. 22B

**Material examined.** AD4503: A1342 (**PQ449276**).

**Localities.** Mound 12 (990 m).

**Remarks.** Possibly *Raricirrusmaculatus*, described from southern California, 46–70 m (Hartman 1961).

#### ﻿Annelida | Polychaeta | Sedentaria | Canalipalpata | Cirratulida | Acrocirridae

***Macrochaeta* stet.**

Fig. 22C

**Material examined.** AD4512: A1457; AD4918: A8420 (**PQ449307**); AD4985: A9854.

**Localities.** Quepos Slide (~ 338–411 m), Mound 12 (~ 995–1002 m).

#### ﻿Annelida | Polychaeta | Sedentaria | Canalipalpata | Cirratulida | Flabelligeridae

We thank Sergio Salazar-Vallejo (El Colegio de la Frontera Sur) for morphological identification of these specimens.

**Bradabyssacf.pilosa (Moore, 1906)**

Fig. 22D

**Material examined.** AD4987: A9840 (**PQ449330**), A9902 (**PQ449333**).

**Localities.** Mound 12 (1010–1012 m).

**Remarks.***B.pilosa* has been recorded from Alaska (type locality, 362–573 m) to Baja California, 40–1800 m, including seeps off Oregon and southern California (Salazar-Vallejo 2017). If confirmed as *B.pilosa* by genetic comparison to material from the type locality, the CRM specimens would represent a new southern record for the species. A9840 was associated with parasitic copepods (C14494), likely Bradophilidae.

***Bradabyssa* sp. SIO_BIC_A1356**

Fig. 22E

**Material examined.** AD4504: A1356 (**PQ449277**); AD4590: A1962 (**PQ450390**).

**Localities.** Mound 11 (~ 1004–1011 m), Jacó Scar (~ 1791–1800 m).

**Flabelligeracf.bophortica Annenkova-Chlopina, 1924**

Fig. 22F

**Material examined.** AD4512: A1458; AD4976: A9751 (**PQ449324**).

**Localities.** Quepos Slide (~ 344–411 m), Jacó Scar (1887 m).

**Remarks.** These specimens resemble *F.bophortica*, which was described from the Chukchi Sea, Arctic Ocean, 13–48 m (Salazar-Vallego 2012). The main morphological difference is that in *F.bophortica* the dorsum is areolate, whereas in the CRM specimens it is smooth (Sergio Salazar-Vallejo, pers. comm. 12 February 2020). Specimen A9751 was associated with experimentally deployed wood.

***Lamispinapolycerata* Salazar-Vallejo, 2020**

Fig. 22G

**Reference.**Salazar-Vallejo 2020a**.

**Localities.** Mound 12 (999 m; type locality).

**Distribution.** Known only from the CRM seeps.

***Saphobranchiacanela* Salazar-Vallejo, 2020**

Fig. 22H

**Reference.**Salazar-Vallejo 2020a**.

**Localities.** Mound 12 (~ 987–997 m; type locality), Jacó Scar (1785 m).

**Distribution.** Known only from the CRM seeps.

**Figure 23.**
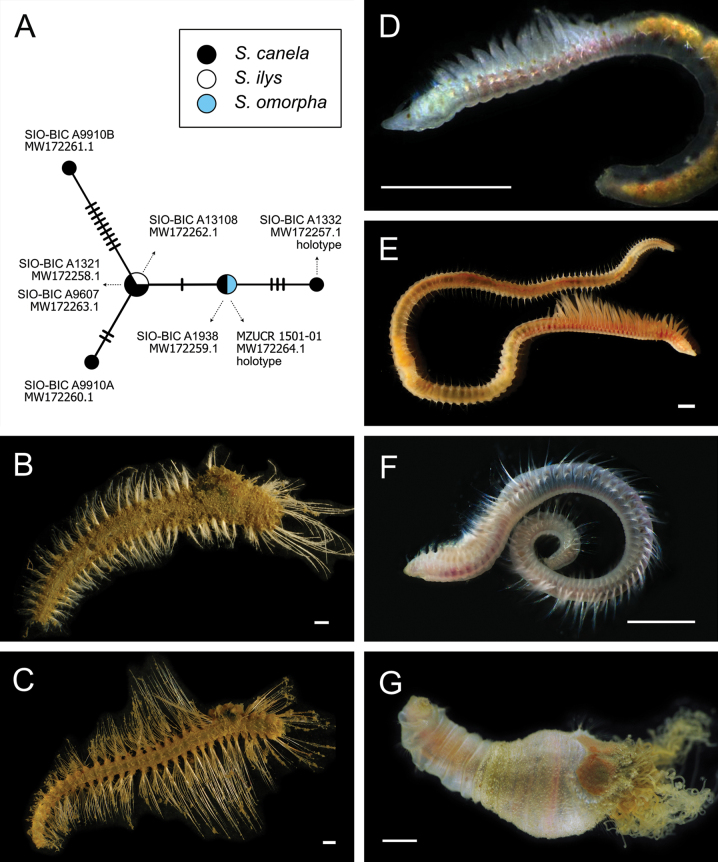
Annelida: Flabelligeridae, Paraonidae, and Sternaspidae, representative live images **A** haplotype network of COI sequences from the descriptions of *Saphobranchiacanela*, *S.ilys*, and *S.omorpha***B***Saphobranchiailys* (MZUCR 1503-01) **C***Saphobranchiaomorpha* (MZUCR 1501-01) **D***Aricideamirifica* (A1464) **E***Aricidearubra* (A10049) **F***Aricidea* sp. A sec. Langeneck et al. 2019 (A1970) **G***Sternaspis* stet. (A1473). Scale bars: 1 mm.

**Remarks.***S.canela* is distinguished from *S.ilys* and *S.omorpha* based on morphology, but genetic data do not corroborate the delineation of three species, as acknowledged in the original description (Salazar-Vallejo 2020a). The COI variation between specimens described as *S.ilys* and *S.omorpha* is less than the variation within *S.canela* (Fig. 23A). Collection of additional specimens and investigation of potential polymorphic conditions will be important for further work.

***Saphobranchiailys* Salazar-Vallejo, 2020**

Fig. 23B

**Reference.**Salazar-Vallejo 2020a**.

**Localities.** Jacó Scar (1783–1784 m; type locality).

**Distribution.** Known only from the CRM seeps.

***Saphobranchiaomorpha* Salazar-Vallejo, 2020**

Fig. 23C

**Reference.**Salazar-Vallejo 2020a**.

**Localities.** Jacó Scar (1795 m; type locality).

**Distribution.** Known only from the CRM seeps.

#### ﻿Annelida | Polychaeta | Sedentaria | Canalipalpata | Sternaspida | Paraonidae

***Aricideamirifica* Strelzov, 1973**

Fig. 23D

**Reference.**Langeneck et al. (2019) for DNA sequences and phylogenetic analysis, in which GenBank sequences MH700680 and MH688926 correspond to specimen A1464.

**Material examined.** AT15-44 MC1-2: A1464.

**Localities.** Near Mound 12 (1019 m).

**Distribution.** Also reported from shallow Pacific waters of Costa Rica, 18–26 m, as well as the eastern and western Pacific and Antarctic (Wehrtmann and Cortés 2009b).

**Remarks.** This specimen was collected in a sediment core adjacent to Mound 12, ca 500 m from known sites of active seepage and likely representing the far-transition zone to the surrounding environment.

***Aricidearubra* Hartman, 1963**

Fig. 23E

**Reference.**Langeneck et al. (2019) for DNA sequences and phylogenetic analysis, in which GenBank sequences MH700679, MH688925, and MH700708 correspond to specimen A1616 from Mound 12.

**Material examined.** AD4511: A1616; S0213: A10049 (**PQ449252**).

**Localities.** Jacó Summit (~ 730–820 m; this study), Mound 12 (988 m).

**Distribution.** Originally described from submarine canyons in southern California, 603–1298 m (Hartman 1963) and recorded from the South Atlantic, eastern and western Pacific, and Scotia Sea (Blake 1996).

***Aricidea* sp. A sec. Langeneck et al. 2019**

Fig. 23F

**Reference.**Langeneck et al. (2019) for DNA sequences and phylogenetic analysis, in which GenBank sequences MH700686, MH688937, and MH700724 correspond to specimen A1970.

**Material examined.** AD4591: A1970.

**Localities.** Jacó Scar (~ 1752–1795 m).

**Remarks.** This specimen is an epitoke.

#### ﻿Annelida | Polychaeta | Sedentaria | Canalipalpata | Sternaspida | Sternaspidae

***Sternaspis* stet.**

Fig. 23G

**Reference.**Drennan et al. (2019) for phylogenetic analysis.

**Material examined.** AT15-44 MC1-2: A1473 (16S: MK810080; 18S: MK809977); AD4989: A9943, A9945 (**PQ449338**), A9948.

**Localities.** Near Mound 12 (1019 m), Jacó Scar (1762 m; this study).

**Remarks.** Based on 16S and 18S sequences, specimen A1473 is phylogenetically distinct from its closest reported relative, the Antarctic species *Sternaspissendalli* Salazar-Vallejo, 2014 (Drennan et al. 2019). Consistent with this result, the closest COI BLASTN results on GenBank for A9945 were specimens of *S.sendalli* (e.g., MK810006.1, NC_068907.1; up to 90.85% identity). A1473 was collected in a sediment core adjacent to Mound 12, ca 500 m from known sites of active seepage and likely representing the far-transition zone to the surrounding environment.

#### ﻿Annelida | Polychaeta | Sedentaria | Canalipalpata | Siboglinidae

***Escarpiaspicata* Jones, 1985**

Fig. 24A

**Figure 24.**
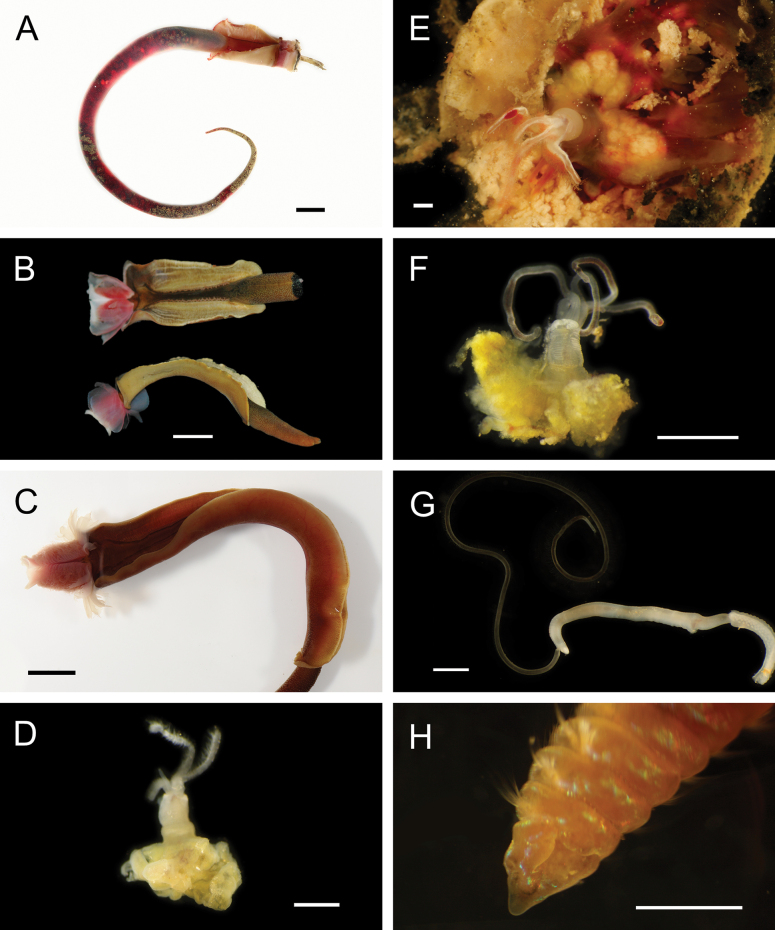
Annelida: Siboglinidae and Orbiniidae, representative live images **A***Escarpiaspicata* (A8364) **B***Lamellibrachiabarhami* (A1564, incomplete specimens) **C***Lamellibrachiadonwalshi* (A8382) **D***Osedaxfrankpressi* (A9592) **E***Osedaxfrankpressi* (A9594, in bone) **F***Osedaxknutei* (A9617) **G***Siboglinum* stet. (A8349) **H***Leitoscoloplos* sp. SIO_BIC_A9939 (A9939). Scale bars: 1 cm (**A–C**); 1 mm (**D–H**).

**References**. Levin et al. (2012, 2015) for occurrences at Mound 12, Jacó Scar, and Parrita Seep.

**Material examined.** AD4503: A1343; AD4507: A1390 (**PQ450388**); AD4511: A1445; AD4513: A1463; AD4590: A1826; AD4591: A1838; AD4914: A8364; AD4924: A8464; AD4971: A9618, A9619, A9620, A9621, A9622, A9623, A9624, A9625, A9626, A9627; AD4987: A9901; S0217: A10089; S0230: A10172.

**Localities.** Mound 12 (~ 1000 m), The Thumb (1074 m; this study), Parrita Seep (~ 1400 m), Parrita Scar (~ 1600 m; this study), Jacó Scar (~ 1800 m), Mound Jaguar (1909 m; this study).

**Distribution.** Originally described from seeps at 1829 m in the San Clemente Basin off California (Karaseva et al. 2016), *E.spicata* has been reported as far south as northern Chile (Kobayashi and Araya 2018). It has been recorded from a range of reducing environments and depths, including a whale fall at 1240 m near the type locality (Feldman et al. 1998); sedimented vents and seeps at 1568–2020 m in the Guaymas Basin, Gulf of California (Black et al. 1997; Karaseva et al. 2016); and a seep at 2756 m in the Middle America Trench, off southern Mexico (Southward et al. 2002).

**Remarks.** The three described species of *Escarpia* (*E.laminata* Jones, 1985; *E.southwardae* Andersen et al., 2004; *E.spicata* Jones, 1985) are morphologically distinguishable with separate geographic ranges (Karaseva et al. 2016), but the most commonly used DNA sequence markers (COI, 16S, CytB) do not provide reliable differentiation among these species due to low sequence variation (Black et al. 1997; McMullin et al. 2003; Cowart et al. 2013). Alternative marker sequences, such as the nuclear hemoglobin subunit B2 intron (HbB2i), have been used to support the existing species designations, but resolving their phylogenetic relationships remains complex (Cowart et al. 2013; Kobayashi and Araya 2018). Nevertheless, all eastern Pacific occurrences of *Escarpia* are presently accepted as *E.spicata* (Karaseva et al. 2016).

***Lamellibrachiabarhami* Webb, 1969**

Fig. 24B

**References.**McMullin et al. 2003; Han et al. 2004; Mau et al. 2006; Sahling et al. 2008; Levin et al. 2012, 2015; McCowin and Rouse 2018.

**Localities.** Parrita Seep (~ 1400 m), Jacó Scar (~ 1800–1890 m), Parrita Scar (~ 2200 m).

**Distribution.** Originally described from seeps at 1125 m off southern California (Webb 1969), *L.barhami* occurs at seeps and sedimented vents (Middle Valley, Juan de Fuca Ridge; Pinkie’s “Vent,” Guaymas Basin), from Vancouver Island, Canada, to northern Chile, 1000–2400 m (Black et al. 1997, 1998; Bright and Lallier 2010; Karaseva et al. 2016; Kobayashi and Araya 2018; McCowin and Rouse 2018). The species shows only minimal COI divergence (<0.2%) across its range of more than 10,000 km (Kobayashi and Araya 2018; McCowin and Rouse 2018).

***Lamellibrachiadonwalshi* McCowin & Rouse, 2018**

Fig. 24C

**Reference.**McCowin and Rouse 2018**.

**Localities.** Mound 12 (~ 1000 m; type locality), Mound 11 (~ 1040 m).

**Distribution.** Known only from the CRM seeps.

***Osedaxfrankpressi* Rouse, Goffredi & Vrijenhoek, 2004**

Fig. 24D, E

**Reference.**Berman et al. (2023) for DNA sequences and haplotype networks.

**Material examined.** AD4972: A9591 (OM994442), A9592 (OM994444), A9593 (OM994443), A9594 (OM994445).

**Localities.** Jacó Scar (1845 m).

**Distribution.** Originally described from Monterey Submarine Canyon off California (Rouse et al. 2004). Recorded in the eastern Pacific from the Oregon margin to the CRM as well as in the Atlantic off Brazil, 642–2891 m (Berman et al. 2023).

**Remarks.** Collected from experimentally deployed pig bones.

***Osedaxknutei* Rouse, Goffredi, Johnson & Vrijenhoek, 2018**

Fig. 24F

**Reference.**Berman et al. (2023) for DNA sequences and haplotype networks.

**Material examined.** AD4974: A9617 (ON041090).

**Localities.** Mound 12 (992 m).

**Distribution.** Monterey Submarine Canyon off California (type locality) to the CRM, 845–2898 m (Berman et al. 2023).

**Remarks.** Collected from experimentally deployed pig or cow bones.

***Siboglinum* stet.**

Fig. 24G

**Material examined.** AD4911: A8349 (**PQ449298**); AD4916: A8388; AD4989: A9942.

**Localities.** Jacó Scar (~ 1757–1892 m).

**Remarks.** Collected from sediment cores, except for A8349 which was associated with a rock substrate. The occurrence of a field of frenulate siboglinids at Jacó Scar was noted by Levin et al. (2012).

#### ﻿Annelida | Polychaeta | Sedentaria | Orbiniida | Orbiniidae

***Leitoscoloplos* sp. SIO_BIC_A9939**

Fig. 24H

**Material examined.** AD4989: A9939.

**Localities.** Jacó Scar (1785 m).

**Remarks.** This single damaged specimen likely represents an undescribed species (Jim Blake, pers. comm. 7 October 2019).

#### ﻿Annelida | Polychaeta | Sedentaria incertae sedis

***Cossura* stet.**

Fig. 25A

**Figure 25.**
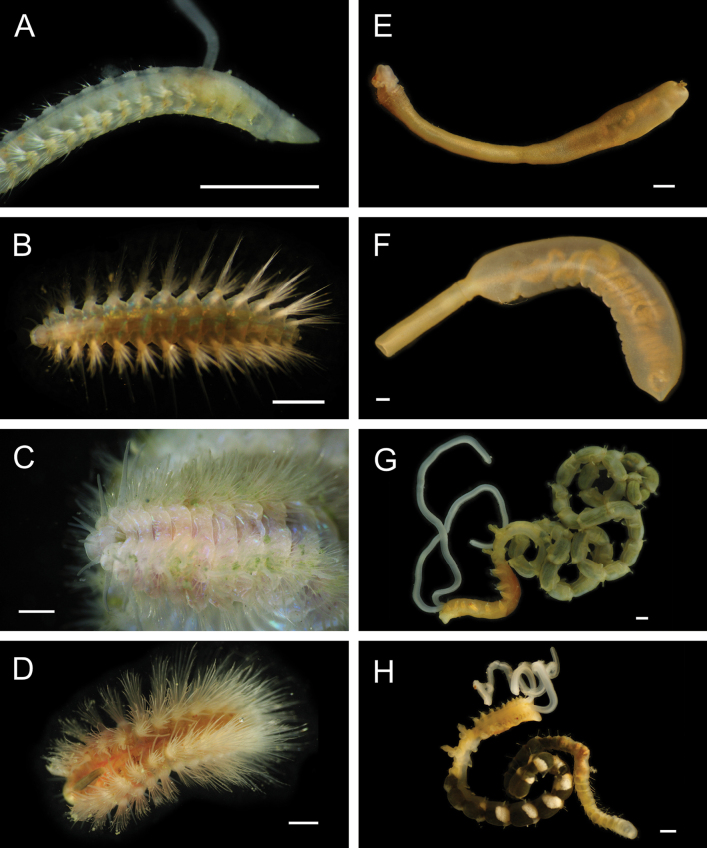
Annelida: *Cossura*, Amphinomidae, Sipuncula, and Chaetopteridae, representative live images **A***Cossura* stet. (A1388) **B**Amphinomidae stet. (A10107) **C**Amphinominae sp. SIO_BIC_A1379 (A1379) **D***Archinomelevinae* (A1398) **E**Sipuncula sp. SIO_BIC_A9803 (A9803) **F**Sipuncula sp. SIO_BIC_A9839 (A9839) **G***Phyllochaetopterus* sp. 6 sec. Moore et al. 2017 (A1540) **H***Phyllochaetopterus* sp. SIO_BIC_A8429 (A8429). Scale bars: 1 mm.

**Material examined.** AD4507: A1388; AD4988: A9922.

**Localities.** Mound 11 (~ 1005–1025 m), Parrita Scar (~ 1659–1663 m).

#### ﻿Annelida | Polychaeta | Amphinomida

**Amphinomidae stet.**

Fig. 25B

**Material examined.** S0219: A10107 (**PQ449269**).

**Localities.** Rio Bongo Scar (661 m).

**Remarks.** The COI sequence did not closely match any available GenBank reference sequences (<82% identity).

**Amphinominae sp. SIO_BIC_A1379**

Fig. 25C

**Material examined.** AD4506: A1379 (**PQ300673**).

**Localities.** Parrita Seep (1186 m).

**Remarks.** An undescribed genus and species. We thank Liz Borda (Texas A&M University San Antonio) for providing the COI sequence.

***Archinomelevinae* Borda, Kudenov, Chevaldonné, Blake, Desbruyères, Fabri, Hourdez, Pleijel, Shank, Wilson, Schulze & Rouse, 2013**

Fig. 25D

**Reference.**Borda et al. 2013**.

**Localities.** Mound 12 (~ 1000 m), Mound 11 (~ 1040 m; type locality), Parrita Seep (1402 m), Jacó Scar (~ 1800 m).

**Distribution.** Also known from vents in the Guaymas Basin, Gulf of California, ~ 2400 m (Borda et al. 2013).

#### ﻿Annelida | Polychaeta | Sipuncula

**Sipuncula sp. SIO_BIC_A9803**

Fig. 25E

**Material examined.** AD4978: A9803 (**PQ449329**).

**Localities.** Mound 12 (997 m).

**Remarks.** The closest COI BLASTN results on GenBank were an unidentified sipunculan from southern California (MK550656.1, 85.74% identity) and several golfingiids (~ 79–81% identity).

**Sipuncula sp. SIO_BIC_A9839**

Fig. 25F

**Material examined.** AD4987: A9839 (**PQ450416**).

**Localities.** Mound 12 (1012 m).

**Remarks.** Collected near a naturally occurring wood fall. The closest COI BLASTN results on GenBank were *Nephasomaabyssorum* (Koren & Danielssen, 1876) (JN865109.1, 82.14% identity) and several other golfingiids (~ 78–79% identity).

#### ﻿Annelida | Chaetopteriformia | Chaetopteridae

***Phyllochaetopterus* sp. 6 sec. Moore et al. 2017**

Fig. 25G

**Reference.**Moore et al. (2017) for DNA sequences and phylogenetic analysis of specimen A1540.

**Material examined.** AD4501: A1324 (**PQ449275**); AD4505: A1540 (KX896487); AD4508: A1394; S0217: A10085 (**PQ449263**).

**Localities.** Mound 11 (~ 1019–1025 m), Mound 12 (~ 984–997 m; this study), The Thumb (~ 940–1070 m; this study), Parrita Seep (1402 m; this study).

**Remarks.** An undescribed species.

***Phyllochaetopterus* sp. SIO_BIC_A8429**

Fig. 25H

**Material examined.** AD4922: A8429 (**PQ449310**); AD4988: A9916 (**PQ449335**), A9918 (**PQ449336**).

**Localities.** Mound 11 (1010 m), Mound 12 (1002 m).

**Remarks.** Associated with naturally occurring wood falls. Despite its sympatry with *Phyllochaetopterus* sp. 6 at Mound 12, this undescribed species is morphologically and genetically distinct, with a COI divergence of 14.89–15.41% from the previously published *P.* sp. 6 voucher SIO-BIC A1540 (KX896487.1). The closest COI BLASTN result on GenBank was an undescribed species of *Spiochaetopterus* from the East Pacific Rise (KX896501.1, voucher SIO-BIC A3619, 90.36–90.73% identity) (Moore et al. 2017). *Phyllochaetopterus* and *Spiochaetopterus* are not reciprocally monophyletic, and further phylogenetic investigation of their respective type species is required for revision of these genera (Moore et al. 2017).

### ﻿﻿Nemertea

We list higher-level taxonomy according to Strand et al. (2018).

#### ﻿Nemertea | Palaeonemertea | Tubulanidae

**Tubulanuscf.lutescens Cantell, 2001**

Fig. 26A

**Figure 26.**
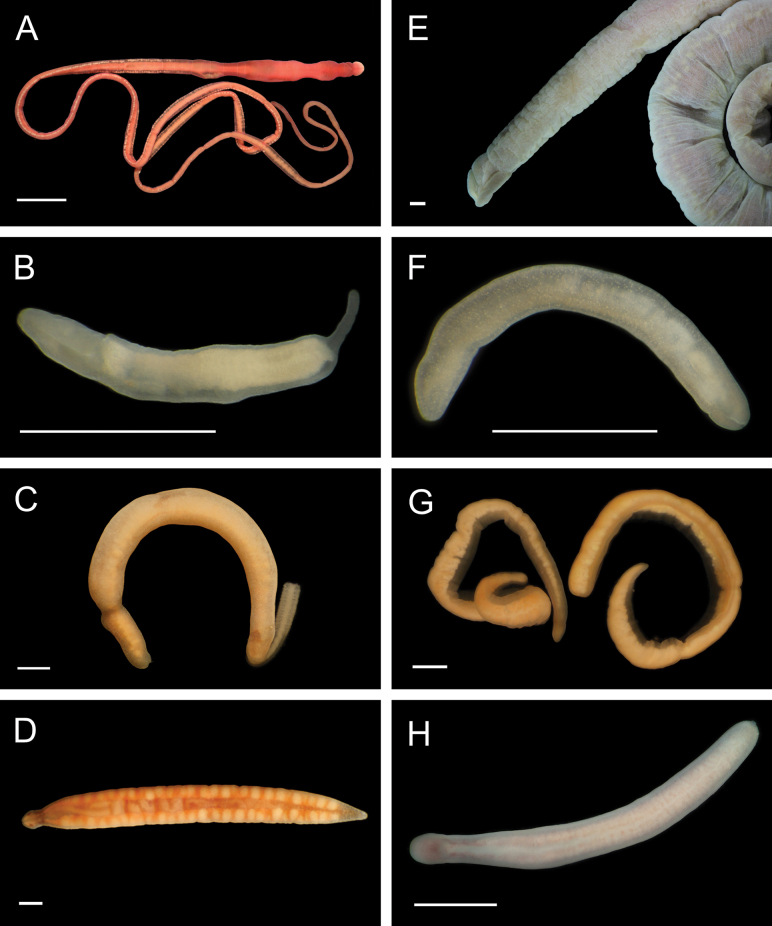
Nemertea, representative live images **A**Tubulanuscf.lutescens (N233) **B**Lineidae sp. SIO_BIC_N254 (N254) **C***Tetrastemmapolyakovae* (N258) **D***Tetrastemmasundbergi* (N256) **E**Eumonostilifera sp. SIO_BIC_N109 (N109) **F***Alvinonemerteschristianeae* (N253) **G***Alvinonemertesdariae* (N262) **H***Chernysheviaescarpiaphila* (N266). Scale bars: 1 cm (**A**); 1 mm (**B–H**).

**Reference.**Sagorny et al. 2022.

**Localities.** Mound 11 (~ 1019–1025 m), Mound 12 (~ 982–998 m).

**Remarks.** Despite disparate geography and internal and external morphological differences, the CRM specimens show only 1.3% COI divergence from *Tubulanuslutescens* Cantell, 2001, known from shallow waters off Sweden, warranting further examination (Sagorny et al. 2022).

#### ﻿Nemertea | Pilidiophora | Heteronemertea | Lineidae

**Lineidae sp. SIO_BIC_N254**

Fig. 26B

**Reference.**Sagorny et al. 2022.

**Localities.** Jacó Scar (1887 m).

**Remarks.** Associated with experimentally deployed wood. This single specimen (destroyed for DNA extraction) could not be attributed to a known genus and likely represents an undescribed species (Sagorny et al. 2022).

#### ﻿Nemertea | Hoplonemertea | Monostilifera | Amphiporina

***Tetrastemmapolyakovae* Sagorny, von Döhren, Rouse & Tilic, 2022**

Fig. 26C

**Reference.**Sagorny et al. 2022**.

**Localities.** Mound 12 (~ 996–999 m; type locality).

**Distribution.** Known only from the CRM seeps.

***Tetrastemmastrandae* Sagorny, von Döhren, Rouse & Tilic, 2022**

**Reference.**Sagorny et al. 2022** (histological sections published; no live images captured).

**Localities.** Jacó Scar (1885 m; type locality).

**Distribution.** Known only from the CRM seeps.

***Tetrastemmasundbergi* Sagorny, von Döhren, Rouse & Tilic, 2022**

Fig. 26D

**Reference.**Sagorny et al. 2022**.

**Localities.** Mound 12 (~ 996–999 m; type locality).

**Distribution.** Known only from the CRM seeps.

#### ﻿Nemertea | Hoplonemertea | Monostilifera | Eumonostilifera

**Eumonostilifera sp. SIO_BIC_N109**

Fig. 26E

**Reference.**Sagorny et al. 2022.

**Localities.** Parrita Seep (~ 1401–1419 m).

**Remarks.** An undescribed species represented by a single specimen (Sagorny et al. 2022).

#### ﻿Nemertea | Hoplonemertea | Monostilifera | Oerstediina

***Alvinonemerteschristianeae* Sagorny, von Döhren, Rouse & Tilic, 2022**

Fig. 26F

**Reference.**Sagorny et al. 2022**.

**Localities.** Jacó Scar (1887 m; type locality).

**Distribution.** Also reported from the non-seep seamount Quepos Plateau, 67 km south of Jacó Scar, at 2184 m depth.

**Remarks.** Associated with experimentally deployed or naturally occurring wood (Sagorny et al. 2022).

***Alvinonemertesdariae* Sagorny, von Döhren, Rouse & Tilic, 2022**

Fig. 26G

**Reference.**Sagorny et al. 2022**.

**Localities.** Parrita Seep (1407 m; type locality).

**Distribution.** Known only from the CRM seeps.

**Remarks.** Associated with *Paragorgia* stet. (Co3054) (Sagorny et al. 2022).

***Chernysheviaescarpiaphila* Sagorny, von Döhren, Rouse & Tilic, 2022**

Fig. 26H

**Reference.**Sagorny et al. 2022**.

**Localities.** Jacó Scar (~ 974–1856 m), Mound Jaguar (1909 m; type locality).

**Distribution.** Known only from the CRM seeps.

**Remarks.** Associated with the exterior surfaces and tubes of *Escarpiaspicata* (Sagorny et al. 2022).

### ﻿﻿Brachiopoda

***Platidiaanomioides* (Scacchi & Philippi in Philippi, 1844) sp. inc.**

Fig. 27A, B

**Figure 27.**
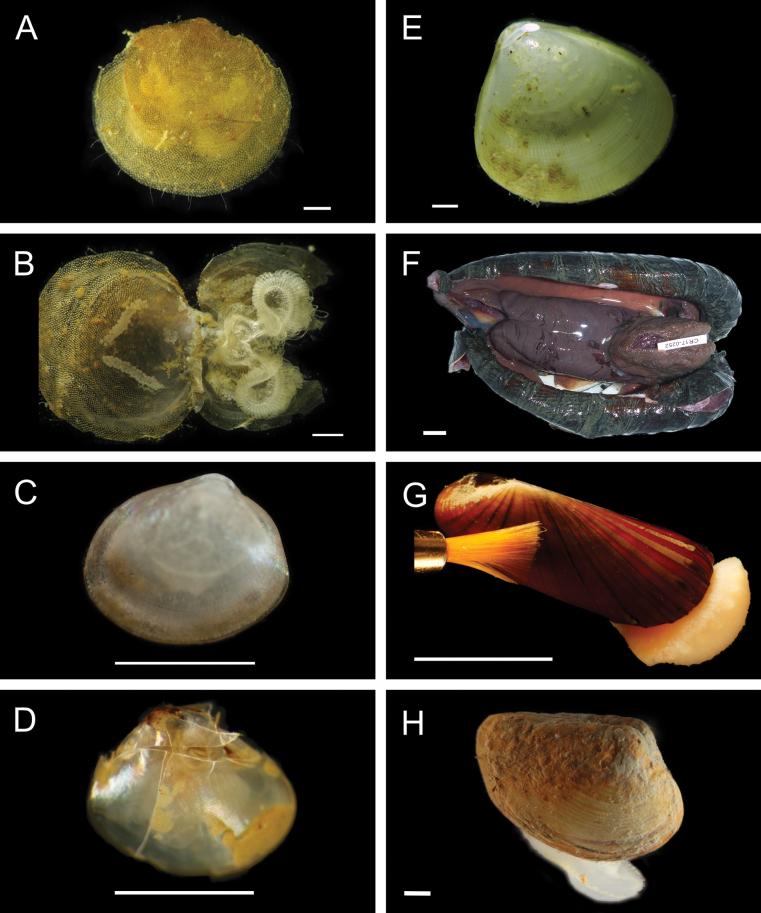
Brachiopoda and Mollusca: Bivalvia: Nuculidae, Solemyidae, and Nuculanidae, representative live images **A***Platidiaanomioides* sp. inc. (B211, exterior view) **B***Platidiaanomioides* sp. inc. (B211, interior view) **C***Ennuculacolombiana* (M17879) **D***Ennucula* stet. (M16966) **E***Nuculachrysocoma* (M11989) **F**Acharaxcf.johnsoni (M15768, adult specimen, opened) **G**Acharaxcf.johnsoni (M17077, young specimen) **H**Nuculanacf.callimene (M17063). Scale bars: 1 mm (**A–E, H**); 1 cm (**F, G**).

**Material examined.** AD4512: B211; AD4979: M16876.

**Localities.** Quepos Slide (~ 380–395 m).

**Remarks.** We thank Sandra Carlson (University of California Davis) for the identification of these specimens as *Platidia*, most likely *P.anomioides*, which is considered cosmopolitan at depths ﻿18–2190 m (Foster 1989; Santagata and Tunnell 2009). More definitive identification requires dissolving soft tissues for analysis of the cardinalia and spicules. At least one specimen contains eggs in the mantle canals (Fig. 27B).

### ﻿﻿Mollusca

We list the major clades according to the phylogenetic relationships in Smith et al. (2011).

#### ﻿Mollusca | Bivalvia

We list entries following the taxonomic arrangement in Valentich-Scott et al. (2020).

#### ﻿Mollusca | Bivalvia | Protobranchia | Nuculida | Nuculidae

***Ennuculacolombiana* (Dall, 1908)**

Fig. 27C

**Material examined.** AD4588: M17879.

**Localities.** Mound 12 (997 m).

**Distribution.** Originally described from the Gulf of Panama, 54 m, and known from the Gulf of California to Peru, 11–734 m (Coan and Valentich-Scott 2012).

**New records.** The CRM specimen represents a new maximum depth record for this species.

***Ennucula* stet.**

Fig. 27D

**Material examined.** AD4988: M16966 (**PQ449418**).

**Localities.** Mound 11 (1010 m).

**Remarks.** This damaged juvenile specimen was associated with a naturally occurring wood fall. The closest COI BLASTN results on GenBank were nuculids: *Ennuculacumingii* (Hinds, 1843) (KC984750.1, 84.89% identity), *E.tenuis* (Montagu, 1808) from Japan (LC144804.1, 83.23% identity), *E.niponica* (E. A. Smith, 1885) from Japan (LC144803.1, 83.10% identity), and *Acilamirabilis* (A. Adams & Reeve, 1850) from Japan (LC144802.1, 83.36% identity).

***Nuculachrysocoma* Dall, 1908**

Fig. 27E

**Material examined.** AD4503: M11989.

**Localities.** Mound 12 (~ 965–995 m).

**Distribution.** Originally described from several stations off Peru, Ecuador, and southern Mexico, 734–4064 m (Dall, 1908), and known from Cascadia Abyssal Plain, Oregon, to Coquimbo, central Chile, 734–4134 m (Valentich-Scott et al. 2020).

#### ﻿Mollusca | Bivalvia | Protobranchia | Solemyida | Solemyidae

**Acharaxcf.johnsoni (Dall, 1891)**

Fig. 27F, G

**References.**Neulinger et al. (2006) for DNA sequences and phylogenetic analysis; occurrences at Mound 11 and Mound 12 (Aguado and Rouse 2011; Levin et al. 2015; Rouse et al. 2018).

**Additional material examined.** AD4503: M11980; AD4505: M12003; AD4507: M12012 (juvenile); AD4511: M12054, M16239; AD4513: M12072; AD4910: M15768 (18S: **PQ304648**), M15769; S0217: M17062; S0220: M17077 (18S: **PQ304649**; juvenile).

**Localities.** Mound 12 (~ 1000 m), Mound 11 (~ 1020 m), The Thumb (1069 m; this study), Parrita Scar (~ 1659–1663 m; this study), Jacó Scar (1744 m; this study), Subduction Plume (3410 m; this study). The collection locality in Neulinger et al. (2006) (9°10.4'N, 084°48.2'W, 763 m) matches the site referenced in this work as Jacó Summit.

**Remarks.** Originally described from 1838 m off Baja California (Dall 1891), *A.johnsoni* has been reported along the eastern Pacific margin from Alaska to Peru, as well as in the northwestern Pacific, 100–5379 m (Coan and Valentich-Scott 2012). According to 18S phylogenies, at least two clades of deep-sea *Acharax*, morphologically similar to *A.johnsoni*, occur in the Pacific and warrant consideration as separate species (Neulinger et al. 2006; Fukasawa et al. 2017). In the absence of genetic data from the type locality (only empty shells have been recovered from recent expeditions to the region (Hendrickx et al. 2016; Suárez-Mozo et al. 2019)), we report the CRM specimens as A.cf.johnsoni.

The two 18S sequences in this study matched different previously reported clades, suggesting the presence of at least two cryptic species at the CRM, potentially segregated by depth. M17007 from Subduction Plume (3410 m) showed 99.94% identity to the Jacó Summit specimen (AJ563763.1) in Neulinger et al. (2006). M17007 also grouped with the “*Acharax* 1” clade of (Fukasawa et al. 2017), showing 99.61–99.94% identity to sequences from the Aleutian Trench (AJ563760.1), Java Trench (AJ563756.1, AJ563757.1), Chishima Trench seeps (LC186962.1), Japan Trench seeps (LC186965.1, LC186966.1), Hine Hina vents in the Lau Basin (LC186970.1), and Haima seeps (OQ836650.1). M15768 from Mound 12 (~ 1000 m) grouped with the “*Acharax* 2” clade of Fukasawa et al. (2017), showing 99.14–99.77% identity to sequences from seeps off Oregon (AJ563751.1, AJ563752.1, AJ563753.1, AJ563754.1, AJ563755.1), off Peru (AJ563762.1), and at the Nankai Trough (LC186957.1). The “*Acharax* 1” and “*Acharax* 2” clades show the same depth range globally (765–5300 m and 780–5300 m, respectively) (Fukasawa et al. 2017), so further sequencing and sampling are needed to assess depth stratification of *Acharax* at the CRM. A detailed comparison of these clades may also illuminate distinguishing morphological features.

CRM specimens from Mound 11 and Mound 12 are hosts of copepods (see Cyclopoida sp. SIO_BIC_C12780), the chrysopetalid *Natsushimasashai* (Aguado and Rouse 2011), and the hesionid *Neogyptisjeffruoccoi* (Rouse et al. 2018).

#### ﻿Mollusca | Bivalvia | Protobranchia | Nuculanida | Nuculanidae

**Nuculanacf.callimene (Dall, 1908)**

Fig. 27H

**Material examined.** S0220: M17063.

**Localities.** Subduction Plume (3434 m).

**Remarks.** This specimen is morphologically similar to *N.callimene* except for the posterior end and may represent an undescribed species. *N.callimene* is known from western Baja California to the Pacific margin of Panama (type locality), 183–3200 m (Coan and Valentich-Scott 2012; Hendrickx et al. 2016).

**Nuculanacf.hamata (Carpenter, 1864)**

Fig. 28A

**Figure 28.**
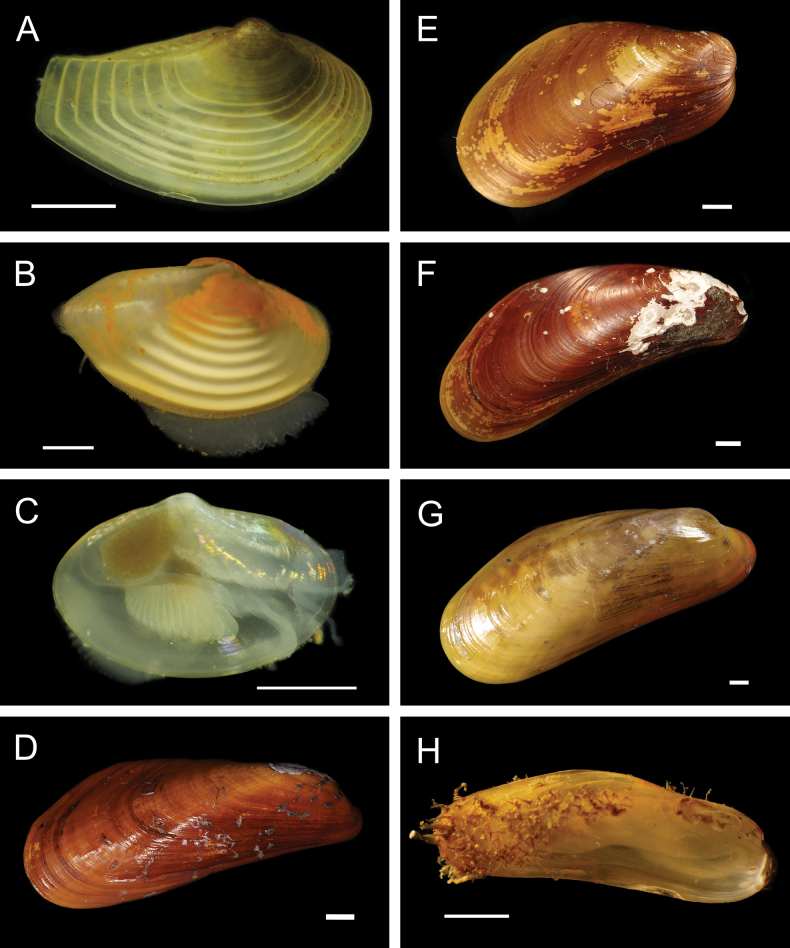
Mollusca: Bivalvia: Nuculanidae, Bathyspinulidae, Yoldiidae, and Mytilidae, representative live images **A**Nuculanacf.hamata (M12047) **B***Tindariopsisgrasslei* (M16806) **C***Yoldiella* stet. (M12046) **D***Bathymodiolusbillschneideri* (M12074) **E***Bathymodiolusearlougheri* (M14479) **F***Bathymodiolusnancyschneiderae* (M14531) **G***Bathymodiolusthermophilus* (M17023) **H***Idas* stet. (M17069). Scale bars: 1 mm (**A–C, H**); 1 cm (**D–G**).

**Material examined.** AD4510: M12047.

**Localities.** Jacó Summit (742 m).

**Remarks.** This specimen resembles *N.hamata*, which is known from Alaska to the Gulf of California (type locality: Catalina Island, southern California), 20–1100 m, and may include cryptic species; records of this morphologically variable species from further south are considered suspect and warrant further investigation (Coan and Valentich-Scott 2012; Hendrickx et al. 2016). Genetic comparison to N.cf.hamata recorded from Baja California, 750–850 m (Hendrickx et al. 2016), may be informative.

#### ﻿Mollusca | Bivalvia | Protobranchia | Nuculanida | Bathyspinulidae

***Tindariopsisgrasslei* (Allen, 1993)**

Fig. 28B

**Material examined.** AD4503: M11985 (dead shell only); AD4506: M12005; AD4590: M12141; AD4971: M16735, M16736; AD4973: M16749, M16750; AD4977: M16806; AD4987: M16902; AD4985: M16908; AD4988: M16964; S0217: M17042.

**Localities.** Mound 12 (990 m, dead shell; 992–1010 m, live specimens), Mound 11 (1009 m), Parrita Seep (~ 1030–1179 m), The Thumb (1069 m), Jacó Scar (1783–1817 m).

**Distribution.** Known from the Guaymas Basin, Gulf of California (type locality, 2003 m), to the Costa Rica Subduction Zone, 1400–2012 m (Coan and Valentich-Scott 2012).

**New records.**CRM specimen M16908 from 992 m represents a new minimum depth for this species.

**Remarks.** This species is placed “with reluctance” in *Tindariopsis* (Coan and Valentich-Scott 2012). Preliminary molecular work suggests a position closer to *Malletia*. M11985 was associated with a naturally occurring wood fall.

#### ﻿Mollusca | Bivalvia | Protobranchia | Nuculanida | Yoldiidae

***Yoldiella* stet.**

Fig. 28C

**Material examined.** AD4510: M12046.

**Localities.** Jacó Summit (742 m).

#### ﻿Mollusca | Bivalvia | Pteriomorphia | Mytilida | Mytilidae

***Bathymodiolusbillschneideri* McCowin, Feehery & Rouse, 2020**

Fig. 28D

**Reference.**McCowin et al. 2020**.

**Localities.** Parrita Seep (~ 1400 m), Jacó Scar (~ 1750–1900 m; type locality).

**Distribution.** Known only from the CRM seeps and apparently found no shallower than ~ 1400 m.

**Remarks.***B.billschneideri* is a host of the scaleworms *Branchipolynoeeliseae*, *Br.halliseyae*, *Br.kajsae*, and *Br.meridae* (Lindgren et al. 2019). Previous reports of mytilid mussels at Parrita Seep (previously published as “Quepos Seep”) (Sahling et al. 2008) and brown-colored *Bathymodiolus* mussels at Jacó Scar (Levin et al. 2012, 2015) are now known to correspond to *B.billschneideri*.

***Bathymodiolusearlougheri* McCowin, Feehery & Rouse, 2020**

Fig. 28E

**Reference.**McCowin et al. 2020**.

**Localities.** Mound 12 (~ 1000 m), The Thumb (1073 m), Jacó Scar (~ 1750–1900 m; type locality).

**Distribution.** Known only from the CRM seeps.

**Remarks.***B.earlougheri* likely also occurs at sites of intermediate depth (e.g., Parrita Seep, ~ 1400 m) but has not been collected there, likely due to limited sampling (McCowin et al. 2020). *B.earlougheri* is the only known host of the chrysopetalid *Laubierusalvini* (Aguado and Rouse 2011). It is also a host of the scaleworms *Branchipolynoehalliseyae*, *Br.kajsae*, *Br.meridae*, and possibly *Br.eliseae* (Lindgren et al. 2019). Previous reports of golden-colored *Bathymodiolus* mussels at multiple CRM seep sites (Levin et al. 2012, 2015) are now known to correspond to *B.earlougheri*.

***Bathymodiolusnancyschneiderae* McCowin, Feehery & Rouse, 2020**

Fig. 28F

**Reference.**McCowin et al. 2020**.

**Localities.** Mound 12 (~ 1000 m), Jacó Slope (1063 m; type locality), The Thumb (1073 m).

**Distribution.** Known only from the CRM seeps and apparently found no deeper than ~ 1000 m.

**Remarks.***B.nancyschneiderae* is a host of the scaleworms *Branchipolynoeeliseae*, *Br.halliseyae*, *Br.kajsae*, and possibly also *Br.meridae* (Lindgren et al. 2019). Previous reports of brown-colored *Bathymodiolus* spp. at Mound 11 and Mound 12 (Levin et al. 2015) are now known to correspond to *B.nancyschneiderae*.

***Bathymodiolusthermophilus* Kenk & B. R. Wilson, 1985**

Fig. 28G

**References.**Levin et al. 2012; McCowin et al. 2020.

**Localities.** Jacó Scar (1794–1814 m).

**Distribution.** Originally described from 2495 m at the Galápagos Rift (Kenk and Wilson 1985) and distributed along the East Pacific Rise between 13°N and 21°S latitude (Johnson et al. 2013), *B.thermophilus* was previously thought to be restricted to hydrothermal vents but also occurs at the Jacó Scar “hydrothermal seep” site (Levin et al. 2012; McCowin et al. 2020).

**Remarks.***B.thermophilus* specimens from the CRM seeps were not observed to contain *Branchipolynoe* spp. scaleworms, but this apparent absence may reflect limited sample size and the tendency of the worms to evacuate the mussels upon disturbance.

***Idas* stet.**

Fig. 28H

**Reference.**Xu et al. (2017) for phylogenetic analysis.

**Material examined.** AD4587: M13006 (KU975037); AD4907: M16100; S0219: M17069 (**PQ450399**).

**Localities.** Rio Bongo Scar (661 m; this study), Mound 12 (996–999 m).

**Remarks.** Associated with naturally occurring and experimentally deployed wood. As described in Xu et al. (2017), M13006 shows morphological similarity to *Idaswashingtonius* (Bernard, 1978), which occurs in the eastern Pacific from Washington to the Guaymas Basin and in the western Pacific, 1240–2200 m (Coan et al. 2000), but genetically it is more closely related to *I.macdonaldi* Gustafson, Turner, Lutz & Vrijenhoek, 1998, which occurs at hydrocarbon seeps in the Gulf of Mexico (Gustafson et al. 1998). The paraphyly of *Idas* (Lorion et al. 2013; Xu et al. 2017) warrants further investigation.

#### ﻿Mollusca | Bivalvia | Pteriomorphia | Pectinida | Pectinidae

***Delectopectenvancouverensis* (Whiteaves, 1893)**

Fig. 29A

**Figure 29.**
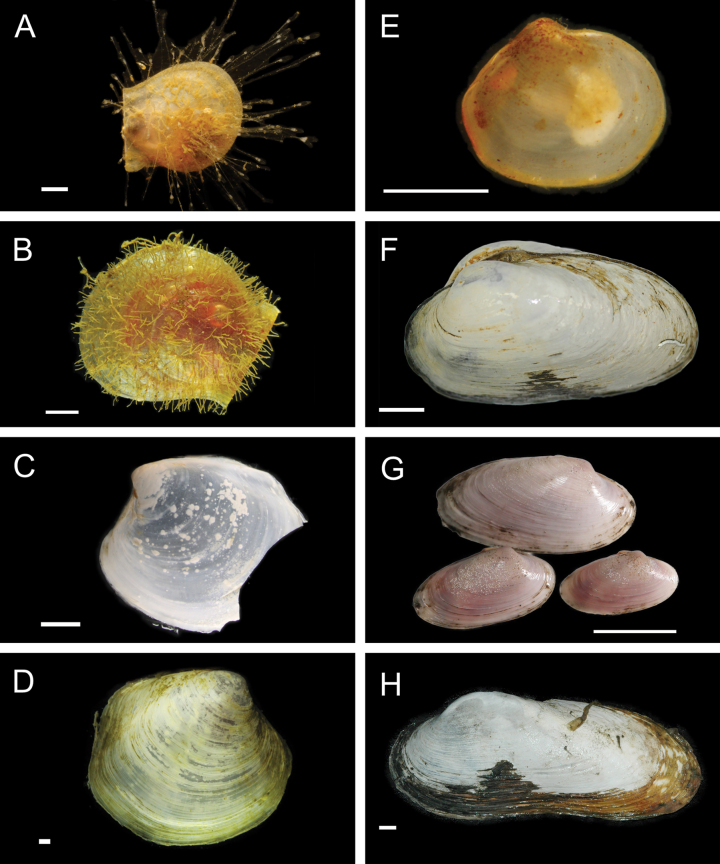
Mollusca: Bivalvia: Pectinidae, Thyasiridae, Galeommatidae, and Vesicomyidae, representative live images **A***Delectopectenvancouverensis* (M16184) **B***Delectopectenzacae* (M16188) **C***Thyasiramethanophila* (M17098, valve only) **D***Thyasira* stet. (M12059) **E**Axinodoncf.redondoensis (M16845) **F***Archivesicagigas* (M12011) **G***Archivesica* sp. 6 sec. Audzijonyte et al. 2012 (M16141) **H**Archivesica sp. SIO_BIC_M12070 aff. gigas (M12070). Scale bars: 1 mm (**A–E**); 1 cm (**F–H**).

**Material examined.** AD4512: M12063; AD4918: M16184.

**Localities.** Quepos Slide (338–~ 400 m).

**Distribution.** Originally described from British Columbia, Canada; recorded in the eastern Pacific from Alaska to Baja California as well as the Bering Sea and northwestern Pacific to the Sea of Japan, 20–4100 m (Coan and Valentich-Scott 2012; Kamenev 2013; Hendrickx et al. 2016). GBIF includes a museum record from the Galápagos Islands, 466 m (Blum and Fong 2016c).

**New records.** Pending verification of the GBIF record, the CRM specimens represent new southern records for this species.

**Remarks.** M16184 was associated with epibiotic hydroids. DNA sequences could not be obtained.

***Delectopectenzacae* (Hertlein, 1935)**

Fig. 29B

**Material examined.** AD4921: M16188.

**Localities.** Quepos Slide (~ 345–394 m).

**Distribution.** Known from Baja California (type locality: Cabo San Lucas, 37–402 m) (Hertlein 1935), to Peru and the Galápagos Islands, 10–1840 m (Coan and Valentich-Scott 2012).

**Remarks.** DNA sequences could not be obtained.

#### ﻿Mollusca | Bivalvia | Heterodonta | Lucinida | Thyasiridae

***Thyasiramethanophila* P. G. Oliver & Sellanes, 2005**

Fig. 29C

**Reference.**Coan and Valentich-Scott (2012), in which the locality of specimen SBMNH 350533 (9.033, -84.621; 1408 m; no DNA sequences; Santa Barbara Museum of Natural History) aligns with the locality known in this work as Parrita Seep.

**Additional material examined.** S0230: M17098 (juvenile specimen, only valves collected).

**Localities.** Parrita Seep (1408 m); Mound Jaguar (1909 m; this study, valves).

**Distribution.** Also known from seeps at the type locality off Concepción, central Chile, 780 m (Oliver and Sellanes 2005). Confirmation of live specimens at Mound Jaguar would establish a new depth record.

***Thyasira* stet.**

Fig. 29D

**Material examined.** AD4512: M12059 (**PQ450412**); AD4978: M16840; AD4988: M16977.

**Localities.** Quepos Slide (~ 344–411 m), Mound 12 (~ 996–999 m), Jacó Scar (1783 m).

**Remarks.** This morphospecies warrants comparison to other eastern Pacific specimens that have been dubiously reported as *Thyasiraflexuosa* (Montagu, 1803) (type locality: Great Britain) and likely represent several cryptic species (Coan et al. 2000; Coan and Valentich-Scott 2012). For M12059, the closest COI BLASTN results on GenBank were *Thyasirasarsii* (R. A. Philippi, 1845) from Norway (AM706508.1 and AM706509.1, both 97.09% identity). Based on a recent phylogeny (Fukasawa et al. 2017), *Thyasira* appears to require taxonomic revision.

#### ﻿Mollusca | Bivalvia | Heterodonta | Galeommatida | Galeommatidae

**Axinodoncf.redondoensis (T. A. Burch, 1941)**

Fig. 29E

**Material examined.** AD4978: M16845.

**Localities.** Mound 12 (~ 996–999 m).

**Remarks.** Likely an undescribed species. Possibly a range and depth extension of *Axinodonredondoensis*, presently known from Washington to Redondo Beach, southern California (type locality, 137 m), 120–330 m (Harry 1969; Valentich-Scott 1998; Coan et al. 2000).

#### ﻿Mollusca | Bivalvia | Heterodonta | Venerida | Vesicomyidae

We thank Elena Krylova (P.P. Shirshov Institute of Oceanology, Russian Academy of Sciences) for input on this section.

***Archivesicagigas* (Dall, 1896)**

Fig. 29F

**References.**Audzijonyte et al. 2012; Levin et al. 2012.

**Additional material examined.** AD4506: M12007; AD4507: M12011 (**PQ449002**); AD4508: M12014 (**PQ449401**).

**Localities.** Parrita Seep (1186 m, ~ 1400 m; this study), Parrita Scar (~ 1659–1667 m; this study), Jacó Scar (~ 1800 m); an unnamed locality ~ 85 km northwest of Mound Jaguar (10.3000, -86.3053; 1531 m) (Audzijonyte et al. 2012).

**Distribution.** Originally described from the Guaymas Basin, Gulf of California, 1567 m (Dall 1896) and reported as far north as the Gulf of Alaska (Coan and Valentich-Scott 2012). Genetically confirmed records are known from seeps, sedimented vents, and whale falls from the Oregon Subduction Zone to Costa Rica, 1013–2028 m, as well as a seep at 1200 m in Hiroo Submarine Canyon off Hokkaido (Audzijonyte et al. 2012).

***Archivesica* sp. 6 sec. Audzijonyte et al. 2012**

Fig. 29G

**Reference.**Audzijonyte et al. (2012) for DNA sequences and phylogenetic analysis.

**Additional material examined.** AD4590: M13509 (**PQ450381**, **PQ450398**; tissues); AD4912: M16141 (**PQ449413**).

**Localities.** Jacó Scar (1677 m; ~ 1791–1842 m, this study).

**Distribution.** Known only from the CRM seeps.

**Remarks.** Morphologically resembles *A.gigas* and may warrant description as a new species (Audzijonyte et al. 2012).

***Archivesica* sp. 7 sec. Audzijonyte et al. 2012**

**References.**Peek et al. (2000) and Audzijonyte et al. (2012) for DNA sequences and phylogenetic analysis. Not collected in this study.

**Localities.** An unnamed seep at 3002 m (9.69850, -86.06817) and a “low-temperature vent” at 3096 m (9.7112, -86.0777) (Audzijonyte et al. 2012), both ~ 20 km west of Mound Jaguar.

**Distribution.** Also known from a seep at 4300 m in the Middle America Trench off Mexico (Audzijonyte et al. 2012; Decker et al. 2012) and from the Peru margin, 3711–4724 m (Audzijonyte et al. 2012).

**Remarks.** This taxon warrants consideration as a new species based on COI sequences, but no morphological voucher specimens are available (Audzijonyte et al. 2012).

**Archivesica sp. SIO_BIC_M12070 aff. gigas (Dall, 1896)**

Fig. 29H

**Material examined.** AD4513: M12070 (**PQ450402**).

**Localities.** Jacó Scar (1744 m).

**Remarks.** An undescribed species. The COI sequence of this specimen was 94.83–95.20% identical to sequences of *A.gigas* from northern Japan (PP899629.1), the Guaymas Basin, Gulf of California (MF959623.1 (Liu et al. 2018), MT947383.1), and off northern California (MT947382.1).

***Calyptogenacostaricana* Krylova & Sahling, 2006**

**Reference.**Krylova and Sahling 2006**. Not collected in this study.

**Localities.** Mound 10 (2258–2263 m; type locality).

**Distribution.** Also known from seeps in Monterey Canyon, off California, 2193–2219 m, and a vent on the Peru margin, 2500 m (Audzijonyte et al. 2012).

***Calyptogenadiagonalis* Barry & Kochevar, 1999**

Fig. 30A

**Figure 30.**
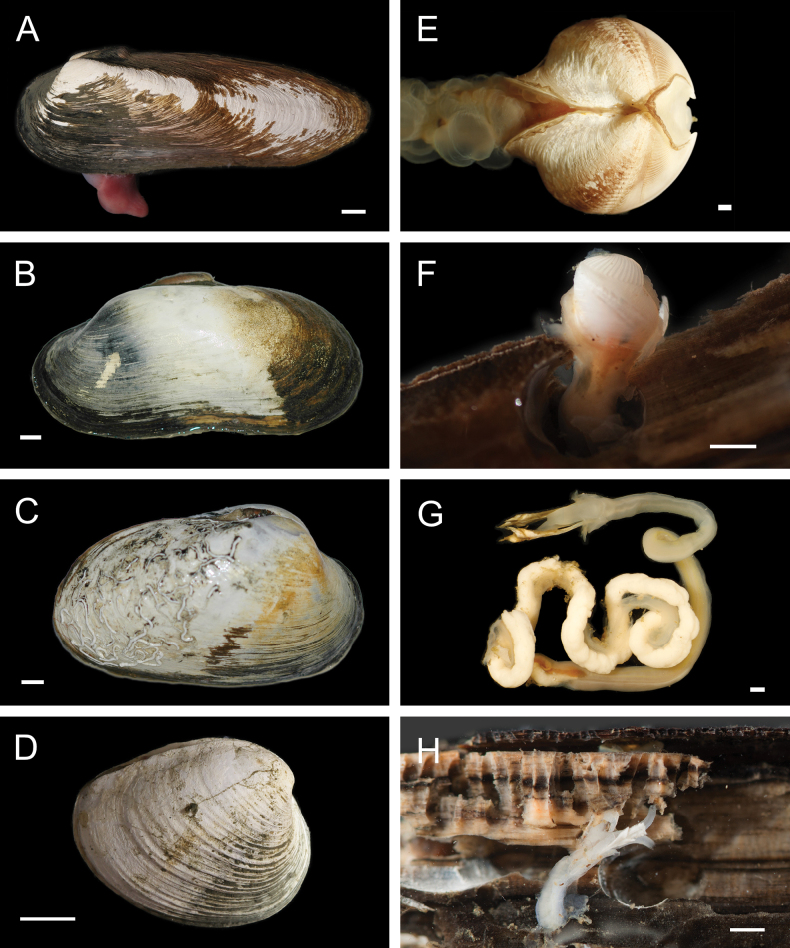
Mollusca: Bivalvia: Vesicomyidae, Xylophagaidae, and Teredinidae, representative live images **A***Calyptogenadiagonalis* (M17064) **B***Phreagenasoyoae* (M11999) **C***Phreagena* sp. 5 sec. Audzijonyte et al. 2012 (M12009) **D***Pliocardiakrylovata* (M17026) **E***Xylophaga* stet. (M16099) **F***Xyloredo* gen. inc. (M17870) **G**Teredinidae sp. SIO_BIC_M17100 (M17100) **H**Teredinidae stet. (M12290). Scale bars: 1 cm (**A–D**); 1 mm (**E–H**).

**References.**Barry and Kochevar 1999**; Audzijonyte et al. 2012.

**Additional material examined.** S0220: M17064, M17065.

**Localities.** Subduction Plume (3408 m; this study); an unnamed seep (9°42.28'N, 86°4.38'W; 2980–3800 m; type locality) (Barry and Kochevar 1999) and a “low-temperature vent” (9.7112, -86.0777; 3096 m) (Audzijonyte et al. 2012), both ~ 20 km west of Mound Jaguar.

**Distribution.** Also known from seeps at the Oregon margin, 2021–2089 m (Barry and Kochevar 1999; Audzijonyte et al. 2012; Coan and Valentich-Scott 2012), vents at the Juan de Fuca Ridge, 2400–2437 m (Peek et al. 1997; Audzijonyte et al. 2012), and the Peru margin, 2290–3070 m (Audzijonyte et al. 2012).

**Remarks.** M17065 was associated with an anemone, Co3084. Based on morphological and genetic similarities, Audzijonyte et al. (2012) recommend synonymization of this species (referenced as *Archivesicadiagonalis*) with the western Pacific nominal species *A.magnocultellus* (Okutani, Kojima & Iwasaki, 2002), pending examination of the type material of *A.magnocultellus*. Formal synonymization would extend the species’ range to seeps off southern Japan, 1900–2500 m (Audzijonyte et al. 2012).

***Calyptogena* sp. 3 sec. Audzijonyte et al. 2012**

**Reference.**Audzijonyte et al. (2012) for DNA sequences and phylogenetic analysis. Not collected in this study.

**Localities.** Unnamed seep ~ 25 km west of Mound Jaguar (9.6678, -86.1193; 3560 m) (Audzijonyte et al. 2012).

**Distribution.** Also known from seep and whale fall habitats off central California, 2895–3449 m (Audzijonyte et al. 2012).

**Remarks.** This taxon warrants consideration as a new species based on COI sequences, but no morphological voucher specimens are available (Audzijonyte et al. 2012).

***Calyptogena* sp. mt-V sec. Goffredi et al. 2003**

**References.**Goffredi et al. (2003) and Audzijonyte et al. (2012) for DNA sequences and phylogenetic analysis. Not collected in this study.

**Localities.** Unnamed “low-temperature vent” ~ 20 km west of Mound Jaguar (9.7112, -86.0777; 3096 m) (Audzijonyte et al. 2012).

**Remarks.** The single genetic sample of this taxon was originally identified as *Calyptogenapacifica* (Peek et al. 2000) and underwent several nomenclatural revisions upon further phylogenetic analyses (Goffredi et al. 2003; Audzijonyte et al. 2012). This taxon warrants consideration as a new species based on COI sequences, but no morphological voucher specimens are available (Audzijonyte et al. 2012).

***Phreagenaextenta* (Krylova & Moskalev, 1996)**

**Reference.**Peek et al. (2000) for DNA sequences and phylogenetic analysis. Not collected in this study.

**Localities.** Unnamed seep ~ 20 km west of Mound Jaguar (9.6983, -86.0682; 3002 m).

**Distribution.** Originally described from a seep in Monterey Bay at 3041 m; recorded from the Gulf of Alaska to Costa Rica, 2889–4445 m (Peek et al. 2000; Audzijonyte et al. 2012; Coan and Valentich-Scott 2012), as well as the Kuril Trench, 3512 m (Okutani et al. 2009; Audzijonyte et al. 2012).

**Remarks.** Previously placed in *Ectenagena* and then *Calyptogena* (Coan and Valentich-Scott 2012), this species is provisionally, and with an acknowledgment of doubt, allocated to *Phreagena* (Johnson et al. 2017).

***Phreagenasoyoae* (Okutani, 1957)**

Fig. 30B

**Reference.**Audzijonyte et al. (2012) for DNA sequences and phylogenetic analysis.

**Additional material examined.** AD4505: M11999 (**PQ449397**, **PQ449398**, **PQ449399**, **PQ450407**, **PQ450408**).

**Localities.** Mound 11 (~ 1019-1025 m; this study); Mound Carablanca, Nicaragua margin (1432 m).

**Distribution.** Originally described from seeps off Japan, 750–1500 m (type locality: Sagami Bay, 750 m), *P.soyoae* is found at seep, vent, and whale fall habitats from the Juan de Fuca Ridge to the Costa Rica and Nicaragua margins, 519–2400 m (Audzijonyte et al. 2012).

***Phreagena* sp. 5 sec. Audzijonyte et al. 2012**

Fig. 30C

**Reference.**Audzijonyte et al. (2012) for DNA sequences and phylogenetic analysis.

**Additional material examined.** AD4506: M12009 (**PQ449400**), M12085 (**PQ450380**); AD4508: M12088 (**PQ450379**); AD4988: M16960 (**PQ449417**).

**Localities.** Mound 11 (1012 m; this study), Jacó Slope (1024 m), Parrita Seep (1186 m and ~ 1400 m, this study; 1408 m). The locality “Mound Quepos” at 1408 m and the upslope region of Jacó Scar at 1024 m in Audzijonyte et al. (2012) are reported as “Parrita Seep” and “Jacó Slope,” respectively, in this work.

**Remarks.** Morphologically resembles *Phreagenasoyoae* and may warrant description as a new species (Audzijonyte et al. 2012).

***Pliocardiakrylovata* A. M. Martin & Goffredi, 2012**

Fig. 30D

**Reference.**Martin and Goffredi 2012**.

**Additional material examined.** S0213: M17026.

**Localities.** Jacó Summit (~ 741–744 m; type locality), Mound 12 (~ 967–995 m).

**Distribution.** Known only from the CRM seeps.

**Remarks.** Found in sediments near microbial mats and typically positioned with approximately half the shell length above the sediment, forming “a spatial bridge between the oxic overlying water and the sulphide-rich sediment” (Martin and Goffredi 2012). Assignment of this species to the genus *Pliocardia* was tentative based on uncertainty within Pliocardiinae (Martin and Goffredi 2012), and the clade containing this taxon was later suggested to warrant a new genus (Johnson et al. 2017).

#### ﻿Mollusca | Bivalvia | Heterodonta | Myida | Pholadoidea | Xylophagaidae

***Xylophaga* stet.**

Fig. 30E

**Material examined.** AD4906: M15767, M16099, M16111.

**Localities.** Mound 12 (1002 m).

**Remarks.** Associated with experimentally deployed wood. We thank Chiara Romano (University of Gastronomic Sciences) for this identification.

***Xyloredo* gen. inc.**

Fig. 30F

**Material examined.** AD4588: M17870.

**Localities.** Mound 12 (~ 1000 m).

**Remarks.** Associated with a naturally occurring palm wood fall.

#### ﻿Mollusca | Bivalvia | Heterodonta | Myida | Pholadoidea | Teredinidae

We thank Reuben Shipway (University of Plymouth) for these identifications.

**Teredinidae sp. SIO_BIC_M17100**

Fig. 30G

**Material examined.** AD4913: M16147; S0230: M17100 (**PQ449424**).

**Localities.** Jacó Scar (1817 m), Mound Jaguar (1896 m).

**Remarks.** An undescribed species associated with naturally occurring wood falls.

**Teredinidae stet.**

Fig. 30H

**Material examined.** AD4588: M12290.

**Localities.** Mound 12 (995 m).

**Remarks.** Associated with a naturally occurring wood fall. DNA sequences could not be obtained.

#### ﻿Mollusca | Bivalvia | Heterodonta | Anomalodesmata | Cuspidariidae

***Bathyneaeratillamookensis* (Dall, 1916)**

Fig. 31A

**Figure 31.**
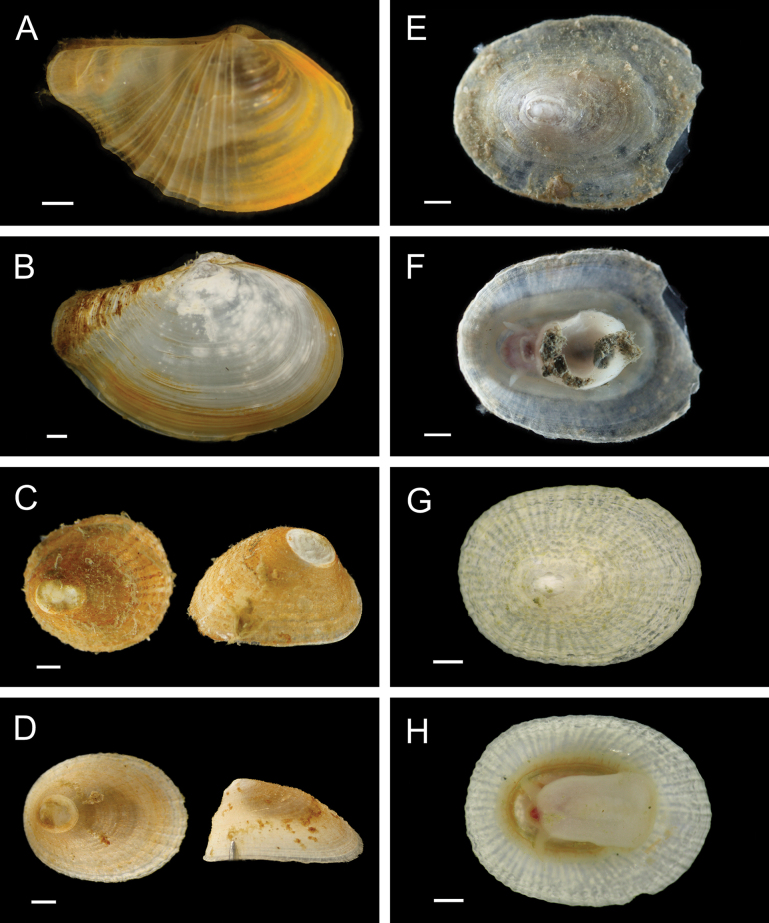
Mollusca: Bivalvia and Gastropoda: Patellogastropoda, representative live images **A***Bathyneaeratillamookensis* (M16982) **B***Luzoniachilensis* (M16808) **C***Iothia* stet. (M16172, separate specimens in dorsal and lateral view) **D***Iothia* stet. (M16173, separate specimens in dorsal and lateral view) **E***Eulepetopsis* gen. inc. (M17882, dorsal view) **F***Eulepetopsis* gen. inc. (M17882, ventral view) **G**Neolepetopsidae stet. (M11963, dorsal view) **H**Neolepetopsidae stet. (M11963, ventral view). Scale bars: 1 mm.

**Material examined.** AD4989: M16982.

**Localities.** Jacó Scar (1762 m).

**Distribution.** Originally described from the Oregon margin, 1438 m, and known from British Columbia, Canada, to Peru as well as the Atlantic and New Zealand, 439–2850 m (Coan and Valentich-Scott 2012).

***Luzoniachilensis* (Dall, 1890)**

Fig. 31B

**Material examined.** AD4508: M12026; AD4974: M16769; AD4977: M16808; AD4989: M16980, M16981, M16983.

**Localities.** Mound 12 (990 m), Parrita Seep (1402 m), Jacó Scar (1762–1783 m).

**Distribution.** Originally described from Isla Mocha, central Chile, 1238 m, and known from the U.S. Pacific coast off Washington to southern Chile, 100–1875 m (Coan and Valentich-Scott 2012).

#### ﻿Mollusca | Gastropoda

We list the six widely accepted major gastropod clades (Bouchet et al. 2017) in a sequence reflecting their current phylogenomic relationships (Uribe et al. 2022; Zhong et al. 2022), acknowledging the possibility of further revision of higher-level clade names. Within these clades we organize the listings following WoRMS. We recognize the need for considerable further taxonomic and genetic work, especially on limpet-shaped forms such as Neolepetopsidae and Cocculiniformia (Desbruyères et al. 2006; Chen et al. 2019b, 2023). Several gastropod morphospecies occurring at Jacó Scar were provisionally identified in Electronic supplemental table S6 of Levin et al. (2012) and are here linked to voucher specimens where possible.

#### ﻿Mollusca | Gastropoda | Patellogastropoda | Lottioidea | Lepetidae

***Iothia* stet.**

Fig. 31C, D

**Reference.** Electronic supplemental table S6 of Levin et al. (2012) (occurrences only, Jacó Scar).

**Material examined.** AD4508: M12025 (**PQ450378**); AD4916: M16172, M16173.

**Localities.** Parrita Seep (~ 1401–1419 m), Jacó Scar (1611 m).

**Remarks.** For M12025, the closest COI BLASTN results on GenBank were specimens of *Iothiafulva* (O. F. Müller, 1776) from the North Sea (KR084663.1, KR084581.1; 94.53% identity).

#### ﻿Mollusca | Gastropoda | Patellogastropoda | Lottioidea | Neolepetopsidae

***Eulepetopsis* gen. inc.**

Fig. 31E, F

**Reference.** Electronic supplemental table S6 of Levin et al. (2012) as *Eulepetopsis* sp. (occurrences only, Jacó Scar).

**Material examined.** AD4506: M12004; AD4590: M17882, M18921, M19153.

**Localities.** Parrita Seep (1186 m), Jacó Scar (~ 1800 m).

**Remarks.** Morphologically similar to *Eulepetopsis*, although the two described species of *Eulepetopsis* are known only from high-temperature vents and Neolepetopsidae may require revision (Chen et al. 2023).

**Neolepetopsidae stet.**

Fig. 31G, H

**Reference.** Several morphospecies were reported in Electronic supplemental table S6 of Levin et al. (2012) (occurrences only, Jacó Scar).

**Material examined.** AD4501: M11963; AD4502: M12093; AD4586: M18777, M18780, M18800, M18802, M18804, M18807; AD4587: M19015, M19116, M19155, M19156; AD4588: M19019, M19020, M19025, M19031, M19034, M19035, M19040, M19041, M19060, M19065, M19071, M19097, M19108, M19138, M19046, M19050, M19053, M19064, M19070, M19084, M19154; AD4589: M18813, M18827, M18839, M18842, M18848, M18858, M18864, M18910, M18918, M18926, M18929, M18958, M18964, M18966, M19139, M19140, M19141, M19157; AD4590: M18990; AD4591: M18874, M18882, M18885, M18892, M18902, M18903, M19163.

**Localities.** Mound 12 (~ 1000 m), Jacó Scar (~ 1800 m).

**Remarks.** Under genetic and morphological investigation (Betters et al. (2024), in press at the time of this work’s acceptance). Neolepetopsidae and *Paralepetopsis* appear to be paraphyletic (Chen et al. 2023).

**Paralepetopsiscf.clementensis J. H. McLean, 2008**

Fig. 32A

**Figure 32.**
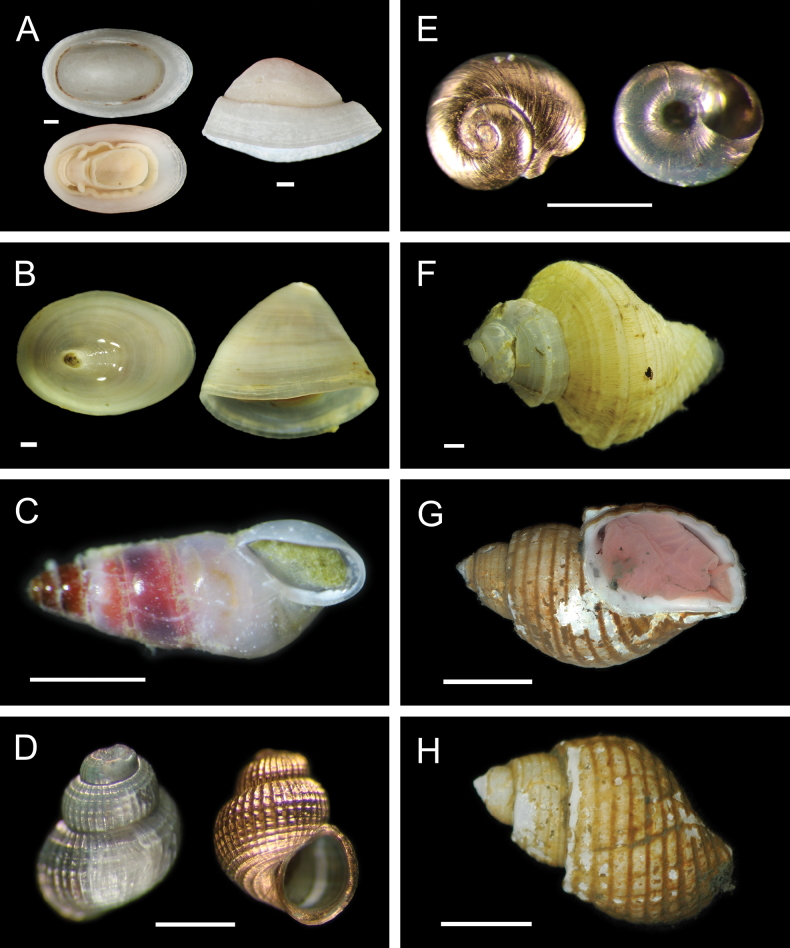
Mollusca: Gastropoda: Patellogastropoda and Caenogastropoda, representative images. Live specimens are depicted unless otherwise specified **A**Paralepetopsiscf.clementensis (M18937, preserved specimen in dorsal, ventral, and lateral view) **B***Bathyacmaea* stet. (M11987, same specimen in dorsal and lateral view) **C***Fuscapex* stet. (M12006) **D***Alvania* stet. (M18834, same preserved specimen in lateral and apertural view) **E**Vitrinellidae stet. (M19091, same preserved specimen in apical and umbilical view) **F***Neptunea* stet. (M12023) **G**Cancellarianr.rosewateri (M12051, apertural view) **H**Cancellarianr.rosewateri (M12051, lateral view). Scale bars: 1 mm (**A–F**); 1 cm (**G, H**).

**Reference.** Reported in Electronic supplemental table S6 of Levin et al. (2012) as “*Neolepetopsis* [sic] *clementensis*” (occurrences only).

**Material examined.** AD4590: M18930, M18935, M18937, M18961, M18969, M18976, M18980, M18989, M19142; AD4591: M18884, M18901.

**Localities.** Jacó Scar (~ 1800 m).

**Distribution.***Paralepetopsisclementensis* is known only from a whale fall off southern California at 1800 m depth (McLean 2008). Genetic confirmation of the CRM seep occurrences would represent new southern records and the first seep records for this species. Additional specimens of *Paralepetopsis* are under morphological and genetic investigation (Betters et al. (2024), in press at the time of this work’s acceptance).

#### ﻿Mollusca | Gastropoda | Patellogastropoda | Lottioidea | Pectinodontidae

***Bathyacmaea* stet.**

Fig. 32B

**Reference.** Electronic supplemental table S6 of Levin et al. (2012) (occurrences only, Jacó Scar).

**Material examined.** AD4503: M11987; AD4587: M18812; AD4590: M18925, M18928, M18945, M18953, M18960, M18968, M18978, M18987; AD4591: M18873, M18881, M18888, M18893, M19151.

**Localities.** Mound 12 (~ 1000 m), Jacó Scar (~ 1800 m).

**Remarks.** Under genetic and morphological investigation (Betters et al. (2024), in press at the time of this work’s acceptance).

#### ﻿Mollusca | Gastropoda | Caenogastropoda | Littorinimorpha | Eulimidae

***Fuscapex* stet.**

Fig. 32C

**Material examined.** AD4506: M12006.

**Localities.** Parrita Seep (~ 1030–1033 m).

**Remarks.** The host of this specimen was not determined, but *Fuscapex* is only known as a parasite on ophiuroids (Bouchet and Warén 1986). The specimen was recovered among various animals associated with a coralliid (voucher specimen Co2271), including potential hosts *Ophiacanthamoniliformis* (voucher specimen E4390) and Ophiuroglyphacf.meridionalis (voucher specimen E7978).

#### ﻿Mollusca | Gastropoda | Caenogastropoda | Littorinimorpha | Rissoidae

***Alvania* stet.**

Fig. 32D

**Material examined.** AD4589: M18834.

**Localities.** Mound 12 (997 m).

**Remarks.** Associated with a naturally occurring wood fall.

#### ﻿Mollusca | Gastropoda | Caenogastropoda | Littorinimorpha | Vitrinellidae

**Vitrinellidae stet.**

Fig. 32E

**Material examined.** AD4587: M19091.

**Localities.** Mound 12 (996 m).

**Remarks.** Associated with a naturally occurring wood fall.

#### ﻿Mollusca | Gastropoda | Caenogastropoda | Neogastropoda | Buccinidae

***Neptuneaamianta* (Dall, 1890) sp. inc.**

Fig. 32F

**Material examined.** AD4508: M12023; AD4913: M16143; S0230: M17099.

**Localities.** Parrita Seep (~ 1401–1419 m), Jacó Scar (1847 m), Mound Jaguar (1908 m).

**Remarks.** Possibly a southern range extension of *Neptuneaamianta*, which was originally described from southern California at 757 m and has been reported from the Bering Sea off Alaska to northern Baja California, 100–3500 m (McLean 1996). Scavenging aggregations of *N.amianta* have been associated with organic falls and seeps (McClain and Nekola 2007).

#### ﻿Mollusca | Gastropoda | Caenogastropoda | Neogastropoda | Cancellariidae

**Cancellarianr.rosewateri Petit, 1983**

Fig. 32G, H

**Material examined.** AD4510: M12051.

**Localities.** Jacó Summit (744 m).

**Remarks.** This specimen warrants further comparison to *Cancellariarosewateri*, described from the Gulf of Mexico, 366 m (Petit 1983).

#### ﻿Mollusca | Gastropoda | Caenogastropoda | Neogastropoda | Raphitomidae

***Gymnobela* stet.**

Fig. 33A

**Figure 33.**
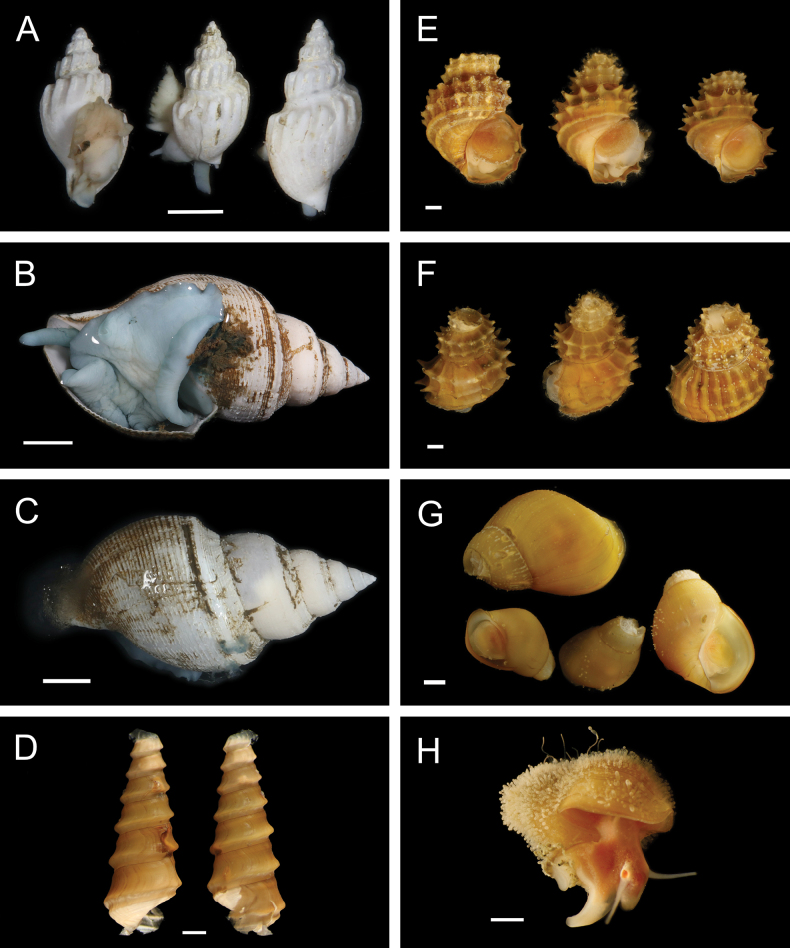
Mollusca: Gastropoda: Caenogastropoda, representative images. Live specimens are depicted unless otherwise specified **A***Gymnobela* stet. (M17041, three specimens) **B***Phymorhynchus* gen. inc. (M17038, apertural view) **C***Phymorhynchus* gen. inc. (M17038, lateral view) **D***Abyssochrysos* stet. (M18994, same preserved specimen in lateral and apertural view) **E***Provannaios* (M16807, apertural views) **F***Provannaios* (M16807, apico-lateral views) **G***Provannalaevis* (M16104) **H***Provannalaevis* (M17030, with bacteria). Scale bars: 1 cm (**A–C**); 1 mm (**D–H**).

**Material examined.** AD4504: M11990 (**PQ449396**); AD4988: M16958; S0217: M17041.

**Localities.** Mound 11 (~ 999–1025 m), The Thumb (1069 m).

**Remarks.** For M11990, the closest COI BLASTN result on GenBank was a specimen of *Typhlosyrinx* (Raphitomidae) from Papua New Guinea, 680–689 m (MH308407.1, 93.26% identity) (Uribe et al. 2018).

***Phymorhynchus* gen. inc.**

Fig. 33B, C

**Material examined.** S0214: M17038.

**Localities.** Jacó Scar (1801 m).

#### ﻿Mollusca | Gastropoda | Caenogastropoda | Abyssochrysoidea | Abyssochrysidae

***Abyssochrysos* stet.**

Fig. 33D

**Material examined.** AD4587: M18994.

**Localities.** Mound 12 (~ 9900–996 m).

#### ﻿Mollusca | Gastropoda | Caenogastropoda | Abyssochrysoidea | Provannidae

***Provannaios* Warén & Bouchet, 1986**

Fig. 33E, F

**Reference.**Betters and Cordes 2024.

**Localities.** Jacó Scar (~ 1800–2000 m) and an unnamed locality (8.5958, -84.4370) at 1917 m depth, ca 12 km northwest of Parrita Seep.

**Distribution.** Eastern Pacific vents, 21°N to 17°S (type locality: 13°N on the East Pacific Rise) (Warén and Bouchet 1986), and the CRM seeps, 1757–2620 m depth (Sasaki et al. 2010; Betters and Cordes 2024).

***Provannalaevis* Warén & Ponder, 1991**

Fig. 33G, H

**References.**Betters and Cordes 2024.

**Localities.** Jacó Summit (~ 740–760 m), Mound 12 (~ 900–1050 m), The Thumb (~ 1071–1075 m).

**Distribution.** Originally described from the Guaymas Basin, Gulf of California, 2004 m (Warén and Ponder 1991), and reported at eastern Pacific vents and seeps from the Juan de Fuca Ridge to the CRM, 500–2004 m depth (Warén and Bouchet 2001; Betters and Cordes 2024). A recent synonymization of *Provannaglabra* Okutani, Tsuchida & Fujikura, 1992, extends the range of *P.laevis* to western Pacific seeps from Iheya Ridge to Sagami Bay (Betters and Cordes 2024).

**Remarks.** Some CRM specimens were associated with naturally occurring or experimentally deployed wood (Betters et al. 2023; Betters and Cordes 2024). Consistent with previous reports from the Oregon Margin (Warén and Bouchet 2001), Pyropeltacf.corymba was often attached to the shells. Some *P.laevis* shells were coated with elongated pustules (Fig. 33H) resembling the *Thiomargarita*-like sulfur-oxidizing bacteria reported on other gastropods from the CRM seeps (Bailey et al. 2011). Similar pustules have been observed on the shells of vent gastropods such as *Depressigyra* spp. (Warén and Bouchet 1989; Desbruyères et al. 2006) and *Lepetodrilusgordensis* S. B. Johnson, C. R. Young, W. J. Jones, Warén & Vrijenhoek, 2006 (Johnson et al. 2006).

***Provannapacifica* (Dall, 1908)**

Fig. 34A, B

**Figure 34.**
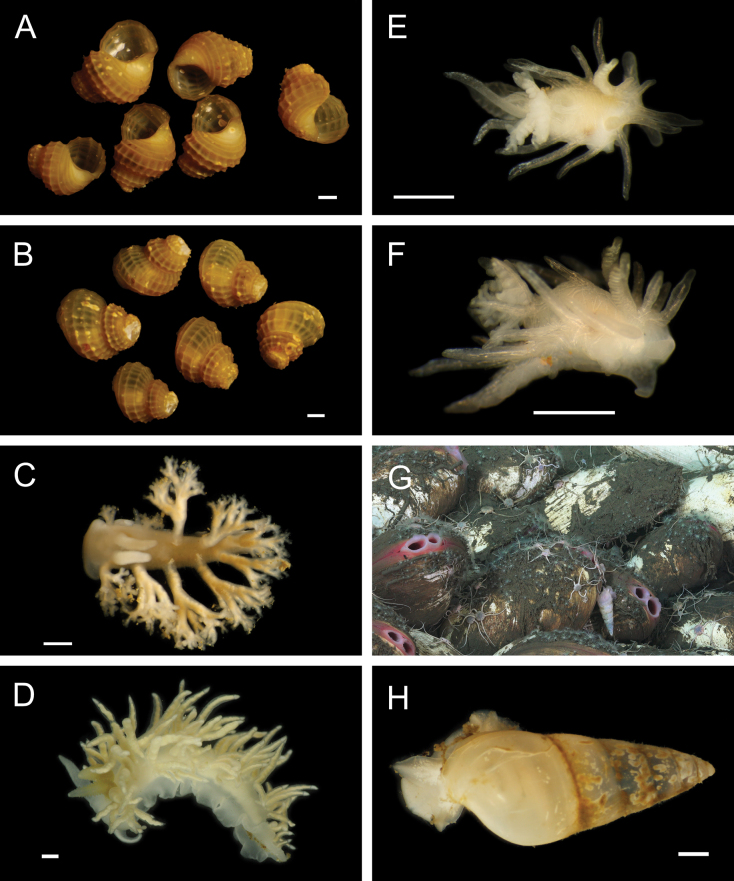
Mollusca: Gastropoda: Caenogastropoda and Heterobranchia, representative live images **A***Provannapacifica* (M16955, apertural views) **B***Provannapacifica* (M16955, lateral views) **C**Aeolidioidea stet. (M16811) **D**Fionoidea stet. (M11992) **E**Goniodorididae sp. SIO_BIC_M16185 (M16185, dorsal view) **F**Goniodorididae sp. SIO_BIC_M16185 (M16185, lateral view) **G***Eulimellalomana* (*in situ*), Dive S0214 at Jacó Scar, 1781 m. Credit: ROV SuBastian/Schmidt Ocean Institute **H**Pyramidellidae stet. (M16901). Scale bars: 1 mm.

**Reference.**Betters and Cordes 2024.

**Localities.** Mound 11 (~ 1000–1100 m), Parrita Seep (~ 1300–1500 m).

**Distribution.** Associated with naturally occurring sunken wood and seeps, Oregon Margin to the Gulf of Panama (type locality), 1000–2750 m (Warén and Bouchet 1986; Betters and Cordes 2024).

#### ﻿Mollusca | Gastropoda | Heterobranchia | Euthyneura | Ringipleura | Nudibranchia | Cladobranchia

**Aeolidioidea stet.**

Fig. 34C

**Material examined.** AD4975: M16811; AD4985: M16907.

**Localities.** Mound 12 (997–1002 m).

**Fionoidea stet.**

Fig. 34D

**Material examined.** AD4504: M11992; AD4906: M16094.

**Localities.** Mound 11 (~ 1004–1011 m), Mound 12 (~ 997–1002 m).

**Remarks.** M11992 was associated with naturally occurring sunken plant material.

#### ﻿Mollusca | Gastropoda | Heterobranchia | Euthyneura | Ringipleura | Nudibranchia | Doridina

**Goniodorididae sp. SIO_BIC_M16185**

Fig. 34E, F

**Material examined.** AD4918: M16185 (**PQ449415**).

**Localities.** Quepos Slide (394 m).

**Remarks.** The closest COI BLASTN results on GenBank were within Goniodorididae, e.g., *Ceratodorispilosa* (Bouchet & Ortea, 1983) from Japan (MW357567.1, 88.94% identity), *Okeniamediterranea* (Ihering, 1886) from Italy (MK645760.1, 88.78% identity), and *O.amoenula* (Bergh, 1907) from South Africa (KF192606.1, 88.63% identity).

#### ﻿Mollusca | Gastropoda | Heterobranchia | Euthyneura | Tectipleura | Pyramidellidae

***Eulimellalomana* (Dall, 1908)**

Fig. 34G

**Reference.** Electronic supplemental table S6 of Levin et al. (2012) (occurrences only, Jacó Scar).

**Material examined.** AD4590: M18971.

**Localities.** Jacó Scar (~ 1800 m).

**Distribution.** Known from seeps, vents (likely sedimented vents), and whale falls, 1168–2008 m, from southern California (type locality) and the Guaymas Basin, Gulf of California (Warén and Bouchet 1993; Sasaki et al. 2010; Portail et al. 2015).

**New records.** The CRM specimen represents a new southern record for this species.

**Remarks.** Pyramidellids are thought to be exclusively parasitic, typically on mollusks or polychaetes, but a definitive host has not been identified for *E.lomana* (Warén and Bouchet 1993). Specimen M18971 could not be linked to a potential host. Another specimen at Jacó Scar (not collected) was observed on vesicomyid clams (Fig. 34G).

**Pyramidellidae stet.**

Fig. 34H

**Material examined.** AD4508: M12029; AD4912: M16125; AD4987: M16901; AD4989: M16942.

**Localities.** Mound 12 (1010 m), Parrita Seep (~ 1401–1419 m), Jacó Scar (1842 m).

**Remarks.** At least one additional morphospecies is present and distinct from *E.lomana*.

#### ﻿Mollusca | Gastropoda | Heterobranchia | Mesoneura | Tjaernoeioidea | Aplustridae

***Parvaplustrum* stet.**

Fig. 35A, B

**Figure 35.**
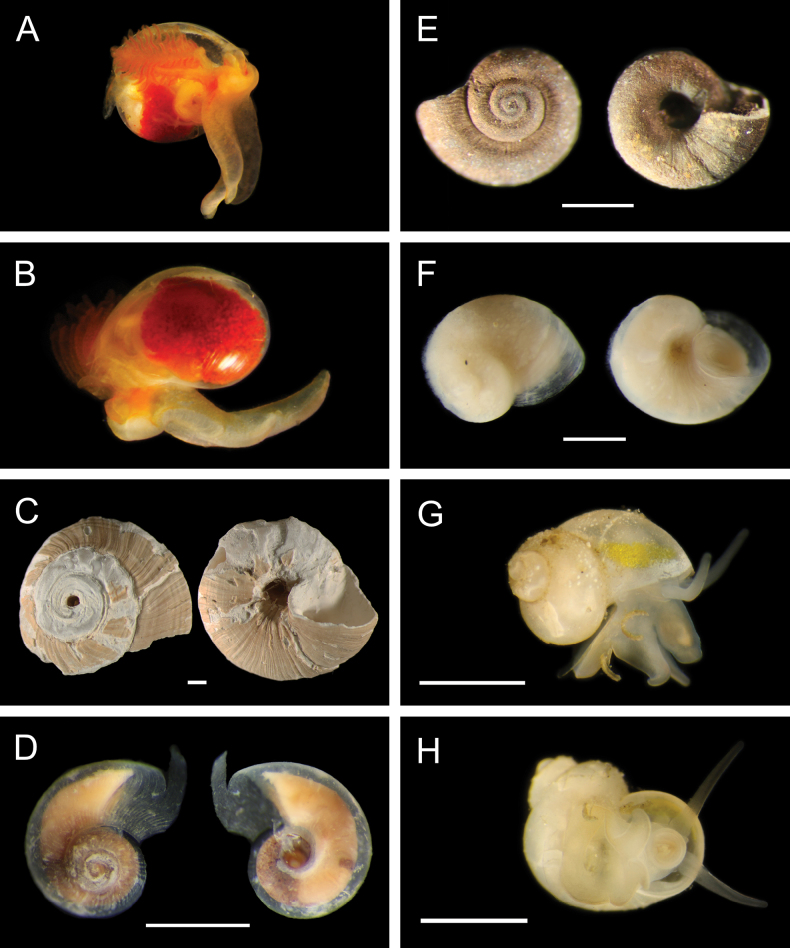
Mollusca: Gastropoda: Heterobranchia, representative images. Live specimens are depicted unless otherwise specified **A***Parvaplustrum* stet. (M17071, apertural view; scale not recorded for specimens destroyed in DNA extraction; estimated shell length 1-2 mm) **B***Parvaplustrum* stet. (M17071, lateral view) **C**Architectonicidae fam. inc. (M19335, same preserved specimen in apical and umbilical view) **D***Lurifax* gen. inc. (M19337, same preserved specimen in apical and umbilical view) **E***Orbitestella* gen. inc. (M19136, same preserved specimen in apical and umbilical view) **F***Hyalogyra* stet. (M18944, same preserved specimen in apical and umbilical view) **G***Hyalogyrina* sp. SIO_BIC_M16774 (M16774, lateral view) **H***Hyalogyrina* sp. SIO_BIC_M16774 (M16774, ventral view). Scale bars: 1 mm.

**Material examined.** S0219: M17071.

**Localities.** Rio Bongo Scar (609 m).

**Remarks.** Collected from a microbial mat. DNA sequences could not be obtained.

#### ﻿Mollusca | Gastropoda | Heterobranchia | “Lower Heterobranchia” | Architectonicoidea | Architectonicidae

**Architectonicidae fam. inc.**

Fig. 35C

**Material examined.** AD4505: M19335.

**Localities.** Mound 11 (1025 m).

**Remarks.** Identification is uncertain due to the corroded condition of the shell.

#### ﻿Mollusca | Gastropoda | Heterobranchia | “Lower Heterobranchia” | Orbitestelloidea | Orbitestellidae

***Lurifax* gen. inc.**

Fig. 35D

**Material examined.** AD4504: M19337; AD4505: M19336.

**Localities.** Mound 11 (1009–1025 m).

**Remarks.** Identification is uncertain.

***Orbitestella* gen. inc.**

Fig. 35E

**Material examined.** AD4589: M19136.

**Localities.** Mound 12 (997 m).

**Remarks.** Identification is uncertain due to the corroded condition of the shell.

#### ﻿Mollusca | Gastropoda | Heterobranchia | “Lower Heterobranchia” | Valvatoidea | Hyalogyrinidae

***Hyalogyra* stet.**

Fig. 35F

**Material examined.** AD4586: M18799; AD4587: M19010, M19087; AD4588: M19026, M19043, M19074, M19088, M19135; AD4589: M18837, M19137; AD4590: M18907, M18919, M18920, M18942, M18944, M19145, M19338.

**Localities.** Mound 12 (~ 1000 m), Jacó Scar (~ 1800 m).

**Remarks.** Two morphospecies were distinguished as “tall” and “planispiral”.

***Hyalogyrina* stet.**

Fig. 35G, H

**Reference.** Electronic supplemental table S6 of Levin et al. (2012) (occurrences only, Jacó Scar).

**Material examined.** AD4510: M12043; AD4587: M13003, M13004, M18991, M18992, M19000, M19001, M19007, M19144, M19147; AD4590: M18323, M18981, M19330; AD4914: M16151 (**PQ449414**); AD4974: M16774; S0213: M17029 (**PQ449419**).

**Localities.** Jacó Summit (741–742 m), Mound 12 (~ 1000 m), Jacó Scar (~ 1800 m).

**Remarks.** Most specimens were collected from microbial mat habitats. At least two morphospecies were distinguished (Levin et al. 2012). One morphospecies, represented by M17029, has a pale shell with a broad yellow blaze on the outer whorl. The CRM specimens warrant comparison to *Hyalogyrinagrasslei* Warén & Bouchet, 1993, which is known from hydrothermal vents and seeps at ~ 2000 m in the Guaymas Basin, Gulf of California (Warén and Bouchet 1993; Sasaki et al. 2010).

#### ﻿Mollusca | Gastropoda | Vetigastropoda | Lepetellida | Lepetelloidea | Caymanabyssiidae

***Colotrachelus* stet.**

Fig. 36A

**Figure 36.**
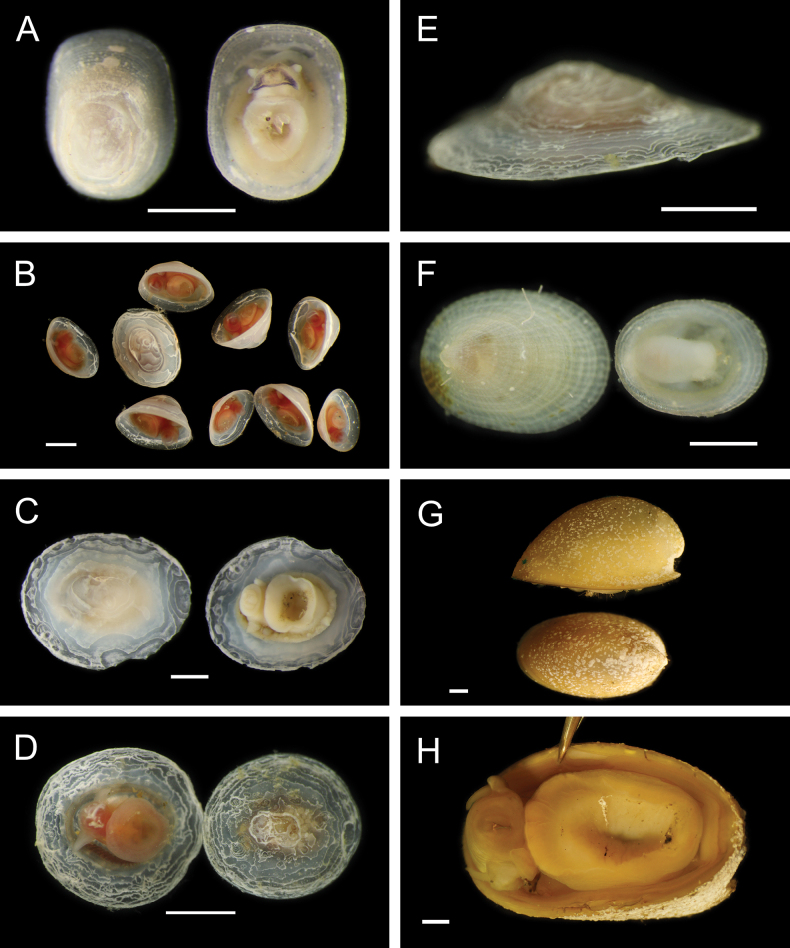
Mollusca: Gastropoda: Vetigastropoda, representative images. Live specimens are depicted unless otherwise specified **A***Colotrachelus* stet. (M19101, separate preserved specimens in dorsal and ventral view) **B**Pyropeltacf.corymba (M16105) **C**Pyropeltacf.musaica (M19044, same preserved specimen in dorsal and ventral view) **D**Pyropeltacf.wakefieldi (M11966, separate specimens in dorsal and ventral view) **E**Pyropeltacf.wakefieldi (M11966, lateral view) **F**Lepetodrilusaff.shannonae (M11973, separate specimens in dorsal and ventral view) **G***Lepetodrilusguaymasensis* (M16142, separate specimens in lateral and dorsal view) **H***Lepetodrilusguaymasensis* (M16142, ventral view). Scale bars: 1 mm.

**Material examined.** AD4587: M19093, M19101, M19120, M19129.

**Localities.** Mound 12 (~ 1000 m).

#### ﻿Mollusca | Gastropoda | Vetigastropoda | Lepetellida | Lepetelloidea | Pyropeltidae

**Pyropeltacf.corymba McLean & Haszprunar, 1987**

Fig. 36B

**Reference.** Reported in Electronic supplemental table S6 of Levin et al. (2012) as *Pyropeltacorymba* (occurrences only, Jacó Scar).

**Material examined.** AD4501: M11968; AD4586: M18801; AD4587: M19096, M19343; AD4588: M19033, M19049, M19059, M19073, M19160, M19347; AD4589: M18322, M18830, M18841, M18851, M18863, M19078; AD4590: M18908; AD4910: M16105, M16107; AD4917: M16157; AD4978: M16874.

**Localities.** Mound 12 (~ 1000 m), Jacó Scar (~ 1800 m).

**Remarks.***Pyropeltacorymba* was originally described from hydrothermal vents in the Guaymas Basin, Gulf of California, 2022 m (McLean and Haszprunar 1987), and subsequently reported from seeps on the Oregon Margin, 524 m (Warén and Bouchet 2001), as well as from whale falls off southern California, 940–1240 m (McLean 1992). If confirmed as *P.corymba* by genetic comparison to material from the type locality, the CRM seep specimens would represent new southern records for this species. Consistent with previous reports (Warén and Bouchet 2001; Desbruyères et al. 2006), many CRM specimens were associated with *Provannalaevis*.

**Pyropeltacf.musaica McLean & Haszprunar, 1987**

Fig. 36C

**Material examined.** AD4586: M17850, M18781, M18811, M19348; AD4587: M19008, M19090, M19109, M19113, M19122; AD4588: M19022, M19023, M19032, M19044, M19054, M19066, M19085, M19162, M19346; AD4589: M18831, M18843, M18850, M19077; AD4590: M18940, M18988; AD4591: M18324, M18883, M18900.

**Localities.** Mound 12 (~ 1000 m), Jacó Scar (~ 1800 m).

**Remarks.***Pyropeltamusaica* was originally described from hydrothermal vents at the Axial Seamount, Juan de Fuca Ridge, 1575 m (McLean and Haszprunar 1987). It has been reported from the Jalisco Block seeps, Mexico, at 3000–3775 m (Warén and Bouchet 2001; Desbruyères et al. 2006), and from whale falls across California at 940–1400 m (McLean 1992). If confirmed as *P.musaica* by genetic comparison to material from the type locality, the CRM seep occurrences would represent new southern records for this species.

**Pyropeltacf.wakefieldi McLean, 1992**

Fig. 36D, E

**Material examined.** AD4501: M11966 (**PQ450396**); AD4586: M17849, M18797, M18803, M18808, M18810; AD4587: M19011, M19016, M19112; AD4588: M19021, M19030, M19048, M19072, M19075, M19134, M19148, M19150, M19340, M19341, M19342, M19344; AD4589: M18829, M18835, M18849, M18857, M18862, M19076; AD4590: M18956, M18959.

**Localities.** Mound 12 (~ 1000 m), Jacó Scar (~ 1800 m).

**Remarks.***Pyropeltawakefieldi* was originally described from a whale fall off Point Sur, California, 940 m (McLean 1992). Genetic confirmation of the CRM seep occurrences would represent new southern records and new seep records for this species. The closest COI BLASTN result on GenBank was *Pyropelta* sp. SWA-2009 from the Gulf of Mexico (FJ977753.1, 90.99% identity).

#### ﻿Mollusca | Gastropoda | Vetigastropoda | Lepetellida | Lepetodriloidea | Lepetodrilidae

**Lepetodrilusaff.shannonae Warén & Bouchet, 2009**

Fig. 36F

**Reference.** Electronic supplemental table S6 of Levin et al. (2012) (occurrences only, Jacó Scar).

**Material examined.** AD4501: M11973; AD4511: M12057; AD4590: M18904, M18943, M18985, M19152; AD4591: M18875, M18879, M18891, M18898.

**Localities.** Mound 12 (~ 1000 m), Jacó Scar (~ 1800 m).

**Remarks.** The CRM specimens appear morphologically similar to *L.shannonae*, known only from hydrocarbon seeps off the Congo River, West Africa, 2300–3150 m (Warén and Bouchet 2009).

***Lepetodrilusguaymasensis* McLean, 1988**

Fig. 36G, H

**References**: Johnson et al. 2008; Matabos and Jollivet 2019; Electronic supplemental table S6 of Levin et al. (2012) (occurrences only, Jacó Scar).

**Additional material examined.** AD4508: M12020 (**PQ450397**); AD4586: M18778, M18809; AD4587: M19107, M19111, M19118; AD4588: M19018, M19028, M19058, M19069; AD4589: M18817, M18828, M18836, M18845, M18856, M18860; AD4590: M18911, M18914, M18922, M18927, M18938, M18947, M18950, M18967, M18970, M18979, M18983, M19349 to M19382; AD4591: M18872, M18878, M18890, M18897; AD4912: M16142; AD4917: M16159.

**Localities.** Mound 12 (~ 1000 m; this study), Parrita Seep (~ 1400 m; this study), Jacó Scar (~ 1800 m), “Mudpie” seep ~ 10 km west of Parrita Scar (8.983, -84.717; 1917 m) (Johnson et al. 2008).

**Distribution.** Originally described from sedimented hydrothermal vents and seeps in the Guaymas Basin, Gulf of California, 2000–2019 m, often in association with *Riftiapachyptila* Jones, 1981 (McLean 1988, 1993; Sasaki et al. 2010). Specimens from the CRM “Mudpie” seep were confirmed as *L.guaymasensis* based on COI comparison to specimens from the type locality (Matabos and Jollivet 2019).

**Remarks.** The COI sequence of M12020 was 100.00% identical to that of *L.guaymasensis* from the CRM Mudpie site (EU306419.1, voucher SMNH 82443, Swedish Museum of Natural History).

***Lepetodrilus* stet.**

Fig. 37A

**Figure 37.**
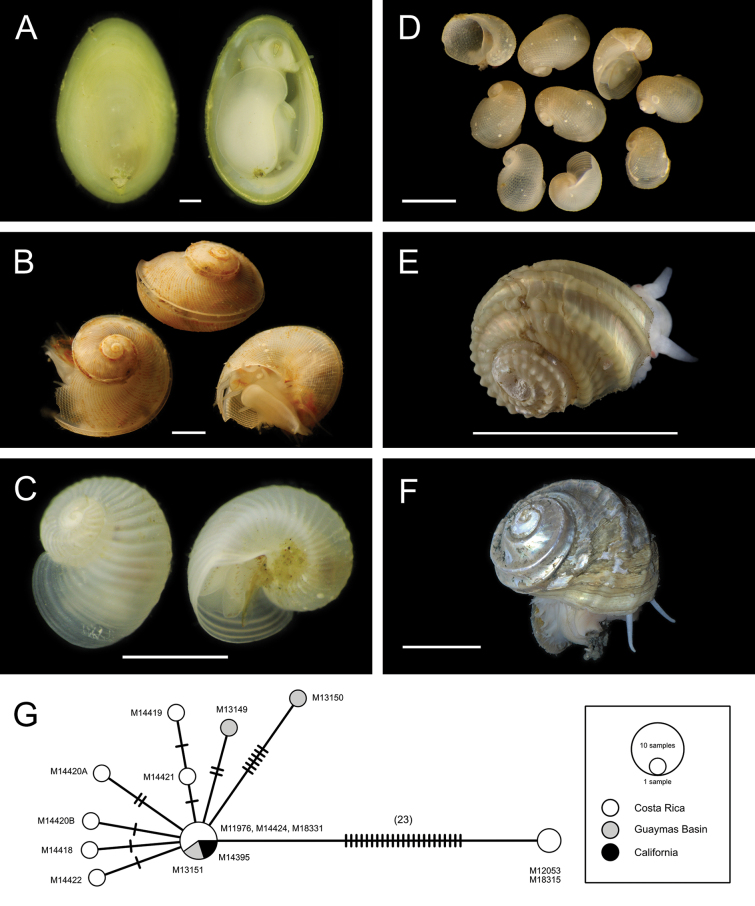
Mollusca: Gastropoda: Vetigastropoda, representative live images **A***Lepetodrilus* stet (M11965, same specimen in dorsal and ventral view) **B***Anatoma* stet. (M16187, same specimen in apical, lateral, and umbilical view) **C***Bathyxylophila* stet. (M12038, separate specimens in apical and umbilical view) **D***Bathyxylophila* stet. (M16199) **E***Kanoiamyronfeinbergi* (M16771) **F**Kanoiacf.myronfeinbergi (M12053) **G** Haplotype network of *Kanoia*COI sequences. Scale bars: 1 mm (**A–D**); 1 cm (**E, F**).

**Material examined.** AD4501: M11965.

**Localities.** Mound 12 (~ 984–997 m).

**Remarks.** Additional *Lepetodrilus* morphospecies may occur at the CRM. For example, an undescribed *Lepetodrilus* “sp. CR” has been reported from a CRM seep site at 1900 m, but it is represented by a single specimen without available DNA sequences (Johnson et al. 2008). Specimen M11965 and others resemble *L.elevatus* J. H. McLean, 1988, which was originally described as two subspecies from the East Pacific Rise and the Galápagos Rift (McLean 1988). The *L.elevatus* species complex comprises four genetically distinct lineages (Johnson et al. 2008; Matabos and Jollivet 2019). Additional specimens of Lepetodrilidae from the CRM are under genetic and morphological investigation (Betters et al. (2024), in press at the time of this work’s acceptance).

#### ﻿Mollusca | Gastropoda | Vetigastropoda | Lepetellida | Scissurelloidea | Anatomidae

***Anatoma* stet.**

Fig. 37B

**Reference.** Electronic supplemental table S6 of Levin et al. (2012) (occurrences only, Jacó Scar).

**Material examined.** AD4591: M18899; AD4918: M16187.

**Localities.** Quepos Slide (~ 333–408 m), Jacó Scar (1753 m).

#### ﻿Mollusca | Gastropoda | Vetigastropoda | Lepetellida | Scissurelloidea | Larocheidae

***Bathyxylophila* stet.**

Fig. 37C, D

**Material examined.** AD4508: M12028; AD4509: M12038, M12039, M18869; AD4587: M18317, M19092, M19099, M19128; AD4923: M16198, M16199; AD4976: M16797, M16798, M16801; AD4988: M16954, M16965.

**Localities.** Mound 12 (~ 1000 m), Mound 11 (1010 m), Parrita Seep (~ 1000–1400 m), Jacó Scar (~ 1800 m).

**Remarks.** Associated with naturally occurring or experimentally deployed wood falls. Likely at least two morphospecies are represented. Some of the specimens of M16199 (Fig. 37D) require further verification.

#### ﻿Mollusca | Gastropoda | Vetigastropoda | Seguenziida | Seguenzioidea | Cataegidae

***Kanoiamyronfeinbergi* Warén & Rouse, 2016**

Fig. 37E

**References.**Warén and Rouse 2016**. Prior to description, this morphospecies was reported at Jacó Scar as “*Cataegis* sp.” in Electronic supplemental table S6 of Levin et al. (2012).

**New sequences.** We provide COI sequences for the following specimens cited in Warén and Rouse (2016), in some cases under catalog numbers from the Swedish Museum of Natural History, SMNH: AD4501: M11976 (**PQ449395**); AD4587: M14422 (**PQ449410**; ex SMNH 108692), M14424 (**PQ449411**; ex SMNH 109248); AD4588: M18331 (**PQ449427**; ex SMNH 108742); AD4589: M14418 (**PQ449405**; tissue from voucher SMNH 108441); AD4590: M14421 (**PQ449409**; tissue from voucher SMNH 108604); AD4591: M14419 (**PQ449406**; ex SMNH 108504), M14420A (**PQ449407**; ex SMNH 108525), M14420B (**PQ449408**; ex SMNH 108525); Guaymas Basin seeps: M13149 (**PQ432664**), M13150 (**PQ432665**), M13151 (**PQ432666**); Del Mar seeps: M14395 (**PQ432667**).

**Localities.** Mound 12 (~ 1000 m; type locality), Jacó Scar (~ 1800 m); additional seep sites off Costa Rica and Nicaragua (1002–1917 m) (Warén and Rouse 2016).

**Distribution.** Also known from seeps in the Guaymas Basin at ~ 1570 m and seeps off Del Mar, California, ~ 1020 m (Warén and Rouse 2016).

**Kanoiacf.myronfeinbergi Warén & Rouse, 2016**

Fig. 37F

**Reference.**Warén and Rouse 2016.

**New sequences.** AD4510: M12053 (**PQ449403**); AD4587: M18315 (**PQ449426**; tissue from voucher SMNH 108680). In Warén and Rouse (2016), specimen M12053 was morphologically identified as *K.myronfeinbergi* whereas M18315 was not identified to species due to corrosion of the shell.

**Localities.** Jacó Summit (~ 741–744 m), Mound 12 (~ 990–996 m).

**Remarks.** The description of *K.myronfeinbergi* notes the possibility of a second, cryptic species “with less distinct sculpture, more similar to *Kanoiameroglypta* from the Caribbean,” although detailed assessment of shell sculpture is difficult due to the corrosion on many specimens (Warén and Rouse 2016).

To investigate the genetic basis for this variation, we constructed a COI haplotype network using a subset of the specimens examined in the original description (Fig. 37G). Two COI sequences showed a minimum uncorrected distance of 4.3% (over 539 bp) from the others. Although there is no universally applicable threshold for species delimitation, numerous deep-sea gastropod species are separated by a minimum of 4–7% COI divergence (Johnson et al. 2008; Chen et al. 2019a). We conservatively designate the two high-divergence specimens as K.cf.myronfeinbergi, and we note the co-occurrence of this haplotype with others at Mound 12. The other *K.myronfeinbergi* sequences showed minimal variation across localities from Costa Rica to California (maximum uncorrected distance 1.11%). Further clarification on *Kanoia* species delimitation will require genetic and morphological analysis of more individuals from various localities and depths, especially those < 1000 m to explore potential depth segregation.

#### ﻿Mollusca | Gastropoda | Vetigastropoda | Seguenziida

***Xyloskenea* stet.**

Fig. 38A

**Figure 38.**
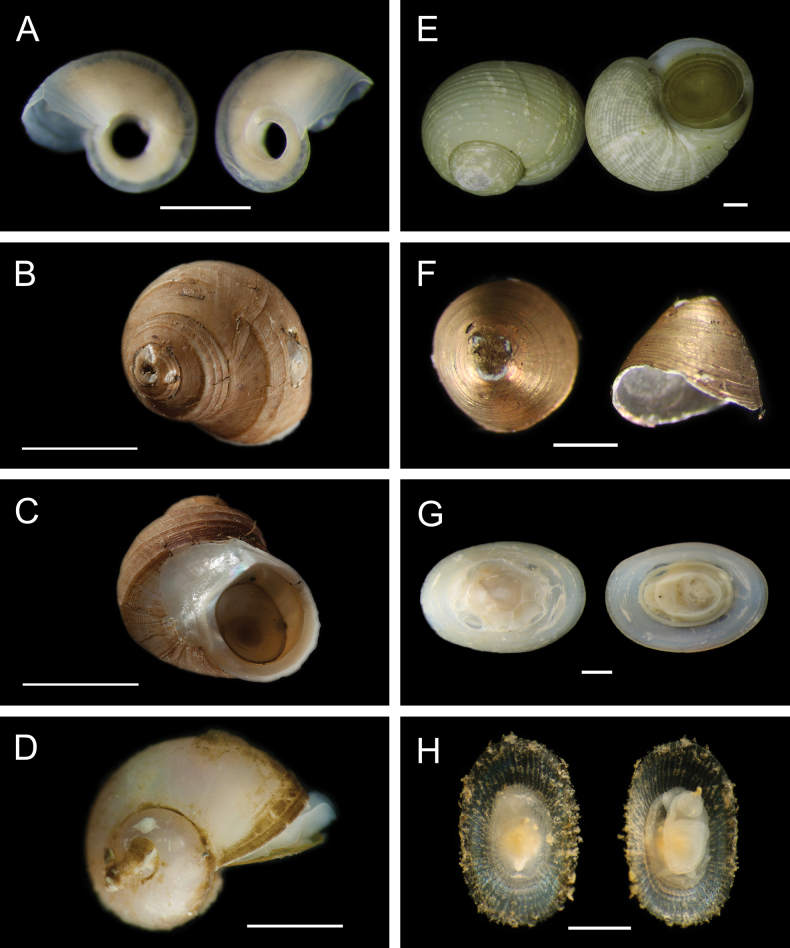
Mollusca: Gastropoda: Vetigastropoda and Neomphaliones, representative images. Live specimens are depicted unless otherwise specified **A***Xyloskenea* stet. (M19013, two preserved specimens) **B***Escondidacantraineapanamensis* (M17848, apical view) **C***Escondidacantraineapanamensis* (M17848, umbilical view) **D***Dillwynella* stet (M17884) **E***Fucaria* stet. (M12068, two specimens) **F***Bathysciadium* stet. (M19002, same preserved specimen in dorsal and lateral view) **G***Cocculina* stet. (M19166, same preserved specimen in dorsal and ventral view) **H**Cocculinidae fam. inc. (M16727, same specimen in dorsal and ventral view). Scale bars: 1 mm (**A, D–H**); 1 cm (**B, C**).

**Material examined.** AD4509: M18870, M19159; AD4587: M19013, M19334; AD4923: M16197.

**Localities.** Mound 12 (~ 1000 m), Parrita Seep (~ 1100 m), Jacó Scar (~ 1800 m).

**Remarks.** M16197 and M18870 were associated with naturally occurring wood falls.

#### ﻿Mollusca | Gastropoda | Vetigastropoda | Trochida | Trochoidea | Colloniidae

***Escondidacantraineapanamensis* (Dall, 1908)**

Fig. 38B, C

**Material examined.** AD4587: M17848, M19103.

**Localities.** Mound 12 (995–996 m).

**Distribution.** Originally described from the Gulf of Panama, 1015 m (Dall 1908), and recorded south to the Concepción seeps off central Chile, 740–870 m (Sasaki et al. 2010).

**Remarks.** M17848 and M19103 were associated with naturally occurring wood falls.

#### ﻿Mollusca | Gastropoda | Vetigastropoda | Trochida | Trochoidea | Skeneidae

***Dillwynella* stet.**

Fig. 38D

**Reference.** Reported in Electronic supplemental table S6 of Levin et al. (2012) as “*Dillwynellapanamensis*” (occurrences only, Jacó Scar).

**Material examined.** AD4586: M18805; AD4587: M18320, M19095, M19098, M19117, M19124, M19158; AD4588: M17884, M19052; AD4589: M18832; AD4590: M18957.

**Localities.** Mound 12 (~ 1000 m), Jacó Scar (~ 1800 m).

**Remarks.** Many specimens were associated with wood falls, either experimentally deployed (M17884, M18805, M19052) or naturally occurring (M18320, M19095, M19098, M19117, M19124, M19158). These specimens warrant comparison to *Ganesapanamensis* Dall, 1902, which is known only from the Gulf of Panama, 1865 m, and has been regarded as possibly belonging to the genus *Dillwynella* (Dall 1902; Kunze 2011).

***Fucaria* stet.**

Fig. 38E

**Reference.** Electronic supplemental table S6 of Levin et al. (2012) (occurrences only, Jacó Scar).

**Material examined.** AD4513: M12068; AD4591: M18880, M18887; AD4590: M18906, M18923, M18941, M18946, M18951, M18986, M19165; AD4591: M18896.

**Localities.** Jacó Scar (~ 1800 m).

#### ﻿Mollusca | Gastropoda | Neomphaliones

We follow the subdivisions of Neomphaliones based on the mitogenome phylogeny in Zhong et al. (2022).

#### ﻿Mollusca | Gastropoda | Neomphaliones | Cocculinida | Bathysciadiidae

***Bathysciadium* stet.**

Fig. 38F

**Material examined.** AD4587: M19002.

**Localities.** Mound 12 (~ 990–996 m).

#### ﻿Mollusca | Gastropoda | Neomphaliones | Cocculinida | Cocculinidae

***Cocculina* stet.**

Fig. 38G

**Material examined.** AD4503: M19166; AD4588: M18321, M19089.

**Localities.** Mound 12 (~ 1000 m).

**Cocculinidae fam. inc.**

Figs 38H, 39A, B

**Figure 39.**
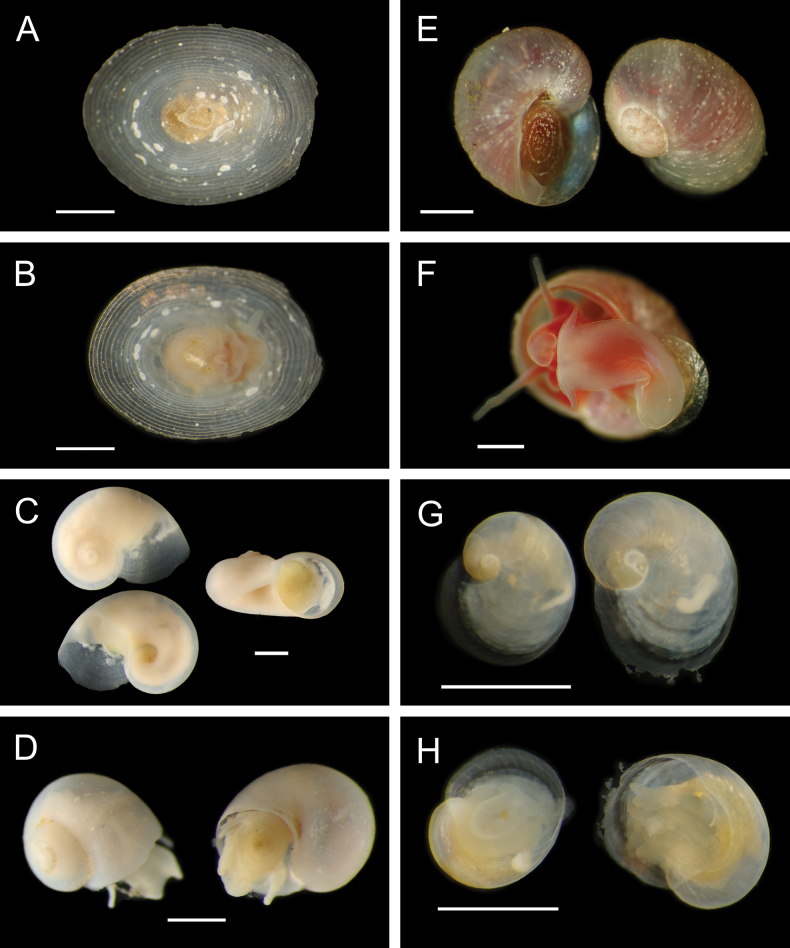
Mollusca: Gastropoda: Neomphaliones, representative images. Live specimens are depicted unless otherwise specified **A**Cocculinidae fam. inc. (M16816, dorsal view) **B**Cocculinidae fam. inc. (M16816, ventral view) **C***Leptogyropsis* gen. inc. (M19102, same preserved specimen in apical, umbilical, and apertural view) **D***Helicrenion* stet. (M18909, two preserved specimens) **E**Neomphalidae sp. SIO_BIC_M14645 (M14645, two specimens) **F**Neomphalidae sp. SIO_BIC_M14645 (M14645, ventral view) **G**Peltospiridae stet. (M16802, dorsal view) **H**Peltospiridae stet. (M16802, ventral view). Scale bars: 1 mm.

**Material examined.** AD4972: M16726, M16727, M16728; AD4974: M16789, M16790; AD4976: M16816, M16817.

**Localities.** Mound 12 (~ 1000 m), Jacó Scar (~ 1800 m).

**Remarks.** Associated with experimentally deployed wood. Under morphological and genetic investigation (Betters et al. (2024), in press at the time of this work’s acceptance).

#### ﻿Mollusca | Gastropoda | Neomphaliones | Neomphalida | Melanodrymiidae

***Leptogyropsis* gen. inc.**

Fig. 39C

**Material examined.** AD4587: M19102, M19146.

**Localities.** Mound 12 (996 m).

**Remarks.** Associated with a naturally occurring wood fall.

#### ﻿Mollusca | Gastropoda | Neomphaliones | Neomphalida | Neomphalidae

***Helicrenion* stet.**

Fig. 39D

**Material examined.** AD4508: M12027; AD4586: M18782; AD4587: M18993, M18999, M19004; AD4589: M18818; AD4590: M18909; AD4988: M16962.

**Localities.** Mound 11 (1009 m), Mound 12 (~ 1000 m), Parrita Seep (~ 1400 m), Jacó Scar (~ 1800 m).

**Remarks.** Possibly an undescribed species. Several specimens (M16962, M18993, M18999, and M19004) were collected from microbial mat habitats.

**Neomphalidae sp. SIO_BIC_M14645**

Fig. 39E, F

**Material examined.** AD4910: M14645; AD4917: M14646 (**PQ449412**).

**Localities.** Mound 12 (~ 1000 m).

**Remarks.** An undescribed genus and species.

#### ﻿Mollusca | Gastropoda | Neomphaliones | Neomphalida | Peltospiridae

**Peltospiridae stet.**

Fig. 39G, H

**Material examined.** AD4976: M16802.

**Localities.** Jacó Scar (1887 m).

**Remarks.** Associated with experimentally deployed wood.

#### ﻿Mollusca | Scaphopoda

**Gadilida stet.**

Fig. 40A

**Figure 40.**
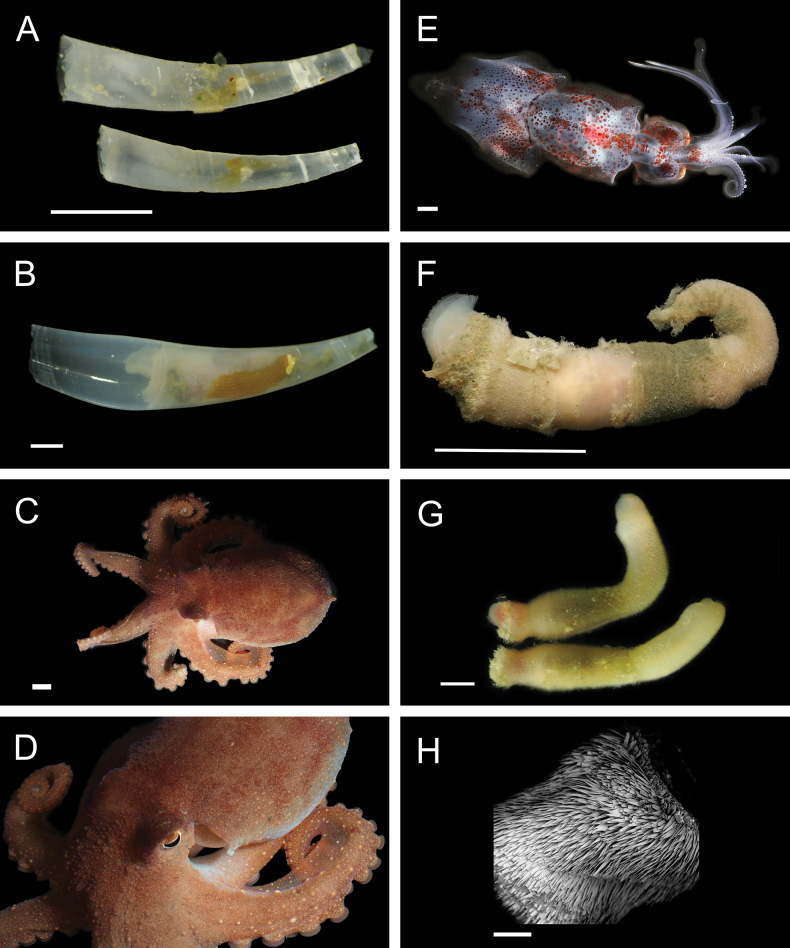
Mollusca: Scaphopoda, Cephalopoda, and Aplacophora: Caudofoveata, representative live and SEM images **A***Siphonodentalium* gen. inc. (M12048) **B**Gadilida stet. (M12049) **C**Octopodoidea stet. (M17037, dorsal view) **D**Octopodoidea stet. (M17037, detail) **E***Planctoteuthisdanae* (M17039) **F***Chaetoderma* stet. (M16155, incomplete specimen, cuticle missing from part of the body) **G**Chaetodermatidae sp. SIO_BIC_M12018 (M12018) **H**Chaetodermatidae sp. SIO_BIC_M12018 (M12019, SEM). Scale bars: 1 mm (**A, B, G**); 1 cm (**C, E, F**); 0.3 mm (**H**).

**Material examined.** AD4510: M12049.

**Localities.** Jacó Summit (744 m).

***Siphonodentalium* gen. inc.**

Fig. 40B

**Material examined.** AD4510: M12048; AD4511: M12056.

**Localities.** Jacó Summit (742 m), Mound 12 (~ 988–997 m).

#### ﻿Mollusca | Cephalopoda

We thank Michael Vecchione (National Marine Fisheries Service National Systematics Laboratory, U.S. National Museum of Natural History) for assistance with these identifications.

#### ﻿Mollusca | Cephalopoda | Octopoda

**Octopodoidea sp. SIO_BIC_ M17037**

Fig. 40C, D

**Material examined.** S0216: M17037.

**Localities.** Quepos Slide (317 m) .

**Remarks.** Likely an undescribed species, possibly an undescribed genus. The animal was observed releasing ink (potentially a diagnostic character).

#### ﻿Mollusca | Cephalopoda | Oegopsida

***Planctoteuthisdanae* (Joubin, 1931)**

Fig. 40E

**Material examined.** S0215: M17039.

**Localities.** Mound 12 (1016 m depth, 2–3 m above the seafloor).

**Distribution.** Originally described from the Gulf of Panama and considered cosmopolitan in tropical and temperate waters worldwide (Jereb and Roper 2010).

#### ﻿Mollusca | Caudofoveata

We use the clade-based high-level taxonomic names in Kocot et al. (2019).

***Chaetoderma* stet.**

Fig. 40F

**Material examined.** AD4917: M16155; AD4977: M16809.

**Localities.** Jacó Scar (1783–1791 m).

**Chaetodermatidae sp. SIO_BIC_M12018**

Fig. 40G, H

**Material examined.** AD4508: M12018 (**PQ449402**), M12019; AD4972: BI1338 (**PQ449340**).

**Localities.** Parrita Seep (~ 1401–1419 m), Jacó Scar (1746 m).

**Remarks.** The closest COI BLASTN result on GenBank was the holotype of *Chaetodermafelderi* Ivanov & Scheltema, 2007 (AM922259.1; 93.33% identity to M12018, 93.18% identity to BI1338). Based on COI, these specimens belong within Chaetodermatidae and may belong to *Chaetoderma* or *Falcidens*, but these genera are not reciprocally monophyletic (Mikkelsen et al. 2019).

**Chaetodermatidae sp. SIO_BIC_M16812**

**Material examined.** AD4975: M16812 (**PQ449416**; no image available).

**Localities.** Mound 12 (1000 m).

**Remarks.** The closest COI BLASTN result on GenBank was the holotype of *Chaetodermafelderi* (AM922259.1; 90.21% identity). As above, this specimen may belong to *Chaetoderma* or *Falcidens*.

**Chaetodermatidae sp. SIO_BIC_M16891**

Fig. 41A

**Figure 41.**
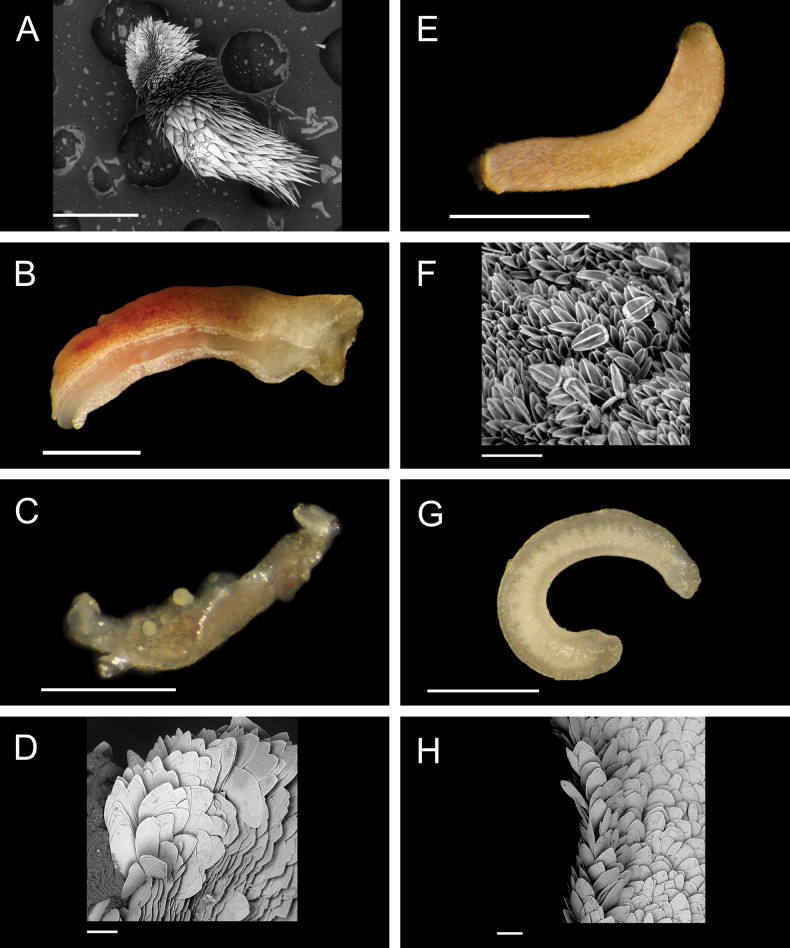
Mollusca: Aplacophora: Caudofoveata and Solenogastres, representative live and SEM images **A**Chaetodermatidae sp. SIO_BIC_M16891 (M16891, SEM) **B***Neomenia* gen. inc. (M18409) **C**Gymnomeniidae stet. (M16924) **D**Gymnomeniidae stet. (M16924, SEM) **E***Wirenia* sp. SIO_BIC_M17072 (M17072) **F***Wirenia* sp. SIO_BIC_M17072 (M17072, SEM) **G**Pholidoskepia stet. (M16923) **H**Pholidoskepia stet. (M16923, SEM). Scale bars: 0.3 mm (**A**); 1 mm (**B, C, E, G**); 0.02 mm (**D, F, H**).

**Material examined.** AD4979: M16891 (**PQ435556**).

**Localities.** Quepos Slide (397 m).

**Remarks.** The closest COI BLASTN result on GenBank was an undescribed species of *Falcidens* (MG855756.1; 93.93% identity). As above, this specimen may belong to *Chaetoderma* or *Falcidens*.

#### ﻿Mollusca | Solenogastres

We use the clade-based high-level taxonomic names in Kocot et al. (2019). Groups that have been historically assigned to the non-monophyletic “Cavibelonia” are listed last.

#### ﻿Mollusca | Solenogastres | ﻿Neomeniamorpha | Neomeniidae

***Neomenia* gen. inc.**

Fig. 41B

**Material examined.** S0219: M18409.

**Localities.** Rio Bongo Scar (606 m).

#### ﻿Mollusca | Solenogastres | Pholidoskepia | Gymnomeniidae

**Gymnomeniidae stet.**

Fig. 41C, D

**Material examined.** AD4990: M16924 (**PQ435558**; 16S: **PQ304664**).

**Localities.** Parrita Seep (1401 m).

***Wirenia* sp. SIO_BIC_M17072**

Fig. 41E, F

**Material examined.** S0219: M17072 (**PQ435553**; 16S: **PQ304665**).

**Localities.** Rio Bongo Scar (606 m).

**Remarks.** This undescribed species has keeled, leaf-like sclerites typical of *Wirenia*, but it is easily distinguished from all described species by the extremely small size (< 200 µm) of the sclerites.

#### ﻿Mollusca | Solenogastres | Pholidoskepia

**Pholidoskepia stet.**

Fig. 41G, H

**Material examined.** AD4990: M16923 (**PQ435557**; 16S: **PQ304663**).

**Localities.** Parrita Seep (1401 m).

**Remarks.** Genetic data place this specimen within Pholidoskepia*sensu*Kocot et al. 2019. It is possibly a member of Sandalomeniidae, pending further molecular characterization of this group.

#### ﻿Mollusca | Solenogastres | Amphimeniidae

**Amphimeniidae stet.**

Fig. 42A

**Figure 42.**
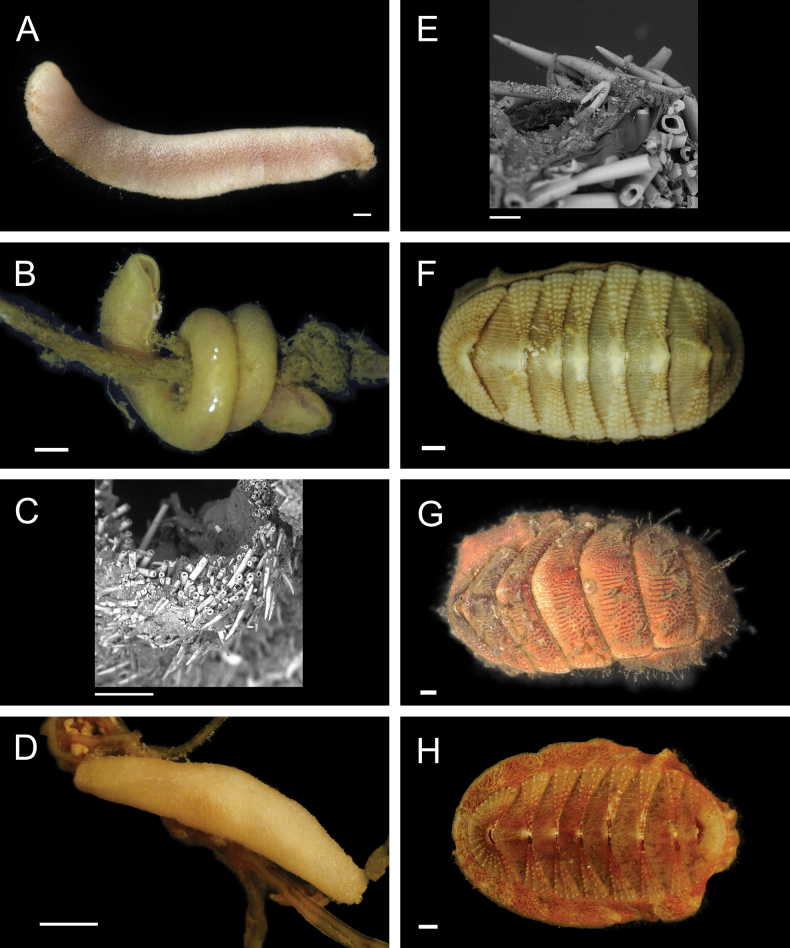
Mollusca: Aplacophora: Solenogastres and Polyplacophora, representative live and SEM images **A**Amphimeniidae stet. (M12292) **B***Dorymenia* stet. (M11997) **C***Dorymenia* stet. (M16156, SEM) **D**Pruvotinidae stet. (M16880) **E**Pruvotinidae stet. (M16885, SEM) **F***Stenosemus* sp. SIO_BIC_M12017 (M12017) **G***Stenosemus* sp. SIO_BIC_M17044 (M17044) **H***Tripoplaxbalaenophila* (M16186). Scale bars: 1 mm (**A, B, D, F–H**); 0.1 mm (**C**); 0.02 mm (**E**).

**Material examined.** AD4587: M12292 (**PQ449404**).

**Localities.** Mound 12 (996 m).

#### ﻿Mollusca | Solenogastres | Proneomeniidae

***Dorymenia* stet.**

Fig. 42B, C

**Material examined.** AD4505: M11997; AD4917: M16156 (**PQ435554**; 16S: **PQ304662**).

**Localities.** Mound 12 (1002 m), Mound 11 (~ 1019–1025 m).

**Remarks.** M11997 and M16156 are the same morphospecies. M16156 was associated with the octocoral *Swiftiasahlingi* (Co2935).

#### ﻿Mollusca | Solenogastres | Pruvotinidae

**Pruvotinidae stet.**

Fig. 42D, E

**Material examined.** AD4978: M16880, M16885 (**PQ435555**).

**Localities.** Mound 12 (997 m).

**Remarks.** M16880 was associated with a hydroid (Co3639).

#### ﻿Mollusca | Polyplacophora | Chitonida | Ischnochitonidae

***Stenosemus* sp. SIO_BIC_M12017**

Fig. 42F

**Material examined.** AD4508: M12017 (16S: **PQ304661**).

**Localities.** Parrita Seep (1402 m).

**Remarks.** An undescribed species.

***Stenosemus* sp. SIO_BIC_M17044**

Fig. 42G

**Material examined.** S0218: M17044 (**PQ449420**).

**Localities.** Parrita Scar (1364 m).

**Remarks.** An undescribed species.

***Tripoplaxbalaenophila* (Schwabe & Sellanes, 2004)**

Fig. 42H

**Material examined.** AD4512: M12064 (**PQ450384**; 16S: **PQ304667**); AD4918: M16186.

**Localities.** Quepos Slide (338 m and ~ 344–411 m).

**Distribution.** Originally described from whale bones at 240 m, within the oxygen minimum zone, off Concepción, central Chile (36°29.9'S, 73°40.8'W) (Schwabe and Sellanes 2004). Specimens identified as Tripoplaxcf.balaenophila have been collected off central Baja California, 530–625 m, in hypoxic conditions near the lower boundary of the oxygen minimum zone (Suárez-Mozo and Hendrickx 2016). Those specimens show morphological variations from the *T.balaenophila* type material and require further taxonomic investigation, including genetic work, to determine whether they represent a range extension of *T.balaenophila* or a very similar undescribed species (Suárez-Mozo and Hendrickx 2016).

**New records.** Pending confirmation of the specimens from Mexico, our CRM specimens represent new northern records, new seep records, and a new maximum depth record for this species (specimen M12064, using 344 m as the most conservative value).

**Remarks.** Consistent with the previous reports of this species in hypoxic conditions, the CRM seep specimens were also collected within the oxygen minimum zone, although not in association with organic falls.

#### ﻿Mollusca | Polyplacophora | Lepidopleurida | Leptochitonidae

*Leptochiton* is paraphyletic and molecular taxonomic revision is needed (Irisarri et al. 2020).

***Belknapchitonhalistreptus* (Dall, 1902)**

Fig. 43A

**Figure 43.**
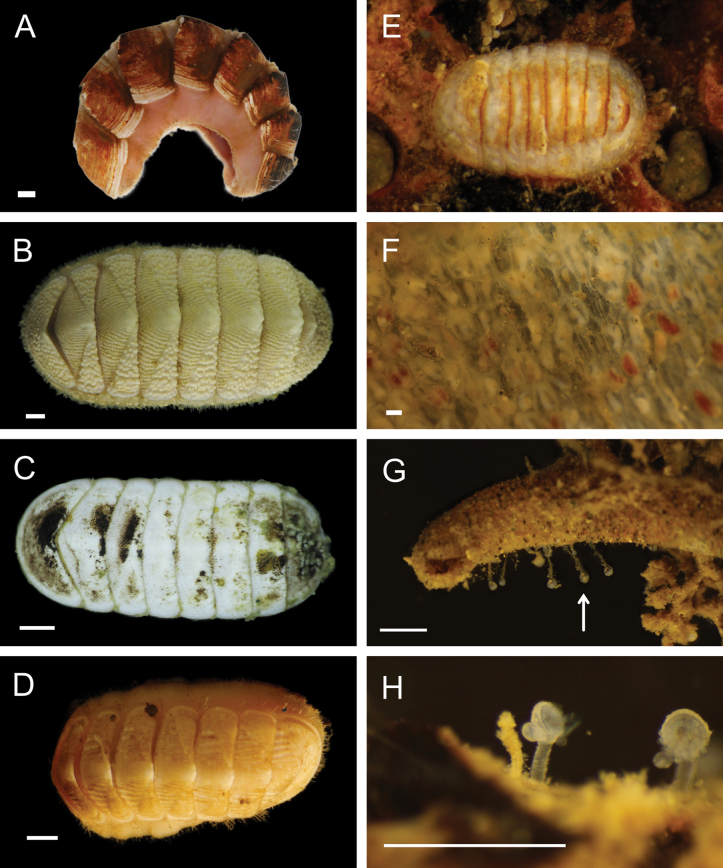
Mollusca: Polyplacophora, Bryozoa, and Entoprocta, representative live images **A***Belknapchitonhalistreptus* (M17045) **B***Hanleyella* sp. SIO_BIC_M11969 (M11969) **C**Leptochitoncf.americanus (M12016) **D**Leptochitoncf.incongruus (M17067) **E***Leptochiton* sp. SIO_BIC_M17068 (M17068; specimen length estimated < 3 mm, maximum 6 mm) **F**Bryozoa stet. (Ep220) **G**Entoprocta stet. (BI1162) **H**Entoprocta stet. (BI1166, detail). Scale bars: 1 mm.

**Material examined.** S0219: M17045 (**PQ449421**).

**Localities.** Rio Bongo Scar (609 m).

**Distribution.** Known only from the type locality off Acapulco, Mexico, 902–3436 m (Dall 1902, 1908; Sirenko et al. 2022). Two subspecies are distinguished by morphology and depth: *B.halistreptushalistreptus* from ~ 3400 m and *B.halistreptusabbreviatus* from ~ 900–1200 m (Dall 1908; Sirenko et al. 2022).

**New records.** The CRM specimen represents a new southern record and a new minimum depth record for this species.

**Remarks.***B.halistreptus* is a member of the recently described genus *Belknapchiton* Sirenko, Saito & Schwabe, 2022. This genus of 22 species includes many of the worldwide chiton specimens obtained from deep water that were formally assigned to *Leptochiton*, and these generally require SEM observations to identify to species. The type species, *B.belknapi* (Dall, 1878), was described from 1840 m off the western Aleutian Islands and is widespread at depths of 100–3724 m in the Pacific, from the Izu-Ogasawara Trench through the Bering Sea and eastern Pacific as far south as central Chile (Sirenko and Sellanes 2016; Sirenko et al. 2022). The CRM specimen has been tentatively identified as one of two similar subspecies of *B.halistreptus*. Both subspecies can be distinguished from *B.belknapi* and other congeners because they have ~ 2× as many gills as *B.belknapi* and a gill row that extends further anterior to approximately the position of the fifth valve (Sirenko et al. 2022).

***Hanleyella* sp. SIO_BIC_M11969**

Fig. 43B

**Material examined.** AD4501: M11969 (16S: **PQ304660**); AD4508: M12015; AD4588: M12131; AD4974: M16766, M16767; AD4978: M16841, M16842, M16883, M16884; AD4987: M16905.

**Localities.** Mound 12 (~ 1000 m), Parrita Seep (1402 m).

**Remarks.** These specimens represent an undescribed species with morphological and genetic similarities to *H.oldroydi* (Dall, 1919), which is found from Alaska to Baja California at depths of 18–455 m (Stebbins and Eernisse 2009).

**Leptochitoncf.americanus Kaas & Van Belle, 1985**

Fig. 43C

**Material examined.** AD4508: M12016; AD4973: M16725; AD4976: M16814; S0230: M17101, M17104, M17107 (**PQ449425**).

**Localities.** Parrita Seep (1419 m), Jacó Scar (1887 m), Mound Jaguar (1896–2000 m).

**Remarks.** All specimens except M16725 were associated with naturally occurring or experimentally deployed wood. These specimens may represent an undescribed species or new depth records of *L.americanus*, which was originally described from the Gulf of Panama, 1188 m, and is known from Oregon to Chile, 311–1400 m (Schwabe and Sellanes 2010).

**Leptochitoncf.incongruus (Dall, 1908)**

Fig. 43D

**Material examined.** S0219: M17067 (**PQ449422**).

**Localities.** Rio Bongo Scar (661 m).

**Remarks.** Associated with a naturally occurring wood fall. Possibly an undescribed species or a juvenile of *L.incongruus*, which was described from the Gulf of Panama, 589 m (Dall 1908).

***Leptochiton* sp. SIO_BIC_M17068**

Fig. 43E

**Material examined.** S0219: M17068 (**PQ449423**; no voucher remaining after DNA extraction).

**Localities.** Rio Bongo Scar (661 m).

**Remarks.** An undescribed species associated with a naturally occurring wood fall.

#### ﻿﻿Bryozoa

**Bryozoa stet.**

Fig. 43F

**Material examined.** AD4591: Ep245; AD4924: Ep220.

**Localities.** Parrita Seep (~ 1400–1410 m), Jacó Scar (~ 1752–1795 m).

**Remarks.** Encrusting on vesicomyid clams.

#### ﻿﻿Entoprocta

**Entoprocta stet.**

Fig. 43G, H

**Material examined.** AD4919: BI1162; AD4921: BI1166.

**Localities.** Quepos Slide (~ 345–397 m).

**Remarks.** BI1162 was associated with the tube of a sabellid worm, *Pseudopotamilla* stet. (A8390). BI1166 was associated with a naturally occurring wood fall.

#### ﻿﻿Platyhelminthes

**Fecampiida stet.**

Fig. 44A, B

**Figure 44.**
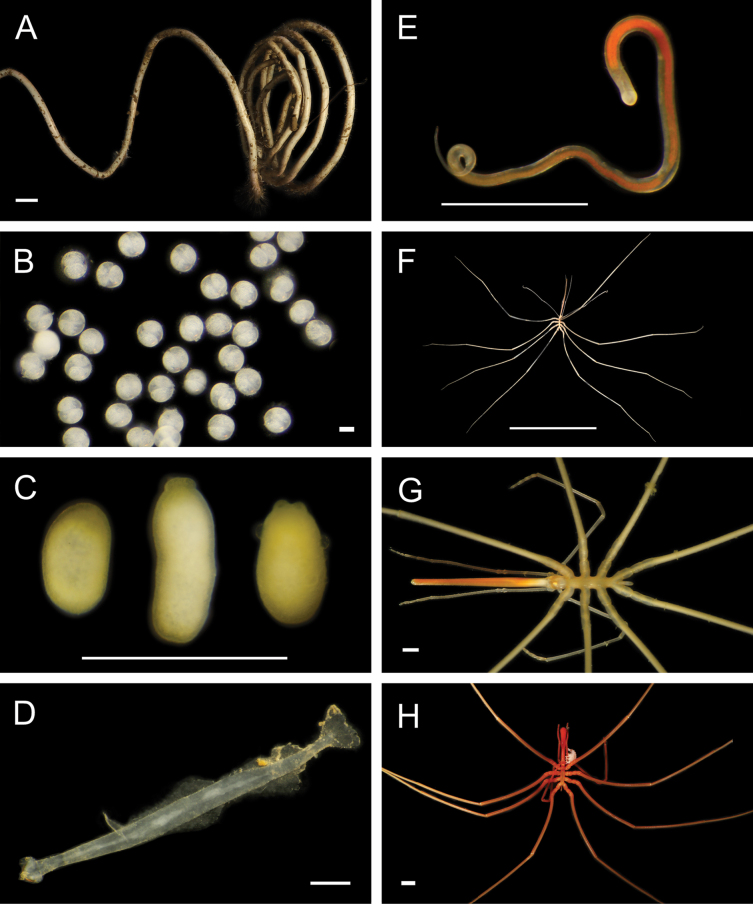
Platyhelminthes, Chaetognatha, Nematoda, and Arthropoda: Pycnogonida, representative live images **A** Fecampiida stet. (Pt64, egg cocoon) **B** Fecampiida stet. (Pt64, detail of eggs) **C**Rhabditophora stet. (Pt72) **D**Chaetognatha stet. (BI1347) **E**Nematoda stet. (Nto35) **F***Colossendeismacerrima* (C12792, wide view) **G***Colossendeismacerrima* (C11151, detail) **H***Colossendeis* stet. (C13919, wide view). Scale bars: 1 cm (**A, H**); 0.1 mm (**B**); 1 mm (**C–E, G**); 10 cm (**F**).

**Material examined.** AD4916: Pt64.

**Localities.** Jacó Scar (1854 m).

**Remarks.** This coiled cocoon of egg capsules was found on soft sediment. Fecampiid cocoons have been previously reported in association with gorgonians in the western Pacific, 92–295 m (Handl and Bouchet 2007).

**Rhabditophora stet.**

Fig. 44C

**Material examined.** AD4978: Pt66; AD4985: Pt68; AD4989: Pt72, Pt73.

**Localities.** Mound 12 (~ 995–1002 m), Jacó Scar (1768 m).

**Remarks.** Pt72 and Pt73 were associated with a tubeworm bush.

#### ﻿﻿Chaetognatha

**Chaetognatha stet.**

Fig. 44D

**Material examined.** AD4985: BI1347.

**Localities.** Mound 12 (991 m).

#### ﻿﻿Nematoda

**Nematoda stet.**

Fig. 44E

**Material examined.** AD4918: Nto35; AD4979: Nto64, Nto65, Nto66, Nto67; S0216: Nto68.

**Localities.** Quepos Slide (~ 275–400 m).

#### ﻿﻿Arthropoda

We list the major arthropod clades according to the phylogenetic relationships in Regier et al. (2010). See Azofeifa-Solano and Cortés (2020) for a detailed review of deep-sea crustaceans recorded from Costa Rican waters.

Many of the copepod, barnacle, and peracarid morphospecies in this study were represented by small single specimens and may represent undescribed species. To minimize the destruction of diagnostic features, we did not attempt extensive genetic investigation. Specimens are available for loan for future examination.

#### ﻿Arthropoda | Chelicerata | Pycnogonida

We list entries following the phylogeny in Ballesteros et al. (2021). We thank Claudia Arango (Queensland Museum) for assistance with morphological identification of these specimens.

#### ﻿Arthropoda | Chelicerata | Pycnogonida | Colossendeidae

***Colossendeismacerrima* Wilson, 1881**

Fig. 44F, G

**Material examined.** AD4509: C11151; AD4914: C12792 (**PQ449344**).

**Localities.** Jacó Scar (~ 974–1856 m).

**Distribution.** Considered cosmopolitan (Munilla and Soler Membrives 2009), originally described from the United States mid-Atlantic coast, 1686 m (Wilson 1881), and previously reported from the Pacific coast of Central America (Hendrickx 2020b).

**Remarks.** The COI sequence of C12792 was 93.19–95.94% identical to sequences of *Colossendeismacerrima* (KF603929.1, KF603928.1, JN018213.1, FJ862873.1), with the closest BLASTN matches corresponding to specimens from southern Chile, 510 m (Weis and Melzer 2012). We interpret this 4–7% divergence as intraspecific variation, based on a previous analysis of *C.macerrima* and nine other Chilean pycnogonid species, in which the maximum intraspecific COI divergence was 10.4% and the minimum interspecific COI divergence was 13.36% (Weis and Melzer 2012).

***Colossendeis* stet.**

Figs 44H, 45A

**Figure 45.**
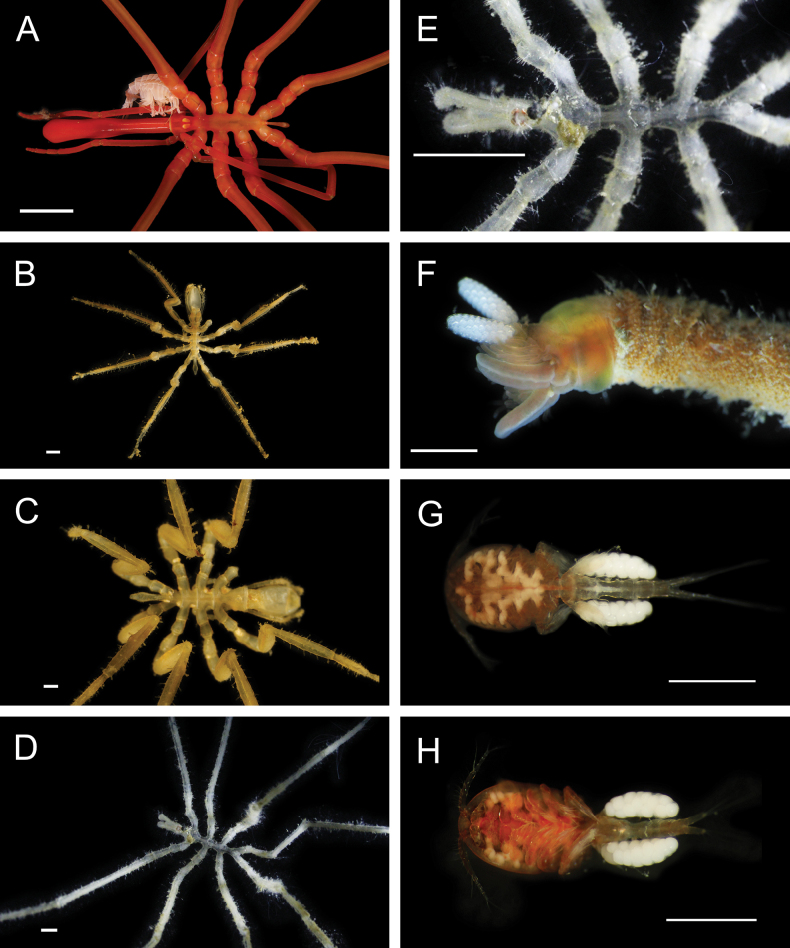
Arthropoda: Pycnogonida and Copepoda, representative live images **A***Colossendeis* stet. (C13919, detail) **B***Sericosura* sp. SIO_BIC_C13774 (C13774) **C***Sericosura* sp. SIO_BIC_C13775 (C13775) **D***Anoplodactylus* gen. inc. (C13825, wide view) **E***Anoplodactylus* gen. inc. (C13825, detail) **F**Bradophilidae stet. (A1451) **G**Cyclopoida sp. SIO_BIC_C12780 (C12780, dorsal view) **H**Cyclopoida sp. SIO_BIC_C12780 (C12780, ventral view). Scale bars: 1 cm (**A**); 1 mm (**B–H**).

**Material examined.** S0218: C13919 (**PQ449356**).

**Localities.** Parrita Scar (1153 m).

**Remarks.** An amphipod, *Mesopleustesabyssorum* (C13920), was attached to the palp of this specimen. The closest COI BLASTN results on GenBank were several species of *Colossendeis* with ~ 88% identity, e.g., *C.colossea* Wilson, 1881 (FJ716626.1, formerly *C.gigas* Hoek, 1881), *C.australis* Hodgson, 1907 (GQ387003.1), *C.tortipalpis* Gordon, 1932 (KT202204.1), and *C.macerrima* (JN018213.1). This level of COI divergence falls between the intraspecific (<10.4%) and interspecific (>13.36%) divergences reported for other pycnogonids (Weis and Melzer 2012), so species-level identification of the CRM specimen will require further investigation.

#### ﻿Arthropoda | Chelicerata | Pycnogonida | Ammotheidae

***Sericosura* sp. SIO_BIC_C13774**

Fig. 45B

**Material examined.** AD4972: C13774 (**PQ449350**).

**Localities.** Jacó Scar (1795 m).

**Remarks.** An undescribed species.

***Sericosura* sp. SIO_BIC_C13775**

Fig. 45C

**Material examined.** AD4972: C13775 (**PQ449351**); AD4989: C13865.

**Localities.** Jacó Scar (1785–1795 m).

**Remarks.** An undescribed species.

#### ﻿Arthropoda | Chelicerata | Pycnogonida | Phoxichilidiidae

***Anoplodactylus* gen. inc.**

Fig. 45D, E

**Material examined.** AD4978: C13793 (**PQ449353**); AD4985: C13825.

**Localities.** Mound 12 (~ 996–1002 m).

**Remarks.** Most likely *Anoplodactylus*, perhaps an undescribed species (Claudia Arango, pers. comm. 24 July 2022).

#### ﻿Arthropoda | Crustacea | Copepoda

We thank Linsey Sala (Scripps Institution of Oceanography Pelagic Invertebrate Collection) for assistance with these identifications.

#### ﻿Arthropoda | Crustacea | Copepoda | Cyclopoida

**Bradophilidae stet.**

Fig. 45F

**Material examined.** AD4511: C14958 (no material remaining); AD4987: C14494.

**Localities.** Mound 12 (~ 988–1012 m).

**Remarks.** Egg masses were attached to the flabelligerid Bradabyssacf.pilosa: C14958 on host A1451 and C14494 on host A9840.

**Cyclopoida sp. SIO_BIC_C12780**

Fig. 45G, H

**Material examined.** AD4503: C11138; AD4587: C11184; AD4910: C12780 (**PQ449343**).

**Localities.** Mound 12 (~ 1000 m).

**Remarks.** Found in the mantle cavity of the solemyid clam Acharaxcf.johnsoni: C11138 with clam M11980, C12780 with clam M15768.

**Cyclopoida sp. SIO_BIC_C12807**

Fig. 46A

**Figure 46.**
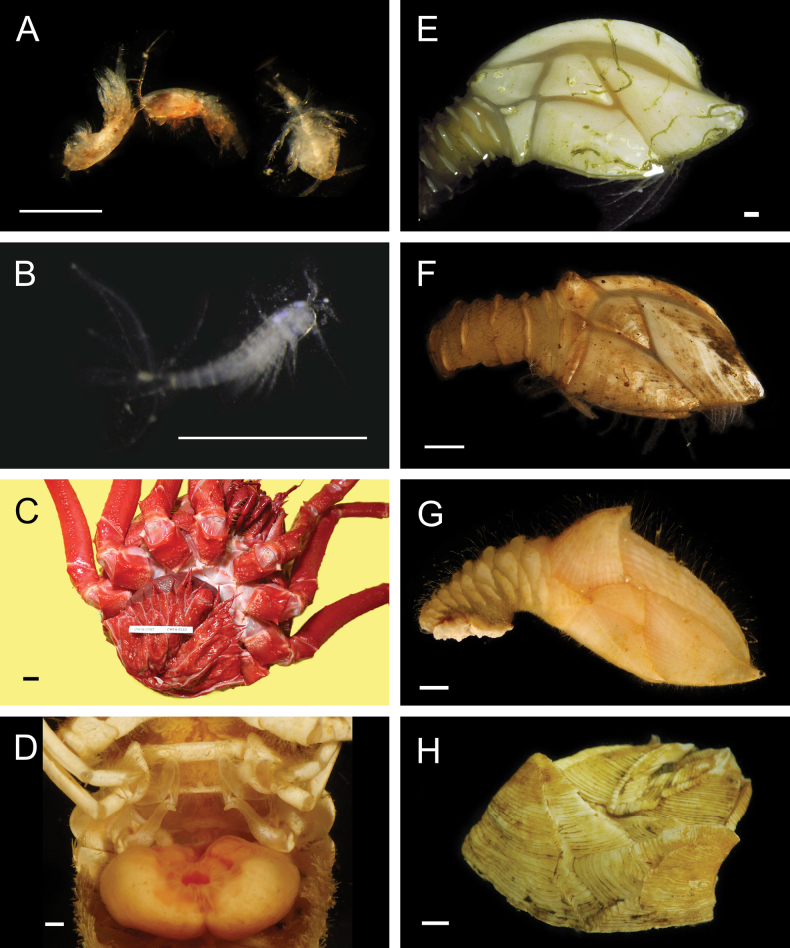
Arthropoda: Copepoda and Cirripedia, representative live images **A**Cyclopoida sp. SIO_BIC_C12807 (C12807) **B**Harpacticoida stet. (C13870) **C**Rhizocephala sp. SIO_BIC_C13776 (C13776) **D**Rhizocephala sp. SIO_BIC_C13875 (C13875) **E***Litoscalpellum* sp. SIO_BIC_C11143 (C11143) **F***Litoscalpellum* sp. SIO_BIC_C12782 (C12782) **G**Scalpellidae sp. SIO_BIC_C13851 (C13851) **H***Metaverruca* gen. inc. (C11148). Scale bars: 1 mm (**A, B, D, E, G, H**); 1 cm (**C, F**).

**Material examined.** AD4918: C12807.

**Localities.** Quepos Slide (~ 333–408 m).

#### ﻿Arthropoda | Crustacea | Copepoda | Harpacticoida

**Harpacticoida stet.**

Fig. 46B

**Material examined.** AD4988: C13870.

**Localities.** Mound 11 (~ 1005–1025 m).

#### ﻿Arthropoda | Crustacea | Copepoda | Siphonostomatoida

***Caligus* stet.**

**Material examined.** AD4503: MZUCR-2770-01 (no images available); AD4505: MZUCR-2771-01 (no images available).

**Localities.** Mound 11 (~ 1020 m), Mound 12 (~ 1000 m).

#### ﻿Arthropoda | Crustacea | Thecostraca | Cirripedia

We list entries following the phylogeny in Chan et al. (2021). We thank Hiromi Watanabe (Japan Agency for Marine-Earth Science and Technology) for the morphology-based identifications.

#### ﻿Arthropoda | Crustacea | Thecostraca | Cirripedia | Rhizocephala

**Rhizocephala sp. SIO_BIC_C13776**

Fig. 46C

**Material examined.** AD4975: C13776 (**PQ448998**).

**Localities.** Mound 12 (1000 m).

**Remarks.** Parasite of a lithodid crab, *Lithodespanamensis* (C13787). The closest COI BLASTN results on GenBank were within Peltogastridae: *Briarosaccus* sp. (OR466125.1, 84.23% identity), *Peltogasterboschmai* Reinhard, 1944 from the San Juan Islands, Washington, USA (MN138416.1, 80.00% identity), and several sequences of *P.lineata* Shiino, 1943 from Korea and Japan (e.g., MK604142.1, 78.82% identity).

**Rhizocephala sp. SIO_BIC_C13875**

Fig. 46D

**Material examined.** AD4989: C13875 (**PQ449355**).

**Localities.** Jacó Scar (1762 m).

**Remarks.** Parasite of a squat lobster, *Munidopsisalvisca* (C13876). The closest COI BLASTN results on GenBank were several species of *Lernaeodiscus* (Peltogastridae), e.g., *L.ingolfi* Boschma, 1928 from Norway (MN605966.1, 80.62% identity) and *L.rybakovi* Korn, Golubinskaya, Rees, Glenner & Høeg, 2020 from Vostok Bay, Russia (MN605964.1, 78.81% identity).

#### ﻿Arthropoda | Crustacea | Thecostraca | Cirripedia | Thoracica | Scalpellomorpha

***Litoscalpellum* sp. SIO_BIC_C11143**

Fig. 46E

**Material examined.** AD4504: C11143.

**Localities.** Mound 11 (~ 1004–1011 m).

**Remarks.** Attached to a vestimentiferan tubeworm. *Litoscalpellum* is polyphyletic and revision is required (Linse et al. 2013).

***Litoscalpellum* sp. SIO_BIC_C12782**

Fig. 46F

**Material examined.** AD4913: C12782.

**Localities.** Jacó Scar (1885 m).

**Scalpellidae sp. SIO_BIC_C13851**

Fig. 46G

**Material examined.** AD4990: C13851.

**Localities.** Parrita Seep (1401 m).

#### ﻿Arthropoda | Crustacea | Thecostraca | Cirripedia | Thoracica | Verrucomorpha

***Metaverruca* gen. inc.**

Fig. 46H

**Material examined.** AD4508: C11148.

**Localities.** Parrita Seep (~ 1401–1419 m).

**Remarks.** Likely *Metaverruca*. Attached to a tubeworm, *Lamellibrachiabarhami*.

***Newmaniverruca* gen. inc.**

Fig. 47A

**Figure 47.**
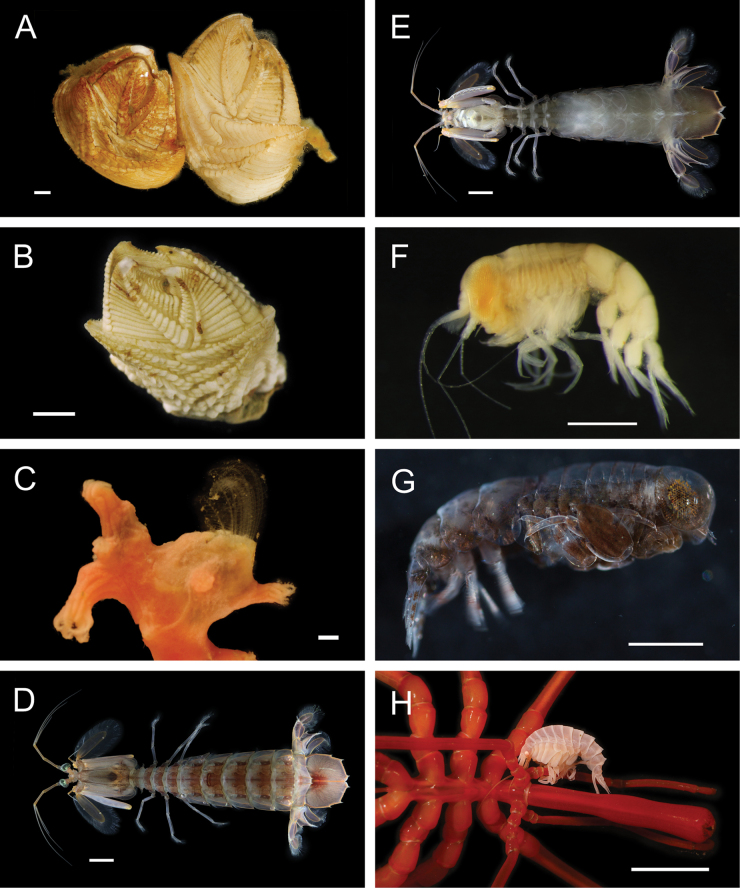
Arthropoda: Cirripedia, Stomatopoda, and Amphipoda, representative images. Live specimens are depicted unless otherwise specified **A***Newmaniverruca* gen. inc. (C12794) **B**Verrucidae sp. SIO_BIC_C11144 (C11144) **C**Pyrgomatidae stet. (C12815) **D***Squillabiformis* (C13807, dorsal view) **E***Squillabiformis* (C13807, ventral view) **F**Hyperiidea stet. (C15409, preserved specimen) **G***Lycaeapulex* (C14397) **H***Mesopleustesabyssorum* (C13920). Scale bars: 1 mm (**A–C, F, G**); 1 cm (**D, E, H**).

**Material examined.** AD4916: C12794.

**Localities.** Jacó Scar (1611 m).

**Remarks.** Likely *Newmaniverruca*.

**Verrucidae sp. SIO_BIC_C11144**

Fig. 47B

**Material examined.** AD4506: C11144.

**Localities.** Parrita Seep (~ 1030–1179 m).

**Remarks.** Likely *Altiverruca* or *Newmaniverruca*.

#### ﻿Arthropoda | Crustacea | Thecostraca | Cirripedia | Thoracica | Balanomorpha

**Pyrgomatidae stet.**

Fig. 47C

**Material examined.** AD4923: C12815.

**Localities.** Parrita Seep (~ 1041–1094 m).

**Remarks.** Associated with a coralliid (Co2947).

#### ﻿Arthropoda | Crustacea | Malacostraca | Hoplocarida | Stomatopoda | Squillidae

***Squillabiformis* Bigelow, 1891**

Fig. 47D, E

**Reference.**Koga and Rouse (2021) for the mitochondrial genome and phylogenetic analysis of C13808.

**Material examined.** AD4979: C13807 (**PQ449354**), C13808 (MW867305); AD4986: MZUCR-3732-01.

**Localities.** Quepos Slide (~ 380–395 m).

**Distribution.** Originally described from La Paz, Gulf of California, at 205 m (Bigelow 1894) and distributed south to Peru at depths of 28–518 m (Hendrickx and Salgado-Barragán 1991; Hendrickx and López 2020). *S.biformis* has been studied from Pacific Costa Rica at depths of 131–350 m (Camp and Kuck 1990; Wehrtmann and Echeverría-Sáenz 2007; Hernáez et al. 2011; Azofeifa-Solano and Cortés 2020).

**Remarks.** Telson morphology indicates that both specimens are female. To our knowledge this study is the first report of this well-known local species in the vicinity of seeps, notably within the oxygen minimum zone as characterized by Levin et al. (2015).

#### ﻿Arthropoda | Crustacea | Malacostraca | Peracarida

Several available checklists of eastern Pacific deep-sea peracarids (Hendrickx 2020a) may be informative for future work on these specimens. Unless otherwise stated, we list entries following the phylogenies in Schwentner et al. (2018) and Höpel et al. (2022).

#### ﻿Arthropoda | Crustacea | Malacostraca | Peracarida | Lophogastrida

***Eucopiasculpticauda* Faxon, 1893**

**Material examined.** AD4513: MZUCR-2818-01 (no image available).

**Localities.** Jacó Scar (~ 1800 m).

**Distribution.** Originally described from several stations in the Gulf of Panama and off the Galápagos Islands, 1618–2487 m (Faxon 1893, 1895). Reported with a wide latitudinal distribution in the Indo-Pacific and Atlantic Oceans, from ~ 1000–7526 m depth (Kou et al. 2019).

#### ﻿Arthropoda | Crustacea | Malacostraca | Peracarida | Amphipoda

We list entries following the World Amphipoda Database (Horton et al. 2022), acknowledging the need for further systematic work as discussed in the molecular phylogeny of Copilaş-Ciocianu et al. (2020).

#### ﻿Arthropoda | … | Amphipoda | Hyperiidea

We thank Linsey Sala for these identifications.

**Hyperiidea stet.**

Fig. 47F

**Material examined.** AT15-59 Plankton Tow 6: C15409.

**Localities.** Jacó Summit (~ 350 m depth, ~ 400 m above the seafloor).

***Lycaeapulex* Marion, 1874**

Fig. 47G

**Material examined.** AT15-59 Plankton Tow 6: C14397.

**Localities.** Jacó Summit (~ 350 m depth, ~ 400 m above the seafloor).

**Distribution.** Considered common and widespread in tropical to warm-temperate oceans worldwide, typically 0–500 m depth (Zeidler 2021).

#### ﻿Arthropoda | … | Amphipoda | Amphilochidea | Amphilochida | Amphilochidira | Amphilochoidea | Pleustidae

***Mesopleustesabyssorum* (Stebbing, 1888)**

Fig. 47H

**Material examined.** S0218: C13920.

**Localities.** Parrita Scar (1153 m).

**Distribution.** Originally described from the subantarctic Indian Ocean off South Africa, 2926 m (Stebbing 1888), *M.abyssorum* is considered “probably cosmopolitan” at depths below ~ 700 m (Barnard 1967). In the eastern Pacific, *M.abyssorum* has been reported from 3479 m off Baja California (Barnard 1967).

**Remarks.** Observed *in situ* attached to the palp of a pycnogonid, *Colossendeismacerrima* (C13919) and remained attached after collection.

***Stenopleustes* gen. inc.**

Fig. 48A

**Figure 48.**
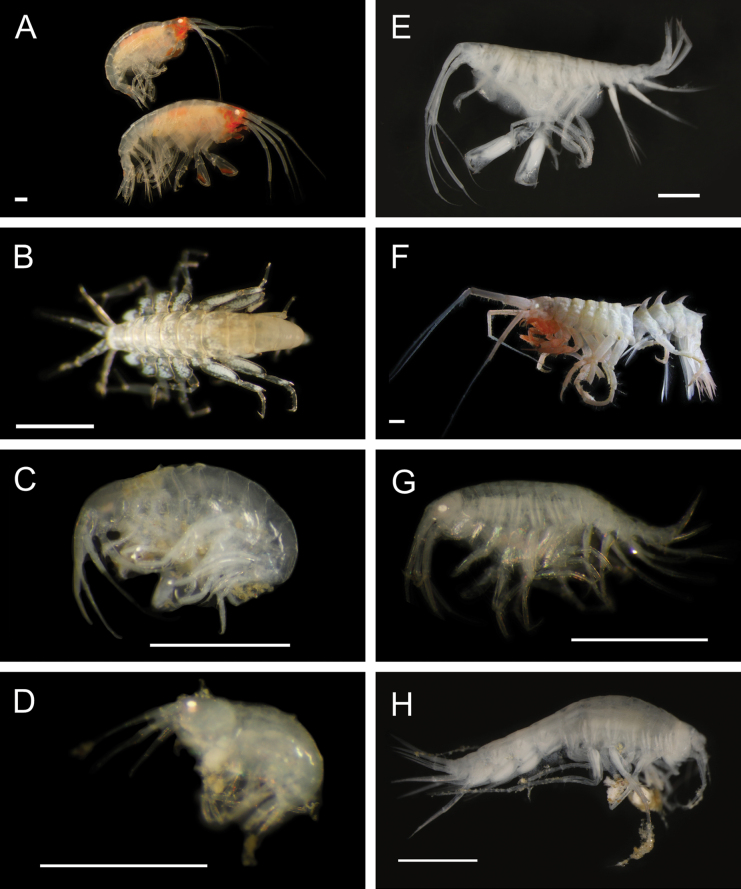
Arthropoda: Amphipoda, representative images. Live specimens are depicted unless otherwise specified **A***Stenopleustes* gen. inc. (C12811) **B***Seba* stet. (C13970) **C***Stenothoe* sp. SIO_BIC_C13857 (C13857) **D***Stenothoe* sp. SIO_BIC_C13867 (C13867) **E***Stenula* stet. (C13784, preserved specimen) **F***Rhachotropis* stet. (C13798) **G***Idunella* gen. inc. (C13856) **H***Monoculodes* stet. (C12801, preserved specimen). Scale bars: 1 mm.

**Material examined.** AD4507: C11146 (**PQ450405**), C14496; AD4916: C12788; AD4922: C12811.

**Localities.** Mound 12 (~ 967 m), Jacó Scar (~ 1852–1855 m), Parrita Scar (~ 1659–1667 m).

**Remarks.** C12811 was associated with an antipatharian coral (specific host not recorded). This morphospecies was identified as most likely *Stenopleustes* (Pleustidae), but an alternative identification is Stenothoidae. More detailed morphological examination of vouchers is needed.

#### ﻿Arthropoda | … | Amphipoda | Amphilochidea | Amphilochida | Amphilochidira | Amphilochoidea | Sebidae

***Seba* stet.**

Fig. 48B

**Material examined.** S0230: C13970.

**Localities.** Mound Jaguar (1896 m).

**Remarks.** Associated with a naturally occurring wood fall.

#### ﻿Arthropoda | … | Amphipoda | Amphilochidea | Amphilochida | Amphilochidira | Amphilochoidea | Stenothoidae

***Stenothoe* sp. SIO_BIC_C13857**

Fig. 48C

**Material examined.** AD4985: C13857.

**Localities.** Mound 12 (991 m).

**Remarks.** This morphospecies lacks eyes.

***Stenothoe* sp. SIO_BIC_C13867**

Fig. 48D

**Material examined.** AD4987: C13867.

**Localities.** Mound 12 (999 m).

**Remarks.** This morphospecies has eyes.

***Stenula* stet.**

Fig. 48E

**Material examined.** AD4974: C13784.

**Localities.** Mound 12 (992 m).

**Remarks.** Associated with experimental deployments of bone and wood.

#### ﻿Arthropoda | … | Amphipoda | Amphilochidea | Amphilochida | Eusirida | Eusiroidea | Eusiridae

***Rhachotropis* stet.**

Fig. 48F

**Material examined.** AD4507: C14497; AD4976: C13798.

**Localities.** Jacó Scar (1887 m), Parrita Scar (~ 1659–1667 m).

**Remarks.** Specimen C13798 may have been associated with experimentally deployed wood.

#### ﻿Arthropoda | … | Amphipoda | Amphilochidea | Amphilochida | Eusirida | Liljeborgioidea | Liljeborgiidae

***Idunella* gen. inc.**

Fig. 48G

**Material examined.** AD4985: C13856.

**Localities.** Mound 12 (991 m).

**Remarks.** This morphospecies has eyes. It is most likely *Idunella* (Liljeborgiidae), but an alternative identification is *Stenopleustes* (Pleustidae). Further morphological examination is needed.

#### ﻿Arthropoda | … | Amphipoda | Amphilochidea | Amphilochida | Oedicerotidira | Oedicerotoidea | Oedicerotidae

***Monoculodes* stet.**

Fig. 48H

**Material examined.** AD4507: C14495; AD4589: C11188 (**PQ449341**); AD4917: C12785; AD4922: C12801.

**Localities.** Mound 12 (965–997 m), Parrita Scar (~ 1659–1667 m).

**Remarks.** C12785 was associated with the antipatharian coral *Lillipathesritamariae*. C12801 was associated with a basket star, *Gorgonocephalus* stet. (E7064).

#### ﻿Arthropoda | … | Amphipoda | Amphilochidea | Lysianassida | Haustoriidira | Haustorioidea | Phoxocephalidae

**Phoxocephalinae subfam. inc.**

Fig. 49A

**Figure 49.**
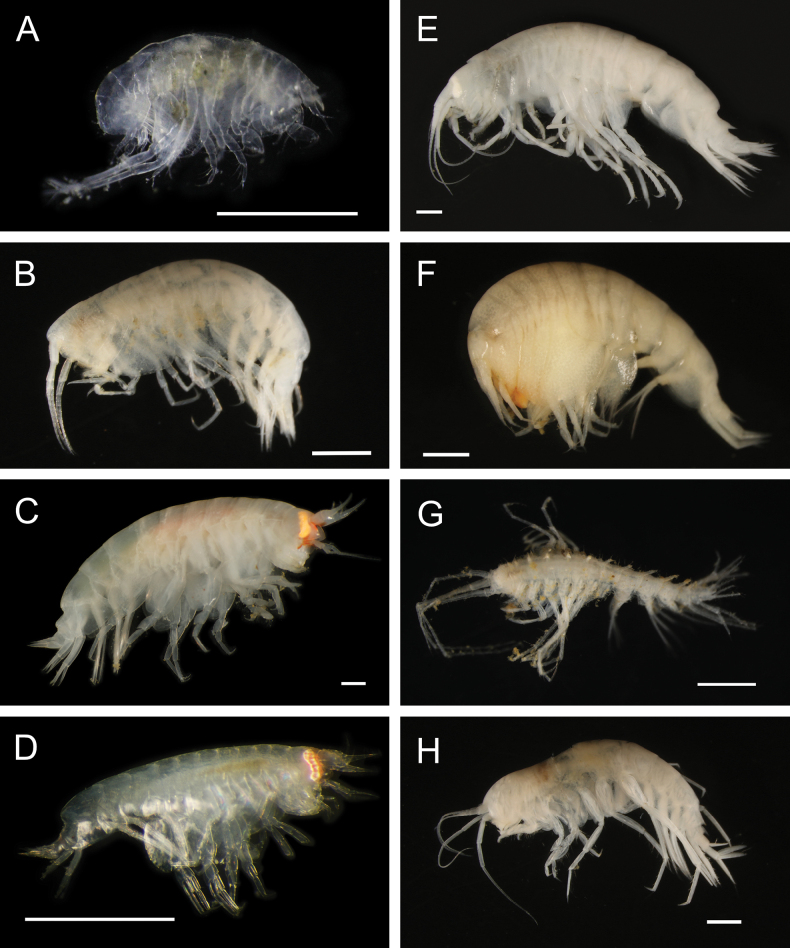
Arthropoda: Amphipoda, representative images. Live specimens are depicted unless otherwise specified **A**Phoxocephalinae subfam. inc. (C13858) **B***Ambasiella* stet. (C13917, preserved specimen) **C***Orchomene* stet. (C13972) **D**Tryphosidae stet. (C13854) **E***Ambasiopsis* stet. (C13790, preserved specimen) **F**Stegocephalidae stet. (C13976, preserved specimen) **G***Lepechinella* stet. (C13929, preserved specimen) **H***Pardalisca* stet. (C13928, preserved specimen). Scale bars: 1 mm.

**Material examined.** AD4984: C13858.

**Localities.** Mound 12 (998 m).

#### ﻿Arthropoda | … | Amphipoda | Amphilochidea | Lysianassida | Lysianassidira | Aristioidea | Ambasiidae

***Ambasiella* stet.**

Fig. 49B

**Material examined.** S0218: C13917.

**Localities.** Parrita Scar (1988 m).

**Remarks.** Associated with a xenophyophore.

#### ﻿Arthropoda | … | Amphipoda | Amphilochidea | Lysianassida | Lysianassidira | Lysianassoidea | Tryphosidae

***Orchomene* stet.**

Fig. 49C

**Material examined.** S0230: C13972, C13977.

**Localities.** Mound Jaguar (1895–1909 m).

**Tryphosidae stet.**

Fig. 49D

**Material examined.** AD4974: C13785; AD4985: C13854.

**Localities.** Mound 12 (992–1002 m).

**Remarks.** C13785 was associated with experimental deployments of bone and wood.

#### ﻿Arthropoda | … | Amphipoda | Amphilochidea | Lysianassida | Lysianassidira | Lysianassoidea incertae sedis

***Ambasiopsis* stet.**

Fig. 49E

**Material examined.** AD4972: C13789, C13790.

**Localities.** Jacó Scar (1845 m).

**Remarks.** Associated with experimentally deployed pig bones.

#### ﻿Arthropoda | … | Amphipoda | Amphilochidea | Lysianassida | Lysianassidira | Stegocephaloidea | Stegocephalidae

**Stegocephalidae stet.**

Fig. 49F

**Material examined.** S0230: C13976, C13978.

**Localities.** Mound Jaguar (1895–1908 m).

#### ﻿Arthropoda | … | Amphipoda | Amphilochidea | Lysianassida | Synopiidira | Dexaminoidea | Lepechinellidae

***Lepechinella* stet.**

Fig. 49G

**Material examined.** S0219: C13929.

**Localities.** Rio Bongo Scar (~ 480–650 m).

#### ﻿Arthropoda | … | Amphipoda | Amphilochidea | Lysianassida | Synopiidira | Dexaminoidea | Pardaliscidae

***Pardalisca* stet.**

Fig. 49H

**Material examined.** S0219: C13928.

**Localities.** Rio Bongo Scar (661 m).

**Remarks.** Associated with a naturally occurring wood fall.

#### ﻿Arthropoda | … | Amphipoda | Amphilochidea | Lysianassida | Synopiidira | Synopioidea | Argissidae

***Argissa* sp. SIO_BIC_C13930**

Fig. 50A

**Figure 50.**
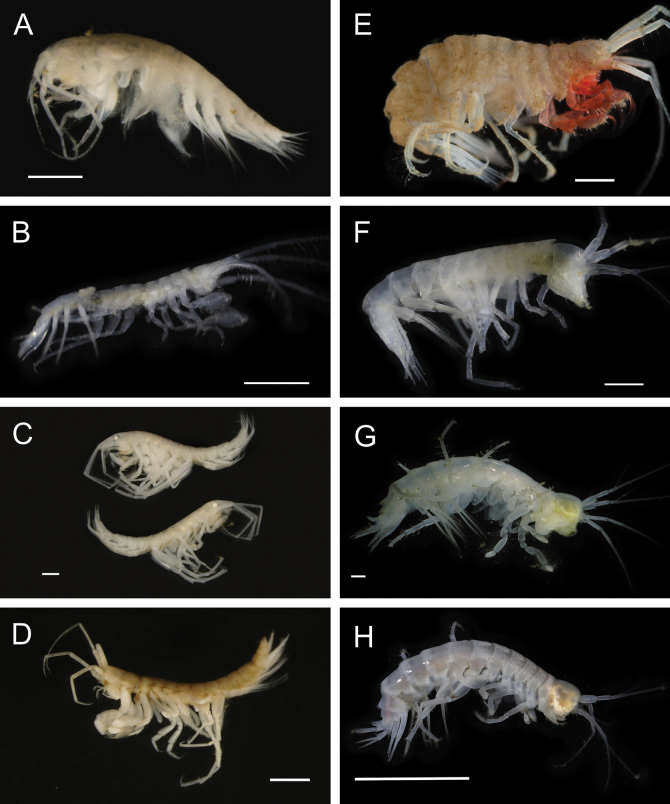
Arthropoda: Amphipoda, representative images. Live specimens are depicted unless otherwise specified **A***Argissa* sp. SIO_BIC_C13930 (C13930, preserved specimen) **B***Bonnierella* stet. (C13797) **C***Gammaropsis* gen. inc. (C13963, preserved specimen) **D***Bemlos* gen. inc. (C13931, preserved specimen) **E**Protomedeiinae stet. (C14396) **F***Oradarea* stet. (C13796) **G***Abludomelita* gen. inc. (C11141) **H***Abludomelita* stet. (C13896). Scale bars: 1 mm (**A–G**); 1 cm (**H**).

**Material examined.** S0219: C13930.

**Localities.** Rio Bongo Scar (~ 480–650 m).

**Remarks.** Possibly an undescribed species, requiring further comparison. *Argissa* is currently accepted as monotypic, with *Argissahamatipes* (Norman, 1869) reportedly occurring across the northern hemisphere at depths of 4–1096 m (Barnard 1967; Winfield et al. 2020). A specimen provisionally identified as *A.hamatipes* has been recorded from western Mexico, minimum depth 1720 m (Barnard 1967).

#### ﻿Arthropoda | … | Amphipoda | Senticaudata | Corophiida | Caprellidira | Photoidea | Ischyroceridae

***Bonnierella* stet.**

Fig. 50B

**Material examined.** AD4916: C12789; AD4972: C13797; AD4974: C13786.

**Localities.** Mound 12 (992 m), Jacó Scar (~ 1646–1751 m).

**Remarks.** C13786 was associated with experimental deployments of bone and wood.

#### ﻿Arthropoda | … | Amphipoda | Senticaudata | Corophiida | Caprellidira | Photoidea | Photidae

***Gammaropsis* gen. inc.**

Fig. 50C

**Material examined.** S0230: C13963.

**Localities.** Mound Jaguar (1895 m).

**Remarks.** One specimen is a brooding female. Further morphological examination is needed for a more definitive identification.

#### ﻿Arthropoda | … | Amphipoda | Senticaudata | Corophiida | Corophiidira | Aoroidea | Aoridae

***Bemlos* gen. inc.**

Fig. 50D

**Material examined.** S0219: C13931.

**Localities.** Rio Bongo Scar (~ 480–650 m).

#### ﻿Arthropoda | … | Amphipoda | Senticaudata | Corophiida | Corophiidira | Corophioidea | Corophiidae

**Protomedeiinae stet.**

Fig. 50E

**Material examined.** AT15-59 MC4: C14396.

**Localities.** Near Mound 11 (1031 m).

**Remarks.** This specimen was collected in a sediment core adjacent to Mound 11, ca 400 m from known sites of active seepage and likely representing the far-transition zone to the surrounding environment.

#### ﻿Arthropoda | … | Amphipoda | Senticaudata | Hadziida | Hadziidira | Calliopioidea | Calliopiidae

***Oradarea* stet.**

Fig. 50F

**Material examined.** AD4913: C12784; AD4922: C12800; AD4923: C14500; AD4976: C13796; AD4988: C13871; S0230: C13971, C13979.

**Localities.** Mound 12 (1001 m), Mound 11 (1010 m), Parrita Seep (~ 1037–1108 m), Jacó Scar (~ 1800–1900 m), Mound Jaguar (1896 m).

**Remarks.** Several specimens (C13871, C13971, C13979; possibly also C12784 and C13796) were associated with naturally occurring wood falls.

#### ﻿Arthropoda | … | Amphipoda | Senticaudata | Hadziida | Hadziidira | Hadzioidea | Melitidae

***Abludomelita* gen. inc.**

Fig. 50G

**Material examined.** AD4503: C11141; AD4923: C14498, C14499.

**Localities.** Mound 12 (990 m), Parrita Seep (~ 1037–1108 m).

**Remarks.** Specimen C11141 was associated with a naturally occurring wood fall. Specimens C14498 and C14499 were likely associated with corals.

***Abludomelita* stet.**

Fig. 50H

**Material examined.** AD4972: C13791; S0212: C13896, MZUCR-3756-01; S0230: C13975, C13980.

**Localities.** Jacó Scar (1845–1896 m), Mound Jaguar (1896–2000 m).

**Remarks.** Associated with experimentally deployed pig bones (C13791) or with naturally occurring wood falls (C13896, C13975, C13980).

#### ﻿Arthropoda | Crustacea | Malacostraca | Peracarida | Tanaidacea | Apseudomorpha | Apseudoidea | Apseudidae

**Apseudidae stet.**

Fig. 51A

**Figure 51.**
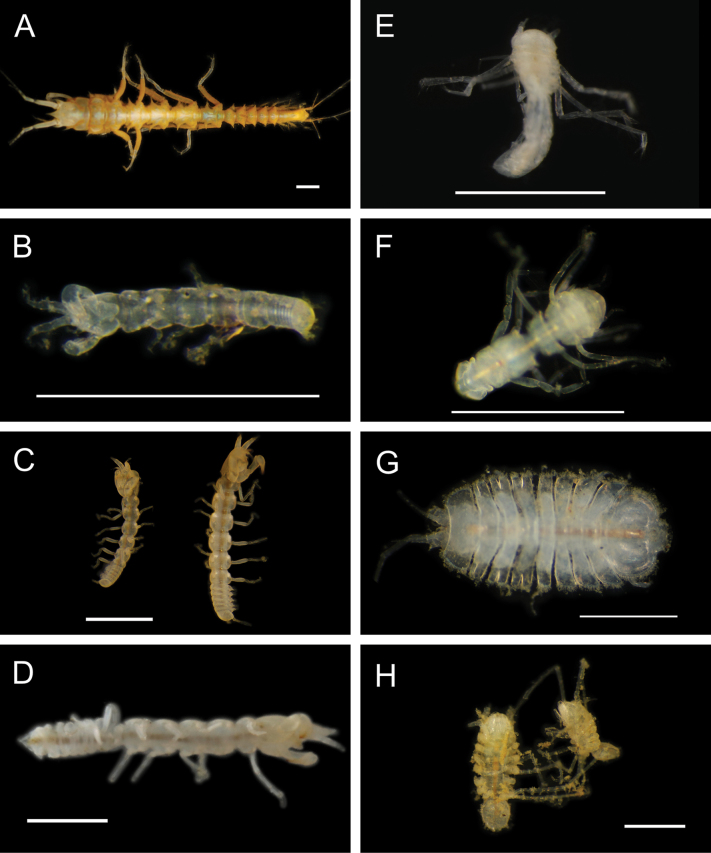
Arthropoda: Tanaidacea and Isopoda, representative images. Live specimens are depicted unless otherwise specified **A**Apseudidae stet. (C13973) **B***Agathotanais* gen. inc. (C13815) **C***Paranarthrura* stet. (C13874) **D***Anarthrurella* gen. inc. (C13777) **E***Desmosoma* stet. (C13855, preserved specimen) **F***Mirabilicoxa* stet. (C13866) **G***Janthura* stet. (C13848) **H***Munna* stet. (C13849). Scale bars: 1 mm.

**Material examined.** S0230: C13973.

**Localities.** Mound Jaguar (1909 m).

#### ﻿Arthropoda | Crustacea | Malacostraca | Peracarida | Tanaidacea | Tanaidomorpha | Paratanaoidea | Agathotanaidae

***Agathotanais* gen. inc.**

Fig. 51B

**Material examined.** AD4979: C13815.

**Localities.** Quepos Slide (397 m).

**Remarks.** Collected 2 cm deep in a sediment core. Identification is uncertain because the specimen is a juvenile.

***Paranarthrura* stet.**

Fig. 51C

**Material examined.** AD4989: C13874.

**Localities.** Jacó Scar (1768 m).

**Remarks.** Collected from a vestimentiferan tubeworm bush.

#### ﻿Arthropoda | Crustacea | Malacostraca | Peracarida | Tanaidacea | Tanaidomorpha | Paratanaoidea | Anarthruridae

***Anarthrurella* gen. inc.**

Fig. 51D

**Material examined.** AD4971: C13777.

**Localities.** Jacó Scar (1824 m).

#### ﻿Arthropoda | Crustacea | Malacostraca | Peracarida | Isopoda | Asellota | Janiroidea | Desmosomatidae

***Desmosoma* stet.**

Fig. 51E

**Material examined.** AD4985: C13855.

**Localities.** Mound 12 (1002 m).

***Mirabilicoxa* stet.**

Fig. 51F

**Material examined.** AD4987: C13866.

**Localities.** Mound 12 (999 m).

#### ﻿Arthropoda | Crustacea | Malacostraca | Peracarida | Isopoda | Asellota | Janiroidea | Janiridae

***Janthura* stet.**

Fig. 51G

**Material examined.** AD4988: C13848.

**Localities.** Mound 11 (1010 m).

**Remarks.** Associated with a naturally occurring wood fall.

#### ﻿Arthropoda | Crustacea | Malacostraca | Peracarida | Isopoda | Asellota | Janiroidea | Munnidae

***Munna* gen. inc.**

Fig. 51H

**Material examined.** AD4988: C13872.

**Localities.** Mound 11 (1010 m).

**Remarks.** Associated with a naturally occurring wood fall.

***Munna* stet.**

Fig. 52A

**Figure 52.**
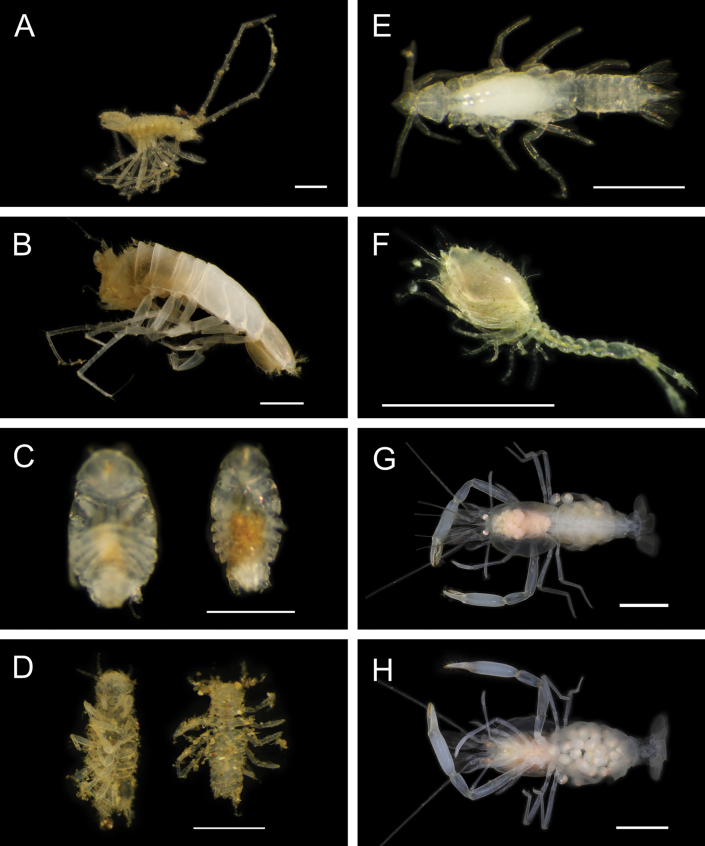
Arthropoda: Isopoda, Cumacea, and Stenopodidea, representative live images **A***Munna* gen. inc. (C13872) **B***Betamorpha* stet. (C13974) **C***Eurycope* stet. (C13850) **D***Tenupedunculus* stet. (C13823) **E**Gnathiidae stet. (C13801) **F***Paraleucon* sp. SIO_BIC_C11152 (C11152) **G**Spongicoloidescf.galapagensis (C12820, dorsal view) **H**Spongicoloidescf.galapagensis (C12820, ventral view). Scale bars: 1 mm (**A–F**); 1 cm (**G, H**).

**Material examined.** AD4988: C13849.

**Localities.** Mound 11 (1010 m).

**Remarks.** Associated with a naturally occurring wood fall.

#### ﻿Arthropoda | Crustacea | Malacostraca | Peracarida | Isopoda | Asellota | Janiroidea | Munnopsidae

***Betamorpha* stet.**

Fig. 52B

**Material examined.** S0230: C13974.

**Localities.** Mound Jaguar (1909 m).

***Eurycope* stet.**

Fig. 52C

**Material examined.** AD4990: C13850.

**Localities.** Parrita Seep (1401 m).

#### ﻿Arthropoda | Crustacea | Malacostraca | Peracarida | Isopoda | Asellota | Stenetrioidea | Stenetriidae

***Tenupedunculus* stet.**

Fig. 52D

**Material examined.** AD4987: C13823.

**Localities.** Mound 12 (999 m).

**Remarks.** Associated with sediment samples.

#### ﻿Arthropoda | Crustacea | Malacostraca | Peracarida | Isopoda | Cymothoida | Cymothooidea | Gnathiidae

**Gnathiidae stet.**

Fig. 52E

**Material examined.** AD4978: C13801.

**Localities.** Mound 12 (~ 996–999 m).

#### ﻿Arthropoda | Crustacea | Malacostraca | Peracarida | Cumacea

***Paraleucon* sp. SIO_BIC_C11152**

Fig. 52F

**Material examined.** AD4510: C11152; AD4987: C13860; AD4988: C13873.

**Localities.** Jacó Summit (741 m), Mound 11 (1010 m), Mound 12 (1010 m).

**Remarks.** Likely an undescribed species. C13860 was associated with a bed of small clams. C13873 was associated with a naturally occurring wood fall.

#### ﻿Arthropoda | Crustacea | Malacostraca | Eucarida | Decapoda

We list entries following the phylogeny in Wolfe et al. (2019).

#### ﻿Arthropoda | … | Decapoda | Pleocyemata | Stenopodidea | Spongicolidae

***Spongicoloidesgalapagensis* Goy, 1980**

Fig. 52G, H

**Material examined.** AD4923: C12735, C12814 (**PQ449346**, **PQ449347**), C12820.

**Localities.** Parrita Seep (~ 1091–1098 m).

**Distribution.** Described from 717 m off the Galápagos Islands (Goy 1980). Comparison to records of S.aff.galapagensis associated with euplectellid sponges at seeps at ~ 1000 m off central Chile (Guzmán and Sellanes 2011) would be informative.

**New records.** The CRM specimens represent new northern records and new maximum depth records for this species (1091 m as the most conservative value).

**Remarks.** Associated with a euplectellid sponge, *Dictyaulus* gen. inc. (P1687). One specimen of C12820 is an ovigerous female.

#### ﻿Arthropoda | … | Decapoda | Pleocyemata | Caridea | Alpheoidea | Thoridae

***Lebbeusscrippsi* Wicksten & Méndez G., 1982**

Fig. 53A

**Figure 53.**
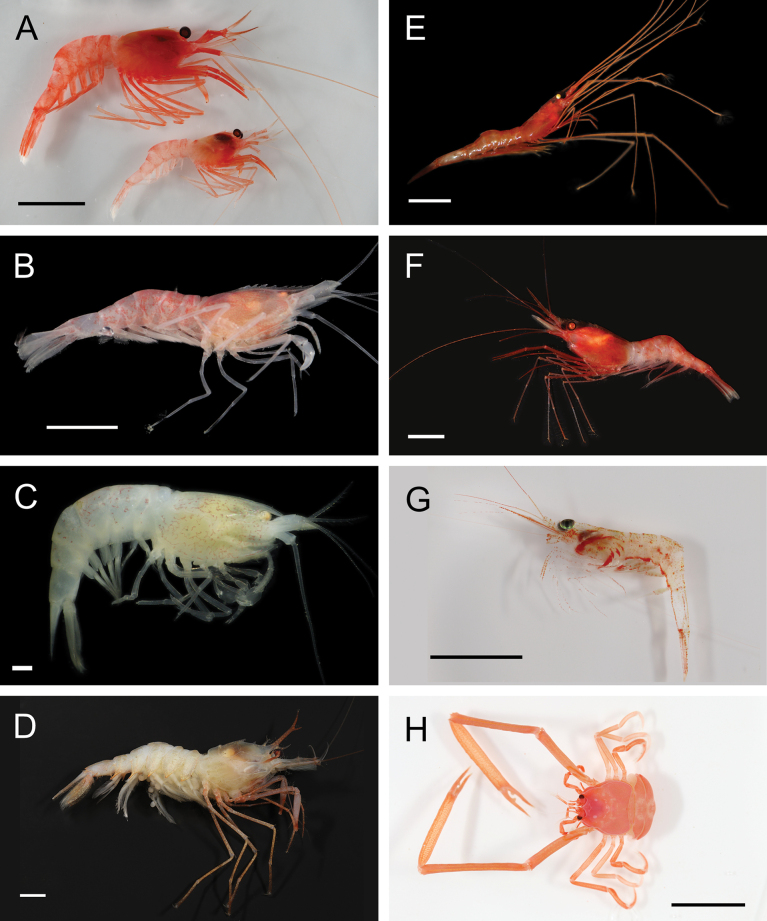
Arthropoda: Caridea and Anomura, representative live images **A***Lebbeusscrippsi* (C12822) **B***Alvinocariscostaricensis* (MZUCR 3748-01) **C***Alvinocaris* sp. SIO_BIC_C11136 (C11136) **D***Paracrangonareolata* (C12751) **E***Nematocarcinusfaxoni* (C13899) **F***Heterocarpusvicarius* (C13921) **G***Plesionikatrispinus* (C12806) **H***Heteroptychusgalapagos* (C12816, dorsal view). Scale bars: 1 cm (**A, B**, **D–H**); 1 mm (**C**).

**Material examined.** AD4924: C12822; AD4989: C13878, C13879, MZUCR-3747-01; AD4990: C13880, C13881, C13882.

**Localities.** Parrita Seep (~ 1400–1410 m), Jacó Scar (~ 1768–1785 m).

**Distribution.** Originally described from northern Chile and southern Peru, 768–1164 m (Wicksten and Méndez 1982), and recorded as far north as the southeastern Gulf of California, 1188–1245 m (Hendrickx 1996, 2001).

**New records.**CRM specimens C13878, C13879, and MZUCR-3747-01 represent new maximum depth records for this species (1768 m as the most conservative value).

#### ﻿Arthropoda | … | Decapoda | Pleocyemata | Caridea | Bresilioidea | Alvinocarididae

***Alvinocariscostaricensis* Martin, Wall, Shank, Cha, Seid & Rouse, 2018**

Fig. 53B

**Reference.**Martin et al. 2018**.

**Additional material examined.** S0213: MZUCR 3748-01 (voucher), C13898 (tissue).

**Localities.** Jacó Summit (742 m; this study), Mound 12 (~ 1000 m; type locality), Jacó Scar (~ 1800 m).

**Distribution.** Known only from the CRM seeps.

**New records.** Specimen MZUCR 3748-01 from Jacó Summit (742 m) represents a new minimum depth record for this species.

***Alvinocaris* sp. SIO_BIC_C11136**

Fig. 53C

**Reference.**Martin et al. 2018.

**Material examined.** AD4501: C11136; AD4503: C13307; AD4589: C11190; AD4974: C13782; AD4978: C13806; AD4984: C13861, C13862, C13863, C13893, MZUCR-3749-01.

**Localities.** Mound 12 (~ 967–999 m).

**Remarks.** An undescribed species, previously noted as morphologically and genetically distinct from *A.costaricensis* (Martin et al. 2018).

#### ﻿Arthropoda | … | Decapoda | Pleocyemata | Caridea | Crangonoidea | Crangonidae

***Paracrangonareolata* Faxon, 1893**

Fig. 53D

**Material examined.** AD4924: C12751 (**PQ449342**; 16S: **PQ304652**; 18S: **PQ304647**).

**Localities.** Parrita Seep (1402 m).

**Distribution.** Southern Gulf of California and western Mexico (type locality: Islas Marías, 1236–1244 m (Faxon 1893, 1895)) to central Chile, 580–1650 m (Hendrickx 1996; Báez and Soto 1997; Wicksten 2020).

**Remarks.** This specimen is an ovigerous female.

#### ﻿Arthropoda | … | Decapoda | Pleocyemata | Caridea | Nematocarcinoidea | Nematocarcinidae

***Nematocarcinusfaxoni* Burukovsky, 2001**

Fig. 53E

**Material examined.** S0213: C13899.

**Localities.** Jacó Summit (763 m).

**Distribution.** Mexico to the Galápagos Islands and southwestern Atlantic, 660–2055 m (Hendrickx and Hernández-Payán 2018; Martínez-Guerrero and López-Pérez 2018).

#### ﻿Arthropoda | … | Decapoda | Pleocyemata | Caridea | Pandaloidea | Pandalidae

***Heterocarpusvicarius* Faxon, 1893**

Fig. 53F

**Material examined.** S0213: C13900; S0219: C13921.

**Localities.** Rio Bongo Scar (661 m), Jacó Summit (763 m).

**Distribution.** Originally described from several stations in the Gulf of Panama, 384–523 m (Faxon 1893, 1895) and recorded from the Gulf of California to Peru, 73–1454 m (Fischer et al. 1995). This species is commercially harvested in Costa Rica, typically at depths of ﻿250–300 m (Fischer et al. 1995; Wehrtmann and Echeverría-Sáenz 2007).

**Remarks.** C13921 was associated with a naturally occurring wood fall.

***Plesionikatrispinus* Squires & Barragan, 1976**

Fig. 53G

**Material examined.** AD4919: C12806.

**Localities.** Quepos Slide (~ 379–397 m).

**Distribution.** Originally described from 209–302 m off the Pacific coast of Colombia (Squires and Barragan 1976) and recorded from the southern Gulf of California to Peru, 96–500 m (Fischer et al. 1995), including the Galápagos Islands (Arnés-Urgellés et al. 2020) and Costa Rica (Wehrtmann and Echeverría-Sáenz 2007; Azofeifa-Solano and Cortés 2020).

#### ﻿Arthropoda | … | Decapoda | Pleocyemata | Anomura | Chirostyloidea | Chirostylidae

***Heteroptychusgalapagos* Baba & Wicksten, 2019**

Figs 53H, 54A

**Figure 54.**
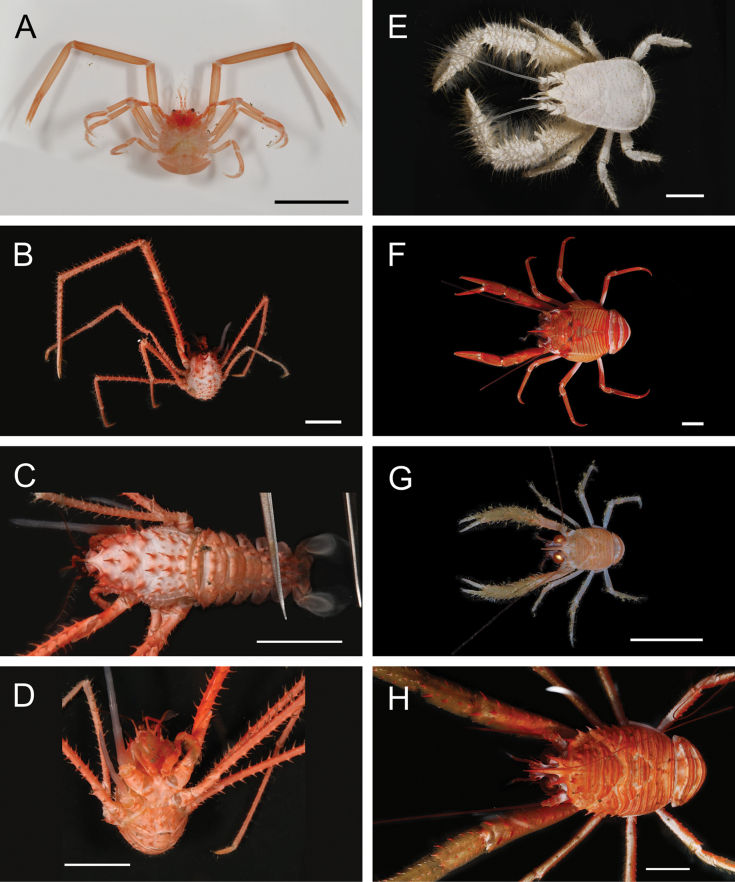
Arthropoda: Anomura, representative live images **A***Heteroptychusgalapagos* (C12817, ventral view) **B***Sternostylusdefensus* (C12804, dorsal view) **C***Sternostylusdefensus* (C12804, dorsal detail) **D***Sternostylusdefensus* (C12804, ventral view) **E***Kiwapuravida* (MZUCR 3735-01) **F***Grimotheamonodon* (C13909) **G***Typhlonidapropinqua* (C11153, juvenile) **H***Typhlonidapropinqua* (C13915, adult). Scale bars: 1 cm.

**Material examined.** AD4923: C12816 (**PQ449348**), C12817 (**PQ449349**).

**Localities.** Parrita Seep (1037 m).

**Distribution.** Previously known only from East Darwin Seamount, Galápagos Islands, 1012 m (Baba and Wicksten 2019).

**New records.** The CRM specimens represent new northern records of this species.

**Remarks.** Associated with isidid corals Co2952, Co2953, and/or MZUCR 3129. C12816 is an ovigerous female.

***Sternostylusdefensus* (Benedict, 1902)**

Fig. 54B–D

**Material examined.** AD4923: C12804 (**PQ450385**).

**Localities.** Parrita Seep (~ 1035–1108 m).

**Distribution.** Originally described from the Galápagos Islands, 717 m (Baba and Haig 1990), and recently reported from 873–1210 m at seamounts in the northern Galápagos Marine Reserve (Arnés-Urgellés et al. 2020; Salinas-de-León et al. 2020).

**New records.** The CRM specimen represents a new northern record of this species.

**Remarks.** Several antipatharians and octocorals were collected during this dive, and the exact host was not recorded. At the Galápagos seamounts, *S.defensus* is likely associated with antipatharian corals and possibly with *Chrysogorgia* (Baba and Wicksten 2019).

#### ﻿Arthropoda | … | Decapoda | Pleocyemata | Anomura | Chirostyloidea | Kiwaidae

***Kiwapuravida* Thurber, Jones & Schnabel, 2011**

Fig. 54E

**Reference.**Thurber et al. 2011**.

**Localities.** Mound 12 (~ 1000 m; type locality), Mound 11 (~ 1000–1040 m).

**Distribution.** Known only from the CRM seeps.

**Remarks.** An iconic megafaunal species of the CRM seeps (Fig. 2D), the “Costa Rican dancing yeti crab” cultivates ectosymbiotic bacteria on its chelipeds and harvests the bacteria as its main source of food; the animals are often found in aggregations at areas of active fluid seepage, where the slow waving of the chelipeds is hypothesized to shear off the nutrient-depleted boundary layer and thereby enhance the productivity of the ectosymbionts (Thurber et al. 2011).

#### ﻿Arthropoda | … | Decapoda | Pleocyemata | Anomura | Galatheoidea | Munididae

***Grimotheamonodon* (H. Milne Edwards, 1837)**

Fig. 54F

**Material examined.** AD4918: C12805; AD4921: C12808 (**PQ449345**); AD4979: C13809, C13810, C13811.

**Localities.** Quepos Slide (~ 333–408 m).

**Distribution.** Previously recorded from Costa Rica, 150–350 m, where it is abundant as bycatch in deepwater shrimp fisheries (Wehrtmann et al. 2010). Also known from central Mexico to southern Chile (type locality: unspecified site on the Chilean coast) (Milne Edwards 1837), to 523 m depth (Faxon 1895; Hendrickx and Harvey 1999; Baba et al. 2008).

**Remarks.** This species is commercially harvested off Chile and serves as prey for other species of commercial interest (Yapur-Pancorvo et al. 2023).

***Typhlonidapropinqua*** (**Faxon, 1893)**

Fig. 54G, H

**Material examined.** AD4510: C11153; S0213: C13901; S0215: C13915, MZUCR-3746-01; S0219: C13925.

**Localities.** Rio Bongo Scar (661 m), Jacó Summit (741–763 m), Mound 12 (991 m).

**Distribution.** Originally described from the Gulf of Panama (838–935 m) and Galápagos Islands (704 m) (Faxon 1895) and distributed south to Peru and central Chile, 700–1713 m (Wicksten 1989, 2020; Baba et al. 2008; Retamal et al. 2020).

**Remarks.** C11153, C13901, and C13925 are juveniles. C13925 was associated with a naturally occurring wood fall.

#### ﻿Arthropoda | … | Decapoda | Pleocyemata | Anomura | Galatheoidea | Munidopsidae

We thank Paula Rodríguez-Flores (Harvard University, U.S. National Museum of Natural History) for contributing to morphological identification of these specimens and generating many DNA sequences. See Rodríguez-Flores et al. (2023) for discussion of the phylogenetics and biogeography of eastern Pacific Munidopsidae.

***Munidopsisagassizii* Faxon, 1893**

Fig. 55A

**Figure 55.**
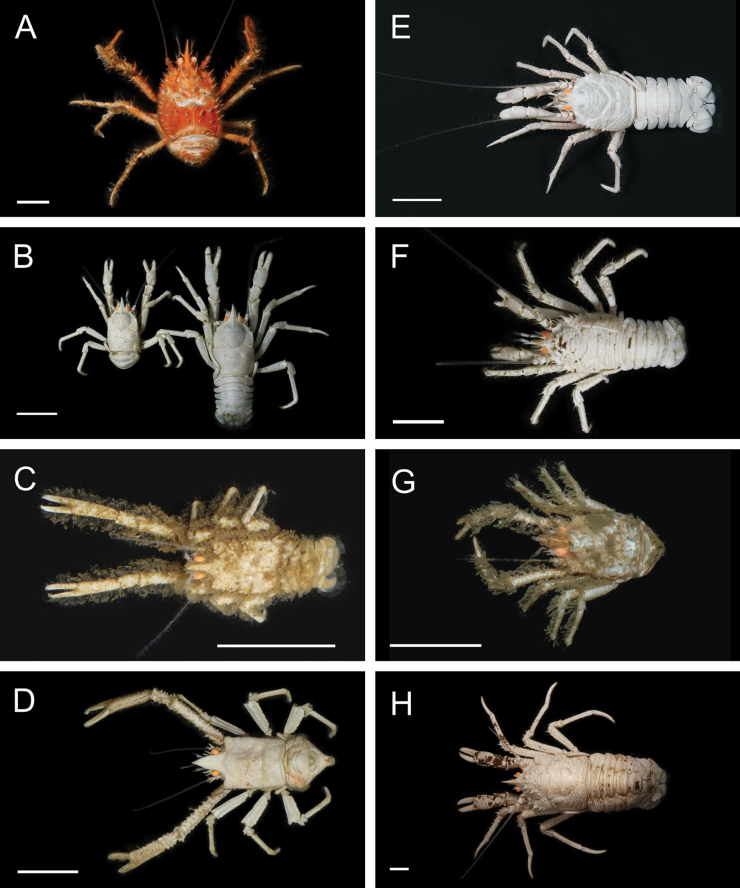
Arthropoda: *Munidopsis*, representative live images **A***Munidopsisagassizii* (C13922) **B***Munidopsisalvisca* (C13904) **C***Munidopsisaspera* (C13912) **D***Munidopsiscarinipes* (C11150) **E**Munidopsiscf.nitida (C12818) **F**Munidopsiscf.nitida (C13903) **G**Munidopsiscf.opalescens (C11154) **H**Munidopsiscf.producta (MZUCR-3745-01). Scale bars: 1 cm.

**Reference.**Rodríguez-Flores et al. (2023) for phylogenetic analysis.

**Material examined.** S0219: C13922 (ON886860), C13923.

**Localities.** Rio Bongo Scar (661 m).

**Distribution.** Gulf of Panama (type locality) to northern Chile, 384–1000 m (Guzman and Sellanes 2015).

***Munidopsisalvisca* A.B. Williams, 1988**

Fig. 55B

**Reference.**Rodríguez-Flores et al. (2023) for phylogenetic analysis.

**Material examined.** AD4509: C11149; AD4513: C11158, C11159 (**PQ450374**); AD4914: C12783 (ON886848), C12790 (ON886849); AD4591: C11181; AD4914: C12791; AD4972: C13792; AD4989: C13876; AD4990: C13883; S0214: C13904.

**Localities.** Parrita Seep (1401 m; this study), Jacó Slope (1459 m; this study), Jacó Scar (~ 1632–1886 m).

**Distribution.** Originally described from vents in the Guaymas Basin (type locality), Gulf of California, 2008 m, and the Explorer and Juan de Fuca Ridges, 1545–1818 m (Williams 1988). Material identified as “most probably *Munidopsisalvisca*” has been reported from Pacific Costa Rica (locality and depth not specified) (Wehrtmann et al. 2010).

**New records.** Confirming the occurrence of *M.alvisca* in Costa Rica, our specimens represent new southern records, new seep records, and a new minimum depth record for this species (specimen C13883 from 1401 m).

**Remarks.** Several specimens are ovigerous females: C11149, C11158, C11181, and C13792. Specimen C13876 was the host of sacculinid parasite C13875.

***Munidopsisaspera* (Henderson, 1885)**

Fig. 55C

**Reference.**Rodríguez-Flores et al. (2023) for phylogenetic and taxonomic treatment.

**Localities.** The Thumb (1065 m).

**Distribution.** Originally described from the Straits of Magellan, 448 m (Henderson, 1885), and recorded along the eastern Pacific from southern California to northern Chile, 166–1398 m (Rodríguez-Flores et al. 2023), including records from Isla del Coco (Baba et al. 2008).

**Remarks.** Specimens C13912 and C13913 from dive S0217 were associated with the holothuroid E7313 (Synallactescf.chuni) and heavily coated with bacteria and/or sediment.

***Munidopsiscarinipes* Faxon, 1893**

Fig. 55D

**Reference.**Rodríguez-Flores et al. (2023) for phylogenetic and taxonomic treatment.

**Localities.** Mound 12 (~ 990–1011 m), Mound 11 (1010 m), Jacó Scar (1459 m).

**Distribution.** Known only from the CRM and western Panama, 915–1459 m (Rodríguez-Flores et al. 2023).

**Remarks.** Specimen C13869 from dive AD4988 was associated with a naturally occurring wood fall. Specimen C13916 from dive S0215 was associated with experimentally deployed cow or pig bones.

**Munidopsiscf.nitida (A. Milne Edwards, 1880)**

Fig. 55E, F

**Reference.**Rodríguez-Flores et al. (2023) for phylogenetic analysis.

**Material examined.** AD4924: C12818 (**PQ450377**); S0214: C13903 (ON886857; 16S: ON858040; 28S: ON858109).

**Localities.** Parrita Seep (1402 m), Jacó Scar (1875 m).

**Remarks.** C13903 was associated with a naturally occurring wood fall. These specimens resemble *M.nitida*, but C13903 belongs to a potential species complex warranting further investigation (Rodríguez-Flores et al. 2023). Originally described from Guadaloupe Island, Caribbean Sea, 1407–1607 m, *M.nitida* has been reported from the western Atlantic, Indian Ocean, western Pacific, and eastern Pacific over a depth range of 592–3680 m (Baba et al. 2008). In the eastern Pacific, *M.nitida* has been reported from the Gulf of Panama and Isla del Coco (Wehrtmann et al. 2010; Azofeifa-Solano and Cortés 2020; Wicksten 2020).

**Munidopsiscf.opalescens Benedict, 1902**

Fig. 55G

**Reference.**Rodríguez-Flores et al. (2023) for phylogenetic analysis (reported as *M.opalescens*).

**Material examined.** AD4510: C11154 (ON886842).

**Localities.** Jacó Summit (741 m).

**Remarks.***M.opalescens* was originally described from the Magellan region of Chile and is known only from Chilean waters, 636–922 m (Wicksten 1989, 2020; Baba et al. 2008; Guzman and Sellanes 2015), with a northernmost record at 32°S (Guzman and Sellanes 2015). Although the CRM specimen is morphologically consistent with *M.opalescens*, the COI sequence was only 98.68% identical to a reference sequence of *M.opalescens* from Chile (JN166759.1) (Ahyong et al. 2011), exceeding the typically low (<1%) intraspecific variation in Pacific *Munidopsis* species (Jones and Macpherson 2007; Rodríguez-Flores et al. 2023). If confirmed as *M.opalescens* by further comparison to type material, the CRM specimen would represent the first record of *M.opalescens* in the northern hemisphere and a northward range extension of ca 5000 km.

**Munidopsiscf.producta Baba, 2005**

Fig. 55H

**Material examined.** S0220: MZUCR-3745-01 (voucher), C13926 (tissue).

**Localities.** Subduction Plume (3399 m).

**Remarks.***M.producta* was originally described from Costa Rica (ca 400 km offshore, 3680 m) and occurs from the Pescadero Basin hydrothermal vents in the Gulf of California (Rodríguez-Flores et al. 2023) to the Gulf of Panama, 3260–3680 m (Baba 2005; Wicksten 2020). The CRM specimen, an ovigerous female, appears morphologically consistent with *M.producta* and was collected ~ 500 km from the type locality at a similar depth. DNA sequences were not obtained due to bacterial contamination and more detailed morphological comparison is required, so we conservatively designate this specimen as M.cf.producta.

**Munidopsiscf.trifida Henderson, 1885**

Fig. 56A, B

**Figure 56.**
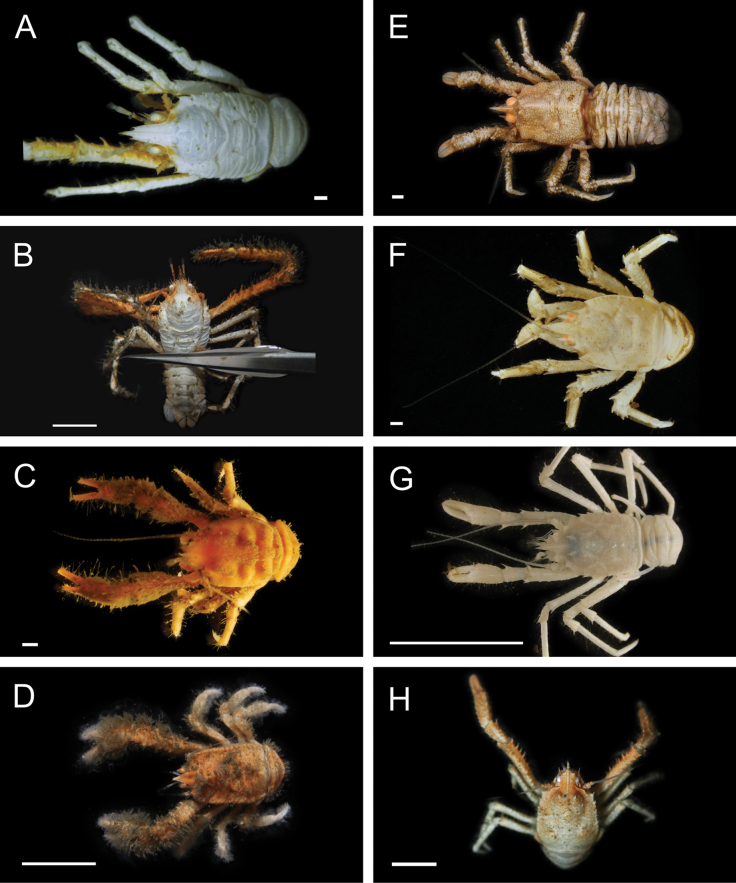
Arthropoda: *Munidopsis*, representative images. Live specimens are depicted unless otherwise specified **A**Munidopsiscf.trifida (C11145) **B**Munidopsiscf.trifida (C12813) **C***Munidopsiscortesi* (C13902) **D***Munidopsisgirguisi* (C13897) **E***Munidopsisgranosicorium* (C12819) **F***Munidopsishendersoniana* (C12810) **G***Munidopsissimilis* (C13964, preserved specimen, credit: Paula Rodríguez-Flores) **H***Munidopsis* sp. SIO_BIC_C11177 (C13830). Scale bars: 1 mm (**A, C, E, F**); 1 cm (**B, D, G, H**).

**Reference.**Rodríguez-Flores et al. (2023) for phylogenetic analysis (reported as *M.trifida*).

**Material examined.** AD4507: C11145 (ON886840); AD4586: C14391; AD4923: C12813 (ON886850; 16S: ON858033; 28S: ON858102); AD4985: C13829 (tissue); S0217: MZUCR-3733-01 (voucher), C13918 (tissue).

**Localities.** Mound 12 (~ 982–998 m), The Thumb (1071 m), Parrita Seep (1091 m), Parrita Scar (1660 m).

**Remarks.***M.trifida* was originally described from the Straits of Magellan, Chile, 732 m (Henderson 1885), and has been reported from the western Pacific and Indian Oceans, 280–1100 m (Baba 2005; Baba et al. 2008). The COI sequence of C11145 was identical to those of specimens from Chile that were previously identified as *M.trifida* (JN166765.1, JN166764.1) (Ahyong et al. 2011). Cryptic diversity within this putatively widespread species is currently under investigation, and the CRM specimens may represent at least one undescribed species. Any CRM specimens confirmed as *M.trifidasensu stricto* would represent new northern records in the eastern Pacific and new depth records for this species.

***Munidopsiscortesi* Rodríguez-Flores, Seid, Rouse & Giribet, 2023**

Fig. 56C

**Reference.**Rodríguez-Flores et al. 2023**.

**Localities.** Jacó Summit (742 m; type locality).

**Distribution.** Known only from the CRM seeps.

***Munidopsisgirguisi* Rodríguez-Flores, Seid, Rouse & Giribet, 2023**

Fig. 56D

**Reference.**Rodríguez-Flores et al. 2023**.

**Localities.** Jacó Summit (~ 730–820 m).

**Distribution.** Eastern Pacific seeps from southern California (type locality) to the CRM, 381–845 m (Rodríguez-Flores et al. 2023).

***Munidopsisgranosicorium* A.B. Williams & Baba, 1989**

Fig. 56E

**Reference.**Rodríguez-Flores et al. (2023) for phylogenetic and taxonomic treatment.

**Localities.** Parrita Seep (1402 m).

**Distribution.** Cascadia Basin off the Strait of Juan de Fuca (type locality) to the CRM, 1402–2020 m (Rodríguez-Flores et al. 2023).

***Munidopsishendersoniana* Faxon, 1893**

Fig. 56F

**Reference.**Rodríguez-Flores et al. (2023) for phylogenetic analysis.

**Material examined.** AD4503: C11142 (ON886839); AD4922: C12803, C12809 (ON886884), C12810, C12812; AD4974: C13783; AD4988: C13868.

**Localities.** Mound 12 (~ 966–1005 m), Mound 11 (1010 m).

**Distribution.** Southwestern Mexico to the Gulf of Panama (type locality), 1101–1869 m (Faxon 1895; Baba 2005; Wicksten 2020).

**Remarks.** Some specimens were associated with naturally occurring wood falls (C11142, C12810, and C13868) or cnidarians (C12799 with hormathiid anemone Co2933; C12803 with hormathiid anemone Co2875; C12809 with anthoptilid sea pen Co2937). C12809 includes an ovigerous female.

***Munidopsissimilis* Smith, 1885**

Fig. 56G

**Reference.**Rodríguez-Flores et al. (2023) for phylogenetic and taxonomic treatment.

**Localities.** Mound Jaguar (1895 m).

**Distribution.** Originally described from the North Atlantic off the eastern United States, 1939 m (Smith 1885), with apparent genetic connectivity from the northwest Atlantic Ocean to the northeast Pacific and Bering Sea, 1390–3314 m (Rodríguez-Flores et al. 2023).

***Munidopsis* sp. SIO_BIC_C11177**

Fig. 56H

**Reference.**Rodríguez-Flores et al. (2023) for phylogenetic analysis.

**Material examined.** AD4590: C11173, C11175, C11177 (**PQ450376**), C11179 (**PQ450375**); AD4985: C13830 (ON886853).

**Localities.** Mound 12 (~ 1000 m), Jacó Scar (1800 m).

**Remarks.** An undescribed species.

#### ﻿Arthropoda | … | Decapoda | Pleocyemata | Anomura | Lithodoidea | Lithodidae

**Glyptolithodesaff.cristatipes (Faxon, 1893)**

Fig. 57A, B

**Figure 57.**
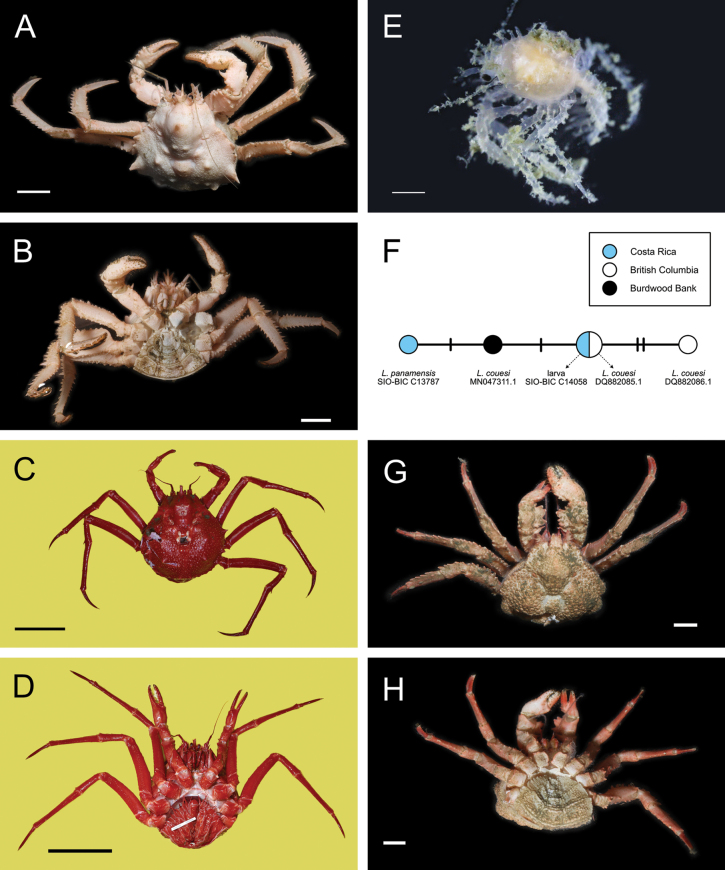
Arthropoda: Lithodidae, representative live images **A**Glyptolithodesaff.cristatipes (C12793, dorsal view) **B**Glyptolithodesaff.cristatipes (C12793, ventral view) **C***Lithodespanamensis* (C13787, dorsal view) **D***Lithodespanamensis* (C13787, ventral view) **E**Lithodescf.couesi (C14058) **F** Haplotype network of *L.panamensis* and *L.couesi*COI sequences **G**Paralomisaff.diomedeae (C13788, dorsal view) **H**Paralomisaff.diomedeae (C13788, ventral view). Scale bars: 1 cm (**A, B, G, H**); 10 cm (**C, D**); 1 mm (**E**).

**Material examined.** AD4916: C12793 (tissue).

**Localities.** Jacó Scar (1742 m).

**Remarks.** Resembles but is distinct from *Glyptolithodescristatipes* (Faxon, 1893), which was described from western Panama, 589 m (Faxon 1895).

**Lithodescf.couesi Benedict, 1895**

Fig. 57C

**Material examined.** AD4985: C14058 (**PQ449358**).

**Localities.** Mound 12 (995 m depth, ~ 5 m above the seafloor).

**Remarks.***L.couesi* was originally described from Unalaska, Aleutian Islands, at 730 m (Benedict, 1895), and has been reported to range from northern Japan to Baja California, with an overall depth range of 258–1400 m (Wicksten, 1989; Hendrickx et al. 2014; Wicksten, 2020). A recent molecular study reported *L.couesi* from the Burdwood Bank, Scotia Arc, southwestern Atlantic Ocean, 605 m, showing only minimal COI haplotype variation compared to individuals from Alaska and British Columbia and indicating a pole-to-pole distribution of this species (Pérez-Barros et al. 2020). Pérez-Barros et al. (2020) hypothesize that *L.couesi* colonized the Burdwood Bank via deep-sea larval circulation, and that with increased sampling effort *L.couesi* is likely to be reported from intermediate locations between Baja California and the Scotia Arc.

Consistent with this hypothesis, the COI sequence of the CRM lithodid larva C14058 is 100% identical to that of *L.couesi* from British Columbia (DQ882085.1) and only 2–3 bases different from additional *L.couesi* sequences (DQ882086.1 from British Columbia, 99.53% identity; MN047311.1 from Burdwood Bank, 99.83% identity) (Fig. 57D). The C14058 sequence is also only 4 bases (<1%) divergent from that of the CRM*Lithodespanamensis* specimen C13787 (below), which represents the first available reference sequence for *L.panamensis*. Evidently, COI does not meaningfully differentiate between these species, and further investigation or revision is warranted.

***Lithodespanamensis* Faxon, 1893**

Fig. 57E, F

**Material examined.** AD4975: C13787 (**PQ449352**).

**Localities.** Mound 12 (1000 m). Previously reported off Puntarenas, Costa Rica, 700–1400 m, although without specific mention of a seep habitat (MacPherson and Wehrtmann 2010).

**Distribution.** Originally described from 838 m in the Gulf of Panama (Faxon 1893, 1895) and reported to the Galápagos seamounts, Peru, and northern Chile, 620–850 m (Wicksten 1989; Arguelles et al. 2020; Arnés-Urgellés et al. 2020; Retamal et al. 2020).

**Remarks.** Host of the rhizocephalan parasite C13776. In regions of Peru where *L.panamensis* co-occurs with *L.wiracocha* Haig, 1974 and *Paralomislongipes* Faxon, 1893, rhizocephalans infest *P.longipes* with a prevalence of 7%, whereas the other two species have not been observed with parasites, suggesting a possible host preference (Arguelles et al. 2020). Our specimen confirms that *L.panamensis* is susceptible to rhizocephalan parasitization, at least at the CRM.

**Paralomisaff.diomedeae (Faxon, 1893)**

Fig. 57G, H

**Reference.**Niemann et al. (2013) for occurrences at Mound 12.

**Material examined.** AD4502: C11137 (**PQ450391**); AD4586: C14950; AD4917: C12795; AD4975: C13788 (**PQ450383**); S0217: C13914.

**Localities.** Mound 12 (~ 987–1000 m), The Thumb (1072 m; this study).

**Remarks.** The voucher specimens above match the description and locality of a *Paralomis* morphospecies that has been observed grazing on microbial mats at Mound 12 (Niemann et al. 2013). This likely undescribed species is morphologically similar to *P.diomedeae* but differs “by the granules on the dorsal carapace surface and the ﻿armature of the chelipeds and walking legs” (Niemann et al. 2013). Our specimens share these notable differences from *P.diomedeae*, which is known from California to the Gulf of Panama (type locality) to Peru, 680–935 m, and has been previously reported from Costa Rica, 770–825 m (MacPherson and Wehrtmann 2010; Hendrickx and López 2020).

#### ﻿Arthropoda | … | Decapoda | Pleocyemata | Anomura | Paguroidea | Parapaguridae

We thank Rafael Lemaitre (U.S. National Museum of Natural History) for identification of these specimens.

***Oncopagurushaigae* (de Saint Laurent, 1972)**

Fig. 58A

**Figure 58.**
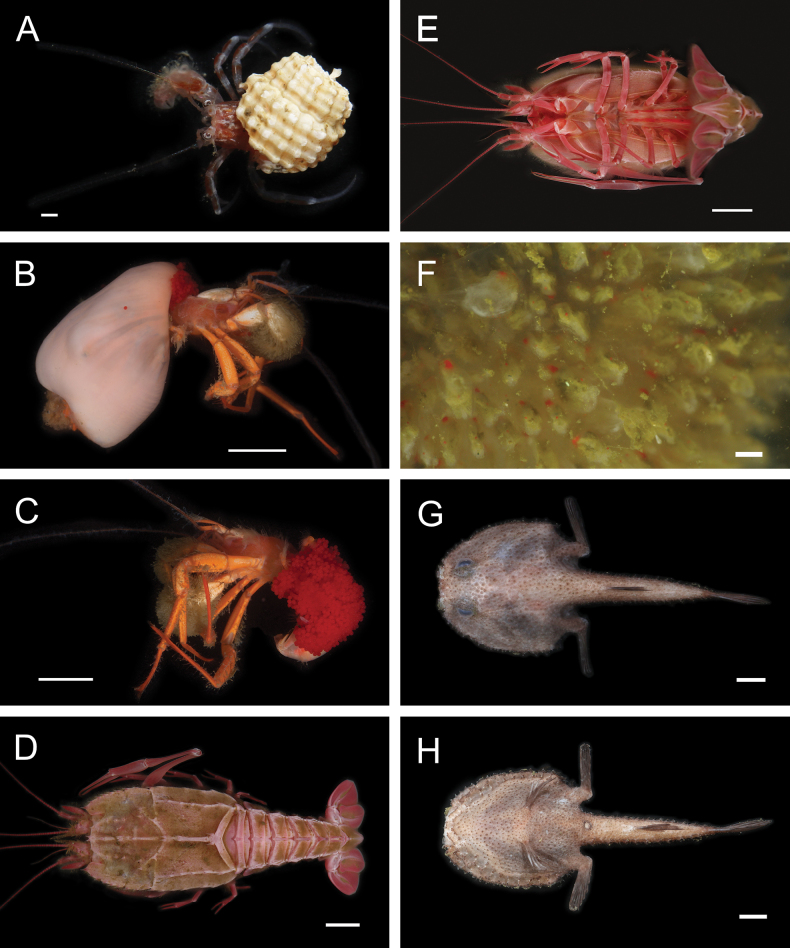
Arthropoda: Decapoda and Chordata, representative live images **A***Oncopagurushaigae* (C13911) **B***Parapagurusforaminosus* (C11210, dorsal view) **C***Parapagurusforaminosus* (C11210, ventral view) **D***Stereomastispacifica* (C13962, dorsal view) **E***Stereomastispacifica* (C13962, ventral view) **F**Pyrosomatidae stet. (BI1641) **G***Dibranchusnudivomer* (UCR-FL-065, dorsal view) **H***Dibranchusnudivomer* (UCR-FL-065, ventral view). Scale bars: 1 mm (**A, F**); 1 cm (**B–E, G, H**).

**Material examined.** S0216: C13911.

**Localities.** Quepos Slide (312 m).

**Distribution.** Eastern Pacific from southern California (type locality) to southern Chile (44°S), 55–993 m (Lemaitre 2014).

**Remarks.** This specimen provides the first record of live color for this species.

***Parapagurusforaminosus* Lemaitre, 1999**

Fig. 58B, C

**Material examined.** AD4590: C11210; AD4591: C13308; AD4916: C12787.

**Localities.** Jacó Scar (~ 1800 m).

**Distribution.** Northern Mexico to Ecuador, including Isla del Coco and the Galápagos Islands (type locality), 915–2807 m (Hendrickx and Ayon-Parente 2009).

**Remarks.** Consistent with previous reports of *P.foraminosus* inhabiting gastropod shells completely covered by actinians (Hendrickx and Ayon-Parente 2009), these specimens were associated with *Paracalliactis* anemones (C11210 with Co2300, C13308 with Co2320, and C12787 with Co2924). C11210 is an ovigerous female.

#### ﻿Arthropoda | … | Decapoda | Pleocyemata | Polychelida | Polychelidae

***Stereomastispacifica* (Faxon, 1893)**

Fig. 58D, E

**Material examined.** S0230: C13962 (**PQ449357**).

**Localities.** Mound Jaguar (1896 m).

**Distribution.** Originally described from the Gulf of Panama (type locality) and several stations off central and southern Mexico, 935–2323 m (Faxon 1893, 1895; Galil 2000). Recorded from northern California to central Chile, 600–3382 m, including previous records from Costa Rica (Galil 2000; Wicksten 2020). The large-bodied larvae occur in midwater to at least 3692 m (Wicksten 1980).

#### ﻿﻿Chordata

﻿**Chordata | Tunicata | Thaliacea | Pyrosomatida**

**Pyrosomatidae stet.**

Fig. 58F

**Material examined.** AD4512: BI1641.

**Localities.** Quepos Slide (400 m).

#### ﻿Chordata | Vertebrata | Actinopterygii | Lophiiformes | Ogcocephalidae

***Dibranchusnudivomer* (Garman, 1899)**

Fig. 58G, H

**Material examined.** S0217: UCR-FL-065 (voucher), ex BI1363 (tissue).

**Localities.** The Thumb (1065 m).

**Distribution.** Originally described from Pacific Colombia, 1271 m (Bradbury 2003), *D.nudivomer* is known from southern Baja California to Peru, 605–1457 m, and has been collected from Pacific Panama at depths of 1101–1314 m (Robertson et al. 2017).

**Remarks.** We thank Ben Frable (Scripps Institution of Oceanography Marine Vertebrate Collection) for the identification of this specimen.

#### ﻿Chordata | Vertebrata | Actinopterygii | Perciformes | Zoarcidae

***Pyrolycusjaco* ﻿Frable, Seid, Bronson & Møller, 2023**

Fig. 59A

**Figure 59.**
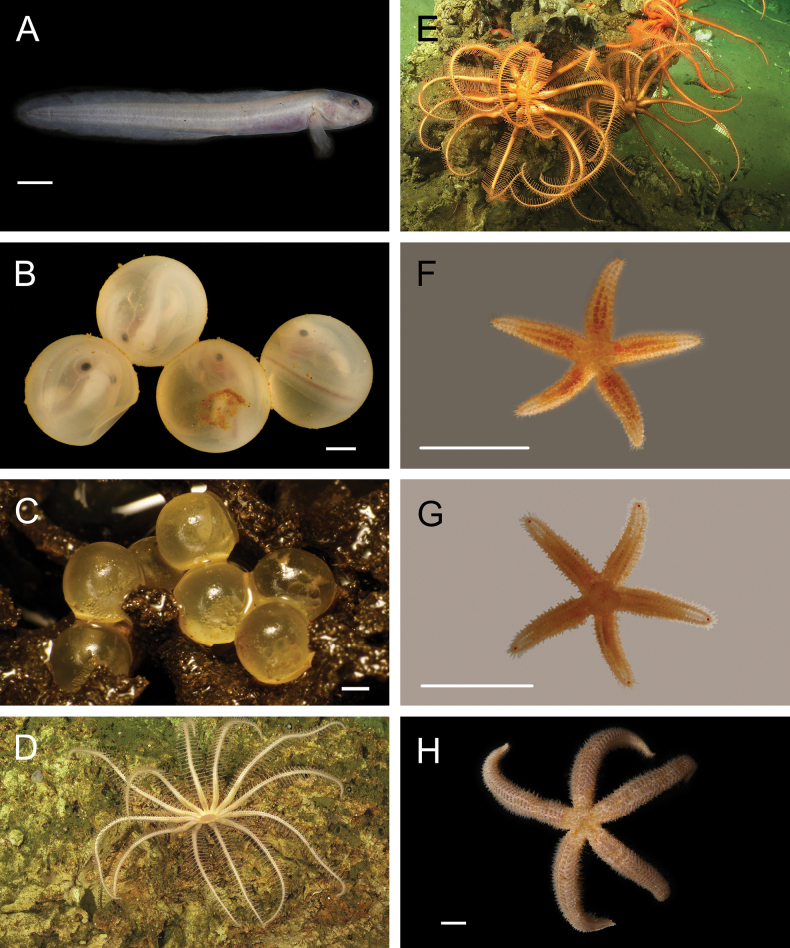
Chordata and Echinodermata: Asteroidea, representative live images **A***Pyrolycusjaco* (BI1159) **B***Acantholiparis* stet. (BI1371, eggs) **C***Paraliparis* stet. (BI1369, eggs) **D***Freyella* stet. (E7365, *in situ*). Credit: ROV SuBastian/Schmidt Ocean Institute **E***Freyella* stet. (E7367, *in situ*). Credit: ROV SuBastian/Schmidt Ocean Institute **F***Ampheraster* gen. inc. (E7325, aboral view) **G***Ampheraster* gen. inc. (E7325, oral view) **H***Pedicellaster* gen. inc. (E7320, aboral view). Scale bars: 1 cm (**A, F–H**); 1 mm (**B, C**).

**Reference.**Frable et al. 2023**.

**Additional material examined.** AD4916: BI1159 (tissue).

**Localities.** Jacó Scar (~ 1604–1854 m; type locality).

**Distribution.** Known only from the CRM seeps.

**Remarks.** Associated with *Lamellibrachiabarhami* and *Escarpiaspicata* tubeworm bushes (Frable et al. 2023).

#### ﻿Chordata | Vertebrata | Actinopterygii | Scorpaeniformes | Liparidae

***Acantholiparis* stet.**

Fig. 59B

**Reference.**Levin and Rouse 2020.

**Localities.** Mound Jaguar (1903 m).

**Remarks.** This cluster of eggs, with visible embryos inside, was associated with a xenophyophore, *Shinkaya* stet., representing one of the first two recorded uses of xenophyophores as nursery habitat by fishes (Levin and Rouse 2020).

***Paraliparis* stet.**

Fig. 59C

**Reference.**Levin and Rouse 2020.

**Localities.** Jacó Scar (1866 m).

**Remarks.** This cluster of eggs was associated with a xenophyophore, *Reticulammina* stet., representing one of the first two recorded uses of xenophyophores as nursery habitat by fishes (Levin and Rouse 2020).

#### ﻿﻿Echinodermata

We list the major clades according to the phylogenetic relationships in Reich et al. (2015).

#### ﻿Echinodermata | Asteroidea

We list entries following the phylogenetic relationships in Linchangco et al. (2017).

#### ﻿Echinodermata | Asteroidea | Brisingida | Freyellidae

***Freyella* stet.**

Fig. 59D, E

**Material examined.** S0230: E7365 (**PQ449392**; arm fragment only), E7366 (**PQ449393**; juvenile), E7367 (**PQ449394**; arm fragment only).

**Localities.** Mound Jaguar (1895–1908 m).

**Remarks.** Morphologically, these specimens are most likely *Freyella*, although *Astrocles* and *Freyellaster* are possible and *Freyella* is not monophyletic (Zhang et al. 2024). The COI sequences were 98.97–99.13% identical to specimens of Freyellacf.fragilissima (Sladen, 1889) from Terre Adélie, Southern Ocean, 1956–2154 m (OR600610.1, OR600611.1), and the Manus Basin, tropical western Pacific, 1708 m (OR600608.1).

#### ﻿Echinodermata | Asteroidea | Forcipulatida | Pedicellasteridae

***Ampheraster* gen. inc.**

Fig. 59F, G

**Material examined.** S0219: E7325 (**PQ449389**; tissue).

**Localities.** Rio Bongo Scar (631 m).

**Remarks.** Juvenile pedicellasterid, likely *Ampheraster* based on spines along the marginals. The closest COI BLASTN results on GenBank were relatively distant, e.g., *Pedicellastermagister* Fisher, 1923 from British Columbia (HM473941.1, 87.80% identity).

***Pedicellaster* gen. inc.**

Figs 59H, 60A

**Figure 60.**
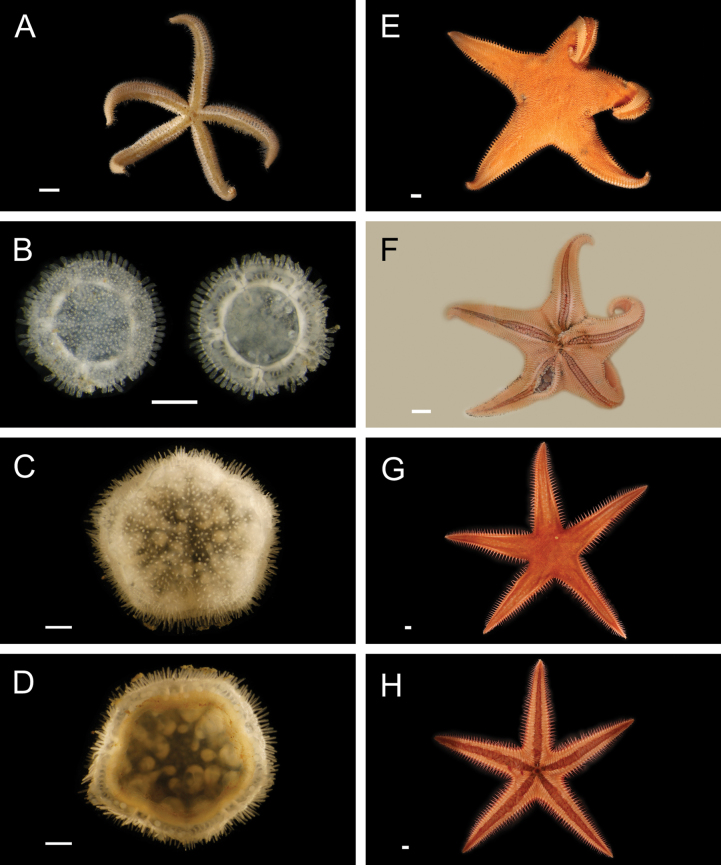
Echinodermata: Asteroidea, representative live images **A***Pedicellaster* gen. inc. (E7320, oral view) **B***Xyloplaxprincealberti* (E7224, aboral and oral views) **C***Xyloplax* sp. SIO_BIC_E7369 (E7369, aboral view) **D***Xyloplax* sp. SIO_BIC_E7369 (E7369, oral view) **E***Tethyastercanaliculatus* sp. inc. (E7036, aboral view) **F***Tethyastercanaliculatus* sp. inc. (E7036, oral view) **G***Thrissacanthiaspenicillatus* (E7246, aboral view) **H***Thrissacanthiaspenicillatus* (E7246, oral view). Scale bars: 1 cm (**A, E–H**); 1 mm (**B–D**).

**Material examined.** S0219: E7320 (**PQ449388**; tissue).

**Localities.** Rio Bongo Scar (609 m).

**Remarks.** The closest COI BLASTN result on GenBank was a specimen of *Ampherastermarianus* (Ludwig, 1905) from British Columbia (HM542909.1, 94.28% identity).

#### ﻿Echinodermata | Asteroidea | Velatida | Xyloplacidae

***Xyloplaxprincealberti* Payne et al., 2023**

Fig. 60B

**Reference.**Payne et al. 2023**.

**Localities.** Jacó Scar (1845 m).

**Distribution.** Juan de Fuca Ridge (type locality) to the CRM seeps, on experimental wood and bone deployments and on a *Ridgeapiscesae* Jones, 1985 tubeworm bush, 1845–2421 m (Payne et al. 2023).

***Xyloplax* sp. SIO_BIC_E7369**

Fig. 60C, D

**Material examined.** AD4972: E7223; AD4976: E7240, E7241; S0230: E7369.

**Localities.** Jacó Scar (1845–1887 m), Mound Jaguar (2000 m).

**Remarks.** An undescribed species, associated with naturally occurring and experimentally deployed wood, to be described in a separate work.

#### ﻿Echinodermata | Asteroidea | Paxillosida | Astropectinidae

***Tethyastercanaliculatus* (A.H. Clark, 1916) sp. inc.**

Fig. 60E, F

**Material examined.** AD4913: E7036.

**Localities.** Jacó Scar (1878 m).

**Remarks.** Further morphological comparison is needed. *T.canaliculatus* was originally described from the Gulf of California at 73 m (Clark 1916a) and has been reported from Mexico to Peru at depths of 23–300 m (Alvarado and Solís-Marin 2013). If confirmed as *T.canaliculatus*, the CRM specimen would represent a new depth record for the species.

***Thrissacanthiaspenicillatus* (Fisher, 1905)**

Fig. 60G, H

**Material examined.** AD4975: E7246 (16S: **PQ304658**).

**Localities.** Mound 12 (1000 m).

**Distribution.** Originally described from the Coronados Islands off northern Mexico, 969–1167 m (Fisher 1905), *T.penicillatus* is known from Washington to the Gulf of California at depths of 55–1503 m (Fisher 1911; Hendrickx et al. 2011; Alvarado and Solís-Marin 2013).

**New records.** The CRM specimen represents a new southern record for the species. Its distribution may extend farther south, pending an unpublished report of *T.penicillatus* from Peru (“﻿Morales-Montecinos (in preparation)” in Hooker et al. (2014)) and further identification of a specimen of *Thrissacanthias* sp. (MZUCR) from Pacific Costa Rica (Alvarado et al. 2017).

**Remarks.** This specimen was the host of at least six *Macellicephala* scaleworms (A9775) associated with the ambulacral groove. A COI sequence could not be obtained, but the closest 16S BLASTN results on GenBank showed 100.00% identity to reference sequences of *T.penicillatus* from California: EU722968.1 (CASIZ 115075; off Mendocino County, 969 m) and FJ177631.1 (SIO-BIC E3857, San Diego Trough, 1215 m).

#### ﻿Echinodermata | Asteroidea | Valvatida | Goniasteridae

***Ceramaster* gen. inc.**

Fig. 61A, B

**Figure 61.**
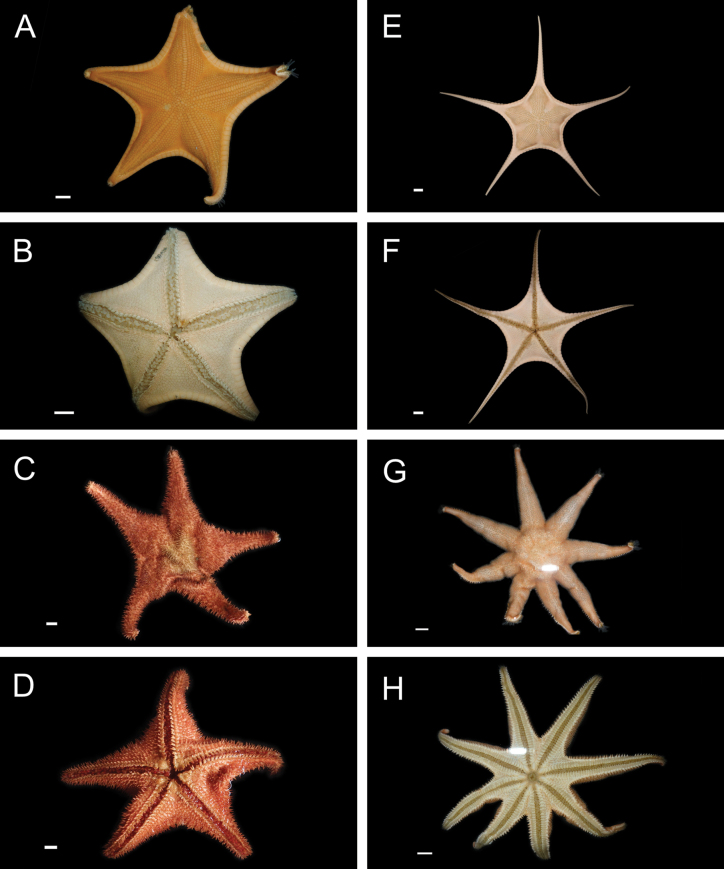
Echinodermata: Asteroidea, representative live images **A***Ceramaster* gen. inc. (E7048, aboral view) **B***Ceramaster* gen. inc. (E7048, oral view) **C***Evoplosomaclaguei* (E7081, aboral view) **D***Evoplosomaclaguei* (E7081, oral view) **E***Nymphasterdiomedeae* (E7235, aboral view) **F***Nymphasterdiomedeae* (E7235, oral view) **G***Crossasterborealis* gen. et sp. inc. (E7050, aboral view) **H***Crossasterborealis* gen. et sp. inc. (E7050, oral view). Scale bars: 1 cm.

**Material examined.** AD4917: E7048.

**Localities.** Mound 12 (997 m).

**Remarks.** Most likely *Ceramaster*, but internal dissection is required to exclude *Mediaster*.

***Evoplosomaclaguei* Mah, Nizinski & Lundsten, 2010 sp. inc.**

Fig. 61C, D

**Material examined.** AD4923: E7081.

**Localities.** Parrita Seep (~ 1037–1108 m).

**Distribution.***Evoplosomaclaguei* is known from the Juan de Fuca Ridge, Rodriguez Seamount (type locality) off southern California, and Islas Tres Marías off central Mexico; 730–2406 m (Mah et al. 2010).

**Remarks.** If confirmed as *E.claguei*, the CRM specimen would represent a new southern record for the species.

***Nymphasterdiomedeae* Ludwig, 1905**

Fig. 61E, F

**Reference.**Mah (2016) for morphological examination of specimen E4386.

**Material examined.** AD4504: E4386 (**PQ449001**); AD4907: E7028; AD4917: E7051; AD4923: E7067, E7069; AD4975: E7235.

**Localities.** Mound 12 (994–1001 m; this study), Mound 11 (~ 1004–1011 m), Parrita Seep (1097 m; this study).

**Distribution.** Originally described from 2149 m off Isla del Coco (Ludwig 1905) and recorded from the Gulf of California to Panama and the Galápagos Islands, 702–1618 m (Wehrtmann and Cortés 2009b; Alvarado et al. 2010; Hendrickx et al. 2011; Alvarado and Solís-Marin 2013; Mah 2016).

#### ﻿Echinodermata | Asteroidea | Valvatida | Solasteridae

***Crossasterborealis* Fisher, 1906 gen. et sp. inc.**

Fig. 61G, H

**Material examined.** AD4917: E7050.

**Localities.** Mound 12 (1000 m).

**Remarks.** Possibly *Crossasterborealis*, which was originally described from Alaska and the Bering Sea, 421–1805 m (Fisher 1906) and is known across the northern Pacific from Japan to southern California (Hayashi 1939). If confirmed as *C.borealis*, the CRM specimen would represent a new southern record for the species.

#### ﻿Echinodermata | Asteroidea | Valvatida | Caymanostellidae

***Caymanostella* sp. SIO_BIC_E11440**

Fig. 62A

**Figure 62.**
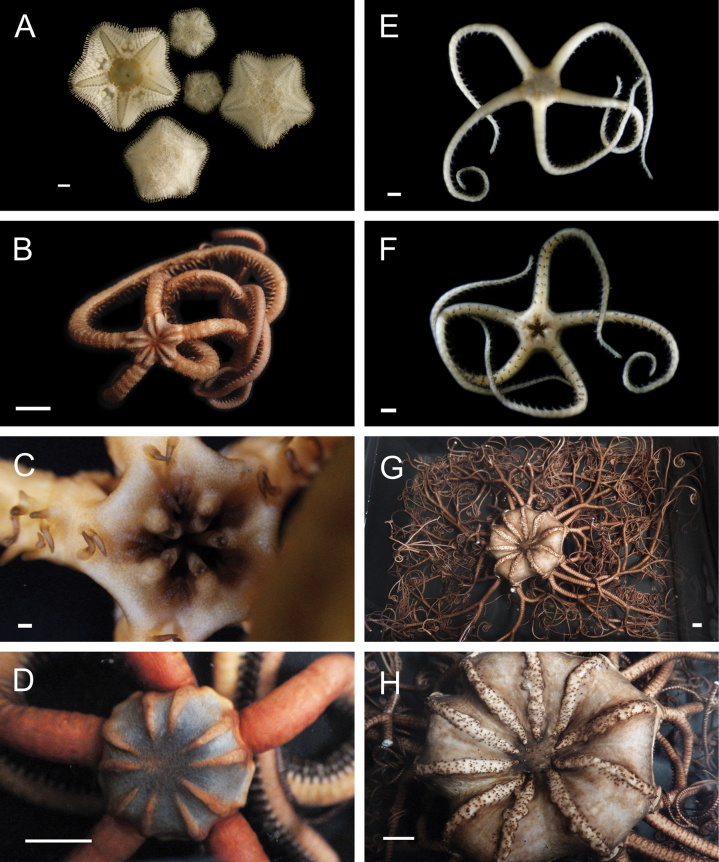
Echinodermata: Asteroidea and Ophiuroidea, representative live images **A***Caymanostella* sp. SIO_BIC_E11440 (E11440) and *Caymanostella* sp. SIO_BIC_E11441 (E7075) as a mixed lot **B***Asteroschemasublaeve* (E7073, dorsal view) **C***Asteroschemasublaeve* (E7073, ventral view) **D***Ophiocreascarnosus* (E7072, dorsal view) **E***Ophiocreascarnosus* (E7035, dorsal view) **F***Ophiocreascarnosus* (E7035, ventral view) **G**Gorgonocephaluscf.pustulatum (E7065, dorsal wide view) **H**Gorgonocephaluscf.pustulatum (E7065, dorsal view detail). Scale bars: 1 mm (**A, C, E, F**); 1 cm (**B, D, G, H**).

**Material examined.** AD4922: MZUCR-ECH2403 (formerly SIO-BIC E11440; PP627125); AD4972: E7226; AD4976: E7238, E7242.

**Localities.** Mound 12 (1002 m), Jacó Scar (1845–1887 m).

**Remarks.** An undescribed species, associated with naturally occurring and experimentally deployed wood, to be described in a separate work (Shen et al. (2024), in press at the time of this work’s acceptance).

***Caymanostella* sp. SIO_BIC_E11441**

Fig. 62A

**Material examined.** AD4503: E4383, E11442; AD4587: E4549, E11221; AD4922: E7075; AD4988: E7289, E7290, E11441 (PP627110), MZUCR-ECH2402.

**Localities.** Mound 12 (990–1002 m), Mound 11 (1010 m).

**Remarks.** An undescribed species, associated with naturally occurring and experimentally deployed wood, to be described in a separate work (Shen et al. (2024), in press at the time of this work’s acceptance).

#### ﻿Echinodermata | Ophiuroidea

We list entries following the phylogeny-based higher taxonomy in O’Hara et al. (2018).

#### ﻿Echinodermata | Ophiuroidea | Euryophiurida | Euryalida | Euryalidae

***Asteroschemasublaeve* Lütken & Mortensen, 1899**

Fig. 62B, C

**Material examined.** AD4916: E7060 (arm fragment only); AD4916: E7061 (**PQ435540**); AD4923: E7073 (**PQ435544**).

**Localities.** Parrita Seep (~ 1034–1108 m), Jacó Scar (1604–1710 m).

**Distribution.** Originally described from several stations in the central eastern Pacific from the vicinity of Islas Marías, Mexico, to Panama, 605–1681 m (Lutken and Mortensen 1899), and also reported from the Gulf of California (Granja-Fernández et al. 2015a).

***Ophiocreascarnosus* Lyman, 1879**

Fig. 62D–F

**Material examined.** AD4914: E7035; AD4923: E7072 (**PQ435543**); S0218: E7318, E7322.

**Localities.** Parrita Seep (1052 m), Parrita Scar (1431–1589 m), Jacó Scar (1632 m).

**Distribution.** Originally described from southern Chile (75°S), 320 m (Lyman 1879; Murray and Thomson 1885). Museum records on GBIF include additional occurrences as far north as central Chile (26°S) at depths 175–983 m (GBIF.org 2024).

**New records.** The CRM specimens represent new northern records and a new maximum depth record for this species (specimen E7035 from 1632 m).

**Remarks.** Associated with corals: *Callogorgia* (E7072 with MZUCR 3140; E7322 with a similar host, not vouchered), *Swiftia* (E7318 with Co3080), or Isididae (E7035 with MZUCR 3547).

#### ﻿Echinodermata | Ophiuroidea | Euryophiurida | Euryalida | Gorgonocephalidae

**Gorgonocephaluscf.pustulatum (H.L. Clark, 1916)**

Figs 62G, H, 63A

**Figure 63.**
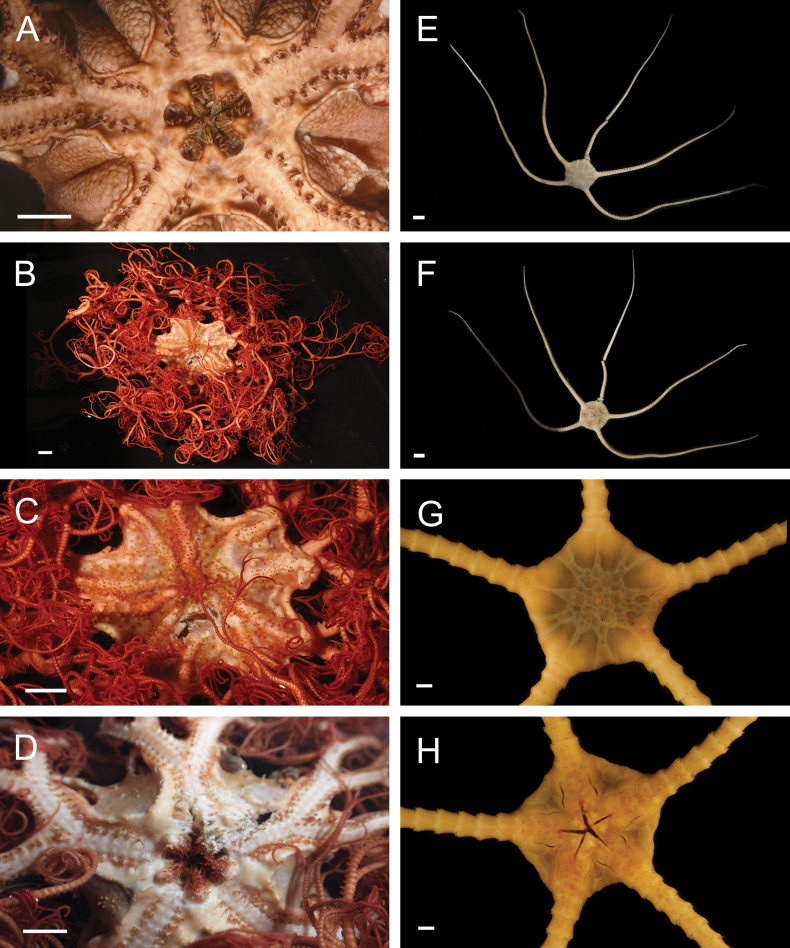
Echinodermata: Ophiuroidea, representative live images **A**Gorgonocephaluscf.pustulatum (E7065, ventral view detail) **B***Gorgonocephalus* stet. (E7064, dorsal wide view) **C***Gorgonocephalus* stet. (E7064, dorsal view detail) **D***Gorgonocephalus* stet. (E7064, ventral view detail) **E***Ophiomusalymani* (E7045, dorsal view) **F***Ophiomusalymani* (E7045, ventral view) **G***Ophiomusalymani* (E7082, dorsal view detail) **H***Ophiomusalymani* (E7082, ventral view detail). Scale bars: 1 cm (**A–F**); 1 mm (**G, H**).

**Material examined.** AD4504: E4387 (**PQ435534**); AD4922: E7065 (**PQ435542**).

**Localities.** Mound 12 (964 m), Mound 11 (~ 1004–1011 m).

**Remarks.** Likely an undescribed species, requiring further morphological comparison to the western Pacific *Gorgonocephaluspustulatum*, originally described from the Bass Strait, 183–549 m (Clark 1916b).

***Gorgonocephalus* stet.**

Fig. 63B–D

**Material examined.** AD4922: E7064 (**PQ435541**).

**Localities.** Mound 12 (965 m).

#### ﻿Echinodermata | Ophiuroidea | Euryophiurida | Ophiurida | Ophiomusina | Ophiomusaidae

***Ophiomusalymani* (Wyville Thomson, 1873)**

Fig. 63E–H

**Material examined.** AD4916: E7045 (**PQ435538**); AD4922: E7082 (**PQ435546**).

**Localities.** Mound 12 (~ 966–996 m), Jacó Scar (1674 m).

**Distribution.** Originally described from the west coast of Ireland (Thomson 1873) and widely distributed (Baker 2016). *O.lymani* has been reported from the Caribbean and Pacific margins of Central America, including the Galápagos Islands and Isla del Coco, over depths of 51–4700 m (Alvarado et al. 2010; Cortés 2012; Alvarado and Solís-Marin 2013; Granja-Fernández et al. 2023).

#### ﻿Echinodermata | Ophiuroidea | Euryophiurida | Ophiurida | Ophiomusina | Ophiosphalmidae

***Ophiosphalmaglabrum* (Lütken & Mortensen, 1899)**

Fig. 64A, B

**Figure 64.**
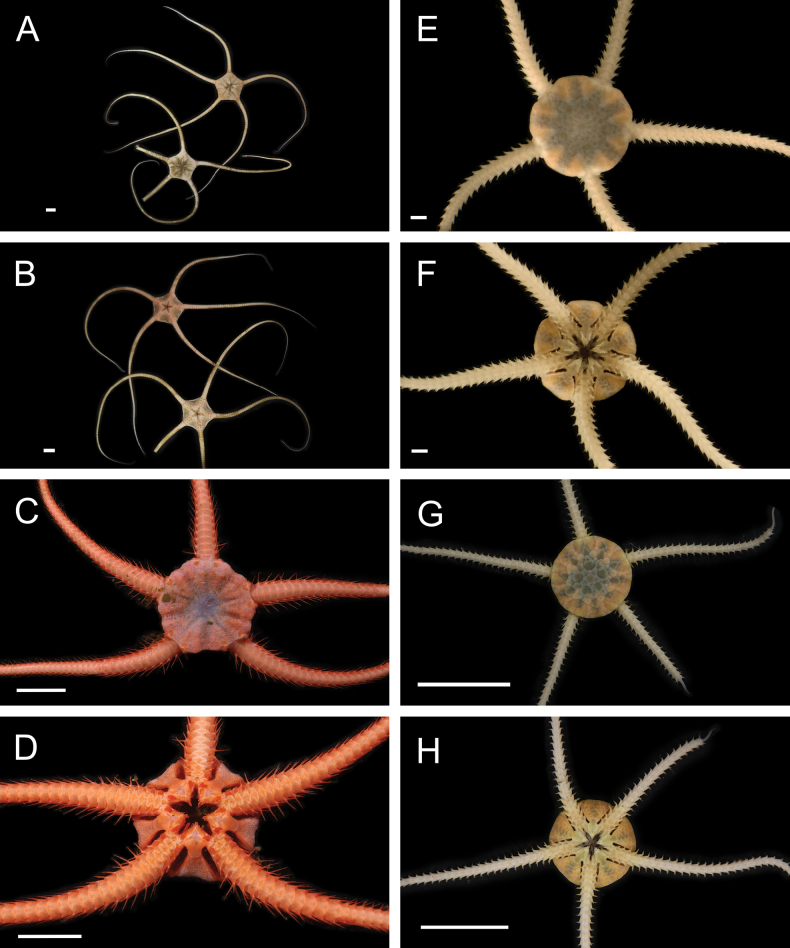
Echinodermata: Ophiuroidea, representative live images **A***Ophiosphalmaglabrum* (E7046, dorsal view) **B***Ophiosphalmaglabrum* (E7046, ventral view) **C***Ophiuraflagellata* (E7315, dorsal view) **D***Ophiuraflagellata* (E7315, ventral view) **E**Ophiuracf.scutellata (E7231, dorsal view) **F**Ophiuracf.scutellata (E7231, ventral view) **G**Ophiuroglyphacf.meridionalis (E7321, dorsal view) **H**Ophiuroglyphacf.meridionalis (E7321, ventral view). Scale bars: 1 cm (**A–D, G, H**); 1 mm (**E, F**).

**Material examined.** AD4916: E7046 (**PQ435539**).

**Localities.** Jacó Scar (~ 1854–1887 m).

**Distribution.** Originally described from several stations in the central eastern Pacific, from the southern Gulf of California to Panama, 1789–4082 m (Lutken and Mortensen 1899), and reported from the Clarion-Clipperton Zone, 4986 m (Christodoulou et al. 2019, 2020). The synonymization of *Ophiomusiummultispinum* H.L. Clark, 1911 (described from Washington, 1604 m depth) and *Ophiomusiumfimbriatum* Koehler, 1922 (described from the Molucca Sea, Indonesia, 2011 m depth) under *Ophiosphalmaglabrum* extends its distribution into the northeastern Pacific and western Pacific, with a total depth range of 878–5203 m (Clark 1913; Baker 2016; Granja-Fernández et al. 2023).

#### ﻿Echinodermata | Ophiuroidea | Euryophiurida | Ophiurida | Ophiurina | Ophiopyrgidae

***Ophiuraflagellata* (Lyman, 1878)**

Fig. 64C, D

**Material examined.** S0217: E7315.

**Localities.** The Thumb (1066 m).

**Distribution.** Originally described from Sagami Bay, Japan, 622 m (Lyman 1878; Murray and Thomson 1885) and widely distributed in the Pacific and Indian Oceans, 96–2330 m (Granja-Fernández et al. 2023). The CRM specimens are morphologically consistent with the synonym *Gymnophiuracoerulescens* Lütken & Mortensen, 1899, which was originally described from the Mexican Pacific, 1681–1820 m (Lutken and Mortensen 1899), and which likely warrants resurrection (Granja-Fernández et al. 2023).

**Remarks.***Ophiura* is polyphyletic with extensive revision required; *O.flagellata* is placed within ﻿Ophiopyrgidae (O’Hara et al. 2017).

**Ophiuracf.scutellata (Lütken & Mortensen, 1899)**

Fig. 64E, F

**Material examined.** AD4509: E4404; AD4590: E4562 (**PQ450406**); AD4912: E7030, E7031, E7032; AD4922: E7066; AD4971: E7230; AD4973: E7231 (**PQ435547**); AD4974: E7233; AD4987: E7272.

**Localities.** Mound 12 (965–1010 m), Jacó Scar (1715–1842 m).

**Remarks.** Likely an undescribed species, to be further compared to *Ophiurascutellata*, described from Islas Marías, Mexico, 1244 m (Lutken and Mortensen 1899).

***Ophiuroglypha* cf**. ***meridionalis* (Lyman, 1879)**

Fig. 64G, H

**Material examined.** AD4506: E7978; AD4586: E4547 (**PQ435536**); AD4587: E4551 (**PQ449362**), E4552 (**PQ449363**), E7923; AD4907: E7027; AD4977: E7239; AD4985: E7273; S0217: E7321.

**Localities.** Mound 12 (~ 982–1002 m), Parrita Seep (~ 1030–1033 m), The Thumb (1071 m), Jacó Scar (1783 m).

**Remarks.** Likely an undescribed species, to be further compared to *Ophiuroglyphameridionalis*, described from the South Atlantic off Río de la Plata, Argentina, 1097 m (Lyman 1879; Murray and Thomson 1885).

**Ophiopyrgidae sp. SIO_BIC_E7310**

Fig. 65A, B

**Figure 65.**
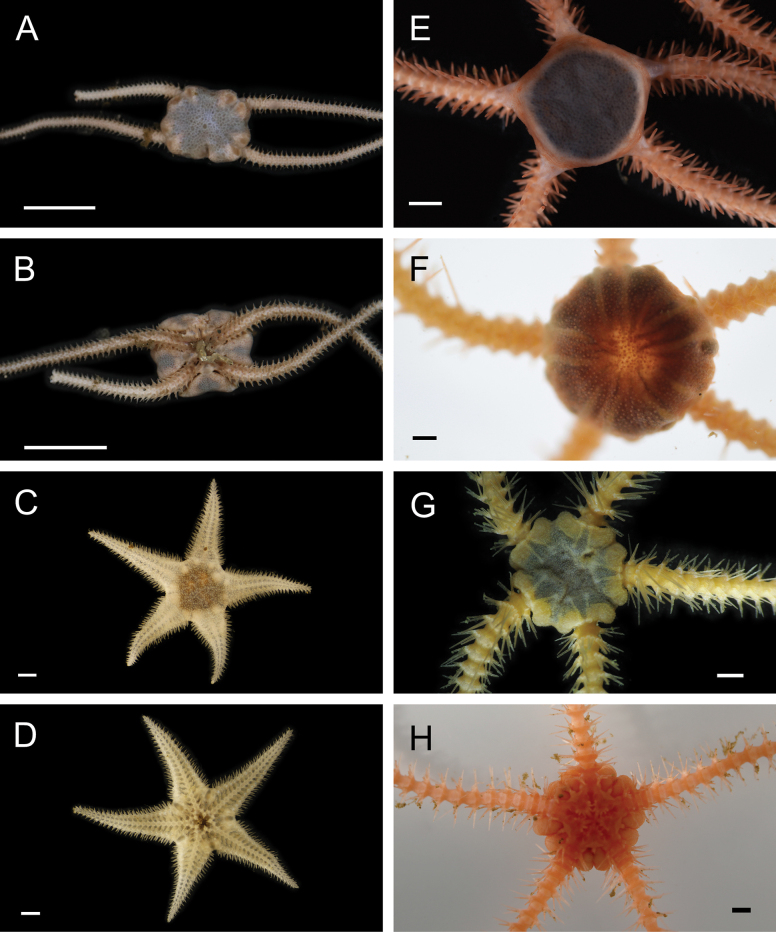
Echinodermata: Ophiuroidea, representative live images **A**Ophiopyrgidae sp. SIO_BIC_E7310 (E7310, dorsal view) **B**Ophiopyrgidae sp. SIO_BIC_E7310 (E7310, ventral view) **C**Ophiambixcf.epicopus (E7291, dorsal view) **D**Ophiambixcf.epicopus (E7291, ventral view) **E***Ophiophruraliodisca* (E4561) **F***Ophiacanthainconspicua* (E7245) **G***Ophiacanthamoniliformis* (E4390, dorsal view) **H***Ophiacanthamoniliformis* (E7323, ventral view). Scale bars: 1 cm (**A, B**); 1 mm (**C–H**).

**Material examined.** AD4990: E7293; S0214: E7310 (**PQ435550**).

**Localities.** Parrita Seep (1400 m), Jacó Scar (1803 m).

**Remarks.** An undescribed species, likely also an undescribed genus.

#### ﻿Echinodermata | Ophiuroidea | Ophintegrida | Ophioscolecida | Ophioscolecidae

***Ophiambix* cf**. ***epicopus* Paterson & Baker, 1988**

Fig. 65C, D

**Material examined.** AD4508: E7979; AD4587: E4550 (**PQ449361**); AD4906: E7023; AD4922: E7076; AD4988: E7291.

**Localities.** Mound 12 (996–1002 m), Mound 11 (1010 m), Parrita Seep (~ 1401–1419 m).

**Remarks.** Associated with naturally occurring wood falls. Likely an undescribed species, to be further compared to *Ophiambixepicopus*, described from the vicinity of the Kermadec Islands, South Pacific, 530–567 m (Paterson and Baker 1988).

***Ophiophruraliodisca* H.L. Clark, 1911**

Fig. 65E

**Material examined.** AD4589: E4561; S0219: E7980 (**PQ435551**).

**Localities.** Rio Bongo Scar (606 m), Mound 12 (997 m).

**Distribution.** Originally described from central Japan, off Omaezaki, 869–924 m (Clark 1911), and recorded from New Zealand, 1421 m (GenBank KU895351.1 (Hugall et al. 2016)). GBIF includes additional occurrences from western Pacific seamounts, 678–1620 m (GBIF.org 2022b).

**New records.** The CRM specimens represent new records of this species in the eastern Pacific.

#### ﻿Echinodermata | Ophiuroidea | Ophintegrida | Ophiacanthida | Ophiacanthina | Ophiacanthidae

***Ophiacanthainconspicua* Lütken & Mortensen, 1899**

Fig. 65F

**Material examined.** AD4913: E7041 (**PQ435552**); AD4972: E7245.

**Localities.** Jacó Scar (~ 1751–1896 m).

**Distribution.** Originally described and currently known only from several stations off Pacific Panama, 1430–1865 m, and the Galápagos Islands, 1251–1618 m (Lutken and Mortensen 1899).

***Ophiacanthamoniliformis* Lütken & Mortensen, 1899**

Fig. 65G, H

**Material examined.** AD4506: E4390 (**PQ450392**); AD4923: E7078; S0219: E7323.

**Localities.** Rio Bongo Scar (606 m), Parrita Seep (~ 1030–1108 m).

**Distribution.** Originally described from several stations off Acapulco and Islas Marías, Mexico, 902–1244 m (Lutken and Mortensen 1899) and recorded north to the San Pedro Basin, southern California, 750 m (Hartman 1955; Hartman and Barnard 1958), with an overall reported depth range of 377–1244 m (Granja-Fernández et al. 2023). GBIF includes additional occurrences from Panama and Ecuador, 55–137 m (GBIF.org 2022a).

**New records.** Pending verification of the GBIF records, our CRM specimens represent new southern records for this species.

***Ophiolebes* sp. SIO_BIC_E4382**

Fig. 66A

**Figure 66.**
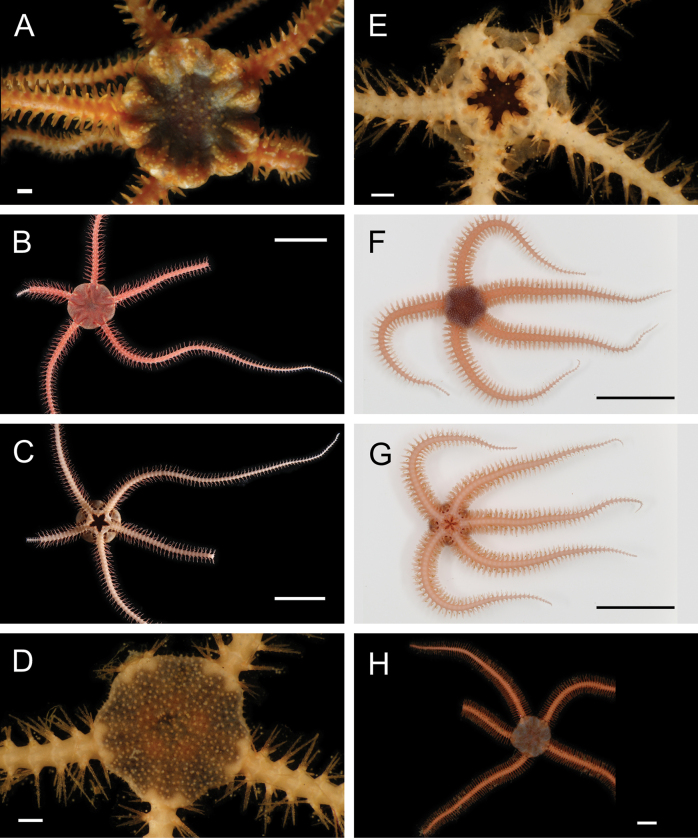
Echinodermata: Ophiuroidea, representative live images **A***Ophiolebes* sp. SIO_BIC_E4382 (E4382, dorsal view) **B***Ophiolimna* sp. SIO_BIC_E7303 (E7303, dorsal view) **C***Ophiolimna* sp. SIO_BIC_E7303 (E7303, ventral view) **D***Ophiomitrapartita* (E7232, dorsal view) **E***Ophiomitrapartita* (E7232, ventral view) **F***Ophiopristis* sp. SIO_BIC_E7074 (E7074, dorsal view) **G***Ophiopristis* sp. SIO_BIC_E7074 (E7074, ventral view) **H***Ophiotreta* sp. SIO_BIC_E4406 (E4406, dorsal view). Scale bars: 1 mm (**A, D, E**); 1 cm (**B, C, F, G**).

**Material examined.** AD4501: E4382; AD4503: E4385 (**PQ435533**).

**Localities.** Mound 12 (~ 967–995 m).

**Remarks.** An undescribed species. Associated with the antipatharian coral *Lillipathesritamariae* (E4382 with Co2293; E4385 with Co2267).

***Ophiolimna* sp. SIO_BIC_E7303**

Fig. 66B, C

**Material examined.** AD4974: E7234; S0213: E7303 (**PQ435549**).

**Localities.** Jacó Summit (763 m), Mound 12 (992 m).

**Remarks.** An undescribed species.

#### ﻿Echinodermata | Ophiuroidea | Ophintegrida | Ophiacanthida | Ophiacanthina | Ophiotomidae

***Ophiomitrapartita* Lütken & Mortensen, 1899**

Fig. 66D, E

**Material examined.** AD4973: E7232 (**PQ435548**).

**Localities.** Jacó Scar (1887 m).

**Distribution.** Originally described and currently known only from Islas Marías, Mexico, 1236 m (Lutken and Mortensen 1899).

**New records.** The CRM specimen represents a new southern record and a new maximum depth record for this species.

***Ophiopristis* sp. SIO_BIC_E7074**

Fig. 66F, G

**Material examined.** AD4922: E7074 (**PQ435545**).

**Localities.** Mound 12 (~ 967 m).

**Remarks.** An undescribed species. Associated with the antipatharian coral *Lillipathesritamariae*.

***Ophiotreta* sp. SIO_BIC_E4406**

Figs 66H, 67A

**Figure 67.**
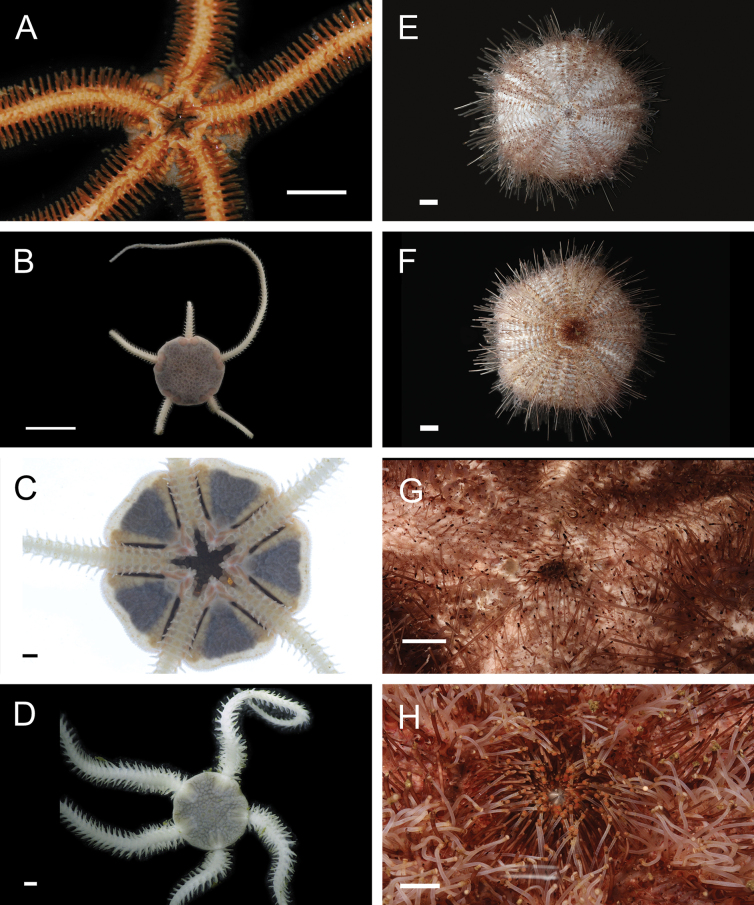
Echinodermata: Ophiuroidea and Echinoidea, representative live images **A***Ophiotreta* sp. SIO_BIC_E4406 (E4406, ventral view) **B***Ophioleucegracilis* (E7040, dorsal view) **C***Ophioleucegracilis* (E7244, ventral view) **D***Amphiura* sp. SIO_BIC_E4397 (E4397, dorsal view) **E***Araeosomaleptaleum* (E7319, aboral view) **F***Araeosomaleptaleum* (E7319, oral view) **G***Araeosomaleptaleum* (E7021, detail of aboral view) **H***Araeosomaleptaleum* (E7021, detail of oral view). Scale bars: 1 cm (**A, B, E–H**); 1 mm (**C, D**).

**Material examined.** AD4510: E4406 (**PQ435535**).

**Localities.** Jacó Summit (~ 741–744 m).

**Remarks.** An undescribed species.

#### ﻿Echinodermata | Ophiuroidea | Ophintegrida | Ophioleucida | Ophioleucidae

***Ophioleucegracilis* Belyaev & Litvinova, 1976**

Fig. 67B, C

**Material examined.** AD4913: E7040; AD4916: E7059; AD4971: E7229; AD4972: E7244 (tissue).

**Localities.** Jacó Scar (1710–1751 m, ~ 1817–1896 m).

**Distribution.** Known as a bathyal (2000–3000 m) Pacific species (Stöhr et al. 2012) and previously reported from the eastern Clarion-Clipperton Zone, 2882 m (Christodoulou et al. 2019, 2020).

**New records.**CRM specimen E7059 from 1710 m represents a new minimum depth record for this species.

#### ﻿Echinodermata | Ophiuroidea | Ophintegrida | Amphilepidida | Gnathophiurina | Amphiuridae

***Amphiuraseminuda* Lütken & Mortensen, 1899**

**Material examined.** AD4587: E7924 (**PQ449044**; no images available).

**Localities.** Mound 12 (996 m).

**Distribution.** Originally described from the southern Gulf of California off Mazatlán, 1558 m (Lutken and Mortensen 1899). Recorded from the San Pedro Basin off southern California, 750 m (Hartman 1955; Hartman and Barnard 1958), to the ﻿Gulf of Tehuantepec (Granja-Fernández et al. 2015b), and off the Tuamotu Archipelago, south-central Pacific, 1476 m (Clark 1917).

***Amphiura* sp. SIO_BIC_E4397**

Fig. 67D

**Material examined.** AD4508: E4397; AD4586: E4563; AD4907: E7033; AD4912: E7029 (**PQ435537**).

**Localities.** Mound 12 (~ 990 m), Jacó Scar (1842 m), Parrita Seep (~ 1400 m).

**Remarks.** An undescribed species.

#### ﻿Echinodermata | Echinoidea | Echinothurioida | Echinothuriidae

***Araeosomaleptaleum* A. Agassiz & H.L. Clark, 1909**

Fig. 67E–H

**Reference.** Mongiardino Koch et al. (2018) for phylogenomic analysis.

**Material examined.** AD4923: E7021 (transcriptome: SRR7513578); AD4984: E7287; S0218: E7319 (**PQ449387**).

**Localities.** Mound 12 (964 m; this study), Parrita Seep (1091 m), Parrita Scar (1271 m; this study).

**Distribution.** Known from 740–1063 m along the Pacific margin of Panama (type locality: Mariato Point, 1063 m (Agassiz and Clark 1909)) and the Galápagos seamounts (Mooi et al. 2004; Lessios 2005). GBIF includes occurrences from seamounts off central California, 1093–1106 m (identification confirmed by R. Mooi) (Blum and Fong 2016a, 2016b).

**New records.**CRM specimen E7319 from 1271 m represents a new maximum depth record for this species.

#### ﻿Echinodermata | Echinoidea | Pedinoida | Pedinidae

**Caenopedinacf.hawaiiensis H.L. Clark, 1912**

Fig. 68A, B

**Figure 68.**
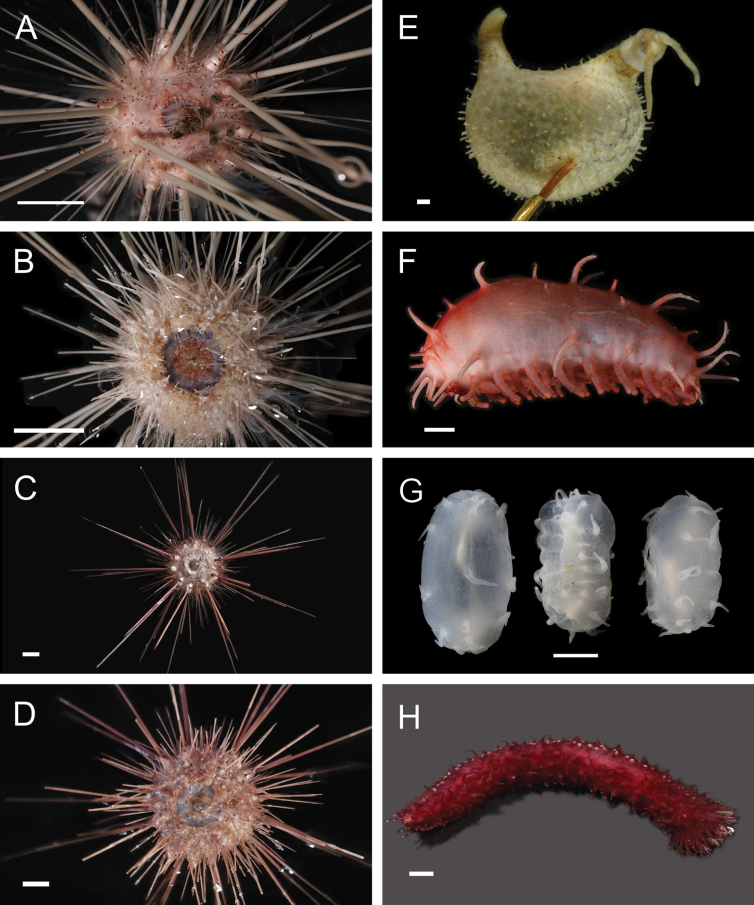
Echinodermata: Echinoidea and Holothuroidea, representative live images **A***Caenopedinadiomedeae* (E7304, aboral view) **B***Caenopedinadiomedeae* (E7304, oral view) **C**Caenopedinacf.hawaiiensis (E7047, aboral view) **D**Caenopedinacf.hawaiiensis (E7047, oral view) **E***Ypsilothuriabitentaculata* (E4400) **F**Deimatidae stet. (E7335, adult) **G**Deimatidae stet. (E7336, young) **H**Bathyplotescf.moseleyi (E7037). Scale bars: 1 cm (**A–D, F–H**); 1 mm (**E**).

**Reference.** Mongiardino Koch et al. (2018) for phylogenomic analysis.

**Material examined.** AD4507: E4396 (**PQ450394**); AD4913: E7020 (transcriptome: SRR7513589); AD4916: E7047.

**Localities.** Jacó Scar (1853–1889 m), Parrita Scar (~ 1659–1667 m; this study).

**Distribution.***C.hawaiiensis* was originally described from 468–1977 m off the Hawaiian Islands (Clark 1912). GBIF includes occurrences from New Zealand, 392–919 m (Mills and Mackay 2019). Identification of these records and additional records spanning 380–1140 m depth were confirmed by Owen Anderson, National Institute of Water and Atmospheric Research (pers. comm. 22 November 2020). Pending genetic data from the type locality, the specimens from the CRM seeps would represent the first records of this species in the eastern Pacific.

***Caenopedinadiomedeae* Mortensen, 1939**

Fig. 68C, D

**Material examined.** AD4984: E7288; S0213: E7304 (**PQ449379**).

**Localities.** Jacó Summit (759 m), Mound 12 (966 m).

**Distribution.** Originally described from 850 m from the Gulf of Panama (Downey 1968) and reported from 723–933 m in the Gulf of Chiriqui near the Costa Rican border (Lessios 2005).

#### ﻿Echinodermata | Holothuroidea

We list entries following the phylogenetic relationships in Miller et al. (2017) and Mongiardino Koch et al. (2023).

#### ﻿Echinodermata | Holothuroidea | Dendrochirotida | Ypsilothuriidae

***Ypsilothuriabitentaculata* (Ludwig, 1893)**

Fig. 68E

**Material examined.** AD4509: E4400.

**Localities.** Jacó Scar (~ 974–1856 m).

**Distribution.** Originally described from several stations in the eastern Pacific from the Galápagos Islands to central Mexico, 1236–4082 m (Ludwig 1893, 1894), this species is considered circum-Pacific from 135–4000 m, with subspecies or varieties also occurring in the Atlantic and Caribbean (Pawson 1965, 1970; Martins and Tavares 2018). In the eastern Pacific, *Y.bitentaculata* is distributed from central California along the coast of Mexico south to Ecuador (Massin and Hendrickx 2011), including previous reports from Isla del Coco (Wehrtmann and Cortés 2009b) and from 1430 m off northern Panama (Ludwig 1894).

#### ﻿Echinodermata | Holothuroidea | Synallactida | Deimatidae

**Deimatidae stet.**

Fig. 68F, G

**Material examined.** S0220: E7335 (**PQ449391**), E7336.

**Localities.** Subduction Plume (3430 m).

**Remarks.** Female (E7335) with internal brooding of four young (E7336). Likely *Orphnurgus* or *Oneirophanta*.

#### ﻿Echinodermata | Holothuroidea | Synallactida | Synallactidae

**Bathyplotescf.moseleyi (Théel, 1886)**

Figs 68H, 69A

**Figure 69.**
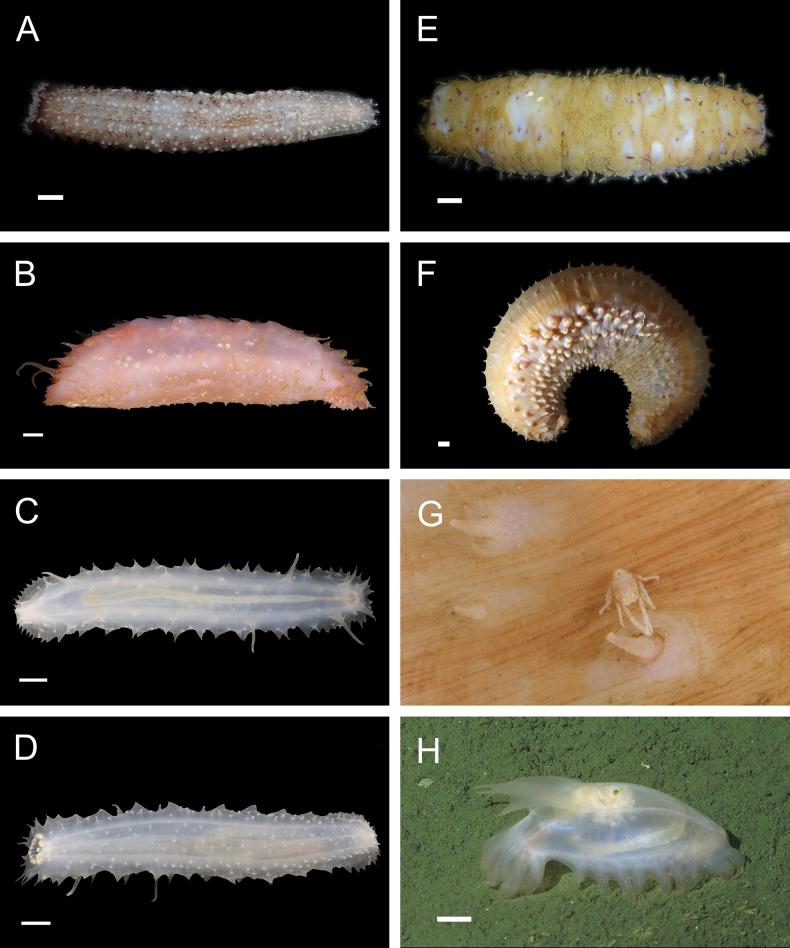
Echinodermata: Holothuroidea, representative live images **A**Bathyplotescf.moseleyi (E7038) **B***Bathyplotes* sp. SIO_BIC_E7063 (E7063) **C***Bathyplotes* sp. SIO_BIC_E7063 (E7314, dorsal view) **D***Bathyplotes* sp. SIO_BIC_E7063 (E7314, ventral view) **E**Synallactescf.chuni (E7049, dorsal view) **F**Synallactescf.chuni (E7313, lateral view) **G**Synallactescf.chuni (E7313, detail with *Munidopsisaspera*) **H***Achlyonice* stet. (E7297, *in situ*). Credit: ROV SuBastian/Schmidt Ocean Institute. Scale bars: 1 cm.

**Reference.** Mongiardino Koch et al. (2023) for phylogenomic analysis.

**Material examined.** AD4913: E7037 (**PQ449365**), E7038 (**PQ450395**); AD4916: E7054 (**PQ449368**), E7055 (**PQ449369**), E7058 (**PQ449370**); S0212: E7302 (**PQ449378**); S0214: E7307 (**PQ449380**), E7308 (OR082751; transcriptome: SRR24876224), E7309 (**PQ449381**).

**Localities.** Jacó Scar (1847–1875 m).

**Remarks.** Color variable from deep magenta to brownish to white. Closely related to Antarctic specimens (e.g., SIO-BIC E6356: KX874357.1, COI ~ 96–98% identical) in the *Bathyplotesmoseleyi* species complex (O’Loughlin et al. 2011). Synallactidae is non-monophyletic and the placement of *Bathyplotes* warrants reconsideration (Miller et al. 2017; Mongiardino Koch et al. 2023).

***Bathyplotes* sp. SIO_BIC_E7063**

Fig. 69B–D

**Material examined.** AD4922: E7063 (**PQ449372**); S0217: E7314 (**PQ449385**).

**Localities.** Mound 12 (1006 m), The Thumb (1065 m).

**Remarks.** Color variable, white or pink.

**Synallactescf.chuni Augustin, 1908**

Fig. 69E–G

**Material examined.** AD4917: E7049 (**PQ449367**); AD4922: E7062 (**PQ449371**); S0217: E7313 (**PQ449384**).

**Localities.** Mound 12 (965–966 m), The Thumb (1065 m).

**Remarks.** E7313 was associated with *Munidopsisaspera* (C13913). These specimens represent the same taxon as voucher specimen E5607, Synallactescf.chuni, from the Guaymas Basin, Gulf of California, 1064 m (Miller et al. 2017). *S.chuni* has been reported from the northwestern Pacific, 75–653 m and possibly to 1000 m (Stepanov and Panina 2016).

#### ﻿Echinodermata | Holothuroidea | Elasipodida | Elpidiidae

***Achlyonice* stet.**

Figs 69H, 70A

**Figure 70.**
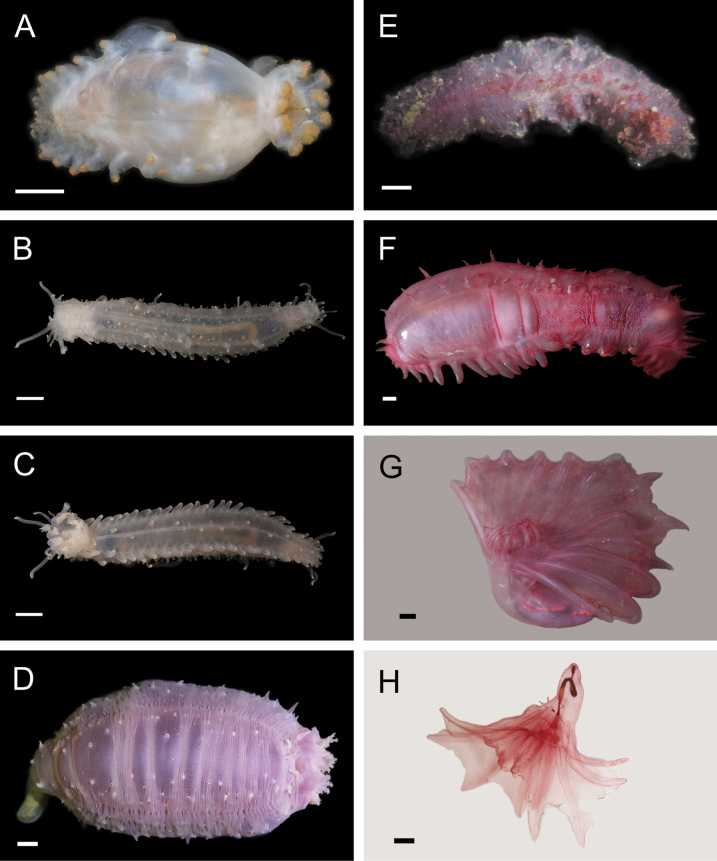
Echinodermata: Holothuroidea, representative live images **A***Achlyonice* stet. (E7042, ventral view) **B***Pannychia* sp. SIO_BIC_E7080 (E7317, dorsal view) **C***Pannychia* sp. SIO_BIC_E7080 (E7317, ventral view) **D***Pannychia* sp. SIO_BIC_E7286 (E7286) **E***Pannychia* stet. (E7070) **F**Laetmogonidae fam. inc. (E7068) **G***Pelagothurianatatrix* (E7311, frontal view) **H***Pelagothurianatatrix* (E7312, lateral view). Scale bars: 1 cm.

**Material examined.** AD4914: E7042, E7043 (**PQ449366**); AD4916: E7053, E7056, E7057; S0212: E7297, E7298, E7299, E7300, E7301; S0214: E7305.

**Localities.** Jacó Scar (~ 1793–1869 m).

**Remarks.** Three specimens (E7299, E7305, E7306) were collected swimming 1–9 m above the seafloor; the other specimens were benthic. The closest COI BLASTN result on GenBank was an unidentified elpidiid from the Indian Ocean (PP778426.1, voucher NMV F296840, 93.53% identity).

#### ﻿Echinodermata | Holothuroidea | Elasipodida | Laetmogonidae

We thank Akito Ogawa (Japan Agency for Marine-Earth Science and Technology) for input on these identifications.

**Laetmogonidae fam. inc.**

Fig. 70B

**Material examined.** AD4923: E7068 (**PQ449373**), E7071 (**PQ449375**).

**Localities.** Parrita Seep (1108 m).

**Distribution.** These specimens morphologically resemble *Laetmogonescotoeides* (H.L. Clark, 1913), which was described from Ballenas Bay, Baja California, 1180 m (Clark 1913), and reported to 1420 m off Guerrero, Mexico (Granja-Fernández et al. 2015b), but further comparison to the type material is required (Akito Ogawa, pers. comm. 10 August 2022). The COI sequences did not closely match any available GenBank reference sequences (<84% identity), and phylogenetic interpretation is challenging, as Laetmogonidae is paraphyletic (Miller et al. 2017; Mongiardino Koch et al. 2023).

***Pannychia* sp. SIO_BIC_E7080**

Fig. 70C, D

**Material examined.** AD4923: E7080 (**PQ450401**); S0218: E7317 (**PQ449386**).

**Localities.** Parrita Seep (~ 1037–1108 m), Parrita Scar (1364 m).

**Remarks.** A potentially undescribed species, within the *Pannychiamoseleyi* Théel, 1882 complex, which requires extensive revision (Ogawa et al. 2022, 2023).

***Pannychia* sp. SIO_BIC_E7286**

Fig. 70E

**Material examined.** AD4990: E7286 (**PQ449377**).

**Localities.** Parrita Seep (1408 m).

**Remarks.** A potentially undescribed species, within the *Pannychiamoseleyi* complex, which requires extensive revision (Ogawa et al. 2022, 2023).

***Pannychia* stet.**

Fig. 70F

**Material examined.** AD4923: E7070 (**PQ449374**), E7079 (**PQ449376**).

**Localities.** Parrita Seep (1091–1098 m).

**Remarks.** A potentially undescribed species, near the *Pannychiamoseleyi* complex.

#### ﻿Echinodermata | Holothuroidea | Elasipodida | Pelagothuriidae

***Pelagothurianatatrix* Ludwig, 1893**

Fig. 70G, H

**Material examined.** S0215: E7311 (**PQ449382**); S0217: E7312 (**PQ449383**).

**Localities.** Mound 12 (982 m depth, 1 m above the seafloor), The Thumb (1056 m depth, 11 m above the seafloor).

**Distribution.** Originally described from several stations in the eastern Pacific from the Gulf of Panama to the Galápagos Islands, 605–3350 m (Ludwig 1893, 1894) and previously reported from Isla del Coco (Cortés 2012). Globally, *Pelagothuria* has been reported from the tropical regions of all ocean basins, at depths of 197–4441 m, but collection of specimens is important to ascertain whether these records represent a single species (Selig et al. 2019).

#### ﻿Echinodermata | Holothuroidea | Elasipodida | Psychropotidae

**Benthodytescf.sanguinolenta Théel, 1882**

Fig. 71A

**Figure 71.**
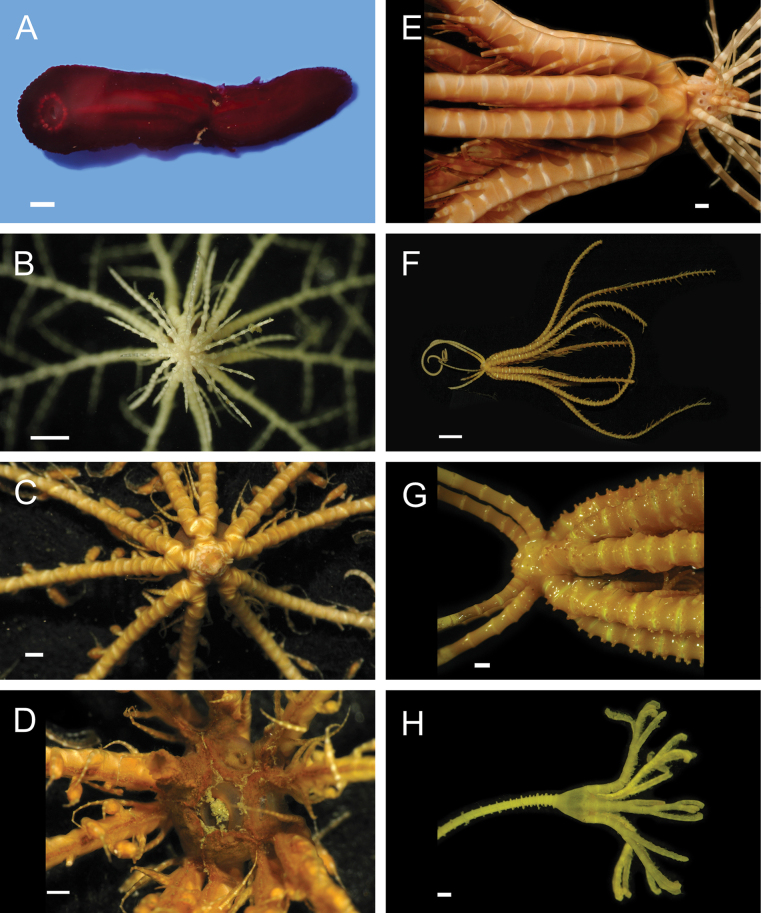
Echinodermata: Holothuroidea and Crinoidea, representative live images **A**Benthodytescf.sanguinolenta (E7334) **B***Fariometra* sp. SIO_BIC_E4389 (E4389) **C***Fariometra* sp. SIO_BIC_E4399 (E4399, aboral view) **D***Fariometra* sp. SIO_BIC_E4399 (E4399, oral view) **E**Psathyrometracf.fragilis (E7034) **F***Thalassometraagassizii* (E4391, lateral view) **G***Thalassometraagassizii* (E4391, detail) **H***Calamocrinusdiomedae* (E4394). Scale bars: 1 cm (**A, F**); 1 mm (**B–E, G, H**).

**Material examined.** S0220: E7334 (**PQ449390**).

**Localities.** Subduction Plume (3453 m depth, 2 m above the seafloor).

**Remarks.** Collected while swimming. The closest COI BLASTN results on GenBank were Benthodytescf.sanguinolenta from the Indian Ocean (PP778424.1, voucher NMV F308225, 98.91% identity) and an Antarctic specimen of *B.sanguinolenta* (HM196505.1, 97.85% identity), which is a species complex (O’Loughlin et al. 2011).

#### ﻿Echinodermata | Crinoidea

We thank and commemorate Charles Messing (Nova Southeastern University) for assistance with these identifications.

#### ﻿Echinodermata | Crinoidea | Comatulida | Antedonoidea | Antedonidae

***Fariometra* sp. SIO_BIC_E4389**

Fig. 71B

**Material examined.** AD4506: E4389 (**PQ449360**).

**Localities.** Parrita Seep (~ 1030–1179 m).

**Remarks.**Antedonidae is not monophyletic and requires major revision (Hemery et al. 2013). This specimen warrants comparison to *Fariometraparvula* (Hartlaub, 1895) which has been recorded from the tropical eastern Pacific including Costa Rica (Alvarado et al. 2022).

***Fariometra* sp. SIO_BIC_E4399**

Fig. 71C, D

**Reference.**Summers et al. (2014a) for DNA sequences (KM014345, reported as *Antedon* sp.) and phylogenetic analysis of specimen E4399.

**Material examined.** AD4509: E4399.

**Localities.** Jacó Scar (1789 m).

**Remarks.** Host of *Pulvinomyzostomuminaki* (A1408 and A1579) (Summers et al. 2014b). This specimen represents a different morphospecies than E4389, with COI 96.15% identical, also warranting comparison to *Fariometraparvula*.

#### ﻿Echinodermata | Crinoidea | Comatulida | Antedonoidea | Zenometridae

**Psathyrometracf.fragilis (AH Clark, 1907)**

Fig. 71E

**Material examined.** AD4913: E7034 (**PQ449364**).

**Localities.** Jacó Scar (1878 m).

**Distribution.***Psathyrometrafragilis* has been reported along the Pacific Rim from Japan (type locality, 914 m) (Clark 1907) through the Aleutian Islands and California to the Gulf of Panama, 439–2903 m (Messing and White 2001).

**Remarks.** Host of *Myzostomajosefinae* (A8362) and *Pulvinomyzostomum* sp. SIO_BIC_A8361.

#### ﻿Echinodermata | Crinoidea | Comatulida | Tropiometroidea | Thalassometridae

***Thalassometraagassizii* (Hartlaub, 1895)**

Fig. 71F, G

**Material examined.** AD4506: E4391 (**PQ450400**).

**Localities.** Parrita Seep (~ 1030–1179 m).

**Distribution.** Originally described from the Galápagos Islands, 598–1250 m, and Panama, 1429 m (Clark 1915) and previously reported from Isla del Coco (Wehrtmann and Cortés 2009b).

#### ﻿Echinodermata | Crinoidea | Hyocrinida | Hyocrinidae

***Calamocrinusdiomedae* Agassiz, 1890**

Fig. 71H

**Material examined.** AD4507: E4394 (**PQ450393**).

**Localities.** Parrita Scar (~ 1659–1667 m).

**Distribution.** Originally described from 717 m off the Galápagos Islands (Agassiz 1890) and known from Pacific Panama and Isla del Coco over a total depth range of 525–1430 m (Roux 2004).

**New records.** The CRM specimen represents a new depth record for this species (1659 m as the most conservative value).

### ﻿﻿Hemichordata

***Saccoglossus* sp. SIO_BIC_H35**

Fig. 72A

**Figure 72.**
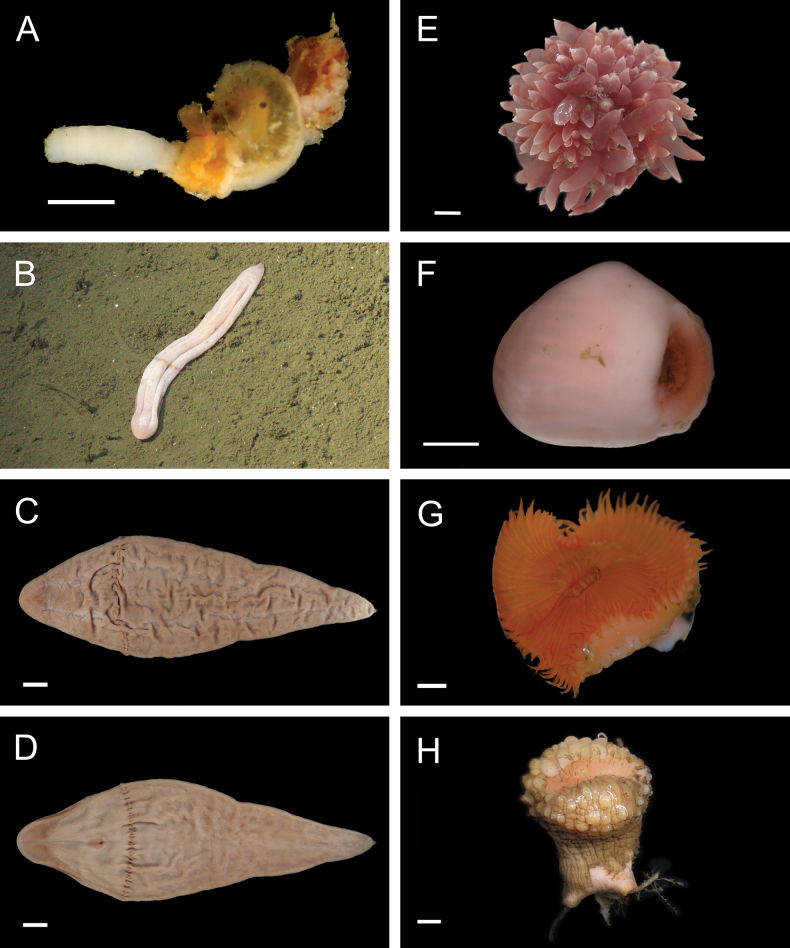
Hemichordata, Xenacoelomorpha, and Cnidaria: Actiniaria, representative live images **A***Saccoglossus* sp. SIO_BIC_H35 (H35) **B***Xenoturbella* sp. SIO_BIC_BI1373 (BI1364, *in situ*). Credit: ROV SuBastian/Schmidt Ocean Institute **C***Xenoturbella* sp. SIO_BIC_BI1373 (BI1373, dorsal view) **D***Xenoturbella* sp. SIO_BIC_BI1373 (BI1373, ventral view) **E***Liponema* stet. (Co2922) **F***Paracalliactis* stet. (Co2300) **G**Hormathiidae stet. (Co2268) **H**Hormathiidae stet. (Co2875). Scale bars: 1 mm (**A**); 1 cm (**C–H**).

**Material examined.** AD4906: H35 (16S: **PQ304659**).

**Localities.** Mound 12 (1000 m).

**Remarks.** The closest 16S BLASTN result on GenBank was a specimen of *Saccoglossus* sp. from Friday Harbor, Washington, 5–10 m (KF683544.1, 96.40% identity). Based on the reported 16S interspecific distances within the monophyletic genus *Saccoglossus* (0.1–17.9%, corrected) and distances between *Saccoglossus* and other harrimaniid genera (>18.2%, corrected) (Cannon et al. 2013), we identify the CRM specimen as within *Saccoglossus* and likely an undescribed species.

### ﻿﻿Xenacoelomorpha

***Xenoturbella* sp. SIO_BIC_BI1373**

Fig. 72B–D

**Material examined.** S0215: BI1364; S0230: BI1372, BI1373, BI1374.

**Localities.** Mound 12 (999 m), Mound Jaguar (1895 m).

**Remarks.** An undescribed species.

### ﻿﻿Cnidaria

#### ﻿Cnidaria | Anthozoa

We list entries following the phylogenetic relationships in McFadden et al. (2021).

#### ﻿Cnidaria | Anthozoa | Hexacorallia | Actiniaria | Actinioidea | Liponematidae

***Liponema* stet.**

Fig. 72E

**Material examined.** AD4913: Co2922.

**Localities.** Jacó Scar (1871 m).

#### ﻿Cnidaria | Anthozoa | Hexacorallia | Actiniaria | Metridioidea | Hormathiidae

**Hormathiidae stet.**

Fig. 72F, G

**Material examined.** AD4504: Co2268; AD4513: Co2280 (16S: **PQ304656**; COIII: **PQ435166**); AD4907: Co2918; AD4922: Co2875, Co2933; AD4923: Co2877.

**Localities.** Mound 11 (~ 1004–1011 m), Mound 12 (994–1002 m), Parrita Seep (1101 m), Jacó Scar (~ 1799 m).

**Remarks.** Multiple species may be represented. Co2268 was attached to the tube of *Lamellibrachiadonwalshi*. Co2875 and Co2933 were associated with squat lobsters, *Munidopsishendersoniana* (C12803 and C12799, respectively). For Co2280 the closest BLASTN results on GenBank were: for 16S, 99.78% identity to *Paraphelliactisxishaensis* Feng, Liu, Xu, Zhou, Zhu, Liu, Wu, Li, Qiu, He, Wang, Zhang & Wang, 2021 (MT997141.1), *Paracalliactis* sp. (FJ489429.1), and *Paraphelliactis* sp. (FJ489431.1); for COIII, 100.00% identity to unidentified Hormathiidae from central California at 1287 m (MN954949.1) and *Chondrophellia* sp. (FJ489489.1).

***Paracalliactis* stet.**

Fig. 72H

**Material examined.** AD4590: Co2300; AD4591: Co2320.

**Localities.** Jacó Scar (~ 1800 m).

**Remarks.** Associated with *Parapagurusforaminosus* hermit crabs, as is characteristic of *Paracalliactis* (Gusmão and Daly 2010; Gusmão et al. 2020) (Co2300 with crab C11210, Co2320 with crab C13308).

#### ﻿Cnidaria | Anthozoa | Hexacorallia | Actiniaria | Metridioidea | Kadosactinidae

**Kadosactinidae cf. sp. B sec. Goffredi et al. 2021**

Fig. 73A, B

**Figure 73.**
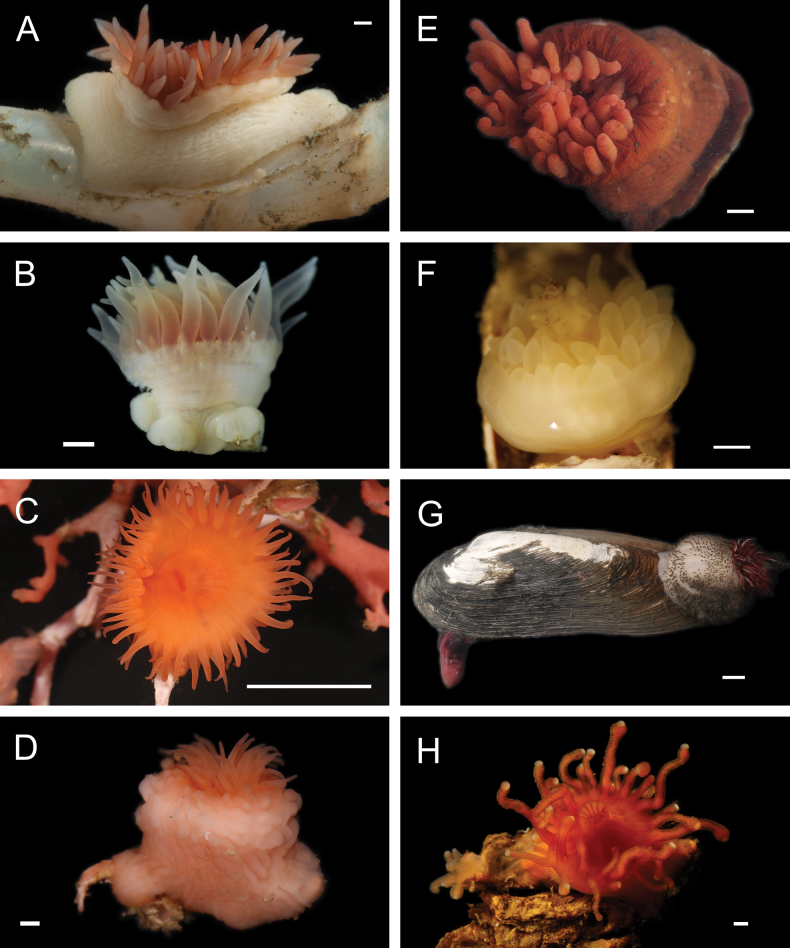
Cnidaria: Actiniaria, representative live images **A**Kadosactinidae cf. sp. B sec. Goffredi et al. 2021 (Co2309, oral view) **B**Kadosactinidae cf. sp. B sec. Goffredi et al. 2021 (Co2265, lateral view) **C**Amphianthidae fam. inc. (Co2874, oral view) **D**Amphianthidae fam. inc. (Co2874, lateral view) **E**Actiniaria sp. SIO_BIC_Co2876 (Co2876) **F**Actiniaria sp. SIO_BIC_Co3056 (Co3056) **G**Actiniaria sp. SIO_BIC_Co3084 (Co3084) **H**Actiniaria sp. SIO_BIC_Co3086 (Co3086). Scale bars: 1 mm (**A, B, D, F, H**); 1 cm (**C, E, G**).

**Material examined.** AD4501: Co2265 (16S: **PQ304653**; COIII: **PQ299038**); AD4513: Co2279 (16S: **PQ304655**); AD4590: Co2309 (**PQ449359**; 16S: **PQ304657**).

**Localities.** Mound 12 (~ 984–997 m), Jacó Scar (~ 1744–1818 m).

**Remarks.** Co2309 was associated with a vestimentiferan tubeworm (*Escarpiaspicata* or *Lamellibrachiabarhami*). The COIII sequence of Co2265 was nearly identical to those of an undescribed vent anemone, Kadosactinidae sp. B, from the Pescadero Basin, Gulf of California, ﻿3692 m (MW148236.1, 99.46% identity) (Goffredi et al. 2021), and to *Alvinactischessi* Zelnio, Rodríguez & Daly, 2009 (Kadosactinidae) (GU473352.1, 99.17% identity). Due to the relatively short sequence lengths and close relationships among taxa, the CRM16S sequences were 100.0% identical to those of Kadosactinidae sp. B (MW172213.1), *A.chessi* (GU473296.1), and *Cyanantheahourdezi* Zelnio, Rodríguez & Daly, 2009 (Kadosactinidae) (GU473293.1). The COI sequence of Co2309 was 99.52–99.68% identical to those of Actinostolidae spp. from vents in the Indian Ocean (MH202753.1, OK267405.1, OK267413.1) and *Maractis* sp. from vents at the Mid-Cayman Spreading Center (KJ566948.1), although no COI sequences from any Kadosactinidae were available for comparison.

#### ﻿Cnidaria | Anthozoa | Hexacorallia | Actiniaria | Metridioidea

**Amphianthidae fam. inc.**

Fig. 73C, D

**Material examined.** AD4506: Co2273 (16S: **PQ304654**; COIII: **PQ299039**); AD4923: Co2874.

**Localities.** Parrita Seep (~ 1030–1094 m).

**Remarks.** Associated with coralliids (Co2273 with coralliid Co2271; Co2874 with coralliid Co2947). For Co2273 the closest BLASTN results on GenBank were: for 16S, *Peronanthus* sp. (KJ482956.1, 99.56% identity), *Galatheanthemum* sp. from the Mariana Trench (OL912950.1, 99.33% identity), and an unidentified sea anemone (U40291.1, 99.33% identity); for COIII, 95.94% identity to *Peronanthus* sp. (KJ482976.1) and *Galatheanthemum* spp. (KJ482977.1, OL912950.1, OQ697433.1).

#### ﻿Cnidaria | Anthozoa | Hexacorallia | Actiniaria

**Actiniaria sp. SIO_BIC_Co2876**

Fig. 73E

**Material examined.** AD4923: Co2876.

**Localities.** Parrita Seep (1041 m).

**Actiniaria sp. SIO_BIC_Co3056**

Fig. 73F

**Material examined.** AD4988: Co3056.

**Localities.** Mound 11 (1010 m).

**Remarks.** Associated with a naturally occurring wood fall.

**Actiniaria sp. SIO_BIC_Co3084**

Fig. 73G

**Material examined.** S0220: Co3084.

**Localities.** Subduction Plume (3408 m).

**Remarks.** Associated with a clam, *Calyptogenadiagonalis* (M17065).

**Actiniaria sp. SIO_BIC_Co3086**

Fig. 73H

**Material examined.** S0219: Co3086.

**Localities.** Rio Bongo Scar (661 m).

**Remarks.** Associated with a naturally occurring wood fall.

#### ﻿Cnidaria | Anthozoa | Hexacorallia | Antipatharia | Schizopathidae

***Bathypathes* stet.**

Fig. 74A, B

**Figure 74.**
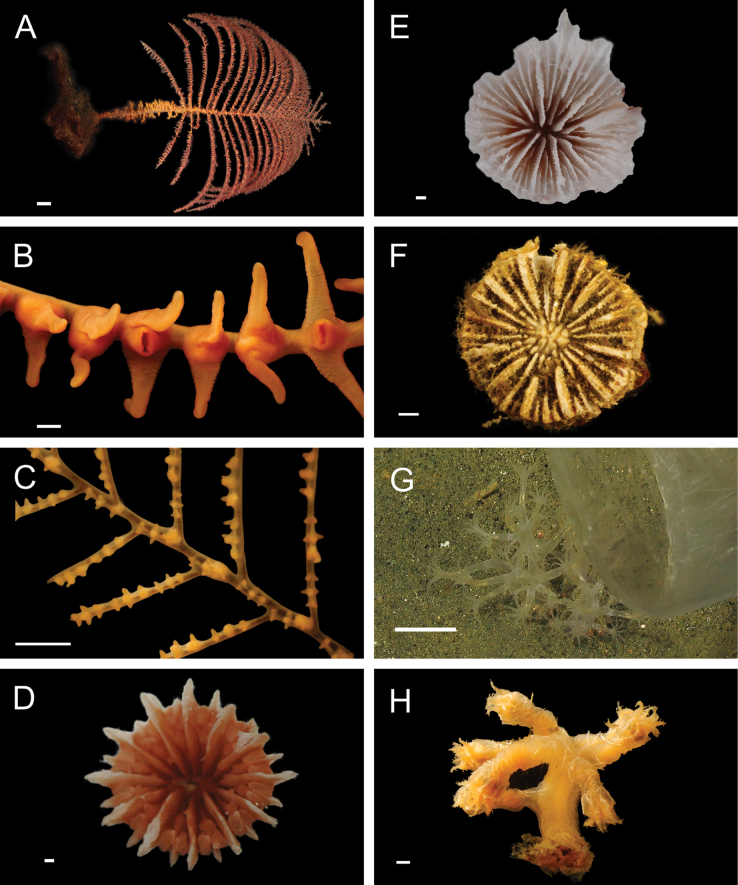
Cnidaria: Antipatharia, Scleractinia, and Octocorallia, representative live images **A***Bathypathes* stet. (MZUCR 3541, colony) **B***Bathypathes* stet. (MZUCR 3541, polyps) **C***Lillipathesritamariae* (Co2944) **D***Javaniacailleti* (Co2276) **E***Polymyceswellsi* (Co2951) **F***Astrangia* gen. inc. (Co2943) **G***Aquaumbraklapferi* (MZUCR 3072, *in situ*). Credit: ROV SuBastian/Schmidt Ocean Institute **H***Aquaumbraklapferi* (MZUCR 3072, detail). Scale bars: 1 cm (**A, G**); 1 mm (**B–F, H**).

**Material examined.** AD4914: MZUCR 3537; AD4922: MZUCR 3541; S0218: MZUCR 3545.

**Localities.** Mound 12 (966 m), Parrita Scar (1154 m), Jacó Scar (1632 m).

***Lillipathesritamariae* Opresko & Breedy, 2010**

Fig. 74C

**Reference.** Opresko and Breedy, 2010** (no DNA sequences available).

**Localities.** Mound 12 (~ 967–995 m; type locality).

**Distribution.** Recorded in the eastern Pacific from the CRM and northern Chile, and in the southwestern Pacific off New Zealand and Antarctica, 1005–1746 m (Araya et al. 2018).

**Remarks.** Host of the myzostome *Eenymeenymyzostoma* sp. SIO_BIC_A8428.

#### ﻿Cnidaria | Anthozoa | Hexacorallia | Scleractinia | Flabellidae

***Javaniacailleti* (Duchassaing & Michelotti, 1864)**

Fig. 74D

**Material examined.** AD4510: Co2276.

**Localities.** Jacó Summit (~ 741–744 m).

**Distribution.** Originally described from the Caribbean (Duchassaing and Michelotti 1864) and considered cosmopolitan (Cairns 1991). Previously reported from Isla del Coco and the Galápagos Islands, 245–576 m (Cairns 1991; Cortés 2012).

***Polymyceswellsi* Cairns, 1991**

Fig. 74E

**Material examined.** AD4923: Co2951 (fragment).

**Localities.** Parrita Seep (~ 1037–1108 m).

**Distribution.** Originally described from the Galápagos Islands, 391–813 m (Cairns 1991), and thought to be cosmopolitan at depths of 355–1682 m (Cairns 2006).

**Remarks.** A new record for Costa Rica.

#### ﻿Cnidaria | Anthozoa | Hexacorallia | Scleractinia | Astrangiidae

***Astrangia* gen. inc.**

Fig. 74F

**Material examined.** AD4921: Co2943 (fragment).

**Localities.** Quepos Slide (~ 345–394 m).

**Remarks.** Damaged specimens, most likely *Astrangia*.

#### ﻿Cnidaria | Anthozoa | Octocorallia

We list entries following the phylogenetic relationships of Malacalcyonacea, Scleralcyonacea, and Pennatuloidea as defined in McFadden et al. (2022), and then alphabetically within those clades.

#### ﻿Cnidaria | Anthozoa | Octocorallia | Malacalcyonacea | Aquaumbridae

***Aquaumbraklapferi* Breedy, van Ofwegen & Vargas, 2012**

Fig. 74G, H

**Reference.**Breedy et al. 2012**.

**Additional material examined.** S0219: MZUCR 3072 (voucher), Co3087 (tissue).

**Localities.** Rio Bongo Scar (610 m; this study).

**Distribution.** Originally described from the insular shelf of Isla del Coco, 268–308 m (Breedy et al. 2012) and subsequently reported from the West Florida Slope, Gulf of Mexico, 421 m (supplementary table 2 in Quattrini et al. (2020); Sequence Read Archive: SRS5647140, BioSample: SRS5647140). The Gulf of Mexico record suggests that the relatively shallow eastern Pacific and western Atlantic populations were separated by the rise of the Isthmus of Panama relatively recently (see O’Dea et al. (2016)).

**New records.** The CRM specimen represents a new depth record for this species and establishes Rio Bongo Scar, 465 km northeast of Isla del Coco, as a second known locality in Costa Rica.

#### ﻿Cnidaria | Anthozoa | Octocorallia | Malacalcyonacea | Clavulariidae

**Clavulariidae sp. MZUCR_3551**

Fig. 75A, B

**Figure 75.**
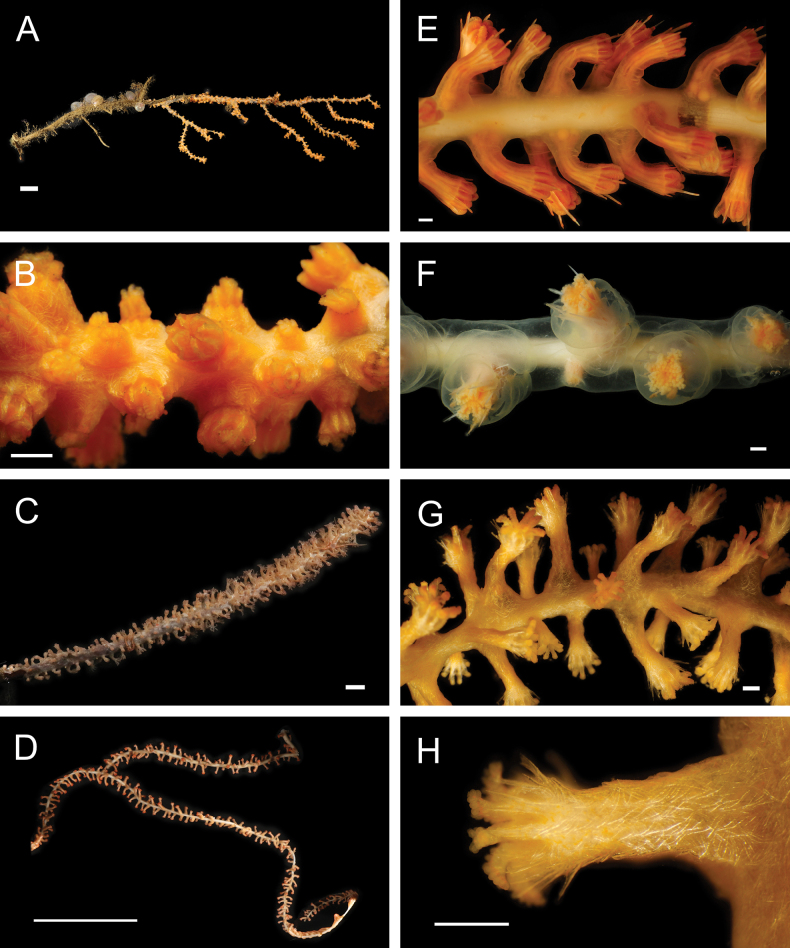
Cnidaria: Octocorallia, representative live images **A**Clavulariidae sp. MZUCR_3551 (MZUCR 3551, colony) **B**Clavulariidae sp. MZUCR_3551 (MZUCR 3551, polyps) **C**Clavulariidae sp. MZUCR_3552 (MZUCR 3552) **D**Isididae sp. MZUCR_3129 (MZUCR 3129, colony) **E**Isididae sp. MZUCR_3129 (Co2953, polyps) **F**Isididae sp. MZUCR_3547 (MZUCR 3547) **G***Acanthogorgia* sp. MZUCR_3113 (MZUCR 3113, colony) **H***Acanthogorgia* sp. MZUCR_3113 (MZUCR 3113, polyps). Scale bars: 1 cm (**A, C**); 1 mm (**B, E–H**); 10 cm (**D**).

**Material examined.** AD4918: MZUCR 3551 (voucher), Co2929 (tissue).

**Localities.** Quepos Slide (338 m).

**Remarks.** The base of the colony was encrusted with hydroids and scallops, *Delectopectenvancouverensis* (M16184).

**Clavulariidae sp. MZUCR_3552**

Fig. 75C

**Material examined.** AD4923: MZUCR 3552 (voucher), Co2940 (tissue).

**Localities.** Parrita Seep (1075 m).

#### ﻿Cnidaria | Anthozoa | Octocorallia | Malacalcyonacea | Isididae

**Isididae sp. MZUCR_3129**

Fig. 75D, E

**Material examined.** AD4923: MZUCR 3129 (voucher), tissue samples Co2952, Co2954; Co2953.

**Localities.** Parrita Seep (1037 m).

**Remarks.** Host of *Heteroptychusgalapagos* (C12816, C12817).

**Isididae sp. MZUCR_3547**

Fig. 75F

**Material examined.** AD4914: MZUCR 3547 (voucher), Co2927 (tissue).

**Localities.** Jacó Scar (1632 m).

#### ﻿Cnidaria | Anthozoa | Octocorallia | Malacalcyonacea | Paramuriceidae

***Acanthogorgia* sp. MZUCR_3113**

Fig. 75G, H

**Material examined.** AD4924: MZUCR 3113 (voucher), Co2956 (tissue).

**Localities.** Parrita Seep (1403 m).

**Remarks.** One colony was attached to a tube of *Lamellibrachiabarhami*.

***Acanthogorgia* sp. MZUCR_3549**

Fig. 76A, B

**Figure 76.**
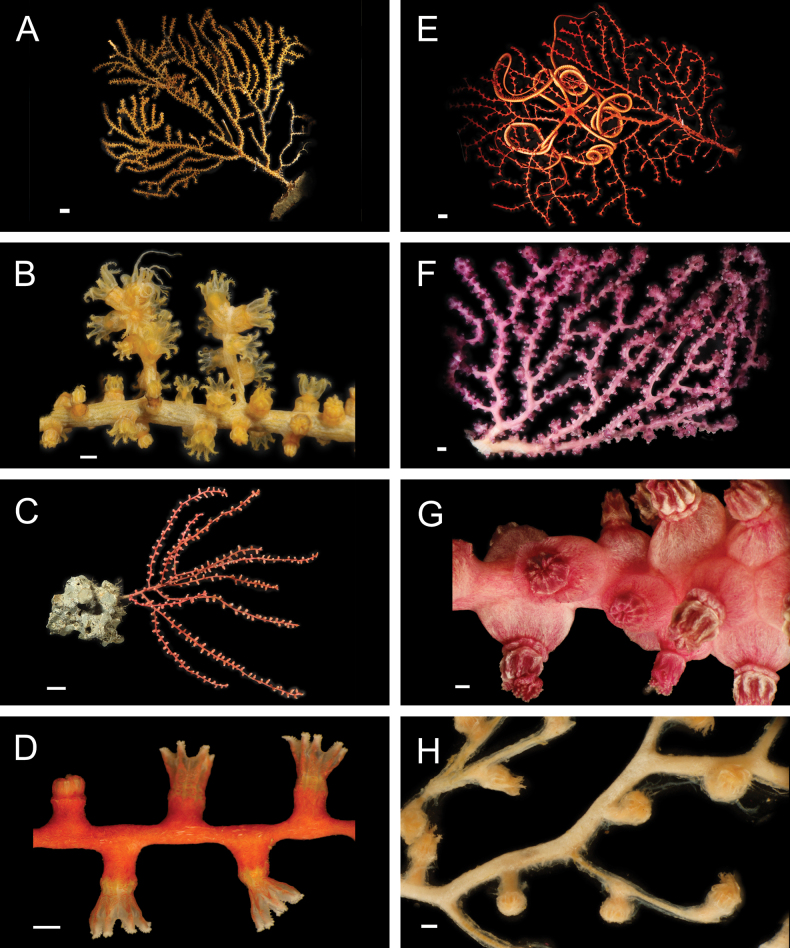
Cnidaria: Octocorallia, representative live images **A***Acanthogorgia* sp. MZUCR_3549 (MZUCR 3549, colony) **B***Acanthogorgia* sp. MZUCR_3549 (MZUCR 3549, polyps) **C***Swiftiasahlingi* (MZUCR 2648, colony) **D***Swiftiasahlingi* (Co2945, polyps) **E***Swiftia* stet. (Co3080) **F***Victorgorgia* stet. (MZUCR 3114, colony) **G***Victorgorgia* stet. (MZUCR 3114, polyps) **H***Chrysogorgia* sp. MZUCR_3057 (MZUCR 3057). Scale bars: 1 cm (**A, C, E, F**); 1 mm (**B, D, G, H**).

**Material examined.** S0216: MZUCR 3549 (voucher), Co3079 (tissue).

**Localities.** Quepos Slide (316 m).

#### ﻿Cnidaria | Anthozoa | Octocorallia | Malacalcyonacea | Plexauridae

***Swiftiasahlingi* Breedy et al., 2019**

Fig. 76C, D

**Reference.**Breedy et al. 2019**.

**Localities.** Mound 12 (996–1002 m; type locality).

**Distribution.** Known only from the CRM seeps.

**Remarks.** Found in dense clusters on authigenic carbonates, often near sites of active methane seepage (Breedy et al. 2019).

***Swiftia* stet.**

Fig. 76E

**Material examined.** S0218: Co3080 (tissue).

**Localities.** Parrita Scar (1431 m).

**Remarks.** Associated with an ophiuroid, *Ophiocreascarnosus* (E7318). Possibly an undescribed species of *Swiftia*, distinct from *S.sahlingi.* Further taxonomic work on eastern Pacific *Swiftia* species is needed, including detailed comparison of this specimen to those from seamounts west of the CRM seeps at 2089–2270 m depth (Breedy et al. 2019).

#### ﻿Cnidaria | Anthozoa | Octocorallia | Malacalcyonacea | Victorgorgiidae

***Victorgorgia* stet.**

Fig. 76F, G

**Material examined.** AD4923: MZUCR 3114 (voucher), Co2939 (tissue).

**Localities.** Parrita Seep (1074 m).

#### ﻿Cnidaria | Anthozoa | Octocorallia | Scleralcyonacea | Chrysogorgiidae

***Chrysogorgia* sp. MZUCR_3057**

Fig. 76H

**Material examined.** AD4923: MZUCR 3057 (voucher), Co2948 (tissue).

**Localities.** Parrita Seep (1041 m).

***Chrysogorgia* sp. MZUCR_3063**

Fig. 77A

**Figure 77.**
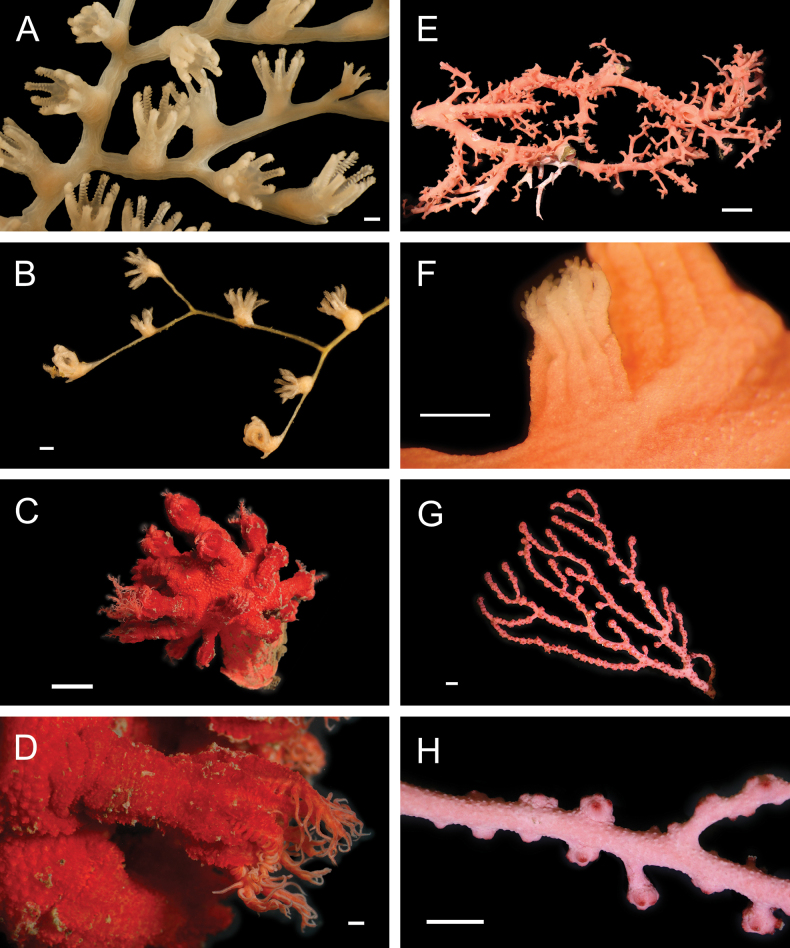
Cnidaria: Octocorallia, representative live images **A***Chrysogorgia* sp. MZUCR_3063 (MZUCR 3063) **B***Chrysogorgia* sp. MZUCR_3142 (MZUCR 3142) **C***Anthomastus* stet. (Co2919, colony) **D***Anthomastus* stet. (Co2919, polyps) **E**Coralliidae stet. (MZUCR 3130, colony) **F**Coralliidae stet. (MZUCR 3130, polyps) **G***Paragorgia* stet. (Co3054, colony) **H***Paragorgia* stet. (Co3054, detail). Scale bars: 1 mm (**A, B, D, F**); 1 cm (**C, E, G, H**).

**Material examined.** AD4505: Co2270; AD4923: MZUCR 3063 (voucher), Co2950 (tissue).

**Localities.** Mound 11 (~ 1019–1025 m), Parrita Seep (1091 m).

***Chrysogorgia* sp. MZUCR_3142**

Fig. 77B

**Material examined.** AD4923: MZUCR 3142 (voucher), Co2949 (tissue).

**Localities.** Parrita Seep (~ 1037–1108 m).

#### ﻿Cnidaria | Anthozoa | Octocorallia | Scleralcyonacea | Coralliidae

***Anthomastus* stet.**

Fig. 77C, D

**Material examined.** AD4913: Co2919; MZUCR 3126 (voucher), Co2923 (tissue).

**Localities.** Jacó Scar (~ 1849–1867 m).

**Coralliidae stet.**

Fig. 77E, F

**Material examined.** AD4506: MZUCR 3550 (voucher), Co2271 (tissue); AD4923: MZUCR 3130 (voucher), Co2947 (tissue).

**Localities.** Parrita Seep (~ 1030–1094 m).

**Remarks.** Host of scaleworms, Gorgoniapolynoecf.caeciliae (A1549 on Co2271, A8455 on Co2947); a pyrgomatid barnacle (C12815 on Co2947); ophiuroids (*Ophiacanthamoniliformis*, E4390 on Co2271; Ophiuroglyphacf.meridionalis, E7978 on Co2271); and anemones (Co2273 on Co2271, Co2874 on Co2947).

***Paragorgia* stet.**

Fig. 77G, H

**Material examined.** AD4990: Co3054 (tissue).

**Localities.** Parrita Seep (1407 m).

**Remarks.** Host of the nemertean *Alvinonemertesdariae* (Sagorny et al. 2022).

#### ﻿Cnidaria | Anthozoa | Octocorallia | Scleralcyonacea | Primnoidae

***Callogorgia* stet.**

Fig. 78A

**Figure 78.**
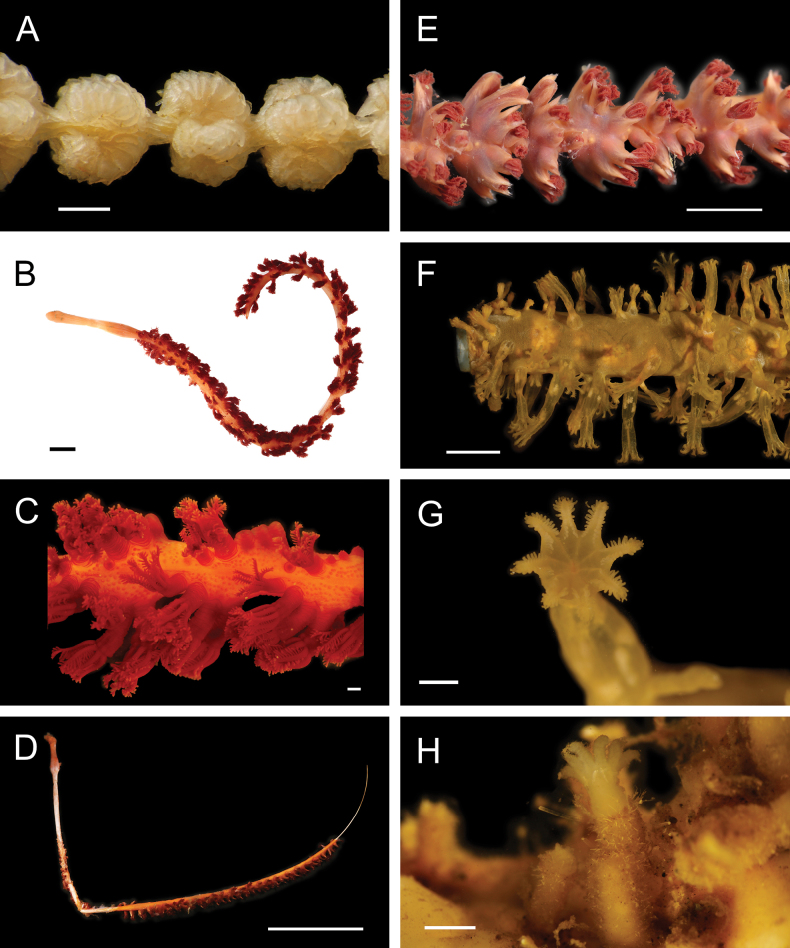
Cnidaria: Octocorallia, representative live images **A***Callogorgia* stet. (MZUCR 3548) **B***Anthoptilumgrandiflorum* sp. inc. (MZUCR 3554, colony) **C***Anthoptilumgrandiflorum* sp. inc. (MZUCR 3554, polyps) **D***Balticina* gen. inc. (MZUCR 3124, colony) **E***Balticina* gen. inc. (MZUCR 3124, polyps) **F**Octocorallia sp. MZUCR_3112 (MZUCR 3112, colony) **G**Octocorallia sp. MZUCR_3112 (MZUCR 3112, polyps) **H**Octocorallia sp. MZUCR_3553 (MZUCR 3553). Scale bars: 1 mm (**A, C, G, H**); 1 cm (**B, E, F**); 10 cm (**D**).

**Material examined.** AD4914: MZUCR 3548 (voucher), Co2926 (tissue); AD4916: MZUCR 3539 (voucher), Co2931 (tissue); AD4923: MZUCR 3140 (voucher), Co2941 (tissue).

**Localities.** Parrita Seep (1052 m), Jacó Scar (~ 1604–1645 m).

**Remarks.** Co2941 was associated with an ophiuroid, *Ophiocreascarnosus* (E7072).

#### ﻿Cnidaria | Anthozoa | Octocorallia | Scleralcyonacea | Pennatuloidea

***Anthoptilumgrandiflorum*** (**Verrill, 1879) sp. inc.**

Fig. 78B, C

**Material examined.** AD4922: MZUCR 3554 (voucher), Co2937 (tissue).

**Localities.** Mound 12 (1005 m).

**Distribution.** Originally described from the north Atlantic off Nova Scotia and New England (Goode 1884), *A.grandiflorum* is considered cosmopolitan (Williams 2011).

**Remarks.** Associated with two squat lobsters, *Munidopsishendersoniana* (C12809). Further morphological examination is needed to confirm the identification.

***Balticina* gen. inc.**

Fig. 78D, E

**Material examined.** AD4506: MZUCR 3538 (voucher), Co2274 (tissue); AD4923: MZUCR 3124.

**Localities.** Parrita Seep (1030–1098 m).

#### ﻿Cnidaria | Anthozoa | Octocorallia

**Octocorallia sp. MZUCR_3112**

Fig. 78F, G

**Material examined.** AD4917: MZUCR 3112 (voucher), Co2934 (tissue).

**Localities.** Mound 12 (965 m).

**Remarks.** Stoloniferan morphology.

**Octocorallia sp. MZUCR_3553**

Fig. 78H

**Material examined.** S0217: MZUCR 3553 (voucher), Co3083 (tissue).

**Localities.** The Thumb (~ 940–1070 m).

**Remarks.** Attached to a serpulid tube. Stoloniferan morphology.

**Octocorallia sp. SIO_BIC_Co2278**

Fig. 79A, B

**Figure 79.**
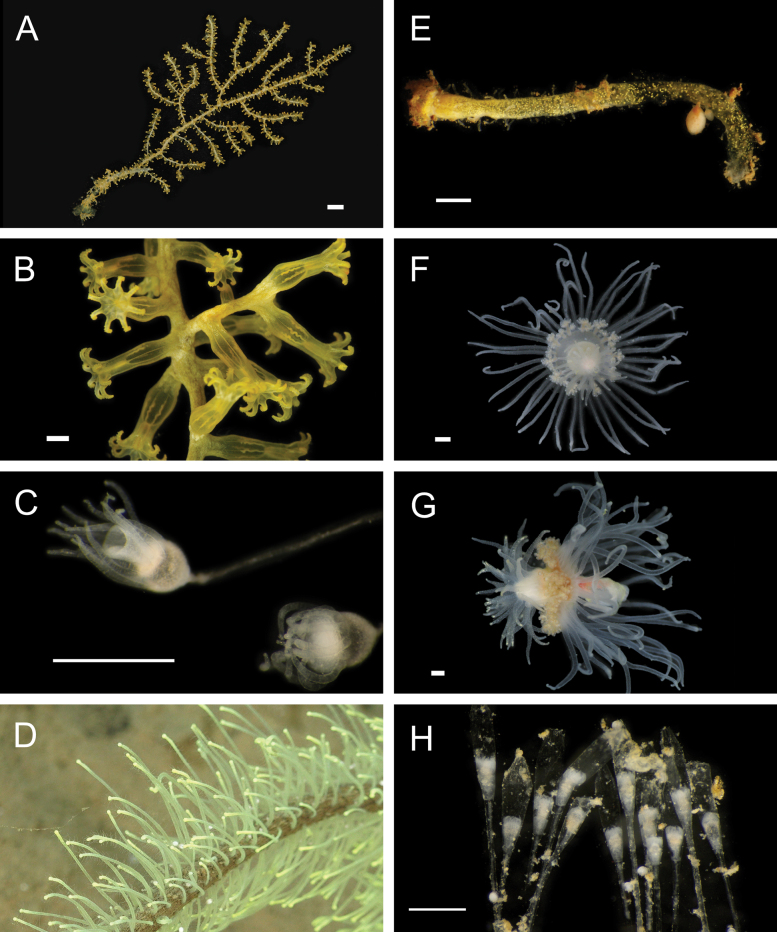
Cnidaria: Octocorallia and Hydrozoa, representative live images **A**Octocorallia sp. SIO_BIC_Co2278 (Co2278, colony) **B**Octocorallia sp. SIO_BIC_Co2278 (Co2278, polyps) **C**Anthoathecata stet. (Co3016) **D***Candelabrum* stet. (Co3088, *in situ* on the tube of Melinnopsiscf.armipotens, A12604). Credit: ROV SuBastian/Schmidt Ocean Institute **E***Candelabrum* stet. (Co3088, polyp) **F***Corymorpha* stet. (Co2272, oral view) **G***Corymorpha* stet. (Co2272, lateral view) **H**Leptothecata sp. SIO_BIC_Co2928 (Co2928). Scale bars: 1 cm (**A**); 1 mm (**B–H**).

**Material examined.** AD4512: Co2278.

**Localities.** Quepos Slide (344 m).

#### ﻿Cnidaria | Hydrozoa | Anthoathecata

**Anthoathecata stet.**

Fig. 79C

**Material examined.** AD4974: Co3016; S0215: Co3082.

**Localities.** Mound 12 (1001–1011 m).

**Remarks.** Co3016 was associated with an experimentally deployed carbonate. Co3082 was associated with the tube of *Lamellibrachiadonwalshi*.

#### ﻿Cnidaria | Hydrozoa | Anthoathecata | Aplanulata | Candelabridae

***Candelabrum* stet.**

Fig. 79D, E

**Material examined.** S0220: Co3088.

**Localities.** Subduction Plume (3503 m).

**Remarks.** Abundant on the exterior of a polychaete tube (Melinnopsiscf.armipotens, A12604), which extended above the seafloor to an estimated length of 20 cm. We thank Dhugal Lindsay (Japan Agency for Marine-Earth Science and Technology) for this identification.

#### ﻿Cnidaria | Hydrozoa | Anthoathecata | Aplanulata | Corymorphidae

***Corymorpha* stet.**

Fig. 79F, G

**Material examined.** AD4506: Co2272.

**Localities.** Parrita Seep (~ 1030–1179 m).

**Remarks.** We thank Dhugal Lindsay for this identification.

#### ﻿Cnidaria | Hydrozoa | Leptothecata

**Leptothecata sp. SIO_BIC_Co2928**

Fig. 79H

**Material examined.** AD4916: Co2928.

**Localities.** Jacó Scar (1854 m).

**Remarks.** Epibionts on the fecampiid egg case Pt64.

#### ﻿Cnidaria | Scyphozoa | Coronamedusae | Coronatae | Nausithoidae

***Nausithoe* stet.**

Fig. 80A

**Figure 80.**
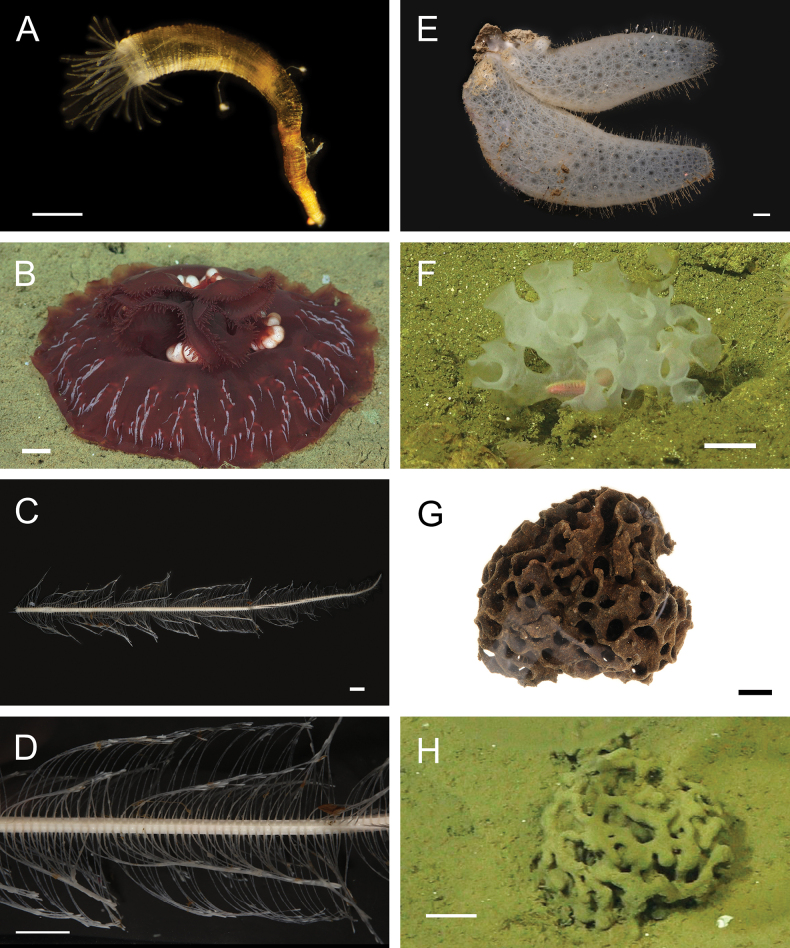
Cnidaria: Scyphozoa, Porifera, and Foraminifera, representative live images **A***Nausithoe* stet. (Co2972, polyp) **B**Ulmaridae stet. (Co3011, medusa *in situ*). Credit: ROV SuBastian/Schmidt Ocean Institute **C***Asbestopluma* gen. inc. (P1754) **D***Asbestopluma* gen. inc. (P1754, detail) **E***Dictyaulus* gen. inc. (P1687) **F***Farreaocca* (P1753, *in situ* with polynoid scaleworms A10096 and A10098 inside). Credit: ROV SuBastian/Schmidt Ocean Institute **G***Reticulammina* stet (BI1172) **H***Shinkaiya* stet. (*in situ*; specimen destroyed). Credit: ROV SuBastian/Schmidt Ocean Institute. Scale bars: 1 mm (**A**); 1 cm (**B–H**).

**Material examined.** AD4918: Co2972.

**Localities.** Quepos Slide (338 m).

**Remarks.** The outer surfaces of the tubes of these polyps show prominent rings with sharp edges and relatively sparse sculpturing, indicative of *Nausithoe* type 1 in Jarms et al. (2002). Differentiation of *Nausithoe* species based on polyp morphology alone is difficult (Molinari et al. 2023). The tube-dwelling polyps of coronate scyphozoans (Atorellidae and Nausithoidae) have been reported from near-surface waters to at least 3,233 m (Song et al. 2021; Molinari et al. 2023). We thank Xikun Song (Institute of Deep-Sea Science and Engineering, Chinese Academy of Sciences) and Dhugal Lindsay for this identification.

#### ﻿Cnidaria | Scyphozoa | Discomedusae | Semaeostomeae

**Ulmaridae stet.**

Fig. 80B

**Material examined.** S0220: Co3011 (**PQ300674**) (tissue; voucher disintegrated during collection).

**Localities.** Subduction Plume (3434 m).

**Remarks.** This specimen was observed alive yet upside down on the seafloor. We thank George Matsumoto (Monterey Bay Aquarium Research Institute) and Rebecca Helm (Georgetown University) for generating the COI sequence. This medusa showed morphological and perhaps behavioral similarities to an undescribed hadal ulmarid that was reported from 8200 m in the New Britain Trench and observed skimming the sediment surface to feed on particulates (Gallo et al. 2015) (Dhugal Lindsay, pers. comm. 25 November 2019).

#### ﻿﻿Porifera

We thank Lonny Lundsten (Monterey Bay Aquarium Research Institute) for the identification of these specimens.

#### ﻿Porifera | Demospongiae | Poecilosclerida | Cladorhizidae

***Asbestopluma* gen. inc.**

Fig. 80C, D

**Material examined.** S0220: P1754 (tissue).

**Localities.** Subduction Plume (3601 m).

**Remarks.** Associated with a scaleworm, *Macellicephala* sp. SIO_BIC_A10099 (A10099).

#### ﻿Porifera | Hexactinellida | Lyssacinosida | Euplectellidae

***Dictyaulus* gen. inc.**

Fig. 80E

**Material examined.** AD4923: P1687.

**Localities.** Parrita Seep (1098 m).

**Remarks.** Host of stenopodids, *Spongicoloidesgalapagensis* (C12735).

#### ﻿Porifera | Hexactinellida | Sceptrulophora | Farreidae

***Farreaocca* Bowerbank, 1862**

Fig. 80F

**Material examined.** S0219: P1752, P1753.

**Localities.** Rio Bongo Scar (602–631 m).

**Distribution.** Considered cosmopolitan at depths of ﻿204–1901 m, with several subspecies occurring in the eastern Pacific between California and Panama, 523–1244 m (Lopes et al. 2011).

**Remarks.** Host of polynoid scaleworms (A10096, A10098).

### ﻿﻿Chromista

#### ﻿Chromista | Foraminifera | Monothalamea | Xenophyophoroidea

***Reticulammina* stet.**

Fig. 80G

**Reference.**Levin and Rouse 2020.

**Additional material examined.** AD4913: BI1172.

**Localities.** Jacó Scar (1866–1885 m).

**Remarks.** One specimen (destroyed) was associated with snailfish eggs (*Paraliparis* stet., BI1369), representing one of the first two recorded uses of xenophyophores as nursery habitat by fishes (Levin and Rouse 2020).

***Shinkaiya* stet.**

Fig. 80H

**Reference.**Levin and Rouse 2020.

**Localities.** Mound Jaguar (1902 m).

**Remarks.** This specimen (destroyed) was associated with snailfish eggs (*Acantholiparis* stet., BI1371) (Levin and Rouse 2020).

***Syringammina* stet.**

Fig. 81A

**Figure 81.**
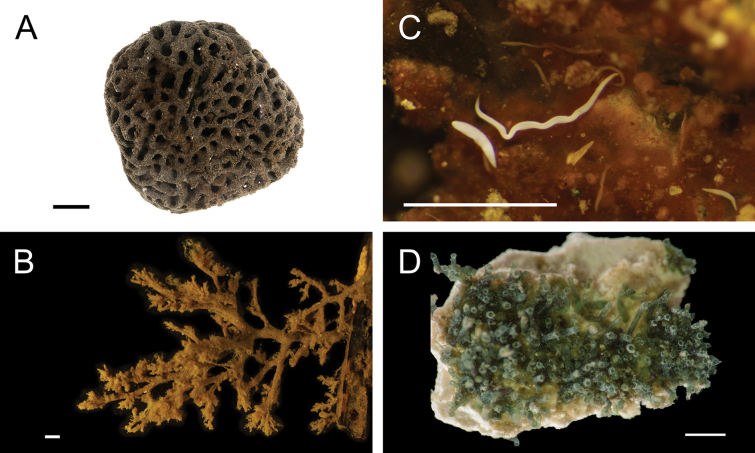
Foraminifera and Ciliophora, representative live images **A***Syringammina* stet. (BI1158) **B**Foraminifera stet. (BI1164) **C**Ciliophora stet. (BI1173) **D***Eufolliculinacaerulea* (BI1051). Scale bars: 1 cm (**A**); 1 mm (**B–D**).

**Material examined.** AD4913: BI1158.

**Localities.** Jacó Scar (1885 m).

#### ﻿Chromista | Foraminifera

**Foraminifera stet.**

Fig. 81B

**Material examined.** AD4921: BI1153, BI1164; S0219: BI1366.

**Localities.** Quepos Slide (~ 345–394 m), Rio Bongo Scar (661 m).

**Remarks.** Arborescent foraminiferans associated with naturally occurring wood falls.

### ﻿﻿Ciliophora

**Ciliophora stet.**

Fig. 81C

**Material examined.** AD4918: BI1173.

**Localities.** Quepos Slide (~ 333–408 m).

**Remarks.** Motile ciliates were associated with a bacterial film on a carbonate rock from a site of active seepage.

***Eufolliculinacaerulea* Pasulka & Rouse in Pasulka et al. 2017**

Fig. 81D

**Reference.**Pasulka et al. 2017**.

**Localities.** Mound 12 (~ 1000 m).

**Distribution.** Also known from eastern Pacific seeps off southern California (type locality: Split Ridge, off San Diego, 865 m) and the Guaymas Basin, total depth range 865–1565 m (Pasulka et al. 2017).

## ﻿﻿Results

### ﻿﻿Taxonomic identification

We report 488 species-level taxa (i.e., distinct taxonomic units in the sense of morphospecies, though often distinguished by genetics rather than morphology) occurring at the CRM seeps, including 131 described (formally named) species and at least 58 undescribed species new to science (Table 3, Suppl. material 1: table S1). The remaining 299 species-level taxa present some degree of uncertainty in their identification. The 488 total taxa include 155 annelids, 96 crustaceans, 42 cnidarians, 55 echinoderms, 108 mollusks, 9 nemerteans, 17 other animals, and 6 chromists. Of the 131 described species, 49 were originally described from the CRM seeps.

**Table 3.** Summary of taxa occurring at the CRM seeps. “Undescribed species” indicates species that have been identified with certainty as new to science but have not yet been formally described and named. “Taxa with taxonomic uncertainty” indicates distinct taxonomic units that could not be identified with certainty to the level of species, e.g., “Parougiacf.sulleyi” or “Chaetognatha stet.”

Higher taxonomy	# described species (# which were originally described from the CRM seeps)	# undescribed species noted in this work	# taxa with taxonomic uncertainty	Total	# species with new biogeographic records at the CRM seeps
Annelida	38 (27)	35	82	155	2
Arthropoda	30 (4)	4	62	96	4
Cnidaria	5 (2)	0	37	42	0
Echinodermata	20 (1)	9	26	55	5
Mollusca	28 (7)	8	72	108	4
Nemertea	6 (6)	1	2	9	0
Other Animalia	3 (1)	1	13	17	0
Chromista	1 (1)	0	5	6	0
**Total**	**131 (49)**	**58**	**299**	**488**	**15**

### ﻿﻿New records

We report 15 range extensions for species occurring in the eastern Pacific: five new northern records, nine new southern records, and one new eastern Pacific record (Table 4, Suppl. material 1: table S1). We also report 16 new depth records (6 new minimum depths, 10 new maximum depths), and 3 new seep records of species known to occur at vents or organic falls (Table 4, Suppl. material 1: table S1).

**Table 4.** New biogeographic records. New northern and southern records are indicated by the location of the previously reported limit. New depth records were defined as a minimum difference of 100 m from a previously reported minimum or maximum depth. New seep records pertain to species previously reported from vents or organic falls. Only occurrences reported in peer-reviewed publications were included in these assessments; other occurrences of potential interest, e.g., unpublished data or GBIF records, are discussed in the text.

Higher taxonomy	Species	Known biogeographic range (including this work)	New biogeographic records reported in this work	Known depth range (including this work)	New minimum depth record	New maximum depth record	New seep records reported in this work
Annelida	*Lindaspiodibranchiata*	Gulf of California to CRM	new southern record (previously Guaymas Basin only)	820–2008 m	820 m (previously 2004 m)		new seep record (previously from sedimented vents)
Annelida	*Myzostomajosefinae*	central California to CRM	new southern record (previously to the Guaymas Basin)	1020–1878 m		1878 m (previously ~ 1350 m)	
Arthropoda	*Alvinocariscostaricensis*	CRM only		742–1800 m	742 m (previously ~ 1000 m)		
Arthropoda	*Heteroptychusgalapagos*	CRM to Galápagos	new northern record (previously Galápagos only)	1012–1037 m			
Arthropoda	*Lebbeusscrippsi*	Gulf of California to northern Chile		768–1768 m		1768 m (previously 1245 m)	
Arthropoda	*Munidopsisalvisca*	Juan de Fuca Ridge to CRM	new southern record (previously to the Guaymas Basin)	1401–2008 m	1401 m (previously 1545 m)		new seep record (previously from vents)
Arthropoda	*Spongicoloidesgalapagensis*	CRM to Galápagos	new northern record (previously Galápagos only)	717–1091 m		1091 m (previously 717 m)	
Arthropoda	*Sternostylusdefensus*	CRM to Galápagos	new northern record (previously Galápagos only)	717–1210 m			
Cnidaria	*Aquaumbraklapferi*	CRM to Isla del Coco; Gulf of Mexico		268–610 m		610 m (previously 421 m)	
Echinodermata	*Araeosomaleptaleum*	California to Galápagos		740–1271 m		1271 m (previously 1106 m)	
Echinodermata	*Calamocrinusdiomedae*	CRM to Galápagos		717–1659 m		1659 m (previously 1430 m)	
Echinodermata	*Ophiacanthamoniliformis*	southern California to CRM	new southern record (previously to southern Mexico)	377–1244 m			
Echinodermata	*Ophiocreascarnosus*	CRM to southern Chile	new northern record (previously central Chile)	175–1632 m		1632 m (previously 983 m)	
Echinodermata	*Ophioleucegracilis*	Pacific, widely distributed		1710–2882 m	1710 m (previously ~ 2000 m)		
Echinodermata	*Ophiomitrapartita*	central Mexico to CRM	new southern record (previously Islas Marías only)	1236–1887 m		1887 m (previously 1236 m)	
Echinodermata	*Ophiophruraliodisca*	western Pacific; CRM	new eastern Pacific record (previously Japan to New Zealand)	606–1620 m			
Echinodermata	*Thrissacanthiaspenicillatus*	Washington to CRM	new southern record (previously to the Gulf of California)	55–1503 m			
Mollusca	*Belknapchitonhalistreptus*	southern Mexico to CRM	new southern record (previously southern Mexico only)	609–3436 m	609 m (previously 902 m)		
Mollusca	*Delectopectenvancouverensis*	Alaska to CRM; Bering Sea to Sea of Japan	new southern record (previously to Baja California)	20–4100 m			
Mollusca	*Ennuculacolombiana*	CRM to Peru		11–997 m		997 m (previously 734 m)	
Mollusca	*Eulimellalomana*	southern California to CRM	new southern record (previously to the Guaymas Basin)	1168–2008 m			
Mollusca	*Tindariopsisgrasslei*	Gulf of California to CRM		992–2012 m	992 m (previously 1400 m)		
Mollusca	*Tripoplaxbalaenophila*	CRM to central Chile	new northern record (previously central Chile only)	240–344 m		344 m (previously 240 m)	new seep record (previously from bone)
		Total	15		6	10	3

### ﻿﻿Biogeographic ranges

Of the 131 described species occurring at the CRM seeps, 38 are known only from Costa Rica/Panama, 64 occur elsewhere in the eastern Pacific, six also occur through the northern Pacific to Japan, six occur more broadly in the Pacific, and 17 occur in multiple ocean basins (Table 5, Suppl. material 1: table S1).

**Table 5.** Known biogeographic ranges of the described species occurring at the CRM seeps. Numbers indicate the count of described species within each category.

Eastern Pacific		Northern range limit			
		Costa Rica/ Panama	Mexico	California	Juan de Fuca region
**Southern range limit**	Costa Rica/Panama	38	10	10	7
	Ecuador	7	2	1	0
	Peru/northern Chile	3	8	3	2
	Central Chile	4	1	1	1
	Southern Chile	1	1	1	1
**Northern Pacific** (Japan to Costa Rica/Panama)		6			
**Pacific, broadly**		6			
**Multiple ocean basins**		17			

### ﻿﻿Occurrences at other chemosynthesis-based habitats

Of the 131 described species occurring at the CRM seeps, 13 also occur at hydrothermal vents, 14 also occur at organic falls, and six occur at all three types of chemosynthesis-based habitats (Table 6, Suppl. material 1: table S1).

**Table 6.** Known occurrences at other chemosynthesis-based habitats for described species occurring at CRM seeps. The count of species in each category is indicated in the bottom row. New seep records of species previously reported from vents or organic falls are indicated in bold. The new seep records were collected from naturally occurring seep substrates rather than wood or bone deployments.

Seeps + vents	Seeps + vents + organic falls	Seeps + organic falls
*Amphisamythafauchaldi*	*Archivesicagigas*	*Alvinonemerteschristianeae*
*Archinomelevinae*	*Escarpiaspicata*	*Escondidacantraineapanamensis*
*Calyptogenadiagonalis*	*Eulimellalomana*	*Heterocarpusvicarius*
*Bathymodiolusthermophilus*	*Phreagenasoyoae*	*Micospinaauribohnorum*
*Calyptogenacostaricana*	*Provannalaevis*	*Munidopsiscarinipes*
*Galapagomystidesverenae*	*Xyloplaxprincealberti*	*Munidopsishendersoniana*
*Lamellibrachiabarhami*		*Myzostomajosefinae*
*Lepetodrilusguaymasensis*		*Osedaxfrankpressi*
***Lindaspiodibranchiata***		*Osedaxknutei*
***Munidopsisalvisca***		*Peinaleopolynoeelvisi*
*Phreagenaextenta*		*Peinaleopolynoemineoi*
*Provannaios*		*Provannapacifica*
*Shinkailongipedata*		***Tripoplaxbalaenophila***
		*Typhlonidapropinqua*
**13**	**6**	**14**

## ﻿﻿Discussion

### ﻿﻿Biodiversity at the CRM seeps

The CRM seeps support a biodiverse fauna and have been a rich site of species discovery. Of the 131 described species occurring at these seeps, 49 were newly described from this area (Table 3, Suppl. material 1: table S1), beginning with the vesicomyid clam *Calyptogenadiagonalis* in 1999 (Barry and Kochevar 1999). Of these 49 species, 47 (excluding *C.diagonalis* and *C.costaricana*) were originally described from the cruises summarized in this work. Many of the 58 undescribed species identified in this study will be formally described in due course. With further work, it may be possible to identify the remaining 299 “species” reported with taxonomic qualifiers as known matches to described species, or to ascertain that they are additional undescribed species.

The extensive field sampling program in this study has been essential for these discoveries. For the 488 taxa reported to occur at the CRM seeps, all occurrence records included material collected during the five cruises of this study, except for five previously published vesicomyid clam species: *Archivesica* sp. 7 sec. Audzijonyte et al. 2012, *Calyptogenacostaricana*, *C.* sp. 3 sec. Audzijonyte et al. 2012, *C.* sp. mt-V sec. Goffredi et al. 2003, and *Phreagenaextenta*. These five species were collected during prior work at deeper seep sites northwest of those targeted in this study (Peek et al. 2000; Goffredi et al. 2003; Audzijonyte et al. 2012). Further biological sampling at the northwestern seeps and others at comparable depths (3000–4000 m; e.g., Subduction Plume) may elucidate whether these clams are restricted by depth and/or local habitat conditions.

### ﻿﻿Comparison to checklists for Costa Rica

An extensive catalog has been compiled for the overall marine biodiversity of Costa Rica by Wehrtmann and Cortés (2009b). At least 4690 marine species are known from the entire Pacific coast, with most records from shallow waters (Cortés 2012). This study adds six species to the checklist of 75 echinoderms reported from Costa Rican waters at > 200 m depth (Alvarado et al. 2022) (*Ophiacanthainconspicua*, *Ophiacanthamoniliformis*, *Ophiocreascarnosus*, *Ophiomitrapartita*, *Ophiophruraliodisca*, *Xyloplaxprincealberti*). This study also adds 21 species to the 139 crustaceans recorded from the Costa Rican Pacific at > 200 m depth (Azofeifa-Solano and Cortés 2020) (*Colossendeismacerrima*, *Eucopiasculpticauda*, *Heteroptychusgalapagos*, *Lebbeusscrippsi*, *Lycaeapulex*, *Mesopleustesabyssorum*, *Munidopsisagassizii*, *Munidopsisalvisca*, *Munidopsiscarinipes*, *Munidopsiscortesi*, *Munidopsisgirguisi*, *Munidopsisgranosicorium*, *Munidopsishendersoniana*, *Munidopsissimilis*, *Nematocarcinusfaxoni*, *Oncopagurushaigae*, *Paracrangonareolata*, *Parapagurusforaminosus*, *Spongicoloidesgalapagensis*, *Sternostylusdefensus*, *Typhlonidapropinqua*). Previous deep-sea work based on the 2009 and 2010 cruises (Levin et al. 2012, 2015) included lists of taxa in the supplemental material. We have referenced those records where identifications could be confidently matched to extant voucher material; many of those listings reflect provisional identifications based on morphology and have since been revised to reflect taxonomic work on reference specimens at SIO-BIC.

### ﻿﻿Comparison to global seep checklists

The 131 described species recorded from the CRM seeps represent a substantial portion of the ~ 600 species recorded from all > 100 seep sites worldwide as of 2011 (German et al. 2011), and of 863 species currently recorded from all chemosynthetic ecosystems worldwide (Ramírez-Llodra 2024). With increased discovery and biological characterization of seeps in recent decades, this global list has expanded approximately threefold. As of 1998, only 211 species (of which 116 were named) had been reported from all 24 seep sites known worldwide at the time (Sibuet and Olu 1998).

### ﻿﻿Comparison to seep-adjacent background habitats

Mobile fauna known from non-seep “background” habitats can utilize and aggregate at seeps (Carney 1994; Sibuet and Olu 1998; Tunnicliffe et al. 1998; Niemann et al. 2013; Levin et al. 2016). As discussed by Carney (1994), any attempt to categorize fauna as “vagrants, colonists, or endemics” with respect to seeps depends on an accurate and thorough inventory of the surrounding environment, which falls outside the scope of this study. We therefore did not assign such categorizations to the fauna reported in this study, and we echo caution in interpreting fauna as seep-endemic or regionally endemic based on absence of evidence.

Several species reported from the shallower CRM seeps in this work (Quepos Seep, Rio Bongo Scar, and Jacó Summit) were previously well known from shelf and slope depths (< 350 m) inshore of the seeps, e.g., the commercially harvested shrimp *Heterocarpusvicarius* and abundant bycatch crustaceans: *Grimotheamonodon*, *Plesionikatrispinus*, and *Squillabiformis* (Wehrtmann and Echeverría-Sáenz 2007; Wehrtmann et al. 2010). Studies of seeps on the northern California slope (~ 500 m depth) (Levin et al. 2000) and the Koryak slope in the Bering Sea (400–700 m) (Rybakova et al. 2022) have also reported strong similarity between seep and non-seep faunas, remarking that the seep fauna at these depths may be pre-adapted to the organic-rich reducing conditions at seeps (Levin et al. 2000).

We acknowledge the importance of future work on the non-seep habitats of the CRM, especially seamounts (Wehrtmann and Cortés 2009a; Cortés 2012). Several seamounts occur at similar depths as the deeper seeps, e.g., Quepos Plateau and Seamount 1 at 1410–2184 m (Hatch et al. 2020; Sagorny et al. 2022), and share geological characteristics with seeps such as Jacó Scar, Parrita Scar, and Rio Bongo Scar (Sahling et al. 2008). The CRM seep sediments also show spatial gradients of macrofaunal assemblages and geochemistry, described in detail in Ashford et al. (2021b). Sampling in this work was centered on sites of active seepage and their transition zone (tens to hundreds of meters) into the surrounding environment, but more extensive sampling of far-transition and background sites (~ 1 km and beyond) at various localities would help to evaluate the full extent of this transition zone.

### ﻿﻿Comparison to other seep, vent, and organic fall sites

Although site-specific taxonomic inventories are not available for many seeps and direct comparisons are complicated by differences of methodology and scope, the count of 488 distinct morphospecies reported at the CRM seeps in this study exceeds those published for other seep sites to date. At least 335 macrofaunal morphospecies occur at the Koryak slope seeps in the Bering Sea (Rybakova et al. 2022), 289 at the Laptev Sea seeps in the Siberian Arctic (Vedenin et al. 2020), 134 at Hydrate Ridge off Oregon (Levin et al. 2017), 101 at the Concepción seeps off central Chile (Sellanes et al. 2008), 83 at the seeps of El Pilar off Trinidad and Tobago (Amon et al. 2017), 65 at the Haima seeps in the South China Sea (He et al. 2023), 65 at the Gulf of Mexico seeps (Bergquist et al. 2003), 60 at the Del Mar seep off southern California (Grupe et al. 2015), 60 at the Taitao Peninsula seep off southern Chile (Zapata-Hernández et al. 2014), 50–60 at the Florida Escarpment and Blake Ridge seeps in the western Atlantic (Turnipseed et al. 2003), 30 at the Guaymas Basin seeps (Portail et al. 2015), and 20 at the South Chamorro Seamount serpentinite-hosted seep on the Mariana Forearc (Chen et al. 2024c).

Recent macrofaunal inventories from vents have reported 130 morphospecies from the Piip Volcano in the Bering Sea (Rybakova et al. 2023), 92 from the Galápagos Rift (Chen et al. 2024b), 91 from the Okinawa Trough vent system (Tunnicliffe et al. 2024), 12–70 at each of ten other western Pacific vent systems (Tunnicliffe et al. 2024), 62 from the Loki’s Castle Vent Field on the Arctic Mid-Ocean Ridge (Eilertsen et al. 2024), and 29 from the Amami Rift vent field off southern Japan (Chen et al. 2024a). Individual whale falls off California have been reported to support 36–190 macrofaunal species (Baco and Smith 2003; Lundsten et al. 2010). Experimental wood deployments in the Gulf of Mexico (McClain et al. 2023) and southwestern Atlantic (Saeedi et al. 2019) have been reported to support 44 and 114 macrofaunal invertebrate species, respectively.

### ﻿﻿Depth ranges at the CRM seeps

At the CRM seeps, pairs of morphologically indistinguishable yet genetically distinct species can be separated by depth. The serpulid tubeworms *Laminatubusjoycebrooksae* and *L.paulbrooksi* occur at ~ 1000 m and ~ 1400–2400 m, respectively (Rouse and Kupriyanova 2021). The chemosymbiont-bearing mussels *Bathymodiolusnancyschneiderae* (1000–1100 m) and *B.billschneideri* (1400–1900 m) occupy non-overlapping depths, yet the morphologically distinct *B.earlougheri* co-occurs with both species across their combined depth range (McCowin et al. 2020). Furthermore, the four species of symbiotic scaleworms (*Branchipolynoe* spp.) that occupy the mantle cavity of these mussels show no apparent depth or host specificity (Lindgren et al. 2019). Although the effects of depth are difficult to disentangle from those of seep geochemistry and other parameters, these examples show that a putative morphospecies can comprise at least two depth-segregated cryptic species, requiring careful taxonomy and potentially different management considerations.

### ﻿﻿Range extensions reinforce eastern Pacific biogeographic connections

The range extensions in this study reinforce the biogeographic connections of the CRM seeps to neighboring regions of the eastern Pacific, from the Juan de Fuca Ridge to southern Chile. The smallest range extensions were new northern records of species previously known only from the Galápagos (representing ~ 8 degrees of latitude and ~ 1,100 km). The greatest eastern Pacific range extension was the new northern record of *Tripoplaxbalaenophila*, previously known only from central Chile (representing ~ 45 degrees of latitude and ~ 5,100 km).

#### ﻿Mexico and Gulf of California

The Middle America Trench connects the CRM to seeps off western Mexico. The East Pacific Rise (EPR) links the Cocos Plate to seeps and vents in the Gulf of California (Kimura et al. 1997; Fiedler and Lavín 2006), including those of the Guaymas Basin (Portail et al. 2015) and the Pescadero Transform Fault (Goffredi et al. 2017). Of the 131 described species at the CRM, 79 species (60%) also range to Mexico or beyond, and 10 of these species are presently known only from the CRM and Mexico. The nine new southern records reported in this work represent range extensions from Mexican waters, and we expect that further deep-sea sampling in both regions will reveal additional overlap. In particular, the Guaymas Basin seeps at ~ 2000 m share several high-biomass, habitat-forming species with the CRM seeps (vestimentiferan tubeworms *Lamellibrachiabarhami* and *Escarpiaspicata*, vesicomyid clams *Archivesicagigas* and *Phreagenasoyoae*) (Portail et al. 2015), suggesting broad similarity of the two communities.

#### ﻿Galápagos and Coco Submarine Volcanic Range

The Galápagos seamounts, rising from the abyssal plain at ~ 3000 m, are connected to Costa Rica by the Coco Submarine Volcanic Range, which was formed by the Galápagos Hotspot and subducts beneath the CRM (Kimura et al. 1997; von Huene et al. 2000; Fiedler and Lavín 2006). The seamount subduction section of the CRM shares the characteristic geochemistry of the Galápagos seamounts (von Huene et al. 2000; Sahling et al. 2008), suggesting biogeographical affinities as well. Of the 131 described species at the CRM, 67 species (51%) also range to the Galápagos region or beyond (including five northern range extensions), and seven species are presently known only from the CRM and Galápagos.

The first systematic ROV characterization of deep-sea benthic invertebrate communities at the Galápagos seamounts (290–3373 m) reported 70 morphospecies based on collection of 90 specimens and extensive imagery (Salinas-de-León et al. 2020). Thirty of those morphospecies were undescribed and new to science at the time, including the squat lobster ﻿*Heteroptychusgalapagos*, which we report here from the CRM seeps as a range extension. These examples illustrate the extent of undiscovered biodiversity along the Coco Submarine Volcanic Range and the importance of FAIR taxonomic inventories (Howell et al. 2020) in describing this biodiversity. Under circumstances where certain data cannot be shared publicly, e.g., permit conditions noted in Salinas-de-León et al. (2020), datasets from biogeographically connected regions such as the CRM will be important for studies of comparative biodiversity.

Located approximately halfway between the Galápagos seamounts and the CRM, Isla del Coco (Cocos Island) is thought to have high rates of endemism, with 1688 marine species recorded and 747 of these not found elsewhere in Pacific Costa Rica (Cortés 2012). The deepest regions of Isla del Coco (> 450 m) remain under-explored as they are beyond the capability of the locally operating DeepSee submersible and may reveal further biogeographic linkages along the Coco Submarine Volcanic Range. For example, the soft coral *Aquaumbraklapferi* was previously known from Isla del Coco, but here we also report it from Rio Bongo Scar, establishing a new depth record and an association with seeps.

#### ﻿Peru-Chile margin

Seeps supporting diverse fauna occur along the Peru-Chile margin (Olu et al. 1996; Sellanes et al. 2008), and this region is an important focus area for further biological investigation (Ramírez Llodra et al. 2003; German et al. 2011; Miloslavich et al. 2011; Tapia-Guerra et al. 2021; Wagner et al. 2021). Of the 131 described species at the CRM, 57 species (44%) also range to Peru or beyond, including two northern range extensions. Additional taxonomic inventories along the Peru-Chile margin (e.g., Valentich-Scott et al. 2020) will be instrumental in addressing biogeographic knowledge gaps, such as the extent of connectivity between the eastern Pacific and Antarctica.

#### ﻿Trans-Pacific and broader ranges

Six of the 131 described species in this work (5%) reportedly also range to Japan, e.g., via the Alaska margin. Four of these species have been confirmed to show trans-Pacific genetic connectivity (*Archivesicagigas*, *Phreagenaextenta*, *Phreagenasoyoae*, *Provannalaevis*), whereas range-wide genetic data are not currently available for the others (*Delectopectenvancouverensis*, *Shinkailongipedata*).

Twenty-three of the described species in this work (18%) reportedly have even wider distributions, extending into the southwestern Pacific or other ocean basins. Some of these examples are corroborated by genetics (e.g., *Osedaxfrankpressi*, *Munidopsissimilis*), whereas others are suspected to represent cryptic species complexes or synonymies warranting resurrection (e.g., *Ophiuraflagellata*).

### ﻿﻿Other biogeographic connections

An important geological factor in the evolutionary history of the CRM seeps is the separation of the Atlantic and Pacific Oceans by the rise of the Isthmus of Panama (O’Dea et al. 2016). For example, the polychaete *Amphisamythafauchaldi* from the CRM seeps is most closely related to the Atlantic *A.lutzi* (Desbruyères & Laubier, 1996) rather than to any other Pacific *Amphisamytha* (Stiller et al. 2013). Similarly, *Lamellibrachiadonwalshi* from the CRM seeps is more closely related to a clade of Atlantic species, including *L.anaximandri* Southward, Andersen & Hourdez, 2011, than to any Pacific *Lamellibrachia* (McCowin and Rouse 2018). The soft coral *Aquaumbraklapferi* has been reported from relatively shallow eastern Pacific and western Atlantic waters (Breedy et al. 2012; Quattrini et al. 2020), consistent with these populations being separated by the rise of the Isthmus of Panama relatively recently compared to deeper-water species.

Where several congeners occur at the CRM seeps, the CRM species are not necessarily sister taxa to one another, and their true relationships reveal a variety of biogeographic affinities. For example, *Lamellibrachiabarhami* is more closely related to *L.satsuma* Miura, 1997 from the western Pacific than to *L.donwalshi* (McCowin and Rouse 2018). *Bathymodiolusnancyschneiderae* is more closely related to a western Pacific *Bathymodiolus* clade than to *B.billschneideri* and *B.earlougheri*, which are sister taxa and form an eastern Pacific clade with *B.thermophilus* (also occurring at the CRM seeps) and *B.antarcticus* (McCowin et al. 2020). As with depth ranges, the patterns for the four CRM seep *Branchipolynoe* species do not track those of their mussel hosts: *B.halliseyae* and *B.kajsae* are sister taxa and most closely related to *B.seepensis* from the western Atlantic, whereas *B.eliseae* and *B.meridae* belong to an eastern Pacific clade along with *B.symmytilida* from the EPR (Lindgren et al. 2019). Together, these examples indicate evolutionary linkages from the CRM to the EPR, western Atlantic, and western Pacific.

### ﻿﻿Intersection of seeps, vents, and organic remains

The first submersible-based accounts of deep-sea fauna at seeps (Paull et al. 1984) and whale remains (Smith et al. 1989) immediately noted the similarity to hydrothermal vent communities. Subsequent comparisons of these three ecosystems have depended on comprehensive lists of well identified taxa (Sibuet and Olu 1998; Tunnicliffe et al. 2003; Watanabe et al. 2010; Bernardino et al. 2012; Kiel 2016).

The biogeographic and evolutionary connections among these ecosystems are complex. Hydrothermal vents and seeps can occur in close geographical proximity, as known from the Guaymas Basin (Simoneit et al. 1990; Portail et al. 2015), Edison Seamount off Papua New Guinea (Herzig et al. 1998), and several regions of Japan (Watanabe et al. 2010). Vents and seeps may intersect with organic falls where they converge with forested coasts, kelp beds, or whale migration routes (Smith and Baco 2003; Bernardino et al. 2012). Furthermore, sedimented vents such as those at Manus Basin, the Guaymas Basin, Escanaba Trough at Gorda Ridge, and the Middle Valley on the Juan de Fuca Ridge, are considered intermediate environments by combining the soft substrate and fluid chemistry characteristic of seeps with the elevated temperatures and metal concentrations characteristic of bare-rock vents (Tunnicliffe et al. 1998, 2003; Levin et al. 2009; Bernardino et al. 2012; Kiel 2016).

### ﻿﻿Overlap of species occurrences

Worldwide, at least ~ 700 morphospecies occur at hydrothermal vents and at least 407 occur at organic remains, compared to ~ 600 at seeps (Smith and Baco 2003; German et al. 2011; Chapman et al. 2019). Comparative studies attempting to control for these conditions and other variables have suggested that seep communities tend to show higher diversity than comparable vent communities (Turnipseed et al. 2003; Bernardino et al. 2012; Portail et al. 2015).

Globally, at least 24 species co-occur at seeps and vents (Portail et al. 2015), at least 20 co-occur at seeps and organic remains (Smith and Baco 2003), and at least 11 co-occur at organic remains and vents (mainly sedimented vents at the Guaymas Basin and the Juan de Fuca Ridge) (Smith and Baco 2003). At least eight species occur at all three habitat types: *Calyptogenaelongata*, *Calyptogenapacifica*, *Chiridotaheheva*, *Cocculinacraigsmithi*, *Eulimellalomana*, *Escarpiaspicata*, *Idaswashingtonia*, *Pyropeltacorymba*, and *P.musaica* (Smith et al. 1989; Tunnicliffe et al. 1998; Smith and Baco 2003; Thomas et al. 2020). At least two of these eight species occur at the CRM seeps (*Eulimellalomana* and *Escarpiaspicata*; Pyropeltacf.corymba and P.cf.musaica require genetic confirmation). In this study, we also add two species to the list of overlap between seeps and vents (*Lindaspiodibranchiata*, *Munidopsisalvisca*), and one species (*Tripoplaxbalaenophila*) to the list of overlap between seeps and whale remains (Table 6).

### ﻿﻿Modes of symbiosis

Modes of chemosynthetic symbiosis can be important for explaining and predicting the occurrence of symbiont-bearing taxa across chemosynthesis-based habitats. At the CRM seeps, all three species of vestimentiferan tubeworms host the same thiotrophic symbiont, whereas the three numerically dominant mussels each host a unique thiotrophic symbiont (Brzechffa and Goffredi 2021). Methanotrophic examples from the CRM seeps include the serpulid worm *Laminatubuspaulbrooski* and the sabellid worm *Bispira* sp. SIO_BIC_A9587 (Goffredi et al. 2020; Rouse and Kupriyanova 2021). Farming of epibiotic ﻿bacteria (likely thiotrophic or methanotrophic) by the yeti crab *Kiwapuravida* represents another conspicuous mode of symbiosis at the CRM seeps (Thurber et al. 2011; Goffredi et al. 2014).

### ﻿﻿Areas for further work

Despite our intensive and collaborative sampling efforts, we acknowledge that this inventory is still surely incomplete.

#### ﻿Geochemistry

The seep sites in this study were located in the central CRM spanning the “mound segment” and the “seamount subduction segment” (Sahling et al. 2008), thereby covering habitats with different geochemical characteristics. Faunal sampling at additional seeps, especially at the distal ends of each segment, may reveal additional diversity and suggest new hypotheses about the ecology and biogeography of this region.

#### ﻿Depth

The depths of sampling locations in this study (~ 300–3600 m) span the known bathymetric range of the entire CRM (Sahling et al. 2008) and approximately half the known depth range for seeps globally (~ 300–7400 m) (Sibuet and Olu 1998; Fujikura et al. 1999; Watanabe et al. 2010). Most of the sampling effort in this study occurred between 400–1800 m (Table 1), so further sampling of the deepest seeps may yield additional new records, species, and ecological findings.

#### ﻿Pelagic linkages

This study focused on benthic habitats, but the influence of seep and vent plumes on benthopelagic and pelagic fauna warrants further investigation (Levin et al. 2016). The opportunistically collected pelagic samples in this study have provided vouchered reference sequences for animals that are generally challenging to collect (e.g., the holopelagic sea cucumber *Pelagothurianatatrix*). The few pelagic fauna encountered on the seafloor (pyrosomes and an undescribed scyphomedusa) offer potential examples of trophic linkage between the CRM seeps and the overlying water column.

## ﻿﻿Conclusions

### ﻿﻿Importance of regional taxonomic inventories

Regional taxonomic inventories provide essential baseline biodiversity data for ecosystem science, monitoring, and management. Species occurrence data form the foundation of trait databases and biogeographic delineations, all of which can inform the design of effective ecological reserves (Van Dover et al. 2012; Chapman et al. 2019). Consequently, knowledge gaps in taxonomy and biogeography are a major impediment to deep-sea conservation, particularly of invertebrates (Cardoso et al. 2011; Ramírez-Llodra et al. 2011; Glover et al. 2018; Sigwart et al. 2023).

This inventory adds to the extensive existing documentation of marine biodiversity in Costa Rica, including recent emphasis on the deep sea (Wehrtmann and Cortés 2009a; Alvarado et al. 2010, 2012, 2022; Cortés 2012; Azofeifa-Solano and Cortés 2020). Much of the rich terrestrial and marine biodiversity of Costa Rica, including 50% of the coastline and an area of seamounts surrounding Isla del Coco National Park, has received conservation status as Protected Areas or other designations (Alvarado et al. 2012).

Recent quantitative economic research has demonstrated that Costa Ricans place high value on their seeps, with a willingness to pay for seep protection being highest for programs that protect seeps with endemic species (Pereira et al. 2024). In a related survey of deep-sea care and understanding, Costa Ricans ranked the provision of habitat and biodiversity as their most important connection to the deep sea (Pereira et al. 2024). Thus, continued biodiversity discovery is significant to the people of Costa Rica and to the stewardship of their deep marine ecosystems. We hope that the biodiversity data in this work will inform future marine policy in Costa Rica and the tropical eastern Pacific.

With thousands of seep sites and hundreds of vent sites estimated to exist across the deep sea (Levin et al. 2016), the ability to make direct and repeatable biodiversity comparisons is important for global research and policy-building. We advocate for the inclusion of genetic data in taxonomic inventories to enhance the robustness of taxonomic identifications and to allow evaluation of species ranges and assessment of population connectivity. The combination of museum voucher specimens, morphologically informative images, and diagnostic DNA sequences establishes critical reference libraries for broader scientific, educational, and policy uses (e.g., Glover et al. 2015). For example, the abyssal fauna of the Clarion-Clipperton Zone are being inventoried in a growing body of specimen-based, DNA-supported taxonomic work (Dahlgren et al. 2016; Glover et al. 2016; Christodoulou et al. 2020; Bribiesca-Contreras et al. 2022).

### ﻿﻿Recommendations

For taxonomic inventories to maximize their potential, we emphasize the importance of careful specimen processing and molecular taxonomy, supported by deep submergence technology and biological collections.

Submersibles enable high-quality genetic and morphological analyses through targeted collection, precise data logging, high-resolution *in situ* imaging, and live recovery of intact animals. These vehicles, equipped with appropriate sampling and imaging tools, are particularly important for work on deep chemosynthetic ecosystems which are not well suited to trawls and other nonselective sampling gear. Reliable access to, and support for, deep-sea technologies remain essential to the advancement of deep-sea research (Liang et al. 2021; Marlow et al. 2022; Miller and Virmani 2023).

During specimen processing at sea, clear documentation and judicious field protocols are critical for scientific and legal traceability (Gemeinholzer et al. 2010; Kroupa and Remsen 2010; Templado et al. 2010; Glover et al. 2015; Howell et al. 2020). For example, unique identifiers (alphanumeric codes, ideally pre-printed on indelible labels) are important for linking physical samples to metadata, digital sequence information, and permissible downstream applications. Not only are voucher specimens critical for the integrity of taxonomic and genomic research, but it is important to record the relationship of the voucher to the tissue used to generate sequence data (i.e., same individual or a proxy) (Pleijel et al. 2008; Buckner et al. 2021). These field practices facilitate the deposition of well-curated physical specimens and their associated data into accessible repositories.

Biological collections have been central to this study and serve as foundations for biodiversity research, among other scientific and societal benefits (Suarez and Tsutsui 2004; National Science and Technology Council Committee on Science Interagency Working Group on Scientific Collections 2009; National Academies of Sciences Engineering and Medicine 2020). We intend for this inventory to underscore the importance of biological collections and to serve as an integrative resource for future studies of biodiversity and biogeography at deep-sea ecosystems.

## ﻿﻿Additional information

### Author ORCIDs

Charlotte A. Seid https://orcid.org/0000-0002-5307-691X

Avery S. Hiley https://orcid.org/0000-0001-8956-5080

Marina F. McCowin https://orcid.org/0000-0001-9674-5557

José I. Carvajal https://orcid.org/0000-0003-2255-9705

Shane T. Ahyong https://orcid.org/0000-0002-2820-4158

Oliver S. Ashford https://orcid.org/0000-0001-5473-7057

Odalisca Breedy https://orcid.org/0000-0001-5686-4164

Douglas J. Eernisse https://orcid.org/0000-0002-5457-7484

Shana K. Goffredi https://orcid.org/0000-0002-9110-9591

Michel E. Hendrickx https://orcid.org/0000-0001-9187-6080

Kevin M. Kocot https://orcid.org/0000-0002-8673-2688

Christopher L. Mah https://orcid.org/0000-0002-0178-8237

Allison K. Miller https://orcid.org/0000-0002-5334-2771

Nicolás Mongiardino Koch https://orcid.org/0000-0001-6317-5869

Rich Mooi https://orcid.org/0000-0003-1533-8488

Timothy D. O’Hara https://orcid.org/0000-0003-0885-6578

Josefin Stiller https://orcid.org/0000-0001-6009-9581

Ekin Tilic https://orcid.org/0000-0003-0463-322X

Paul Valentich-Scott https://orcid.org/0000-0003-0019-7643

Mary K. Wicksten https://orcid.org/0000-0002-9097-353X

Nerida G. Wilson https://orcid.org/0000-0002-0784-0200

Erik E. Cordes https://orcid.org/0000-0002-6989-2348

Lisa A. Levin https://orcid.org/0000-0002-2858-8622

Jorge Cortés https://orcid.org/0000-0001-7004-8649

Greg W. Rouse https://orcid.org/0000-0001-9036-9263

### Data availability

Sequences are available on GenBank and linked to BioProject PRJNA1142170 (CRM specimens, 274 new sequences) or PRJNA1136884 (comparative material from other localities, 5 new sequences). SIO-BIC specimen records are available and continually updated through the online collection database (https://sioapps.ucsd.edu/collections/bi/). The SIO-BIC specimen records are also available, with less frequent updates, as a DarwinCore occurrence dataset via the Global Biodiversity Information Facility (https://www.gbif.org/dataset/a267b6a7-91f9-457c-889a-481e7aa920b6).

## Supplementary materials

﻿Supplementary material 1 List of all taxa occurring at the CRM seeps and new records in this work Data type xlsx Explanation note For described species: depth range at the CRM, known biogeographic range, known occurrences at vents or organic falls, and new records are indicated. If only a single depth is reported, the depth value is shown as a minimum. Exact collection depths are provided where possible; approximations are indicated by ~. This table summarizes the taxonomic section of the main text, including newly published GenBank sequences, but introduces no new information. https://binary.pensoft.net/file/1213058This dataset is made available under the Open Database License (http://opendatacommons.org/licenses/odbl/1.0/). The Open Database License (ODbL) is a license agreement intended to allow users to freely share, modify, and use this Dataset while maintaining this same freedom for others, provided that the original source and author(s) are credited.Charlotte A. Seid, Avery S. Hiley, Marina F. McCowin, José I. Carvajal, Harim Cha, Shane T. Ahyong, Oliver S. Ashford, Odalisca Breedy, Douglas J. Eernisse, Shana K. Goffredi, Michel E. Hendrickx, Kevin M. Kocot, Christopher L. Mah, Allison K. Miller, Nicolás Mongiardino Koch, Rich Mooi, Timothy D. O’Hara, Fredrik Pleijel, Josefin Stiller, Ekin Tilic, Paul Valentich-Scott, Anders Warén, Mary K. Wicksten, Nerida G. Wilson, Erik E. Cordes, Lisa A. Levin, Jorge Cortés, Greg W. Rouse
